# Structure, Assembly, and Function of Tripartite Efflux and Type 1 Secretion Systems in Gram-Negative Bacteria

**DOI:** 10.1021/acs.chemrev.1c00055

**Published:** 2021-04-28

**Authors:** Ilyas Alav, Jessica Kobylka, Miriam S. Kuth, Klaas M. Pos, Martin Picard, Jessica M. A. Blair, Vassiliy N. Bavro

**Affiliations:** † Institute of Microbiology and Infection, College of Medical and Dental Sciences, University of Birmingham, Edgbaston, Birmingham B15 2TT, United Kingdom; ‡ Institute of Biochemistry, Biocenter, Goethe Universität Frankfurt, Max-von-Laue-Straße 9, D-60438 Frankfurt, Germany; § Laboratoire de Biologie Physico-Chimique des Protéines Membranaires, CNRS UMR 7099, Université de Paris, 75005 Paris, France; ∥ Fondation Edmond de Rothschild pour le développement de la recherche Scientifique, Institut de Biologie Physico-Chimique, 75005 Paris, France; ⊥ School of Life Sciences, University of Essex, Colchester, CO4 3SQ United Kingdom

## Abstract

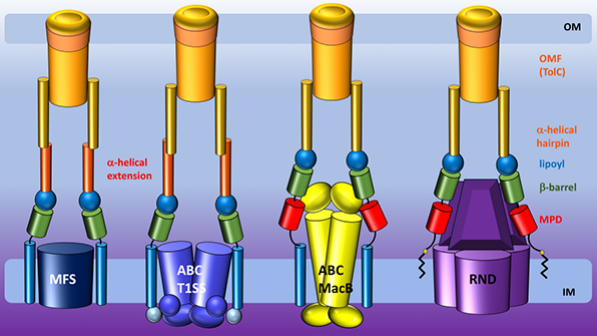

Tripartite efflux pumps and the related type 1 secretion systems (T1SSs) in Gram-negative organisms are diverse in function, energization, and structural organization. They form continuous conduits spanning both the inner and the outer membrane and are composed of three principal components—the energized inner membrane transporters (belonging to ABC, RND, and MFS families), the outer membrane factor channel-like proteins, and linking the two, the periplasmic adaptor proteins (PAPs), also known as the membrane fusion proteins (MFPs). In this review we summarize the recent advances in understanding of structural biology, function, and regulation of these systems, highlighting the previously undescribed role of PAPs in providing a common architectural scaffold across diverse families of transporters. Despite being built from a limited number of basic structural domains, these complexes present a staggering variety of architectures. While key insights have been derived from the RND transporter systems, a closer inspection of the operation and structural organization of different tripartite systems reveals unexpected analogies between them, including those formed around MFS- and ATP-driven transporters, suggesting that they operate around basic common principles. Based on that we are proposing a new integrated model of PAP-mediated communication within the conformational cycling of tripartite systems, which could be expanded to other types of assemblies.

## 1 Introduction: The Problem of Pumping across Two Membranes in Gram-Negative Bacteria

**Figure 1:**
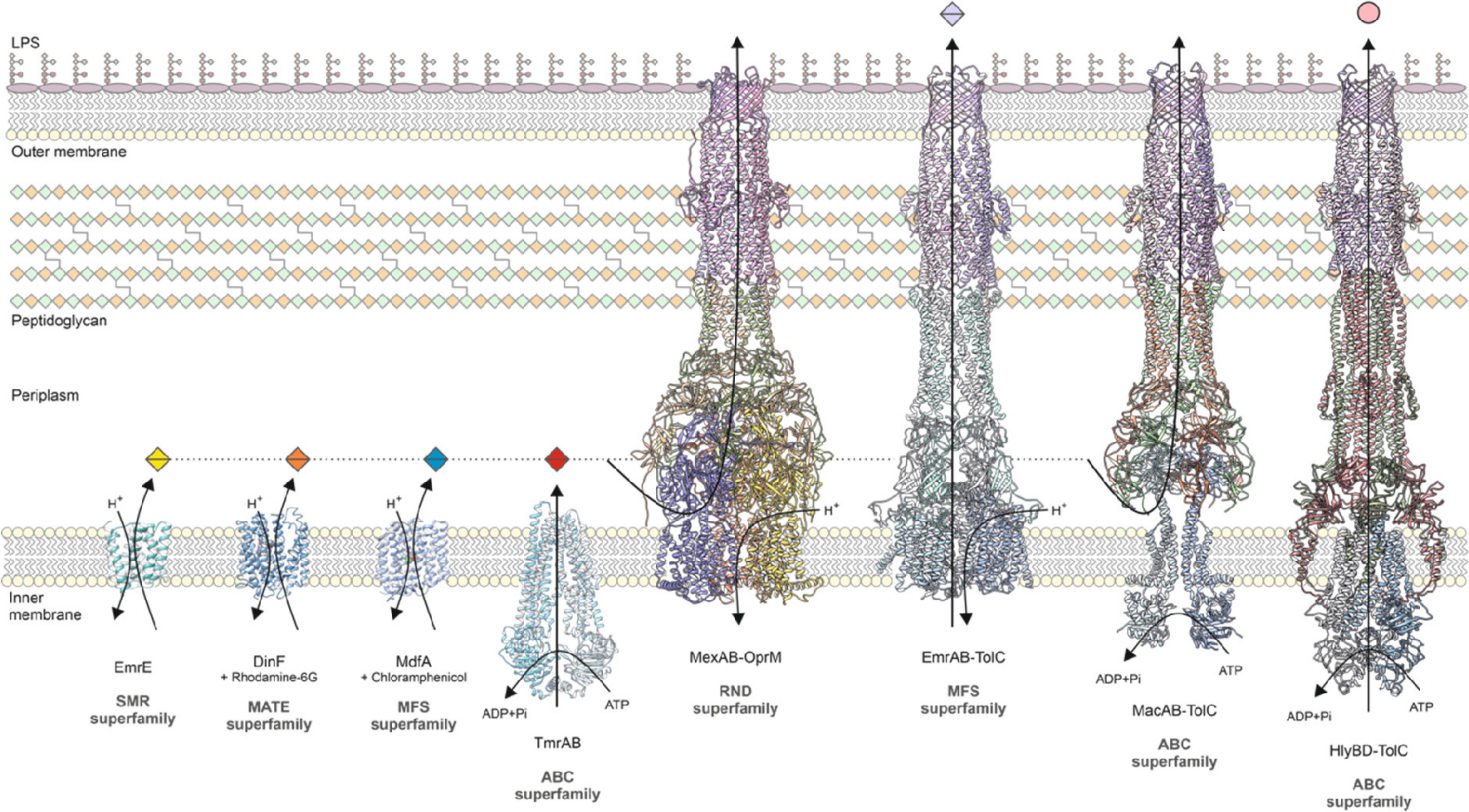
Representative structures of multidrug transporters and tripartite assemblies. Structures of members of the ATP-driven ATP-binding cassette (ABC) superfamily and the proton-motive force-dependent secondary antiporters of the major facilitator superfamily (MFS), multidrug and toxin extrusion (MATE) family, small multidrug resistance (SMR) family, and resistance nodulation-cell division (RND) superfamily are shown. A substrate for each of the transporters is indicated (colored double triangles). The single-component transporters are independent multidrug/H^+^ (or Na^+^) antiporters which transport the drugs from the cytoplasm to the periplasm. The tripartite RND superfamily member MexAB-OprM and the tripartite ABC superfamily member MacAB-TolC are postulated to sequester their substrates from the periplasm (or outer leaflet of the inner membrane) and transport them across the outer membrane. The single component multidrug efflux antiporters and the RND-type tripartite efflux pumps act in a synergistic fashion to efflux drug substrates across both inner and outer membranes. Structures of a proteobacterial antimicrobial compound efflux (PACE) member are elusive and therefore not included in the figure. ABC superfamily members and the 14-transmembrane helix MFS transporters form also tripartite pumps. The ABC superfamily member HlyBD-TolC transports hemolysin from the cytoplasm to the outside of the cell. The ABC-type tripartite MacAB-TolC complex transports macrolide antibiotics, outer membrane glycolipids, lipopeptides, and protoporphyrinand polypeptide virulence factors such as enterotoxin II. The MFS-type tripartite EmrAB-TolC system has been shown to transport several small molecular weight drugs. EmrE Protein Databank (PDB) entry: 3B5D; DinF-BH PDB: 4LZ9; MdfA PDB: 4ZOW; MexAB-OprM EM Data Bank (EMDB) entry: EMD-10395, PDB: 6TA6; EmrAB-TolC adapted from Yousefian et al.;^9^ MacAB-TolC EMDB: EMD-3652, PDB: 5NIK; MacB EMDB: EMD-3653, PDB: 5NIL. The HlyB model (based on PCAT1 (PDB: 4RY2)) and the full assembly of the HlyD-docked homology model are provided by V. Bavro. LPS, lipopolysaccharide.

The cell envelope of Gram-negative bacteria consists of three fundamental layers: the inner or cytoplasmic membrane, the peptidoglycan cell wall, and the outer membrane. The two membrane layers are separated by an aqueous cellular compartment known as the periplasm.^1^ Although the double membrane cell envelope is a sophisticated barrier that affords Gram-negative bacteria protection from various environmental insults, it also presents a biological challenge for transporting molecules out of cells. This has given rise to a plethora of transenvelope transport machinery, namely tripartite efflux systems and the related type 1 secretion systems (T1SSs).^2,3^ Tripartite efflux systems and the related T1SSs consist of a transmembrane inner-membrane transporter protein, a periplasmic adaptor protein (PAP) that spans the periplasm, and an outer membrane factor (OMF) protein that penetrates the outer membrane. This tripartite organization allows Gram-negative bacteria to directly transport molecules across the outer membrane to the extracellular environment. Tripartite efflux systems can be categorized into three superfamilies based on the type of the inner-membrane transporter around which they are built: with multidrug efflux pumps being formed with the participation of transporters belonging to either the ATP-binding cassette (ABC) superfamily, the major facilitator superfamily (MFS), or the resistance-nodulation-division (RND) superfamily, whereas the transporters associated with the related T1SS belong exclusively to the ABC superfamily (Figure 1).^2,4,5^ The T1SSs secrete diverse proteins, many of which are involved in host pathogenesis and virulence.^6^ Tripartite efflux systems play a major role in the multidrug resistance of Gram-negative bacteria, including ESKAPE pathogens such as *Acinetobacter baumannii*, *Enterobacter* spp., *Klebsiella pneumoniae*, and *Pseudomonas aeruginosa*, which have contributed significantly to the increasing rate of antimicrobial resistance (AMR)-related infections.^7^ AMR is a major global public health crisis that undermines not only human health, but also animal health, food security, and development. The severity of this problem has led the World Health Organization to adopt the Global Action Plan on AMR that consists of five strategic objectives to combat AMR, one of which is to stimulate the development of new medicines.^8^ Therefore, to develop new therapeutics to target tripartite efflux systems and T1SSs, we must better understand their physiology and roles in Gram-negative bacteria.

In the sections below, we will discuss the genes, function, and structural organization of tripartite efflux systems and T1SSs. This is a vast topic, which is also reflected in the volume of this systematic review, and to facilitate the reader, we would like to highlight the structure of this manuscript. It is organized into four major divisions, which cover first the biological roles of the tripartite pumps including their contribution to antimicrobial resistance, as well as their genetic organization and regulation (sections 2–4). The second group of sections (5–7) deals with the molecular organization of the individual components of the tripartite complexes, with particular effort being put into highlighting the modularity and similarities in the structural organization of the different transporter groups, notably the ABC and RND families (sections 5.7.6–5.7.7), as well as the unexpected correlations in the organization of the PAP proteins (section 7.7) that may be of interest to structural biologist beyond the immediate field of efflux pumps. The third group of chapters (8–11) focuses on the current knowledge of the mechanisms of assembly of the respective tripartite pumps but also aims to provide a synthesis of the data in the form of a synthetic new model of assembly and functional cycling of the RND-based tripartite pumps (section 8.6), which spotlights the new roles for the PAPs highlighted by the latest research in the field. Insights from this model allow us to underline the communality of the assembly between the RND-, MacB-, MFS-, and T1SS-based systems (sections 9–11). The review concludes with an overview of the current strategies for disruption of the pump function (section 12).

## 2 Involvement of Tripartite Systems in Antibiotic Resistance and Drug Efflux Profiles for Principal Classes of Transporters

Numerous tripartite efflux complexes have been characterized and defined across Gram-negative bacteria (Table 1). The overwhelming majority of tripartite efflux pumps belong to the RND family, although a few pumps in the ABC and MFS families have also been described. Tripartite efflux complexes often recognize and export a range of physicochemically diverse substrates, consisting of some lipophilic (quinolines, macrolides, and glycylcyclines) as well as cationic (aminoglycosides), anionic (β-lactams), and neutral (chloramphenicol) compounds. For instance, AcrAB-TolC is the predominant efflux system in members of Enterobacteriaceae and has a remarkably wide substrate profile, consisting of multiple different classes of antibiotics (chloramphenicols, tetracyclines, quinolones, macrolides, and β-lactams).^10^ Other examples include MexAB-OprM of *P. aeruginosa* and AdeABC of *A. baumannii*, which can also export multiple different classes of antibiotics.^11,12^ Tripartite efflux pumps and their regulatory systems have been shown to be involved in clinically relevant antibiotic resistance. For example, overexpression of the MtrCDE efflux pump due to mutations in *mtrR* have contributed to penicillin resistance in *Neisseria gonorrhoeae*, which has contributed to the inefficacy of older β-lactams in the treatment of gonorrhoeae.^13^ There is now evidence that the efficacy of azithromycin, which is a current first-line treatment option for gonorrhoeae, is also under threat due to overexpression of the MtrCDE pump in clinical isolates.^14^ In another example, overexpression of AcrAB-TolC and MdfA in clinical *Escherichia coli* UTI isolates has been correlated with fluoroquinolone resistance,^15^ severely limiting treatment options. In addition to clinically relevant antibiotics, several tripartite efflux pumps also export commonly used biocides. The AcrAB-TolC pump of *E. coli* can export benzalkonium chloride, chlorhexidine, and triclosan, and *P. aeruginosa* possesses multiple efflux pumps capable of exporting triclosan.^16^ This has serious implications for infection control in a wide range of healthcare settings by jeopardizing the efficacy of important biocides and selecting for cross-resistance to clinically relevant antibiotics.^17^ Tripartite systems also include T1SSs, such as HlyBD-TolC from *E. coli* and LipBCD from *S. marcescens*, which are involved in the export of virulence factors.^18^

**Table 1:** List of Characterized Tripartite Systems in Gram-Negative Bacteria and Their Substrate Profiles^a^

Microorganism	Efflux pump family	Efflux system	Substrates	refs
*Acinetobacter baumannii*	RND	AdeABC	AG, BL, CHL, CHX, ML, FA, FQ, TET, TGC, TMP	(11, 19, 20)
		AdeFGH	CHL, FQ, SUL, TET, TGC, TMP	(21)
		AdeIJK	BL, CHL, CHX, ERY, FA, FQ, NOV, TET, TGC, TMP, TRI	(11)
		AbuO	AG, BL, TGC	(22)
		ArpAB	AG	(23)
	ABC	MacAB-TolC	ML	(24)
*Acinetobacter* Genomic DNA Group 3	RND	AdeDE	CHL, FQ, TET	(25, 26)
		AdeXYZ	CHL, FQ, BL, TET	(25)
*Achromobacter* spp.	RND	AxyXY-OprZ	AG, FQ	(27)
		NccABC	Cd^2+^, Co^2+^, Ni^2+^	(28)
*Aeromonas hydrophila*	RND	AheABC	RIF	(29)
*Aggregatibacter actinomycetemcomitans*	ABC	LtxDB-TolC	LTX	(30)
*Bacteroides fragilis*	RND	BmeABC3	BL, FQ	(31)
*Borrelia burgdorferi*	RND	BesABC	ML	(32)
*Bordetella pertussis*	ABC	CyaBDE	ACT	(33)
*Bradyrhizobium japonicum*	RND	BdeAB-?	AG	(34)
*Brucella suis*	RND	BepDE-BepC	ACR, AMP, DOC, EB, FQ, NOV, PMB, SDS, TET	(35)
		BepFG-BepC	DOC, NAL, SDS	(35)
*Burkholderia cenocepacia*	RND	CeoAB-OpcM	CHL, FQ	(36)
		BCAL2820-BCAL2822	ATM, AG, CHL, FQ	(37)
*Burkholderia pseudomallei*	RND	AmrAB-OprA	AG, ML	(38)
		BpeAB-OprB	AG, CHL, ML, TET	(38, 39)
		BpeEF-OprC	CHL, TET, TMP	(40)
*Burkholderia thailandensis*	RND	AmrAB-OprA	AG, ML, TET	(41)
*Campylobacter jejuni*	RND	CmeABC	CHL, CTX, ML, FA, FQ, TRI	(42, 43)
		CmeDEF	CTX, FQ, TRI	(44)
*Caulobacter crescentus*	RND	NczABC	Co^2+^, Cd^2+^, Ni^2+^, Zn^2+^	(45)
*Cupriavidus metallidurans*	RND	ZneCAB	Zn^2+^	(46)
*Dickeya zeae*	RND	DesABC	ZMN	(47)
Enterobacteriaceae^b^	RND	AcrAB-TolC	ACR, BAC, BL, CHL, EB, ERY, FA, FQ, NOV, OXN, TET, TGC, TRI, RIF, R6G	(10, 48−50, 52)
		OqxAB-TolC^c^	CHL, FQ, ML, NIT, TGC	(53)
*Escherichia coli*	RND	AcrAD-TolC	AG, BL	(54, 55)
		AcrEF-TolC	ACR, BL, CHL, FQs, EB, ERY, R6G, TET, TGC	(10, 56, 57)
		MdtEF-TolC	BL, BAC, ML, OXN	(10, 56−58)
		CusABC	Ag^+^, Cu^+^	(59)
	ABC	MacAB-TolC	ML	(60)
		HlyBD-TolC	AH	(61)
		YhbFGSR-	EB, TET	(62)
		TolC	MJ25	(63)
		YojI?-TolC		
	MFS	EmrAB-TolC	FQ, NOV, TRI	(10, 56)
		EmrKY-TolC	TET	(64)
		MdtNOP	ACR, PUR	(56)
		AaeAB-?	pHBA	(65)
*Helicobacter pylori*	RND	HefABC	AG, BL, CHL, EB, ERY, FQ, NOV, TET	(66)
		HefDEF	Cd^2+^, Ni^2+^, Zn^2+^	(67)
*Klebsiella aerogenes*	RND	EefABC	CHL, BL, ERY, FQ	(68)
*Klebsiella pneumoniae*	RND	AcrAB	BAC, BL, ML, FQ, TET, NOV	(69, 70)
		EefABC	BAC, BL, FQ, ERY, TET, NOV	(70)
		KexEF	BL, ERY, TET, NOV	(70)
		KexD	ML	(71)
		KexVWX	BL, NOV	
		TMexCXD1-TOprJ1^c^	AG, FQ, TGC	(72)
	MFS	KpnGH-TolC	BL, AG, ML	(73)
*Legionella pneumophila*	RND	LpeAB	ML	(74)
*Moraxella catarrhalis*	RND	AcrAB-OprM	CTX, ML	(75)
*Neisseria gonorrhoeae*	RND	MtrCDE	BL, BS, FQ, ML, TET	(76, 77)
	ABC	MacAB-MtrE	ML	(78)
	MFS	FarAB-MtrE	FA	(79)
*Porphyromonas gingivalis*	RND	XepCAB	RIF	(80)
*Pseudomonas aeruginosa*	RND	MexAB-OprM	AG, BL, CHL, COL, EB, FQ, ML, SDS, SUL, TET, TMP, TRI	(12, 81)
		MexCD-OprJ	BL, CHL, CHX, COL, FQ, ML, QAC, TET, TGC, TMP, TRI	(81)
		MexEF-OprN	CHL, FQ, TET, TMP, TRI	(82)
		MexGHI-OpmD	ACR, EB, FQ, TET	(83)
		MexJK-OprM/OpmH	ERY, TET, TRI	(84)
		MexMN-OprM	CHL	(85)
		MexPQ-OpmE	ML, FQ	(85)
		MexVW-OprM	ACR, CHL, EB, ERY, FQ, TET	(86)
		MexXY-OprA/OprM	AG, BL, CHL, FQ, ML, TET, TGC	(81)
		MuxABC-OpmB	ATM, ERY, FQ, NOV, TET	(87)
		TriABC-OpmH	TRI	(88)
*Pseudomonas putida*	RND	ArpABC	BL, CHL, ERY, TET	(89)
		MepABC	BL, ERY, TET	(90)
		ParXY/TtgC	AG, FQ	(91)
		SrpABC	OS	(92)
		TtgABC	BL, CHL, ERY, OS, TET	(93)
		TtgDEF	OS	(94)
		TtgGHI	OS	(95)
*Pseudomonas fluorescens*	RND	EmhABC	CHL	(96)
*Pseudomonas syringae*	RND	PseABC	ACR, ERY, TET	(97)
*Riemerella anatipestifer*	RND	RaeABC	AG, SDS	(98)
		RaeEF-RopN	AG, SDS	(99)
*Salmonella enterica*	RND	AcrAD-TolC	ATM, BL, NOV, SDS	(100)
		AcrEF-TolC	ACR, BAC, BL, CHL, EB, FUS, FQs, NOV, R6G, SDS, TET, TRI	(101)
		MdtABC-TolC	NOV	(101)
		MdsABC	Au^3+^, BAC, BL, CHL, NOV	(102)
		SilABC^c^	Ag^+^	(103)
	ABC	MacAB-TolC	ML	(101)
	MFS	EmrAB-TolC	NAL, NOV, TRI	(101)
*Serratia marcescens*	RND	SdeAB	CHL, FQ, QAC	(104)
		SdeGH	FQ	(105)
		SdePQ	FQ	(105)
		SdeXY-HasF	BAC, CHL, CHX, FQ, ML, QAC, TET, TGC, TRI	(106, 107)
*Sinorhizobium meliloti*	RND	SmeAB-TolC	ACR, CHL, ERY, NAL, RIF, SDS, TET	(108)
		SmeCD-TolC	CHL, NAL	(108)
		SmeEF-TolC	ERY, TET	(108)
*Stenotrophomonas maltophilia*	RND	SmeABC	AG, BL, FQ	(109)
		SmeDEF	CHL, FQ, ML, TET, TRI	(110)
		SmeGH	BL, CHL, FQ, ML, TET	(109, 111)
		SmeIJK	AG, TET	(109)
		SmeOP-TolC	AG, FQ, ML, TET	(112)
		SmeVWX	CHL, FQ, TET	(113)
		SmeYZ	AG, TET	(114)
	MFS	EmrCAB	CCCP, NAL	(115)
	ABC	MacABC	AG, ML, PMB	(116)
		FuaABC	FUA	(117)
		SmaAB	AMK	(118)
		SmaCDEF	LEV	(118)
*Vibrio cholerae*	RND	VexAB- TolC	AMP, ERY, NOV, PEN, PMB, SDS	(119, 120)
		VexCD-TolC	BS, DTG, ERY	(119)
		VexEF- TolC	BAC, DOC, EB, ERY, NOR, NOV, SDS, TET, TMP	(119)
		VexGH	DTG, NOV	(121)
		VexIJK	BS, DTG	(120)
	MFS	VceCAB	CCCP, DOC, NAL	(122)
*Vibrio parahaemolyticus*	RND	VmeAB-VpoC	ACR, BL, BS, EB, FQ, ML, NOV, R6G, SDS, TET, TMP	(123)
		VmeCD-VpoC	BAC, BL, BS, ML, NOV, R6G, SDS	(124)
		VmeEF-VpoC	BC, EB, NOV, R6G, SDS	(124)
		VmeGHI-VpoC	SDS	(124)
		VmeTUV-VpoC	ACR, BL, BAC, BC, CHX, EB, R6G, SDS	(124)
		VmeYZ-VpoC	BC, NOV, SDS	(124)

a ABC, ATP-binding cassette; ACR, acriflavine; ACT, adenylate cyclase toxin; AG, aminoglycosides; AH, α-hemolysin; AMK, amikacin; AMP, ampicillin; ATM, aztreonam; BAC, benzalkonium chloride; BL, β-lactams; BS, bile salts; CCCP, carbonyl cyanide *m*-chlorophenylhydrazone; CHL, chloramphenicol; CHX, chlorhexidine; COL, colistin; CTX, cefotaxime; DOC, deoxycholate; DTG, detergents; ERY, erythromycin; EB, ethidium bromide; FA, fatty acids; FUS, fusidic acid; FQ, fluoroquinolones; FUA, fusaric acid; LEV, levofloxacin; LTX, leukotoxin; MFS, major facilitator superfamily; MJ25, microcin J25; ML, macrolides; NAL, nalidixic acid; NIT, nitrofurantoin; NOR, norfloxacin; NOV, novobiocin; OS, organic solvents; OXN, oxazolidinones; PEN, penicillin; pHBA, *p*-hydroxybenzoic acid; PMB, polymyxin B; PUR, puromycin; QAC, quaternary ammonium compounds; R6G, rhodamine 6G; RIF, rifampicin; RND, resistance-nodulation-division; SDS, sodium dodecyl sulfate; SUL, sulfonamides; TET, tetracycline; TGC, tigecycline; TMP, trimethoprim; TRI, triclosan; ZMN, zeamines.

b A large family of Gram-negative bacteria that includes several important pathogenic species such as *Citrobacter freundii*, *Escherichia coli*, *Klebsiella pneumoniae*, *Salmonella enterica*, and *Shigella flexneri*.

c Pumps that are encoded in plasmids.

## 3 Biological Functions Outside of Multidrug Resistance

Efflux-mediated antibiotic resistance was first discovered in the 1970s by Levy and McMurry,^125^ who showed active efflux of tetracycline from bacterial cells possessing the pBR322 plasmid. Since then, efflux-mediated resistance has been identified for nearly all classes of antibiotics. Although efflux pumps are associated with antimicrobial resistance, they likely existed long before the advent of modern medicine. This is evident from the highly conserved nature of efflux pump genes across bacterial genomes. A recent review by Teelucksingh et al.^126^ found that the majority of *E. coli* efflux pumps are highly conserved and make up 1% of the core genome. This indicates that despite the significant diversity across the *E. coli* strains, efflux pumps have remained relatively stable. Phylogenetic analysis of RND-type transporters by Zwama et al.^127^ revealed that AcrB from *Haemophilus influenzae* is evolutionarily ancient compared to AcrB from *Escherichia coli*. Despite its evolutionary age, AcrB of *H. influenzae* was shown to export the same range of antibiotics as AcrB of *E. coli*. Based on their findings, they speculated that multidrug recognition by RND-type transporters is an ancient trait rather than an evolutionarily acquired ability. A different phylogenetic analysis found that the MdtB and MdtC inner membrane RND transporters are conserved throughout Proteobacteria and that their existence was due to a single gene duplication event before the split of Proteobacteria into the α-, β-, and γ-classes. This also suggests that efflux pump genes have remained relatively stable throughout bacterial evolution.^128^ Similarly, ABC transporters constitute one of the largest transporter superfamilies. In *E. coli*, there are approximately 80 distinct ABC transporters, which represent 5% of the genome.^129^ Many ABC transporters are linked with multidrug resistance; however, it remains unclear how multidrug recognition was acquired. Studies on the resistome of ancient bacteria in archeological samples have also identified multiple different efflux pump genes.^130^ Accordingly, if efflux pumps are so conserved and were present before the age of modern medicine, what are their physiological roles in bacteria? Compared to our knowledge on the role of efflux pumps in antibiotic resistance, our understanding of the biological roles of efflux pumps is lagging. However, studies have suggested increasingly diverse roles for efflux pumps in bacterial physiology.

Genes encoding tripartite efflux systems are ubiquitous among Gram-negative bacteria, including pathogenic and nonpathogenic species, indicating that efflux pumps are evolutionarily ancient with fundamental physiological roles in bacteria.^131^ Studies have demonstrated increasingly diverse roles for efflux pumps in bacterial biology, including virulence, biofilm formation, and quorum sensing (QS). The role of efflux pumps in detoxification and pH homeostasis has been recently reviewed by Teelucksingh et al.^126^ and therefore will not be discussed here. In this section, the role of tripartite efflux systems in virulence, QS, and biofilm formation will be discussed.

### 3.1 Virulence

Tripartite efflux systems have been shown in numerous studies to play an essential role in the ability of Gram-negative bacteria to colonize and disseminate during host infection. These studies have demonstrated that the inactivation or deletion of tripartite efflux components has detrimental effects on virulence (Table 2). Tripartite efflux pump genes are conserved regardless of the host species, highlighting an important role for virulence.

**Table 2:** List of Tripartite Systems That When Deleted or Inactivated Result in Attenuated Virulence in Their Cell/Host Model of Infection^a^

Microorganism	Efflux pump family	Efflux system	Cell/host infection model	ref(s)
*S.* Typhimurium	RND	AcrAB-TolC	Human epithelial cells, murine macrophages, Galleria mellonella, mouse, chicken	(101, 158−160)
		MdtABC	Mouse	(101)
		MdsABC	Mouse	(101)
	ABC	MacAB-TolC	Mouse	(101, 138, 139)
		SiiCDF	Cattle and bovine enterocytes	(156)
*K. pneumoniae*	RND	AcrAB-TolC	Mouse	(51)
*E. coli*	ABC	MacAB-TolC	Galleria mellonella and murine mammary glands	(161)
	RND	MdtB	Mouse spleen	(161)
		MdtEF	Human macrophages	(162)
*A. baumannii*	RND	AdeABC	*Galleria mellonella*	(163)
		AdeIJK	*Caenorhabditis elegans*	(164)
*E. cloacae*	RND	AcrAB-TolC	Mouse	(165)
*F. tularensis*	RND	AcrAB	Mouse	(166)
*N. gonorrhoeae*	RND	MtrCDE	Human neutrophils, mouse	(146, 167)
*P. aeruginosa*	RND	MexAB-OprM	Mouse, canine epithelial cells	(168)
		MexGHI-OpmD	Rat	(83)
*S. maltophilia*	RND	SmeYZ	Mouse	(114)
*B. pseudomallei*	RND	BpeAB-OprB	Human epithelial cells and macrophages	(169)
*B. burgdorferi*	RND	BesABC	Mouse	(32)
*C. jejuni*	RND	CmeABC	Acanthamoeba polyphaga, Chicken	(170)
				(143)
*S. flexneri*	MFS	EmrKY	Human macrophages	(144)
*L. pneumophila*	ABC	LssBD-TolC	Ameoba and human macrophages	(157)
*V. cholerae*	RND	VexAB-TolC, VexCD-TolC, VexIJK	Mouse	(120)
*Riemerella anatipestifer*	RND	RaeEF-RopN	Duck	(99)
*E. amylovora*	RND	MdtABC	Apple rootstock	(147)
		MdtUVW		
		AcrAB		(171)
*R. solanacearum*	RND	AcrAB	Tomato plant	(148)

a ABC, ATP-binding cassette; MFS, major facilitator superfamily; RND, resistance-nodulation-division.

#### 3.1.1 Defense against Host-Derived Molecules

The host environment can be challenging for bacteria due to the presence of various host antimicrobial compounds, such as bile salts, antimicrobial peptides (AMP), and fatty acids.^132^ This especially holds for the mammalian gut, which is enriched with bile salts that confer protection to the gut mucosa against bacteria.^133^ Therefore, tripartite efflux systems are likely to be involved in the extrusion of host innate defense compounds, thereby enabling the survival of bacteria within the host. This is best exemplified by the tripartite efflux system AcrAB-TolC of Enterobacteriaceae, which can actively efflux and confer resistance to bile salts, AMP such as LL-37 and defensin HBD-1, and fatty acids.^134−136^ A recent metabolomics study strongly suggests that oxidized fatty acids are the native substrates for AcrB in both *Salmonella* and *E. coli*.^137^ Additionally, the ABC pump MacAB-TolC has been shown to protect *S*. Typhimurium from oxidative stress. Reactive oxygen species (ROS), such as hydrogen peroxide, are produced by macrophages as a defense mechanism against intracellular pathogens. Bogomolnaya et al.^138^ demonstrated that *macAB* mutants of *S.* Typhimurium were unable to grow in cultured macrophages that produce ROS but grew normally in ROS-deficient macrophages. Furthermore, *macAB* mutants were unable to confer resistance to hydrogen peroxide upon exogenous administration *in vitro*, indicating a role for MacAB in ROS detoxification. Recently, the same group also showed that the linearized enterobactin trimer dihydroxybenzoylserine, an enterobactin metabolite, protects *S.* Typhimurium against peroxide-mediated killing and is a natural substrate of the MacAB pump.^139^ This indicates that the MacAB efflux system plays an important role in the survival of *S.* Typhimurium in the host environment during infection by conferring protection against ROS-mediated oxidative stress. As a pathogen with only one host, *N. gonorrhoeae* is particularly well adapted to infecting humans. The MtrCDE efflux system of *N. gonorrhoeae* has been reported to confer resistance to neutrophil-derived AMP and proteins, indicating that the MtrCDE pump contributes to the defense of *N. gonorrhoeae* against immune cells. The local regulator of the MtrCDE pump, MtrR, has also been shown to bind bile as a physiological inducer, resulting in the depression of *mtrCDE* expression to confer protection from host-derived and clinically relevant antimicrobials.^140^ In a different study, the RND transporter MtrD was shown to export the hormone progesterone and the cationic antimicrobial peptide polymyxin B.^141^ The MFS tripartite pump FarAB-MtrE of *N. gonorrhoeae* can extrude long-chained antibacterial fatty acids, such as linoleic acid, oleic acid, and palmitic acid, thereby enhancing the *in vivo* survival of *N. gonorrhoeae*.^79^ In the related species *N. meningitidis*, the MtrCDE pump has also been reported to confer resistance to human cationic AMP.^142^ Other efflux systems, including VexAB and VexCD of *V. cholerae*, CmeABC of *C. jejuni*, and AcrAB of *S. flexneri*, have also been reported to confer resistance bile salts and AMP.^120,143,144^ In *E. coli*, the AcrAB-TolC and EmrAB-TolC efflux systems can export mammalian steroid hormones, including estradiol, progesterone, and hydrocortisone.^145^ Steroid hormones have been reported to inhibit growth of *N. gonorrhoeae* and *N. meningitidis*; hence, some efflux systems may protect bacteria from such compounds^146^ Recently, the AdeIJK efflux system of *A. baumannii* was shown to export and confer resistance to host antimicrobial fatty acids, such as arachidonic and docosahexaenoic acid.^20^ The plant pathogen *E. amylovora* also requires tripartite efflux systems MdtABC and MdtUVW to cause infection in apple rootstock. Intriguingly, both efflux systems are also involved in resistance to some antimicrobial plant flavonoids, which are produced and commonly used by plants as a defense mechanism against invading pathogens.^147^ The plant pathogen *Ralstonia solanacearum*, the causative agent of bacterial wilt, also requires the RND efflux genes *acrAB* to cause infection. Compared to wild-type, the *acrAB-*deleted mutant strain was significantly less virulent on tomato plants. Furthermore, various plant antimicrobial compounds were found to induce *acrAB* expression, suggesting a protective role for AcrAB against plant antimicrobials.^148^

#### 3.1.2 Export of Virulence Factors and Toxins

Tripartite efflux systems can also contribute to the infection process by extruding virulence factors and toxins that cause damage to the host. Siderophores are virulence factors which are secreted by bacteria to acquire iron and other required metals from their environment. Freely available iron is scarce within the host since it is mostly bound to heme and other circulating proteins; hence, bacteria use siderophores to scavenge for iron to use for intracellular processes.^149^ Several tripartite efflux pumps have been found to secrete siderophores to allow iron acquisition. The VexGH-TolC efflux system of *V. cholerae* can export the siderophore vibriobactin to acquire iron and maintain cellular homeostasis.^150^ In *E. coli*, the AcrAB-TolC, AcrAD-TolC, and MdtABC tripartite efflux systems are required for the secretion of enterobactin, a siderophore with the highest known affinity for iron.^151^ Pyoverdines are another group of siderophores synthesized and secreted by fluorescent Pseudomonads. In *P. aeruginosa*, pyoverdine is exported by an ABC family tripartite efflux system, PvdRT-OpmQ. Furthermore, it can export pyoverdine that has already transported iron into the periplasm and any other pyoverdine–metal complex, suggesting a role for PvdRT-OpmQ in maintaining iron and metal homeostasis.^152^*S. marcescens* employs the T1SS HasDEF/TolC to secrete the heme-binding protein HasA, which can bind here heme and also acquire heme from host hemoglobin.^153^ Toxins are another virulence factor which are exported by tripartite efflux systems. The ABC family pump MacAB-TolC has been reported to be involved in the secretion of enterotoxin II from the periplasm into the extracellular environment in *E. coli*.^154^ MacAB-TolC is also involved in the export of protoporphyrin, the immediate heme precursor. It has been proposed that MacAB-TolC may play a role in maintaining protoporphyrin levels to avoid excess accumulation, which can be toxic to bacteria since it can degrade to ROS in the presence of oxygen.^155^ The *V. cholerae* RND transporter VexH of the VexGH-TolC_Vc_ efflux system has been reported to be involved in the production of the cholera toxin, although its role in secretion has not been determined.^121^

Type 1 secretion systems (T1SSs) are another class of tripartite complexes involved in toxin secretion. T1SSs play a critical role in the virulence of these pathogens by helping to disseminate toxins to host cells. HlyBD-TolC is a T1SS found in pathogenic *E. coli* strains involved in the secretion of the α-hemolysin (HlyA) toxin. Hemolysins plays a major role in the virulence of pathogenic *E. coli* by forming pores on the surface of host cells, such as erythrocytes, thereby causing lysis. This allows bacterial cells to penetrate mucosal barriers and infect effector immune cells to prevent clearance of infection. Another important T1SS in *E. coli* is CvaAB-TolC, which is responsible for exporting colicin V (CvaC), a pore-forming toxin.^6^ As an important human pathogen, *B. pertussis* employs the T1SS CyaBDE to export the adenylate cyclase toxin (CyaA) to colonize host cells.^33^ In *S. enterica*, the SiiCDF T1SS is involved in the secretion of the large nonfimbrial adhesin SiiE. Deletion of either *siiE* or *siiF* results in attenuated colonization in cattle and invasion of bovine enterocytes.^156^ LssBD-TolC is a T1SS from *L.* pneumophila, which has been reported to be important for the secretion of the RtxA toxin.^157^

### 3.2 Quorum Sensing

Quorum sensing (QS) is a process of intercellular communication, wherein bacteria synthesize, secrete, and respond to extracellular signaling molecules known as autoinducers (AI) to modulate gene expression according to cell population density. Therefore, QS allows bacteria to only express genes involved in energetically costly behaviors once the conditions are deemed necessary, for instance during host infection. Bacteria use the concentration of AI in their environment to monitor changes in cell density and modulate the expression of quorum-specific genes. QS has been reported to regulate a wide range of bacterial processes and behaviors, such as antibiotic production, biofilm formation, motility, sporulation, and virulence.^172^ In Gram-negative bacteria, acylated homoserine lactones (AHL) are predominantly used as AI, whereas Gram-positive bacteria use processed oligopeptides. To exert their effect, AHL must be secreted from the cytosol of Gram-negative bacteria to the external environment.^173^

Tripartite efflux systems have been reported to be involved in the export of AHL in some Gram-negative bacteria, thereby contributing to QS. The MexAB-OprM efflux system of *P. aeruginosa* actively exports a class of AHL known as 3-oxo-acyl-homoserine lactones (HSL) of different acyl chain lengths.^174^ Furthermore, MexAB-OprM also influences the accessibility of noncognate HSLs to LasR, the intracellular receptor for HSLs in *P. aeruginosa*.^175^ This suggests a role for MexAB-OprM in regulating the QS response. A recent study reported that in MexAB-OprM overproducing *P. aeruginosa* mutant strains, the observed impairment in the QS response was due to the impaired synthesis of alkyl quinolone QS signals, likely due to the decreased availability of a precursor molecule, such as octanoate.^176^ The MexEF-OprN efflux system has been shown to export the precursor of the AI *Pseudomonas* quinolone signal (PQS), which is important for the virulence of *P. aeruginosa*.^177^ By exporting an AI precursor, MexEF-OprN may play a role in modulating *P. aeruginosa* virulence by limiting the intracellular availability of AI precursors. In the phytopathogen *P. syringae*, MexEF-OprN was also reported to act as a negative determinant of AHL production and accumulation, suggesting that it may also export AI precursors in other Pseudomonads to regulate AHL production.^178^ Interestingly, overexpression of the MexCD-OprJ efflux system in *P. aeruginosa* has been reported to reduce the QS response. Additionally, the MexXD-OprJ pump extrudes kynurenine, a precursor of alkyl-quinolone signals, such as PQS.^179^ Similar to MexEF-OprN, the MexCD-OprJ pump may also play a role in regulating QS by modulating the intracellular levels of PQS precursors and thus PQS production. Although MexEF-OprN and MexCD-OprJ are inducible efflux systems, they seem to be involved in the QS response. This can be puzzling within the context of an antibiotic-free host environment, which can be considered noninducable. However, RND efflux pumps are induced not just by antibiotics but also by host-derived compounds, such as bile and fatty acids,^180^ and possibly the host immune response.^144^ In *B. pseudomallei*, the BpeAB-OprB efflux system is involved in the active efflux of several different types of AHL. Besides, inactivation of *bpeAB-oprB* in *B. pseudomallei* results in complete inhibition of QS and diminished virulence, indicating a critical role for this efflux system in the physiology of *B. pseudomallei*.^181^ In *E. coli*, the AcrAB-TolC efflux system may also be involved in the efflux of AI. A study by Yang et al.^182^ suggested that AcrB-TolC efflux may facilitate more efficient efflux of AI, although the identity of the AI was not determined. These studies suggest a role for tripartite efflux systems in QS; however, further research is required to elucidate their role in regulating QS.

### 3.3 Biofilm Formation

A biofilm is an aggregation of bacteria that is enclosed in a self-produced matrix of extracellular polymeric substances and commonly attached to a surface and/or to each other. Bacteria within a biofilm display an altered phenotype concerning growth rate and gene transcription to reflect the biofilm lifestyle, which differs significantly from planktonic bacterial cells. The biofilm matrix is a highly diverse environment, consisting of polysaccharides, lipids, proteins, nucleic acids, and even inorganic materials, such as mineral crystals and clay particles. The matrix plays a critical role as a structural scaffold to maintain the integrity of the biofilm. Importantly, biofilms are characterized by heterogeneity, such as oxygen, nutrient, pH, and QS gradients, as well as social interactions, consisting of microbial communication, cooperation, and competition.^183^ Bacteria form biofilms in response to a wide range of environmental and mechanical signals, which can trigger a host of processes, including QS, to promote biofilm formation.^184^

Studies suggest several roles for tripartite efflux systems in biofilm formation, including export of harmful metabolic intermediates, export of extracellular polymeric substances, export of AI to modulate biofilm formation, and export of protein factors to enable adhesion.^185^ Inactivation of genes encoding tripartite efflux systems can result in impaired biofilm formation. Kvist et al.^186^ demonstrated that inactivation of efflux pumps in *E. coli* caused impaired biofilm formation. Specifically, the *aaeX*-deleted *E. coli* mutant exhibited significantly lower biofilm formation.^186^ The *aaeX* gene encodes a membrane component of the MFS efflux system AaeAB, which has been previously reported to be involved in the efflux of toxic metabolic intermediates.^65^ The *emrY* gene, encoding a component of the MFS efflux system EmrKY-TolC, has been reported to be important for the growth of *E. coli* biofilms.^187^ Matsumura et al.^188^ reported that the inactivation of all genes encoding for the components of tripartite efflux systems in *E. coli* resulted in significantly reduced biofilm formation. In particular, *acrD-, acrE-, emrK-*, and *mdtE*-deleted mutants displayed extremely lower biofilm formation. In agreement with this study, Baugh et al.^189^ reported that *acrB-, acrD-, acrEF-, mdtABC-, mdsABC-, emrAB*-, and *macAB*-deleted *S.* Typhimurium mutants also exhibited significantly reduced biofilm formation. These studies suggest that tripartite efflux systems contribute to biofilm formation. In *E. coli*, inactivation of *emrK* or *emrY* has been found to significantly impair survival of cells in response to physical and chemical stresses, including UV radiation, mitomycin C, and hydrogen peroxide.^190^ Therefore, the EmrKY-TolC system may protect cells by exporting toxic metabolic intermediates induced by environmental stresses. A different study found that the EmrB and MdtB tripartite efflux components contribute to extreme acid survival in *E. coli*.^191^ The MdtEF-TolC efflux system has been shown to protect *E. coli* against nitrosative damage in anaerobic conditions. Specifically, the *mdtEF*-deleted mutant was found to grow significantly more slowly during anaerobic respiration of nitrate.^192^ This suggests MdtEF-TolC plays an important role in detoxifying cells in anaerobic conditions by exporting harmful nitrosyl indole derivatives. Several *E. coli* cellular metabolites have been found to induce the expression of the *acrAB* operon, possibly by inactivating *acrR* and/or upregulating *marA* and *soxS* expression. Thus, it is possible that the AcrAB-TolC system exports toxic metabolites and/or signaling molecules to maintain homeostasis of gene expression.^193^ T1SSs have also been reported to be involved in biofilm formation. The T1SS LapEBC from *P. fluorescens* is involved in the secretion of LapA, a biofilm-promoting adhesin, into the extracellular environment to enable bacterial cells to adhere to surfaces. Additionally, mutant strains lacking *lapA*, *lapB*, or *lapE* have been shown to display impaired biofilm formation and maturation.^194^

How might tripartite efflux systems contribute to biofilm formation? As described above, the biofilm environment is heterogeneous, with the biofilm core being more anoxic, acidic, and nutrient-deprived than the biofilm surface.^183^ This can exert pressure and stress on cells within the biofilm core; hence, tripartite efflux systems may contribute to their survival. There is some evidence that expression of some efflux pumps is induced by stress within the biofilm core. De Kievit et al.^195^ showed that expression of *mexAB-oprM* and *mexCD-oprJ* in *P. aeruginosa* was the highest in the bottom of the biofilm, near the solid substratum. These efflux systems may also extrude QS molecules to regulate QS, thereby also regulating the expression of biofilm-specific genes. Some tripartite efflux systems like AcrAB-TolC and AaeAB may play roles in exporting harmful metabolic intermediates to prevent toxic accumulation within biofilm cells. Owing to their role in biofilm formation, tripartite efflux systems could be attractive drug targets to inhibit biofilm formation in clinical settings.

### 3.4 Other Reported Biological Roles

The multifunctional OMF protein TolC is also involved in colicin E1 import^196^ and is employed by bacteriophage TLS as a cell surface receptor to enter *E. coli* cells.^197^ Similarly, in *Salmonella* Typhimurium, the TolC appears to be essential for the infection by the Chi-phage.^198^ Likewise, TolC from *V. cholerae* is also utilized by the VP3 phage to gain entry into cells.^199^ The AcrEF-TolC complex from *E. coli* has been posited to play a role in the maintenance of normal cell division. Lau and Zgurskaya^200^ reported that AcrEF-deficient cells exhibited defective chromosome condensation and segregation during cell division. Furthermore, the absence of AcrEF was also found to result in cell filamentation. The SmeIJK efflux system of *S. maltophilia* has been reported to contribute to cell envelope integrity and the envelope stress response. Compared to wild-type, the *smeIJK*-deleted mutant strain was found to exhibit increased sensitivity to membrane damaging agents and elevated RpoE-mediated envelope stress response. Additionally, sublethal concentrations of membrane damaging agents were reported to induce *smeIJK* expression in a RpoE-dependent manner.^201^ In *A. baumannii*, the RND transporter AdeJ has been reported to play a role in surface-associated motility.^164^ Similarly, the AcrD RND transporter from *S. enterica* has also been shown to play a role in swarming motility.^202^ There is also some evidence that tripartite efflux pumps play a role in lipid modulation, which could potentially effect resistance to membrane targeting antibiotics. In *P. fluorescens*, the RND pump EmhABC has been found to be involved in the efflux of fatty acids. Furthermore, changes in the growth temperature altered the composition of fatty acids in the membrane and significantly increased expression of *emhABC*, with increased extracellular fatty acids. This suggests that EmhABC is involved in the efflux of fatty acids that are replaced due to membrane damage or phospholipid turnover.^203^ The AdeIJK RND pump of *A. baumannii* has also been implicated in membrane modulation. Jiang et al. found that the AdeIJK pump can export the ω-6 fatty acid arachidonic acid and the ω-3 fatty acid decosahexaenoic acid. Furthermore, lipid analyses showed that a*deJ* deletion resulted in significant changes in endogenous lipid concentrations.^20^ The role of efflux pumps in lipid modulation could act as a defense mechanism against membrane targeting antibiotics, which has been previously shown in *Staphylococcus auereus* as a daptomycin resistance mechanism.^204,205^

In nitrogen-fixing symbiotic bacteria, tripartite efflux systems have been reported to play various roles in nitrogen-fixation and nodulation. In *B. japonicum*, the RND efflux system BdeAB is crucial for symbiotic nitrogen-fixation of the soybean plant. The *bdeAB-*deleted *B. japonicum* mutant strain has been shown to exhibit strongly diminished nitrogen fixation, which was evident by discolored plant leaves, a typical sign of nitrogen starvation.^34^ In *S. meliloti*, the RND system SmeAB-TolC plays a role in nodulation competitiveness by protecting cells from antimicrobial compounds produced by the host plant.^108^ In the filamentous cyanobacterium *Anabaena* sp. PCC 7120, the tripartite ABC systems DevBCA-HgdD and HgdBCD have been shown to play critical roles for envelope formation in heterocysts, which are specialized nitrogen-fixing cells.^206,207^ These studies suggest an increasingly important and diverse role of tripartite efflux systems in bacterial physiology.

## 4 Genes and Regulation of Expression

**Figure 2:**
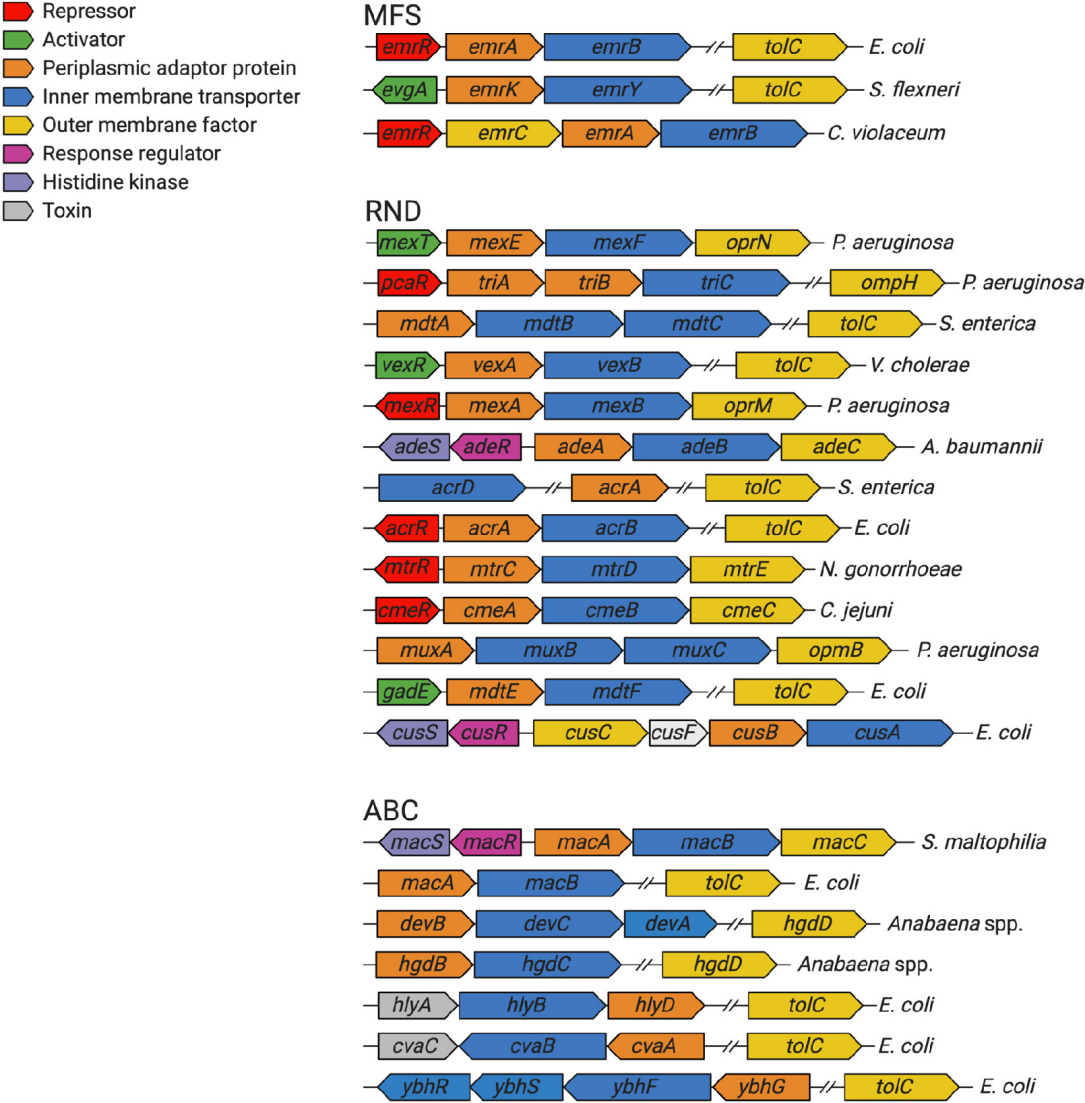
Diverse genetic organization of tripartite efflux systems across a representative sample of Gram-negative bacteria. ABC, ABC-binding cassette; MFS, major facilitator superfamily; RND, resistance-nodulation-division.

Genes encoding tripartite efflux systems are generally organized as operons, wherein the inner membrane tranporter and the PAP genes are located within the same operon (Figure 2). The OMF gene can be encoded within the same operon, such as *oprN* of *P. aeruginosa*,^82^ or elsewhere in the genome, such as *tolC* of *E. coli* and *S. enterica*.^208^ The majority of tripartite efflux systems also have a gene encoding for a local regulator protein within the operon, such as *acrR* and *mexR*, which typically act to repress efflux gene expression.^209,210^ However, a few local regulators can activate efflux gene expression, such as *mexT* and *vexR*, which activate expression of the *mexEF-oprN* and the *vexAB* efflux genes, respectively.^82,211^ Some tripartite efflux systems, such as MacABC of *S. maltophilia* and AdeABC of *A. baumannii*, are regulated by two-component systems (TCS), which are encoded divergently upstream of the efflux pump operon.^116,212^ Certain tripartite efflux systems also have more than one type of PAP or inner membrane transporter encoded within their operon. For example, the *triABC* system of *P. aeruginosa* comprises two PAP genes *triA* and *triB*,^88^ and the *mdtABC* system of *S. enterica* possesses two inner membrane transporter genes *mdtB* and *mdtC*.^213^ T1SSs are also usually organized as operons, with the cognate toxin gene located within the same operon as the efflux pump genes. For example, the *hlyA* gene encoding the α-hemolysin toxin is found upstream of the *hlyB* and *hlyD* genes.^214^ As an extreme example, the genes encoding the AcrAD-TolC efflux system are all located in different genetic loci within the genome.^100^ Therefore, there is significant diversity in the genetic organization of tripartite efflux systems (Figure 2).

Efflux pump overexpression frequently contributes to clinically relevant levels of antibiotic and biocide resistance. Therefore, it is critical to understand how tripartite efflux pumps are regulated to elucidate the mechanisms of overexpression, which could be used to identify novel drug targets to prevent or reverse efflux pump overexpression.^215^ The regulation of tripartite efflux systems can vary between species and even within a species depending on environmental and physiological factors. Normally, efflux pumps are expressed at basal levels through tight regulation of highly complex and interconnected systems. Therefore, the deletion or inhibition of one efflux pump can affect the expression of other pumps. For example, deletion of *acrAB* in *S.* Typhimurium results in significant upregulation of many other genes encoding for tripartite efflux systems, including *acrD, acrF, mdsB, mdtB, macA*, and *emrA*.^216^ Hence, the complexity of efflux pump regulatory networks can often cloud resistance phenotypes.

### 4.1 Local Regulation

**Figure 3:**
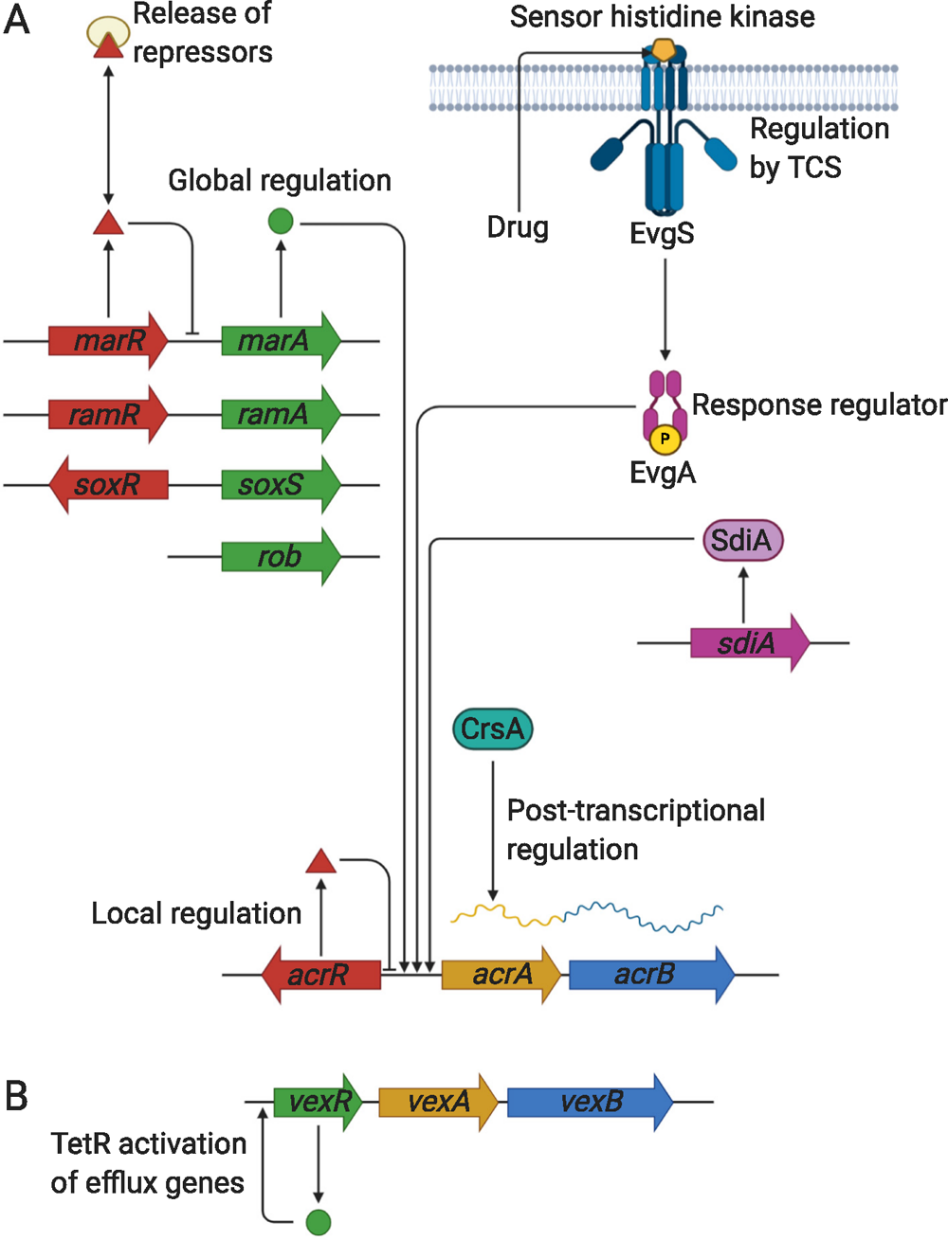
Schematic diagram of the different mechanisms of tripartite efflux pump regulation. (A) Local regulation usually involves a TetR family transcriptional regulator, such as AcrR, that acts to locally repress efflux gene expression and maintain basal levels of expression. Global regulation generally involves AraC/XylS family transcriptional regulators, such as MarA, RamA, and SoxS, that activate efflux pump gene expression. These global regulators themselves are locally regulated by their own TetR family transcriptional regulators, such as MarR, RamR, and SoxR. The presence of antibiotics or other external stressors can release these repressors to allow activation of efflux gene expression. Other types of regulators, such as the quorum sensing regulator SdiA, can also activate efflux gene expression. Two component systems (TCSs) consist of a sensor histidine kinase (EvgS) that detects external stimuli, such as drugs, and phosphorylates a response regulator (EvgA), which becomes activated to trigger efflux pump gene expression. Post-transcriptional regulation includes RNA-binding proteins, such as CrsA, that stabilize efflux gene mRNA to promote efficient efflux protein translation. (B) In V. cholerae, the TetR family transcriptional regulator VexR activates expression of the vexAand vexB efflux pump genes.

Genes encoding for components of tripartite efflux systems are regulated by local and global transcriptional regulators that act to maintain baseline expression (Figure 3). These regulators respond to a wide range of environmental signals, including antibiotic exposure and iron limitation, to increase the expression of efflux pump genes.^215^ The majority of the genes encoding tripartite efflux systems are arranged in an operon, which also contains a divergently oriented gene encoding for a local TetR family regulator (TFTR). TFTRs generally consist of a N-terminal DNA binding domain, which recognizes and binds to a palindromic DNA sequence located in the intergenic region between the regulator and the regulated gene, and a larger C-terminal domain, which is responsible for ligand binding.^217^ Functioning as a dimer, TFTRs act to locally repress the expression of the efflux genes.^218^ Examples of local TFTRs include AcrR, which represses expression of *acrAB* in several different members of Enterobacteriaceae;^209^ MtrR, which represses expression of *mtrCDE* in *N. gonorrhoeae*;^219^ and CmeR, which represses the expression of *cmeABC* in *C. jejuni*.^220^ Binding of ligands to the C-terminal domain of TFTRs induces conformational changes in their structure, thus preventing their binding to target DNA.^217^ For instance, rhodamine 6G, a substrate of the AcrAB-TolC pump, can bind to AcrR and prevent its binding to target DNA, thereby resulting in derepression and expression of *acrAB*.^209^ Mutations conferring loss-of-function of local repressors can result in overexpression of tripartite efflux systems. For example, mutations in *acrR* can lead to overexpression of AcrAB and have been documented in the fluoroquinolone resistance of clinical *E. coli*(221) and *K. pneumoniae*(222) isolates. Likewise, in clinical isolates of *N. gonorrhoeae*, mutations in *mtrR* have been reported to contribute to decreased azithromycin^223^ and cephalosporin susceptibility.^224^

In contrast to trans-acting mutations, cis-acting mutations often have a far greater impact on efflux gene expression. Cis-acting mutations mainly consist of changes in the promoter region of efflux pump genes, whereas trans-acting mutations abrogate repressor activity. For example, a 13-bp inverted-repeat sequence between the divergent *mtrR* and *mtrC* genes in *N. gonorrhoeae*, corresponding to the promoter regions of *mtR* and *mtrC*, has been shown to significantly increase *mtrC* expression and MtrC protein levels, resulting in increased resistance to antiubiotics, detergents, and bile salts.^76^ It has also been found that a 1-bp deletion in a 13-bp inverted repeat within the *mtrR* promoter region is prevalent in 80% of *N. gonorrhoeae* isolates with increased resistance to antimicrobials, bile salts, detergents, and host-derived compounds. These cis-acting mutations impair transcription of *mtR* and promote transcription of *mtrCDE*, even more so than loss of MtrR.^14^

However, negative regulation of tripartite efflux systems is now known to be diverse. Contrasting the conventional negative regulation of the *acrAB* operon, studies have shown that multiple regulators can function as a network to regulate the same efflux pump gene and that non-TFTRs can also regulate efflux pump genes. For example, the *mexAB-oprM* operon in *P. aeruginosa*, which encodes for the MexAB-OprM efflux system, is repressed by the divergently encoded MarR-family protein MexR. Yet, *mexAB-oprM* expression is also repressed by the TFTRs NalC and NalD, which are encoded elsewhere in the *P. aeruginosa* genome.^225,226^ Recently, two additional repressors of the *mexAB-oprM* operon, MdrR1 and MdrR2, have been characterized.^227^ Together, these regulators work as a network to maintain the constitutive basal levels of MexAB-OprM efflux pump expression. Furthermore, not all TFTRs function to repress efflux pump genes. For instance, the RND pump VexAB of *V. cholerae* is organized in an operon with a divergently encoded TFTR VexR, much like AcrAB. However, VexR does not repress *vexRAB* but rather is necessary for the activation of *vexRAB* expression in response to noxious substances (Figure 3).^211^ Notably, some TFTRs are now understood to be much more than simply regulators of efflux pump genes. For example, in addition to repressing the *cmeABC* operon, the TFTR CmeR of *C. jejuni* has been found to regulate the expression of many other genes encoding transport proteins and enzymes involved in capsular polysaccharide biosynthesis. Furthermore, loss-of-function mutations in *cmeR* were shown to diminish the ability of *C. jejuni* to colonize chickens.^228^ In *N. gonorrhoeae*, the TFTR MtrR has been shown to modulate the expression of least 69 genes, which encode for proteins involved in metabolism, general stress, and heat shock response.^229^ Therefore, it has become increasingly evident that TFTRs possess further physiological roles within bacteria.

### 4.2 Global Regulation

Global regulation of tripartite efflux systems is mediated chiefly by the AraC/XylS family of transcription factors. These global regulators are involved in the transcriptional regulation of many processes inside cells, including induction of efflux pump gene expression. In Enterobacteriaceae, characterized AraC/XylS transcription factors include MarA, SoxS, and Rob, all of which can activate expression of the *acrAB* operon in response to harmful substances or stressors (Figure 3).^230^ In *K. pneumoniae*, in addition to *acrAB*, RamA has also been recently shown to upregulate the expression of *oqxAB*.^231^ These regulators recognize and bind to a degenerate approximately 20 bp sequence, which is located in the promoter sequence of *acrAB* and many other genes.^232^ The majority of AraC/XylS transcription factors are encoded alongside their local repressor. For example, MarA, SoxS, and RamA are autoregulated by their local repressors, *marR, soxR*, and *ramR*, respectively (Figure 3).^233^ AraC/XylS family transcriptional regulators involved in efflux pump induction also exist in members of other families, including MtrA of *N. gonorrhoeae* which induces expression of *mtrCDE*.^234^ Repression of global transcriptional regulators is relieved through binding of ligands to the local repressor proteins, which prevents their binding to DNA. For instance, binding of ligands, such as ethidium bromide, rhodamine 6G, and crystal violet, to RamR reduces its binding affinity to target DNA, thus resulting in increased expression of *ramA*.^235^ However, the AraC/XylS family is not the only family of transcription factors involved in the regulation of efflux genes. SdiA is a LuxR-type transcription factor involved in the regulation of cell division through quorum sensing. In addition, SdiA has also been shown to be involved in the positive regulation of AcrAB protein levels (Figure 3).^236^ The T1SS HlyBD of *E. coli* is regulated by the transcription antitermination protein RfaH, which enhances *hlyCABD* transcript elongation. Additionally, RfaH has been found to increase the efficacy of the HlyA toxin protein.^237^ In *S. enterica*, RfaH also regulates the *siiCDEF* operon encoding for the SiiCDEF T1SS and the large nonfimbrial adhesin SiiE.^238^

The increased expression of global transcriptional regulators leads to the induction of efflux pump gene expression. Therefore, mutations that result in derepression of global transcriptional regulators can trigger the overexpression of tripartite efflux pumps. For example, reduced tigecycline susceptibility in clinical *K. pneumoniae* isolates has been associated with loss-of-function mutations in *ramR*, resulting in increased *ramA* and *acrAB* expression.^239^ Mutations in the promoter sequence of global transcriptional regulators can also lead to overexpression by preventing binding of local repressor proteins. Baucheron et al.^240^ demonstrated that mutations in the promoter sequence of *ramA* can prevent binding of RamR. Furthermore, they showed that the MDR phenotype of a clinical *E. coli* isolate was due to a 2-bp deletion in the *ramA* promoter sequence, which compromised RamR-mediated repression and increased *acrAB* and *tolC* expression. Interestingly, a study that investigated the differential impact of *ramRA* mutations in clinical MDR *S.* Typhimurium isolates found that mutations in the promoter region of *ramA* had a greater impact in increasing expression of *ramA* than loss-of-function mutations in *ramR*.^241^ There are also reports of novel mutations that trigger overexpression of global transcriptional regulators. For instance, a study found that in clinical MDR *E. coli* isolates, overexpression of the SoxS protein was not due to mutations in *soxR* or the promoter region of *soxS* but instead a novel alanine to serine substitution (A12S) mutation in *soxS*. Furthermore, overexpression of *acrB* was reported with increased resistance to fluoroquinolones, chloramphenicol, and tetracycline.^242^ Considering that the position of the substitution is not within the DNA binding region of SoxS, the mechanism by which this mutation results in *soxS* overexpression remains to be elucidated. Lastly, overexpression of global transcriptional regulators has been reported to increase mutation rate and frequency. A recent study reported that *ramR* mutants, which overproduce RamA, and thus AcrB, exposed to ciprofloxacin selected for quinolone-resistant *gyrA* mutants. Furthermore, inactivation of *ramA* was found to select for *soxR* mutants, which overproduce SoxS, suggesting a compensatory role for global transcriptional regulators.^243^

Although antibiotics are known to induce expression of efflux pumps, worryingly, nonantibiotic drugs have also been shown to trigger efflux pump overexpression. One study found that upon exposure to the antidepressant fluoxetine, *E. coli* K-12 developed significantly increased resistance to ampicillin. chloramphenicol, amd tetracycline. This was shown to be due to ROS-mediated mutations in the efflux pump regulators *marR* and *rob*, resulting in increased AcrAB-TolC and MdtEF-TolC expression.^244^ Several commonly used biocides have also been documented to trigger efflux pump overexpression. Exposure to subinhibitory concentrations of benzalkonium chloride has been reported to promote antibiotic resistance in *P. aeruginosa* due to mutations in *nfxB*, resulting in overexpression of MexAB-OprM and MexCD-OprJ efflux pumps thereby increasing benzalkonium chloride and ciprofloxacin resistance.^245^ In agreement with this, a recent study also reported the overexpression of the MexCD-OprJ efflux pump in *P. aeruginosa* upon exposure to benzalkonium chlrodide, resulting in increased ciprofloxacin and tetracycline resistance.^246^ Several other studies have also reported similar findings in *P. aeruginosa*, pointing to a link between biocide exposure and cross-resistance to clinically relevant antibiotics.^247,248^ Similarly, triclosan exposure has also been reported to promote significantly high levels of cross-resistance to other antibiotics, including ciprofloxacin, tetracycline, and erythromycin, in *P. aeruginosa*. The triclosan resistant mutants were found to harbor mutations in *nfxB*, the local repressor gene of the *mexCD-oprJ* operon, which resulted in the overexpression of the MexCD-OprJ plasmids carrying antibiotic resistance genes.^249^ Worryingly, the presence of biocides in the environment can also select for MDR. Amsalu et al.^250^ found that 19% of *P. aeruginosa* isolates collected from diverse ecological niches exhibited MexAB-OprM overexpression due to mutations in the efflux pump regulators MexR, NalC, or NalD, resulting in MDR.

Owing to their role as global transcriptional regulators, MarA, SoxS, Rob, and RamA have been shown in several studies to have wider roles in bacterial physiology. For example, MarA, SoxS, Rob, and RamA have been reported to repress flagellar gene expression and motility in *S.* Typhimurium. Specifically, each of these global regulators was found to directly interact with the promoter of *flhDC*, the master regulator of motility in *Salmonella*.^251^ In *E. coli*, MarA has been demonstrated to regulate genes involved in DNA repair and lipid trafficking. Identified MarA targets include the *xseA* gene and the *mlaFEDCB* operon, which encode an endonuclease subunit and a lipid trafficking ABC transporter complex, respectively.^252^ Lastly, the elucidation of the RamA regulon in *K. pneumoniae* found that RamA binds directly to the promoters of *lpxC, lpxL-2*, and *lpxO* genes, which are involved in lipid A biosynthesis. This suggests that RamA plays a role in the modification of the lipid A moiety of lipopolysaccharide (LPS). Furthermore, overexpression of RamA was found to reduce *K. pneumoniae* adhesion and uptake into macrophages, thus conferring protection against the innate host response.^253^ Together, these results signify that in addition to their role in MDR, regulators of tripartite efflux systems also play a wider physiological role in bacteria.

### 4.3 Post-transcriptional Regulation

In contrast to transcriptional regulation, less is known about the regulation of tripartite efflux systems at a post-transcriptional and translational level. However, recent studies have demonstrated that post-transcriptional regulation is likely to play an important role in fine-tuning efflux pump expression. One mechanism of post-transcriptional regulation is through the action of RNA-binding proteins, which are seen as increasingly important modulators of protein function.^254^ The RNA-binding protein CsrA (Carbon Storage Regulator A) acts as a global regulator of a wide range of genes. Ricci et al.^255^ reported that CsrA binds to and stabilizes *acrAB* mRNA, presumably through preventing the formation of repressive RNA structures that hinder ribosome binding (Figure 3). Furthermore, deletion or mutagenesis of *crsA* was shown to decrease the production of AcrAB. Therefore, CsrA may be involved in facilitating the efficient translation of AcrAB. Another study reported that Hfq, an RNA-binding protein and RNA chaperone, may also be involved in the post-transcriptional regulation of AcrB. Deletion of *hfq* in *E. coli* does not affect the transcription of the *acrAB* operon; however, it does diminish the levels of AcrB protein production and increase susceptibility to antibiotics.^256^ In a different study, SdsR, a noncoding small RNA expressed at the stationary phase, was shown to repress the expression of *tolC* by binding to the region upstream of its ribosomal binding site. Furthermore, overexpression of SdsR was found to result in the loss of *tolC* mRNA, a reduction in TolC protein production, and increased susceptibility to some AcrAB-TolC substrates, including novobiocin and crystal violet.^257^ The role of SdsR-mediated *tolC* repression has also been reported in *S.* Typhimurium.^258^

### 4.4 Regulation by Two-Component Systems

Bacteria can adjust their physiological behavior in response to environmental changes through two-component systems (TCSs). In essence, TCSs mediate signal transduction by enabling bacteria to sense external stimuli, such as pH, temperature, nutrient levels, and antibiotics, and convey this signal to the intracellular environment to facilitate changes in gene expression.^259^ TCSs are present in all domains of life and typically consist of a membrane-bound sensor histidine kinase (HK) and its cognate response regulator (RR). Upon sensing external stimuli, the sensor HK catalyzes the autophosphorylation of a conserved histidine residue in its C-terminal domain using ATP. The phosphoryl group is then transferred to a conserved aspartate residue on the receiver domain of the RR. This triggers a conformational change in the effector domain of the RR, resulting in a cellular response. The majority of the characterized RR belongs to the DNA binding superfamily and thus is assumed to function as transcription factors to induce changes in gene expression. TCSs regulate many physiological processes, such as motility, cell division, and nutrient uptake, but also antibiotic resistance and virulence.^260^

In addition to transcriptional regulation, many of the tripartite efflux systems (Table 1) are also regulated by TCSs. For example, the AdeABC efflux system of *A. baumannii* is regulated by the TCS AdeRS, encoded by the divergently encoded *adeRS* operon upstream of *adeABC*. The AdeS protein is a sensor HK that detects environmental stimuli, and the AdeR protein is a DNA-binding RR that induces changes in gene expression.^212^ AdeR activates *adeABC* expression by binding to a direct-repeat motif in the intergenic region between *adeR* and *adeA*.^261^ Importantly, mutations in *adeRS* can result in overexpression of the AdeABC pump. This has been reported in clinical MDR *A. baumannii* isolates due to various point mutations in both AdeR and AdeS.^262^ As a TCS, AdeRS also regulates genes involved in several other processes, including biofilm formation, motility, and virulence.^163^ The tripartite ABC efflux pump MacAB-TolC is regulated by the pleiotropic TCS PhoPQ, which governs virulence, adaptation to magnesium limiting environments, resistance to antimicrobial peptides, and various other processes. Nishino et al.^101^ demonstrated that in *S. enterica*, phosphorylated PhoP binds to a PhoP box upstream of the *macAB* operon thereby directly repressing *macAB* expression. However, a recent study found that PhoP-inducing signals, such as low magnesium, generally act to increase *macAB* transcription in *Salmonella*. The difference in the role of PhoPQ in *macAB* regulation may arise from differences in experimental conditions, PhoP turnover, and changes in rRNA levels in response to low magnesium. Whereas MacAB-TolC remains functional in the majority of *S.* Typhimrium lineages, the *S.* Typhimrium ST313 lineage has been reported to exhibit a pattern of evolutionary convergence toward an impaired MacAB-TolC efflux system. This possibly represents a unique adaptation to allow systemic infections of permissive hosts who are unable to clear infection.^263^ Interestingly, the MacAB-TolC homologue in *S. maltophilia*, MacABC, has been reported to be intrinsically expressed as inactivation of the TCS genes *macRS* was found to not affect *macABC* expression.^116^ Some TCS are capable of regulating more than one tripartite efflux complex. For example, EvgAS in *E. coli* has been demonstrated to regulate the expression of *acrAB*, *emrKY*, *mdtEF*, and *tolC* (Figure 3),^264^ which encode AcrAB-TolC, EmrKY-TolC, and MdtEF-TolC pumps, respectively. Likewise, the TCS BaeSR in *E. coli* and *S.* Typhimurium can detect heavy metals and in response activate the expression of both *mdtABC* and *acrD*, which encode MdtABC and the AcrD RND transporter of AcrAD-TolC efflux systems, respectively.^213,265^ In *A. baumannii*, BaeSR can detect antimicrobial compounds, such as tigecycline and tannic acid, and in response is believed to regulate the expression of AdeABC, AdeIJK, and MacAB-TolC tripartite efflux systems.^266^ Owing to their role in antibiotic resistance and virulence, TCSs could be attractive drug targets for the development of inhibitors.

### 4.5 Other Types of Regulation

Aside from the canonical pathways of efflux pump regulation, several other types of regulatory mechanisms have been posited. Another possible mechanism of efflux pump regulation is through the action of bacterial secondary messenger molecules, such as ppGpp (guanosine pentaphosphate), which is involved in the stringent response. A recent study found that the deletion of the *A1S_0579* gene, which is involved in the synthesis of ppGpp, increased antibiotic susceptibility in *A. baumannii*. To elucidate the mechanism of increased antibiotic susceptibility, expression levels of efflux pump genes were determined. Interestingly, the expression of the genes *adeB* and *adeIJK*, which encode components of tripartite efflux systems, was significantly reduced (at least 10-fold reduction).^267^ This suggests a link between secondary messenger molecules and efflux pump regulation; however, further research is required to elucidate the molecular mechanisms. A recent study found that post-translational modification is another determinant of efflux pump activity. In *C. jejuni*, the CmeABC RND pump is subjected to *N*-linked glycosylation, which was shown to enhance protein thermostability, stabilize protein complexes, and promote protein–protein interaction, thereby facilitating efflux activity.^268^

## 5 Classes of Transporter Proteins

**Figure 4:**
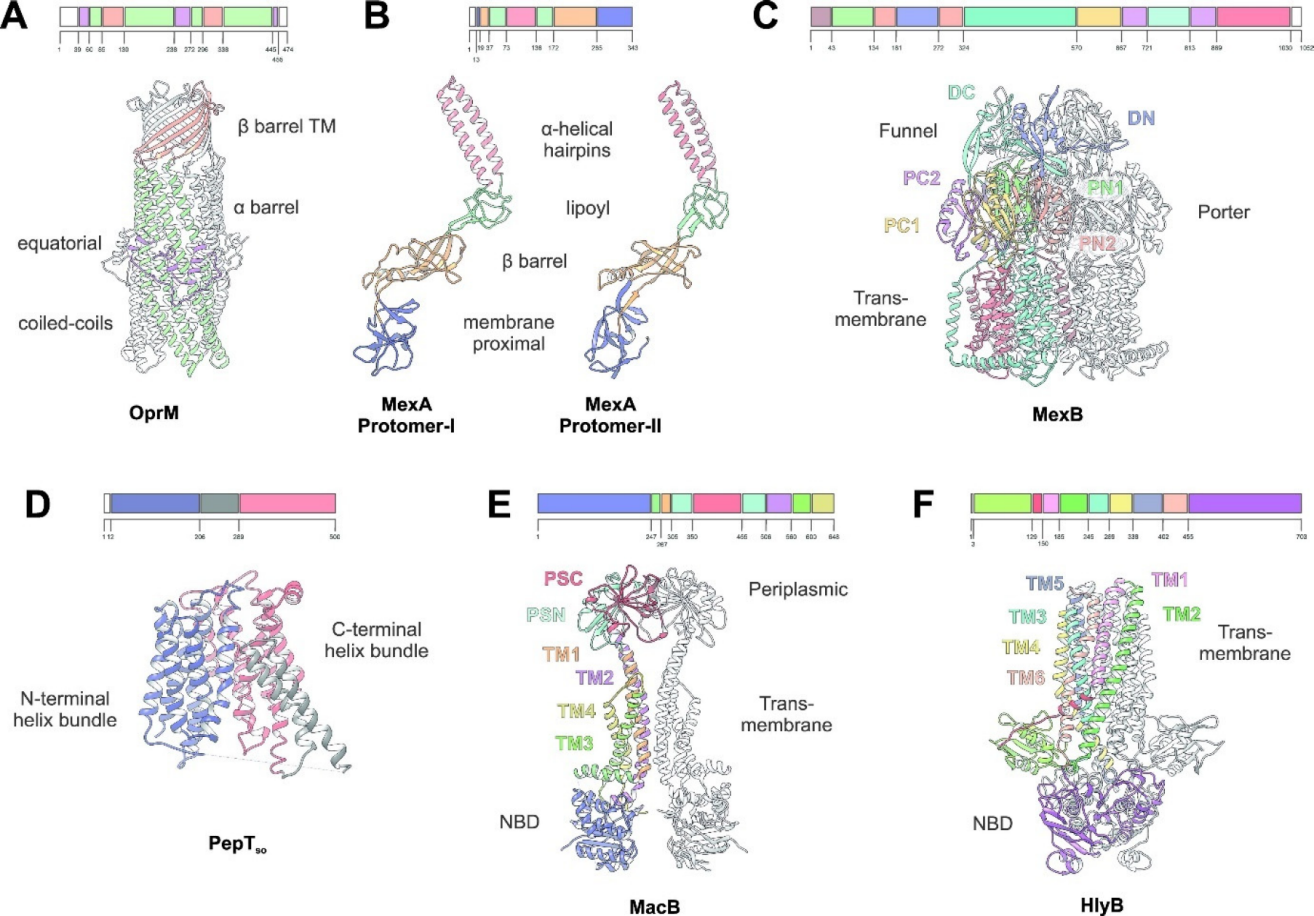
Overview of the principal components of the tripartite assemblies. The primary and tertiary structures of the main group of proteins involved formation of tripartite efflux complexes and type 1 secretion systems. The coloring of the key structural features is the same on the primary and the 3D structure. (A) Outer membrane factor (OMF) family on the example of the *Pseudomonas aeruginosa* OprM channel monomer, based on the PDB ID 1WP1.^269^ (B) Periplasmic adaptor protein (PAP), also known as the Membrane Fusion Protein (MFP) family, on the example of the *Pseudomonas aeruginosa* MexA associating with the RND transporter MexB. Two conformers are presented based on the PDB ID 6TA6;^270^ see text in section 7.2 for details. (C) A representative RND-family transporter—MexB, from *P. aeruginosa*. Trimeric assembly based on PDB ID 6T7S.^270^ PN and PC—periplasmic or porter domain N-terminal and C-terminal subdomains; DN and DC—docking/funnel domain N- and C-terminal subdomains, respectively. See section 5.1 and Figures 5 and 6 for details. (D) A representative structure of the Major Facilitator Superfamily (MFS) transporter with 14-transmembrane helices based on the POT-family member PepTSo from *Shewanella oneidensis*. PBD ID 4UVM,^271^ monomer presented. See Section Y for details. (E) A dimeric structure of the MacB transporter from *E. coli* based on PDB ID 5NIK.^272^ (F) Representative structure of the ABC transporters involved in the formation of the type 1 Secretion system (T1SS), on the example of Hemolysin alpha exporter HlyB. Dimer structure based on a homology model derived from the peptidase containing ABC transporter PCAT1 structure (PBD ID 4RY2).^273^ For details see section 5.6. NBD, nucleotide binding domain; CLD, C39 like domain.

The multidrug efflux transporters of Gram-negative bacteria are diverse and are categorized into one of six families: the Resistance-Nodulation-Division (RND) superfamily, the Major Facilitator Superfamily (MFS), the Multidrug and Toxic Compound Extrusion (MATE) family, the Small Multidrug Resistance (SMR) family, the Proteobacterial Antimicrobial Compound Efflux (PACE) transporter family, and the ATP-Binding Cassette (ABC) superfamily. The representative known structures of the transporters belonging to these families, along with their representative partner proteins, which participate in tripartite complex formation are shown in Figure 4. The individual components, structure, and assembly of each of these systems are described as comprehensively as possible below.

### 5.1 Resistance–Nodulation–Division (RND) Transporter Superfamily

Representatives of the RND superfamily can be found in all domains of life. Members are characterized by conserved transmembrane structures comprising 12, sometimes 13 or 14, transmembrane helices (TMs) arranged in two pseudosymmetric bundles. The transmembrane domains (TMDs) of these secondary active transport proteins are involved in the binding and release of H^+^ (or Na^+^). Between TM1 and TM2 and between TM7 and TM8, soluble loops of lesser conservation constitute domains involved in various biological functions.^274^ From the known structural and functional analysis of these proteins, the soluble domains often display large conformational changes as a result of ligand/substrate binding and release. The energy required for the entire transport process is transduced via the TMD upon consumption of the proton (or sodium-ion) motive force (PMF or SMF). Based on these functions and their phylogeny, RND transporters are assigned to distinct families.^275,276^

#### 5.1.1 Bacterial Drug Efflux Proteins from the HAE Family of RND Transporters

The hydrophobe/amphiphile efflux exporter (HAE) family is subdivided into three subfamilies.^275^ The HAE-1 family is from Gram-negative bacterial origin and includes the secondary active drug efflux RND transporters which are only active *in vivo* in conjunction with PAPs and outer membrane factors/channels (OMFs). The HAE-2 family members are lipid exporters of Gram-positive bacteria, and recently, structures of the monomeric mycolic acid RND transporter MmpL3 from *Mycobacterium smegmatis* were solved in apo-form and in complex with anti-TB drugs.^277^ Members of the HAE-3 family include archaeal and bacterial exporters of hopanoids.^275^ A crystal structure of the homodimeric hopanoid exporter HpnN from *Burkholderia multivorans* was elucidated.^278^

Structures of apo and substrate bound *E. coli* HAE-1 member AcrB,^279−294^*P. aeruginosa* MexB,^291,295,296^ apo and substrate bound *N. gonorrhoeae* MtrD,^297,298^*C. jejuni* CmeB,^299^*A. baumannii* AdeB,^300^ AcrB from S. Typhimurium,^301^ and TriC from *P. aeruginosa*(302) could be solved by crystallography or recently by single particle cryo-EM. HAE-1 family RND transporters are embedded in the inner membrane as homotrimers and rarely also as heterotrimers, such as MdtBC.^303^ The RND pumps form larger two-membrane spanning tripartite complexes in conjunction with a PAP and an OMF; the latter two form channel structures to create a long drug exit tunnel toward the outside of the Gram-negative cell.^270,287,304−307^

#### 5.1.2 The *E. coli* HAE-1 Member Acriflavine Resistance Protein B (AcrB)

Acriflavine resistance protein B (AcrB) from *E. coli* was identified in 1978 to be a factor in resistance against the topical antiseptic acriflavine.^308^ Later, AcrB was shown to act as an efflux pump of bile salts, and its gene is upregulated under several stress conditions.^180,309^ Early reports indicated the large substrate spectrum of this efflux pump,^309,310^ exporting a broad range of structurally diverse molecules comprising diverse classes of antibiotics, including β-lactams, macrolides, quinolones, rifamycins, aminocoumarins, tetracyclines, and oxazolidinones, as well as anticancer drugs, dyes, bile salts, detergents, and solvents^5^ (Table 1). The substrates of the pump commonly harbor hydrophobic moieties. Bianionic β-lactams are apparently poor substrates for AcrB, and the more hydrophilic aminoglycosides are not substrates for the pump. The latter compounds are preferably extruded by the homologous AcrAD-TolC efflux pump in *E. coli*.^55,311,312^

The membrane topology of the AcrB transmembrane domain was initially accurately predicted to comprise 12 TMHs and a periplasmic region composed of two large loops.^313^ Periplasmic domain swapping between AcrB and AcrD^314^ or exchange of the periplasmic domains of MexB and MexY^315^ clearly indicated that the substrate specificity of the RND pumps was determined inside the periplasmic domains. Furthermore, Tikhonova et al.^316^ showed with AcrB and MexB fusions that the specificity of interaction is encoded in the periplasmic T60-V612 region of these transporters. Soon after initial diffraction studies were published,^317^ the first crystal structure of the symmetric trimeric AcrB was solved at a resolution of 3.5 Å in 2002.^279^ The three protomers of the trimer are arranged in 3-fold symmetry perpendicular to the membrane plane, all adopting the same distinct conformation (later defined as the loose (L) or access conformation) and thus constituting a LLL trimer.

**Figure 5:**
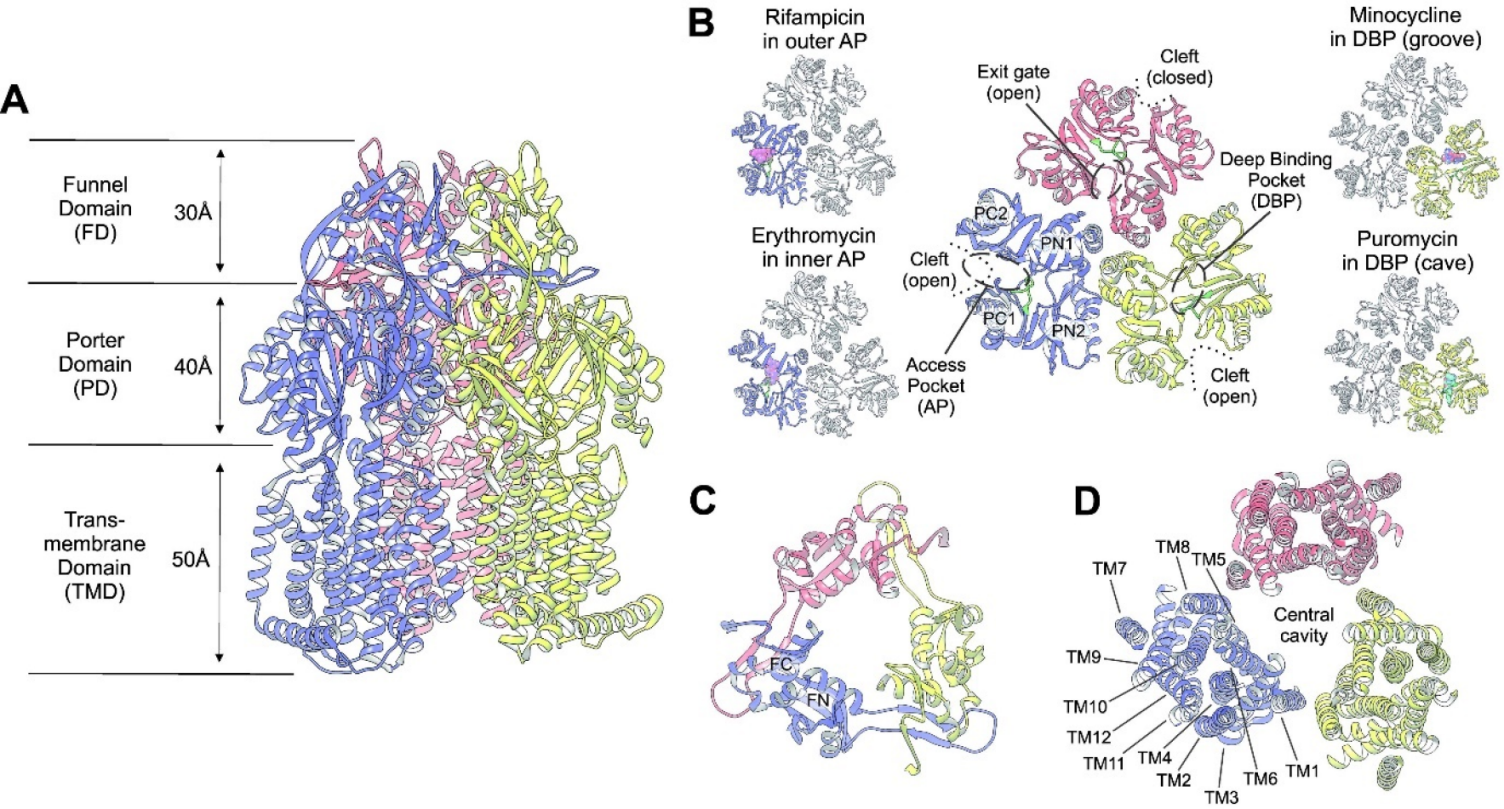
Structure of asymmetric AcrB comprising three protomers in the loose (L, blue), tight (T, yellow), and open (O, red) conformation. (A) Side view of trimeric AcrB along the membrane plane. Indicated are the transmembrane, periplasmic porter, and funnel domains (TMD, PD, and FD). (B) Top view on the porter domain. In the L protomer (blue), the PN1, PN2, PC1, and PC2 subdomains are indicated. The PC1 and PC2 subdomains constitute a cleft as part of the access pocket (AP, dashed oval). The T protomer contains also an open PC1/PC2 cleft but is less voluminous compared to the one in the L protomer. Between the PN2 and PC1 subdomains, the deep binding pocket (DBP, dashed oval) is indicated. The switch loop (green) separates the AP and the DBP. In the O protomer (red), the PC1/PC2 cleft, the AP, and the DBP are closed. From the closed DBP, a tunnel is present which exits at the funnel domain. In the asymmetric LTO AcrB porter domain representations (top view) on the left, binding of drugs to the access pocket is shown. Rifampicin (sphere representation in pink) binds in the L protomer (highlighted in blue) at the proximal side of the access pocket, and erythromycin (sphere representation in pink) binds to most distal part of the access pocket, underneath the switch loop (green).^289^ On the right, the T protomer is highlighted (in yellow), and bound minocycline (sphere representation in pink) is located in the distal groove region of the deep binding pocket,^288,290^ whereas puromycin (sphere representation in cyan) binds to the more proximal cave region of the deep binding pocket underneath the switch loop (in green).^305^ (C) Top view of the funnel domain (FD). The N-terminal FN and the C-terminal FC subdomains are indicated. The AcrB trimer is stabilized by a loop protruding from the FN subdomain connected to the FD subdomain of the neighboring protomer emerging from subdomain DN to DC of the adjacent protomer. The distal FD remains largely unaffected by the conformational cycling and remains structurally unchanged during the LTO cycle. (D) Top view (from the periplasm) of the transmembrane domain (TMD). The TMD displays 12 transmembrane helices (TMHs). The proton relay network consists of residues D407, D408, and K940 associated with R971 and T978 located in the center of the TM-domains. Adjacent protomers interact via TM1 and TM8. The large central cavity is depicted by a circle. This image was constructed with PDB 4DX5 (in complex with minocycline)^290^ for the structure images in complex with drugs. Coordinates for rifampicin (PDB 3AOB), erythromycin (PDB 3AOC), minocycline (PDB 4DX5), and puromycin (PDB 5NC5) were superposed to the 4DX5 structure.

In 2006 and 2007, three independent studies reported an asymmetric trimeric setup of AcrB^288,318,319^ (Figure 5). The use of designed ankyrin-repeat proteins (DARPins) as crystallization chaperones resulted in a higher resolution structure (2.5 Å) of the otherwise congruent asymmetric AcrB trimer.^319^ In 2012, under optimized conditions, a high-resolution structure at 1.9 Å was obtained allowing detailed insight on substrate binding.^290^ Moreover, the presence of water molecules in the TMD suggested the presence of water channels to the H^+^-titratable residues (D407, D408).^292^ The “asymmetric” crystal structures revealed three distinct conformations assigned to consecutive states of the transport cycle, generally denoted as access, binding, and extrusion states^288^ or loose (L), tight (T), and open (O) states,^318^ with the latter designation in analogy to the conformations established for the functional rotation (binding change) mechanism of the F_1_F_0_ ATP-synthase^320^ (Figure 5). The functional rotation mechanism hypothesis was supported by structure-based site-directed disulfide cross-linking studies. Experiments confirmed not only the presence of asymmetric AcrB *in vivo* but also the necessity of conformational cycling in substrate extrusion.^321,322^ Studies with a genetic fusion construct encoding a covalently linked AcrB trimer provided further evidence for interdependent conformer cycling by demonstrating that the inactivation of a single protomer abolishes the function of the entire AcrAB-TolC system.^323^

The first structures in complex with the AcrB substrates minocycline and doxorubicin were obtained by Murakami et al.^288^ at 2.8 Å resolution. The use of brominated minocycline was of crucial importance, to assign the different densities to minocycline, as the anomalous signal of bromine yields unequivocal proof of its presence in the region defined as the deep binding pocket (DBP) (Figure 5B). The use of brominated substrates was later also used to identify a novel binding site for carboxylated drugs, by the use of brominated fusidic acid.^324^

#### 5.1.3 Structural Features of AcrB

**Figure 6:**
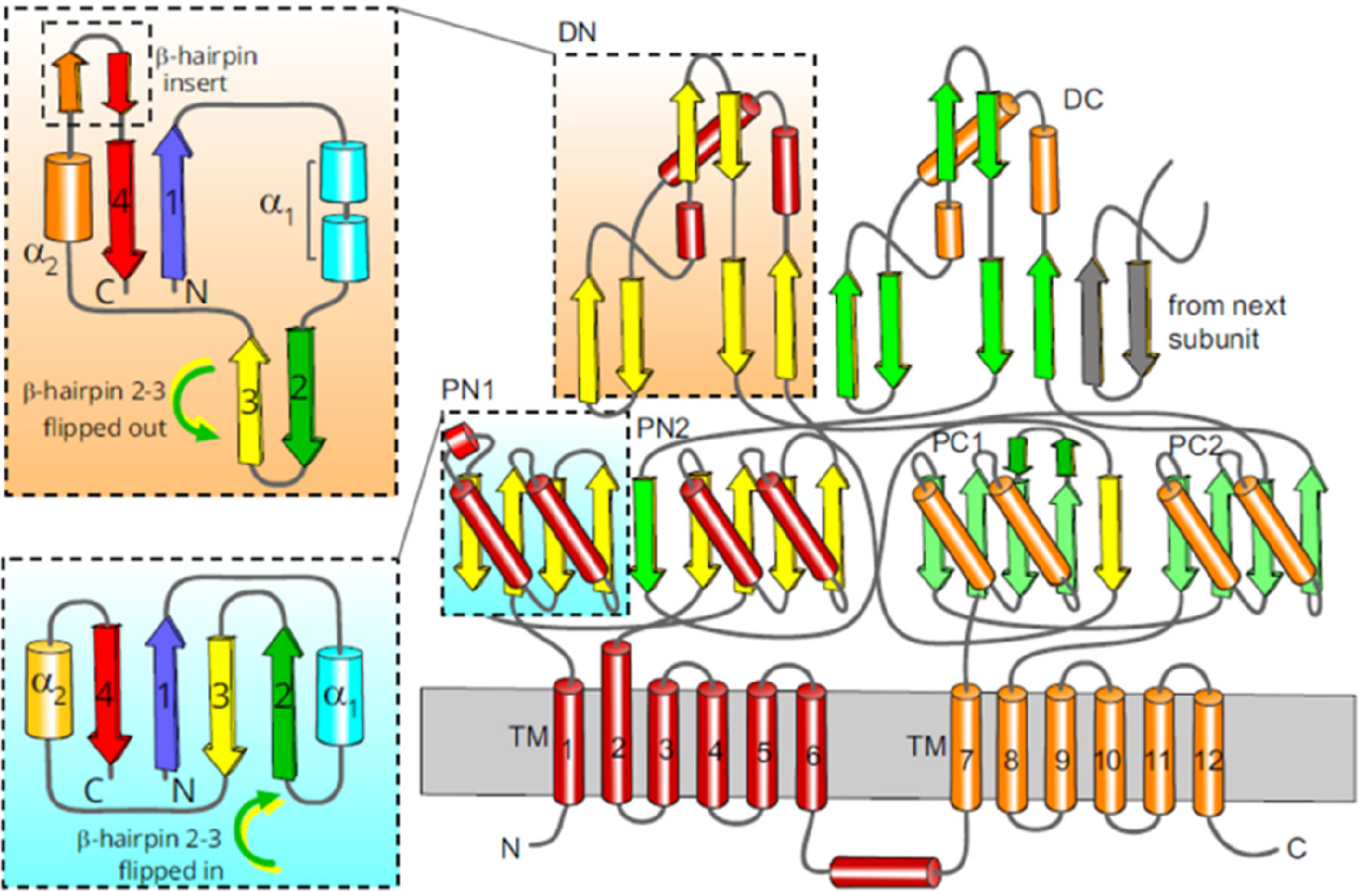
Topology of AcrB protomer. The structure of AcrB is a representation of an architecture that is shared across the HAE and HME families of RND transporters. The protomer of AcrB presents a pseudo-2-fold symmetry, associated with a gene duplication of the ancestral gene, giving two semi-identical N-terminal and C-terminal portions of the protein, each including a 6 TM-helical component with a large periplasmic loop spliced between the TM1/TM2. These loops form the porter or periplasmic domain (PD) and a docking domain. Porter domains display highly modular organization, presenting a mixed α-β sandwich of a general (β–α–β–β–α–β) configuration dubbed the PD-module (lower inset). This is repeated through the porter subdomains PN1/PN2 (belonging to the N-terminal loop) and PC1/PC2 (C-terminal loop), respectively. Subdomains PN1/PC2 and PN2/PC1 form discrete units coupled by a shared β-sheet. The docking or funnel domain comprises two subdomains, DN (or FN) and DC (or FC) respectively, which are topologically derived from the basic PD-fold of the PN/PC domains, by a flippage of the β2-β3 hairpin out of the central β-sheet (upper inset). The long β-hairpins of the DN and DC domains connect neighboring protomers by forming interprotomer β-sheets.

The three protomers within the AcrB trimer^279^ each comprise a transmembrane domain (50 Å height) characterized by two parallel pseudo-2-fold symmetric repeats of 6 TM helices (Figure 5A, D). Two central helices, TM4 from repeat 1 and TM10 from repeat 2, are surrounded by the other TMs. TM1 and TM7 are located more at the periphery of the TMD of each protomer (Figure 5D). Structures and mutational studies were conducted on charged residues within the TMD, i.e. residues D407 and D408 in TM4, K940 in TM10, and R971 in TM11,^279,325−328^ and indicated the importance of the respective charges at these positions. Both TMD repeats are connected between TM6 and TM7 by a horizontal amphipathic helix (Iα), which is located in the cytoplasm presumably near the membrane (Figure 5A). Between the protomers, there is a small contact interface located between TM1 and TM8.^329^ The TMDs of the protomers encircle a lipid filled central cavity^279,330^ (Figure 5D). The extensive periplasmic part with a maximum diameter of 100 Å is formed by the periplasmic loops protruding into the periplasm between TM1 and TM2 as well as TM7 and TM8 (Figure 5A). This part is subdivided into the membrane-proximal porter domain (40 Å length) and the distal funnel domain (30 Å) (Figure 5A, B, C). The porter domain comprises four subdomains (Figure 5B; Figure 6). The periplasmic N-terminal PN1 and PN2 subdomains are protruding from TM1 and TM2; subdomains PC1 and PC2 are the C-terminal segments of the TM7/TM8 loop. The subdomain structures display two α-helices at the external side together with four- (PN2 and PC2) or five- (PN1 and PC1) stranded antiparallel β-sheets at the interior. The cores of the PN1/PN2 and PC1/PC2 domains are organized around a similar basic architecture, which we refer to as the porter domain or PD module, presenting a mixed α-β sandwich of a general (β–α–β–β–α–β) configuration (Figure 6). This is a very versatile module, that is shared across a number of transporters both within and outside the RND family, as well as with a number of other, predominantly periplasmic proteins, as will be discussed in detail in the MacB section below.

**Figure 7:**
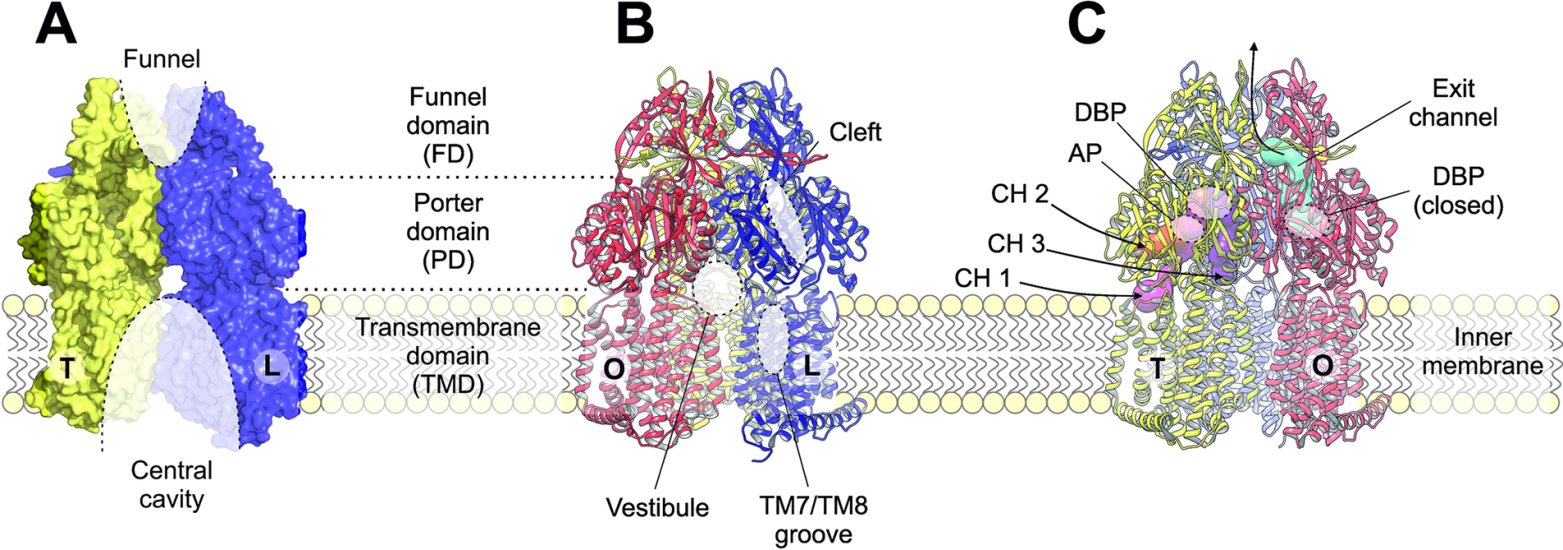
Structural features of AcrB. The asymmetric AcrB trimer comprises three protomers adopting different conformations (L, loose—blue; T, tight—yellow; O, open—red). (a) AcrB can be structurally subdivided into a funnel (FD), a porter (PD), and a transmembrane domain (TMD). The TMD consists of 12 transmembrane helices (TMs) per protomer (shown are the L (blue) and T (yellow) protomers; the O protomer is omitted for clarity). The TMD of the three protomers encloses a central lipid-filled cavity. Likewise, the FD of the AcrB trimer forms a funnel region, which is exposed to the channel structures of AcrA and TolC and facilitates the export of drugs across the OM. (b) Drug entrance sites are highlighted on the L protomer (blue). The vestibules are interprotomeric entrance channels (shown here is the vestibule between the O (red) and L protomers) toward the central cavity. The indicated TM7/8 groove in the L protomer is the entrance toward channel 1 (CH1, shown in panel c). This groove is also present in the T protomer. The cleft between the PC1 and PC2 subdomains, apparent in both the L and T protomers (indicated only for the L protomer), is the entry side for channel 2 (CH2, shown in panel c). (c) The asymmetric AcrB trimer comprises substrate entry channels in the L and T conformations. The entrance of channel 1 (CH1) starts above the TM8/TM9 groove and guides substrates from the outer leaflet of the inner membrane to the AP and further to the DBP. The cleft pathway via channel 2 (CH2) likewise leads to the AP. Compounds sequestered in the central cavity via the vestibules between the protomer interfaces can access channel 3 (CH3) for transport directly to the DBP. All entry channels are closing during the T to O transition. Concomitantly, an exit channel is created in the O protomer that connects the closed DBP to the FD.

Subdomains PN1/PC2 and PN2/PC1 form discrete units coupled by a shared β-sheet. A top view of the porter domain (Figure 5B; Figure 6) exhibits an arrangement of the PN1 subdomain located in the trimer’s inner surface and the more peripheral exposed PN2, PC1, and PC2 subdomains. A cleft between the PC1 and PC2 subdomains in the L and T conformation forms a solvent exposed protomer substrate entry site. The funnel domain (also called the docking domain) comprises two subdomains, FN (DN) of the N-terminal periplasmic loop and FC (DC) of the C-terminal periplasmic loop (Figure 5C; Figure 6). The funnel domain is a large water exposed cavity and is involved in the collection of exported drugs from the O protomer (Figure 7A). The membrane-located and proximal central cavity and the distal periplasmic funnel are separated by the central pore helices of the PN1 subdomains at the center of the trimer porter domain (Figure 6B; Figure 7A). The protomers are noncovalently connected by long interconnecting loops emerging from the FN to the FC subdomains protruding in the neighboring protomers^279^ (Figure 6A) and have been found essential for the trimeric assembly.^331,332^ The asymmetric structure of AcrB comprising the L, T, and O protomers revealed the presence of a substrate tunnel or channels (referred to as CH1 to CH4)^318,319,333,334^ (Figure 7). The concept of tunnels leading from the surface of the protein to the active/substrate binding site of proteins was known for soluble proteins; however, the peristaltic pump concept^318^ was rather different, as it suggested that drugs are not diffusing through these tunnels (Figure 7C) but guided along the path by conformational change, therefore enforcing directionality (to the outside). The initial hypothesis anticipated an ordered binding process (assumed to be valid for all drugs recognized by this pump, e.g., β-lactams, tetracyclines, fluoroquinolones, macrolides, detergents), comprising initial binding to the access pocket, followed by binding to a deep binding pocket and finally extrusion via an exit tunnel. In the L protomer, the AP is open and the DBP is closed. Substrates can access the AP from the periplasm via the open PC1/PC2 cleft (Figure 5B). Furthermore, a large groove is formed by TM7-9, where the substrate dodecylmaltoside is observed in several AcrB structures.^290,319^ However, the T protomer appears to have multiple channels leading to both the AP (reduced in volume compared to the L protomer access pocket) and the DBP (Figure 7C). Channel 1 (CH1) starts at the TM7/8 groove (also referred to as the TM8/9 groove) and leads to the AP, past the switch loop, to the DBP.^290,319,335^ The channel 2 (CH2) entrance is at the PC1/PC2 cleft and also leads to both binding pockets.^318,319,333^ Channel 3 starts from the lipid filled central cavity and leads directly to the DBP (and not to the AP).^333^ Finally, a fourth channel (CH4, not shown) was postulated starting from the TM1/2 groove also directly toward the DBP.^334^ Interestingly, despite the overall substrate polyspecificity of the AcrB efflux pump, the channels appear to have restricted access to certain drugs, although it is not yet clearly defined for every channel and channels might have overlapping specificities.^333^ CH1 is possibly involved in the transport detergents and smaller drugs, such as β-lactams. CH2 has a wide entrance and leads directly from the periplasm to the AP and is most likely involved in the transport of high molecular weight drugs such as macrolides. CH3 has been shown to be involved in planar aromatic cations, such as ethidium or rhodamine 6G.^333^ CH4 is proposed to be involved in the transport of carboxylated drugs, such as fusidic acid and β-lactams.^334^ Further studies have to be conducted to analyze the precise substrate preferences and overlap between the different channels.

#### 5.1.4 Substrate and Inhibitor Binding to Multidrug Efflux RND Transporter

Structural analysis has been conducted for several RND transporters, among them structures revealing the binding of substrates (drugs) to various areas within the porter domain and to the TMD grooves between TM1 and TM2 and between TM7 and TM8. Here, we describe the substrate and inhibitor binding to the multidrug efflux RND transporters from the hydrophobic/amphipathic exporter-1 (HAE-1) family. For an excellent review of antibiotic/inhibitor complex structures of this family and those of the related heavy metal ion exporter family (HME), the reader is referred to the work of the group of Edward Yu and his current review in the same issue of this journal.^336^ The L, T, and O states are structurally different both in the TMD and the porter domain, but the differences are especially apparent at the structural level of the porter domains (Figure 5B). Of note, crystallographic analysis of the symmetric (LLL) state^279−287,337^ suggests that this trimeric conformation might be the resting state of the transporter. Some of the structures presented in the articles referenced above also present drugs bound to the L protomer TMD/periplasm interface region and the PC1/PC2 cleft region in this symmetric state.

For the asymmetric AcrB structures,^288−294^ binding in the trimeric HAE-1 pumps occurs in the L and T states. In the compact O state, the only volume inside the periplasmic porter domain is the exit tunnel (Figure 7C). The drug binding access pocket (AP) is located between the β-sheets of the PC1 and PC2 subdomains in the L state. AcrB co-structures with high molecular weight compounds such as rifampicin, erythromycin^289^ (Figure 5B), and dimeric doxorubicin^290^ indicate different binding preferences within the AP. In accordance with the multisite-drug oscillation hypothesis,^338^ drug binding occurs at various subsites with the AP. While rifampicin and dimeric doxorubicin bind in the approximate center of the AP, the interaction surface of erythromycin is shifted deeper into the AP of the protein (Figure 5B).

During conformational change from the L to the T conformation, the DBP opens and is accessible for drug binding. The AP and DBP are separated by a glycine rich (G614, G616, G619, G621) switch loop emerging from subdomain PC1. Consisting of only 11 amino acids, the switch loop is highly flexible. Structures revealed its function as an adapter for the binding of various drugs, modulating the shape and interaction surface of both binding pockets without changing the overall AcrB structure.^289^ The switch loop is therefore directly involved in substrate binding, adopting diverse conformations. The important role of the switch loop for the drug transport mechanism was further emphasized in biochemical assays, exhibiting dramatic effects of single side chain substitutions (especially substitutions of glycine to proline) on the resistance phenotype.^289,290,339,340^ On the other hand, a complete inactive AcrB variant, where the glycine to proline substitutions apparently rigidify the loop, can be rescued by additional substitution of F615 and F617 to alanine.^340^ Moreover, binding of erythromycin to its binding site as observed in the wild-type protein, appears to be unaffected in an AcrB variant devoid of the switch loop (by deletion).^293^

**Figure 8:**
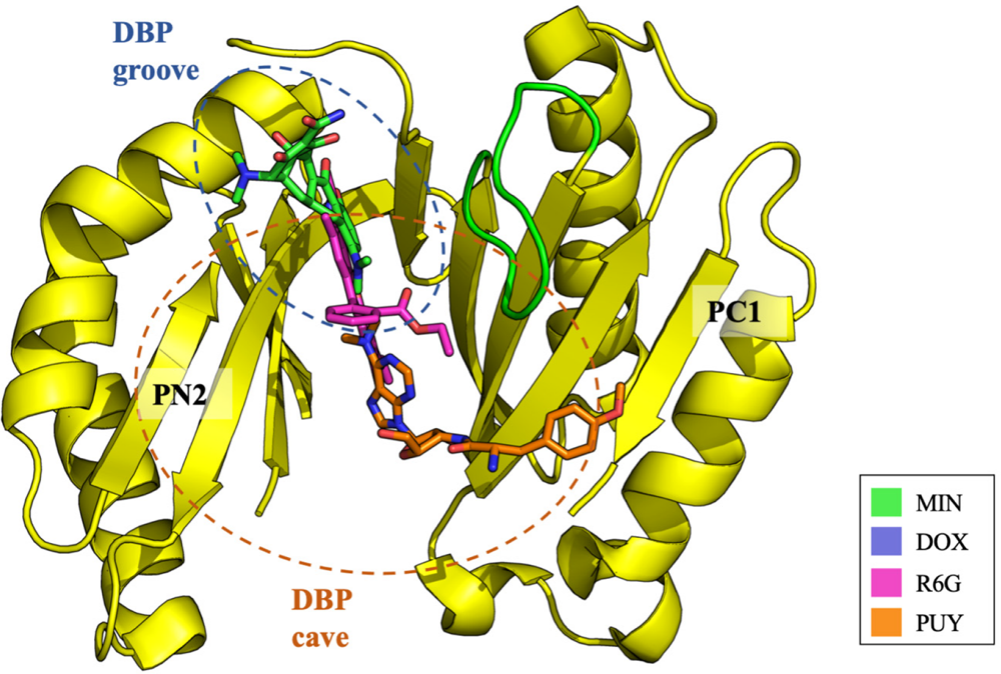
Drug binding to the DBP of the AcrB T conformer. Superimposition of the coordinates of minocycline (MIN, carbon = green; PDB: 4DX5), doxorubicin (DOX, carbon = violet; PDB: 4DX7), rhodamine 6G (R6G, carbon = pink; PDB: 5ENS), and puromycin (PUY, carbon = orange; PDB: 5NC5) in the DBP. Substrates are shown as sticks, the PN2/PC1 subdomains are represented as a cartoon, colored in yellow. The switch-loop is highlighted in green.

The opening of the DBP during the L to T conversion is mediated by an outward movement of the PN2/PC1 subdomains, while the PN1/PC2 subdomain unit remains almost rigid.^292^ Simultaneously, the AP is contracting and narrowing the available binding sites. It is anticipated in this step that substrates can slide underneath the switch loop from the AP into the DBP. Substrates are observed at diverse positions of the DBP.^290,305,341,342^Figure 8 illustrates the positions of the substrate molecules minocycline, doxorubicin, rhodamine-6G, and puromycin in the DBP of AcrB. Binding by conserved Phe residues (F136, F178, F615, F617, and F628) is especially apparent for many of the substrates in the DBP. The phenyl rings of rhodamine 6G favor an interaction via π–π stacking in the DBP cave^341,343^ near the hydrophobic trap, located in the center of the DBP (Figure 5. The planar tetracycline minocycline on the other hand is bound in the more distal narrow groove (DBP groove) in the upper part of the pocket^288,290^ (Figure 5B, Figure 8). The AP in the L protomer binds high molecular weight drugs such as macrolides or ansamycins^289^ (Figure 5B). Direct transport competition of macrolides with another high molecular mass compound, the fluorophore DiBAC_4_-(3), substantiated the drug size-preference by the AP,^344^ whereas low molecular weight drugs such as tetracyclines, which are observed to preferentially bind to the DBP, do not interfere with DiBAC_4_-(3) transport. Low molecular weight compounds rather affect the transport of the low molecular mass fluorophore Nile Red.^344,345^ While planar aromatic cations are preferably transported via CH3, all substrates have to traverse the DBP region during the T to O transition which results in transport toward the funnel domain via the exit gate (Figure 5C).

Analysis of the published AcrB/substrate co-structures indicates which and how many residues are located around the individual substrates within a radius of 3.5 Å, a distance which makes them likely to be involved in substrate binding. Table 3 shows an organization of the residues into the outer and the inner AP, the AP-DBP interface at the switch loop, and the DBP groove and cave as well as an assignment of the (potentially) interacting substrates.

**Table 3:**
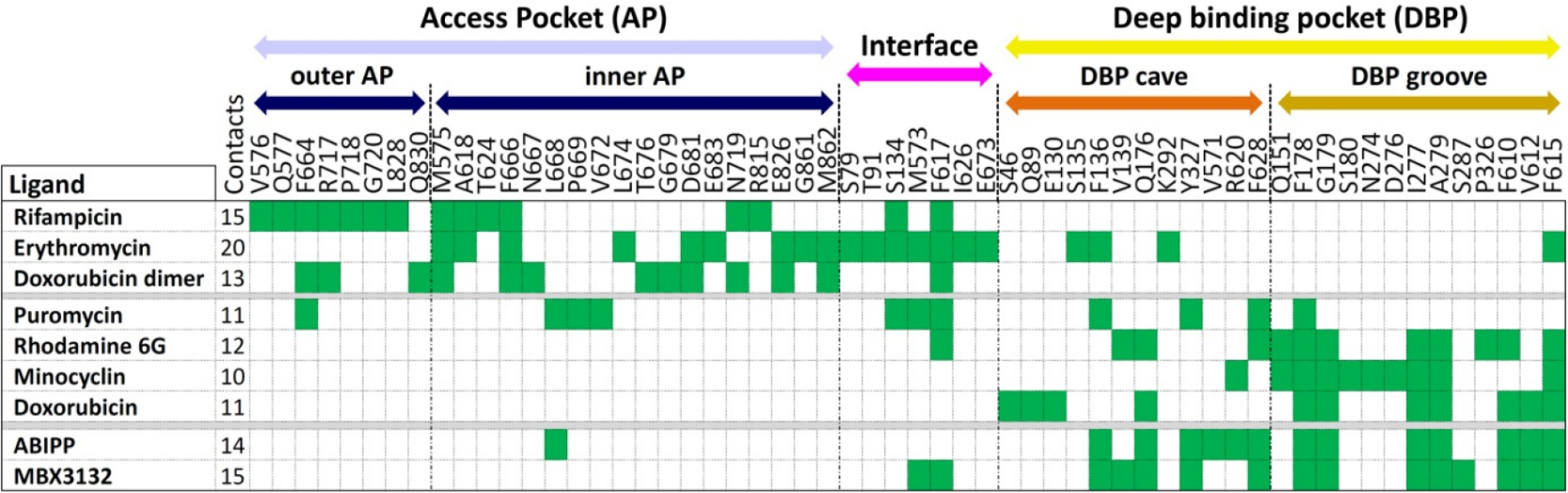
Assignment of Potentially Interacting Residues with Substrates Found in Crystal Structures^a^

a All residues with atoms found in a radius of 3.5 Å around one of the co-crystallized ligands were assigned according to their localization to the outer AP (cleft), the AP-DBP interface, and the DBP cave and groove regions. Potential interactions with a substrate are indicated in green. The numbers after the ligand/substrate give the sum of potential interacting residues per substrate. Rifampicin and the doxorubicin dimer are found exclusively interacting with residues of the AP and the interface region between the AP and DBP, while erythromycin also interacts with parts of the DBP cave and groove. Puromycin, binding underneath the switch loop, makes contact to all five indicated sections. Rhodamine 6G, minocycline, and doxorubicin bind to the DBP, primarily (but not only) to the groove region. The inhibitors, ABIPP and MBX3132 as a consequence of their orthogonal orientation, are interacting with both groove and cave regions of the DBP. Adapted with permission from Kobylka et al.^342^ Copyright 2019, Kobylka, Kuth, Müller, Geertsma & Pos under Creative Commons Attribution 4.0 International Public License: https://creativecommons.org/licenses/by/4.0/legalcode.

The assignment of substrates binding to the upper DBP groove region or the lower DBP cave region or binding to both regions of the DBP (i.e., mixed binders) is based on docking and MD simulations.^343,346^ The groove region is formed by converging β-sheets of the PN2 and PC1 subdomains in the upper part of the DBP, while the cave (limited downward by the TMD) provides a much larger cavity. In contrast to substrate binding, AcrB inhibitors of the pyridopyrimidine (ABI-PP) and the pyranopyridine class (e.g., MBX3132) bind to a much larger area of the DBP.^291,341^ Both inhibitors were shown to significantly potentiate the activity of known antimicrobials without exhibiting membrane disruption effects or antibacterial activity.^347,348^ The co-structures display extensive interactions of the inhibitors with both groove and cave regions. From this, the mechanism of action was suggested to be competition with substrates to bind to the DBP and/or conformational arrest of the T protomer.^291,341^ The aromatic moieties of the inhibitors engage in intensive π–π stacking interactions with specifically side chains F178 and F628. The pyranopyridine core structures of other MBX derivatives (MBX2319, MBX2931, and MBX3135) showed thereby essentially the same binding properties as MBX3132.^341^ Binding and stabilization of the inhibitor includes water-mediated protein–ligand interactions, and computational structural analysis indicated that the binding affinities of the inhibitors were significantly exceeding those of AcrB substrates.^341,349^ Both inhibitors ABIPP and MBX3132 are of similar size as DBP binding drugs; however, the inhibitors contact 2–3 additional side chains (Table 3) apparently stabilizing the inhibitor/protein interaction. Substrate (drug) binding appears to be less reliant on specific interactions as van der Waals interactions are preferred over hydrogen bonding. Cocrystallized rifampicin, erythromycin, and doxorubicin dimer are in contact with 13–20 amino acid side chains within the AP, and residues such as M575 and F666 are contacted by any of these drugs (Table 3). Smaller drugs such as minocycline, doxorubicin monomer, rhodamine 6G, and puromycin display less contact sites in the DBP. The additional hydrogen bonding contacts with the inhibitors might stabilize the overall geometry of inhibitor binding and intensify the van der Waals interactions in other regions of inhibitor contact.

Some progress has also been made in understanding the residues involved in binding of natural substrates to RND transporters. For example, in MtrD critical residues including F136, F176, I605, F610, F612, and F623 have been highlighted to be essential for bacterial resistance to cationic antimicrobial peptides including polymyxin B.^141^ Additionally, the W136 and R176 residues in MtrR have also been shown to be important for binding of the bile salt chenodeoxycholate.^140^

#### 5.1.5 Drug/Proton Antiport Coupling in AcrB

**Figure 9:**
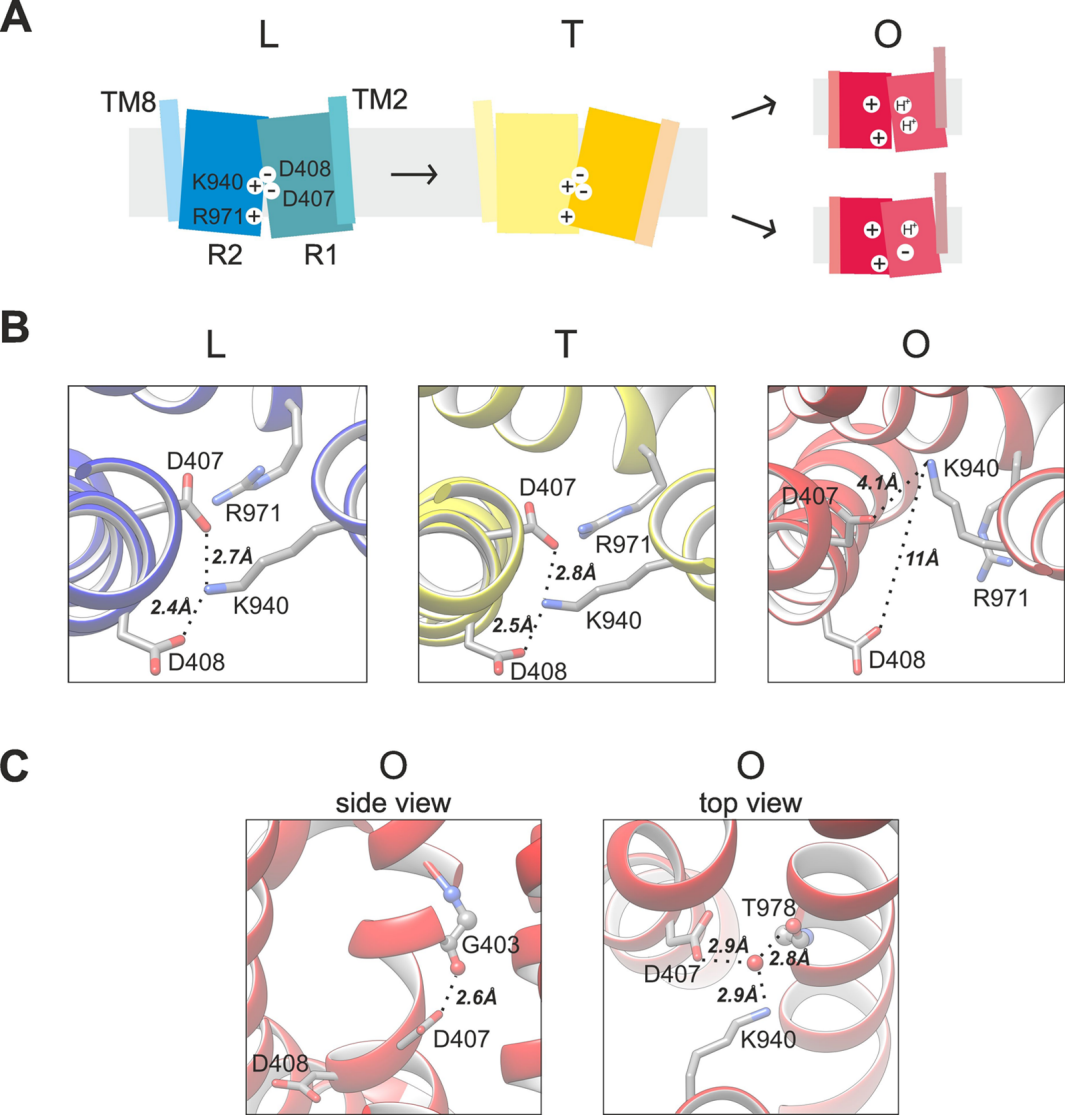
Proton binding or release induces conformational change of the transmembrane domain structural repeats. (A) Substrate/proton antiport catalyzed by AcrB can be described by two coupled mechanisms. A conformational switch from L (blue) to T (yellow) is mediated by substrate binding in the DBP of the porter domain. The rearrangement of subdomain unit PN2/PC1 leads to a downward movement of linked TM2. Consequently, the TMR1–TMR2 interface rearranges and opens a periplasmic proton entry site. Protonation of residues D407 and/or D408 results in separation of the TMR1–TMR2 interaction network. A conformational change to the disengaged state results in a switch to the O-conformation (red). TM2 moves upward and the upper part of TM8 undergoes a random-coil-to-helix transition, while binding pockets in the porter domain collapse and the exit channel becomes accessible. The proton binding site opens to the cytoplasmic side. Protons are released in the cytoplasm, down the electrochemical gradient. Charged D407 and/or D408 switch the TM domain back to the engaged state. This conformational change translates back to the porter domain. Proton entry and exit are presumably mediated through a network of water molecules. (B) Close view of the proton relay comprising D407, D408, K940, and R971. These residues undergo the most dramatic conformational change during the T to O transition. In the L (blue) and T (yellow) conformations, water molecules mediate electrostatic contact between D407/D408/K940 with R971. In the O state (red), the transmembrane domain is (almost) dehydrated, greatly affecting the p*K*_a_ values of the titratable residues. (C) Insight into the structural details of the interaction of D407 in the O conformation; the H-bonding interaction of the carboxyl group of D407 with the carbonyl group of G403 is only possible if D407 is protonated. A central water molecule connects (by hydrogen bonding) the interaction partners on TM4 (D407), TM10 (K940), and TM11 (T978).

For all characterized RND efflux pumps thus far, energy coupling is mediated via the proton-motive force (in one described case, sodium-ion motive force).^119^ In the TMDs of all RND transporters, at least one acidic residue appears to stay central to proton binding and release.^233^ Proton binding or release is coupled to changes in the electrostatics at the interface of the N- and C-terminal transmembrane repeats (TMRs) leading to conformational change and transduction of that movement toward the soluble parts of the transporters involved in substrate or ligand binding. For AcrB, this process has been studied by many laboratories in more detail compared to other RND transporters. Substitution of the central acidic residue or residues has been conducted most of the time for all studied RND proteins and was shown to be essential for activity. For AcrB, proton binding and release is facilitated by residues D407, D408 (on TM4), and K940 (TM10), the proton relay triad, located in the TMD core of each protomer at the interface of the TMRs described below (Figure 9). A further critical residue is R971 on TM11, possibly involved in the coordination of a network of water molecules connecting R971 with D407 and D408 and, in this way, affecting the p*K*_a_ values of the carboxylates.^292^ Substitution of one of these highly conserved residues abolishes substrate extrusion altogether.^279,325−328^ Two TMRs consisting of transmembrane helix 1 (TM1) and TM3-TM6 (transmembrane repeat 1, TMR1) as well as TM7 and TM9-TM12 (TMR2) are arranged in parallel pseudosymmetric fashion.^292^ During conformational cycling, the TMR1 and TMR2 repeats act as rigid bodies and remain internally largely unchanged. However, relative movements between the TMRs are a rocking motion during L to T transition and a shear motion when the TMD changes from T to O. The cytoplasmic horizontal helix Iα (Figure 6A), connecting TMR1 and TMR2, shows a rigid motion in relation to TMR2. Flanking helices TM2 and TM8 are presumably the coupling elements mediating crosstalk between the TMD and the porter domain of AcrB^292^ (Figure 9); TM2 is thereby linked to the PN2/PC1 subdomain unit, and TM8 is connected to the PN1/PC2 tandem (Figure 6B). A downward movement of TM2 relative to TMR1 occurs during the conformational change from L to T. This is most likely due to the binding of substrate to the DBP, which stabilizes the separation of the PN2/PC1 subdomain unit. This process is reversed during the T to O transition. The latter transition is catalyzed by the protonation of the TMD causing conformational change between the two TMRs. As a result, TM8 switches from a disengaged state from TMR2 in the L and T state, to an engaged state in the O conformation. A short sequence between TM8 and the PC2 subdomain, the hoisting loop,^350^ thereby undergoes a random-coil-to-helix transition, and TM8 becomes a compact elongated α-helix.^292^ The hoisting loop is an important factor in conformational coupling of TMD and the periplasmic porter domain. Mutagenesis studies revealed that it is not actively involved in energy transduction, but its high flexibility is crucial for conformational cycling.^350^ High resolution structures of the L-, T-, and O-states, both of wild-type AcrB and D407N, D408N, K940A, and R971A variants, reveal different interaction networks in this energy conversion machinery.^290,292^ In the L- and T-conformations, TMR2 K940 forms an ionic interaction with D407 and D408 in TMR1. An additional hydrogen bond between D407 and T978 in the second repeat further contributes to the stabilization of the TMR1–TMR2 complex. Residue R971 in TMR2 forms yet another interaction with TMR1 via E414 and N415. With change to the O-conformation, the proton relay residues twist and reconfigure as a result of the rigid body motion of TMR 1 and TMR2. In the disengaged state, connections of TMR1 and TMR2 are replaced by intra-TM repeat interactions.^292^ As such, the TMRs switch between an engaged and a disengaged state during cycling (Figure 9). One of the most discussed questions on the proton transport is the number of protons transported per LTO cycle. Since there are two titratable residues (D407 and D408), the anticipated number is either one or two. There is good biochemical, structural, and molecular dynamic evidence that D408 is protonated and deprotonated during the entire cycle.^292,328,351−353^ For D407, molecular dynamics studies show that it remains deprotonated (charged) in the O state,^351,352^ whereas other structural and molecular dynamics studies suggest a protonation of D407 (and D408) in the O state.^292^ In particular, the D407 residue in the wild-type AcrB structure (at 1.9 Å resolution) is in hydrogen bond distance with the carbonyl oxygen of G403 of TM4, implicated to be responsible for the stabilization of the observed kink in TM4 in the O state (Figure 9C). A hydrogen bond as observed in the structure will only be feasible if the D407 is protonated. It is very clear that further analysis, including determination of proton per cycle stoichiometry in *in vitro* reconstituted systems and the protonation state of K940 during the cycle, have to be done to obtain more insights onto this basic question.

#### 5.1.6 Structures of Other HAE-1 RND Transporters

Besides AcrB, HAE-1 RND transporter structures have been solved via X-ray crystallography or single-particle cryo-EM. For MexB from *P. aeruginosa*, crystal costructures showed dodecylmaltoside (DDM),^295^ a pyridopyrimidine inhibitor (D13-9001, also referred to as ABI-PP,^291^ lauryl maltose neopentyl glycol (LMNG), CYMAL6 neopentyl glycol, or CYMAL-7 neopentyl glycol^296^ bound to the DBP of the T protomer. For MtrD of *N. gonorrhoeae*, an X-ray structure of a symmetric LLL trimer^297^ showed a possible resting state of this pump. Recently, a cryo-EM structure of the asymmetric LTO trimer in complex with hydrolyzed, decarboxylated ampicillin or erythromycin in the DBP of the T protomer was solved.^298^ In the case of erythromycin binding, it clearly revealed that a high molecular weight, despite its size, can be present in the DBP cave region. Further structures of HAE-1 family transporters included those of AdeB of *A. baumannii*(300) and CmeB from *C. jejuni*.^299^ The AdeB trimer, with its structure solved by single particle cryo-EM, showed an OOO conformation, where every protomer lacks a distinct drug binding site. This OOO conformation was previously suggested to be sterically precluded in the case of the AcrB trimer transport cycle.^292^ For CmeB, two different trimer conformations were observed from structures obtained by X-ray crystallography. One state appeared to be again an approximate OOO state, i.e. with every protomer in the trimer in the extrusion state revealing an exit channel toward the funnel domain. In the other trimeric state, three different conformations were observed, one binding state, similar to the T state of AcrB, and one extrusion or O state, and the third protomer was in an intermediate state, unlike the L, T, and O states, which was designated a “resting” state. A similar protomer conformation was found for the HME-family Cu^+^-RND pump, CusA, in the apo-state^354^ of the crystal structure. The functional implication for this observation was tested with CmeB-containing proteoliposomes and different labeling of the protomers in order to observe single-molecule FRET signals upon conformational change of the protomers relative to each other. Interestingly, the results were not in accordance with a concerted cycling of the protomers in the trimer but rather supported the independent (i.e., not dependent on the conformation of the neighboring protomer) conformational change of each of the protomers during transport. Of note, the study was done with the isolated RND component (CmeB). Concerted cycling, however, might be highly supported by the PAPs (like in this case CmeA) and the outer membrane channel (CmeC) to generate a concerted cycling *in vivo*. Recently, single-particle cryo-EM structures of AcrB wild-type and of a clinically relevant resistance mutant G288D^355^ from *S*. Typhimuirum were solved using purified samples in styrene maleic acid (SMA) copolymer.^301^ The AcrB trimer structure revealed a similar state as observed for the *E. coli* LLL state;^279^ however, slight differences between the protomers were observed, in accordance with previously predicted flexibility within the porter domain of *E. coli* AcrB by molecular dynamics simulations.^356^ For the G288D variant, it was observed that the residues around D288 show a notable shift, which may represent the observed increase in bulk waters and large change in electrostatics.^355^ All-atom molecular dynamics simulations indicated the increase in volume in the area close to D288 compared to the wild-type (G288) and increase in the number of water molecules present in that region.

#### 5.1.7 HME Family

The members of the Gram-negative heavy metal-ion exporter (HME) family catalyze the efflux of heavy metal ions from the periplasm across the outer membrane albeit that *in vitro* studies show that these proteins are also able to transport heavy metal ions across the inner membrane. These family members can be further categorized into several subfamilies (HME1-5).^357^ The tripartite Zn^2+^-exporter ZneCAB is involved in the extrusion of specifically Zn^2+^ ions, and the structures of the RND-component ZneA were solved in different conformations of the anticipated transport cycle, with Zn^2+^ bound to a proximal or distal binding site in the periplasmic domain.^358^ Reconstitution of ZneA in liposomes and Zn^2+^ transport experiments suggests transport of Zn^2+^ from the cytoplasm or periplasm, in an electrogenic fashion. The CusCBA complex catalyzes the efflux of Cu^+^ or Ag^+^. Structures of CusA alone and in complex with the PAP CusB have been solved.^354,359^ The TMD and the periplasmic part of the RND transporter CusA contains five methionine clusters which are proposed to coordinate metal ion transport through the protein. The uptake of Cu^+^ or Ag^+^ from the periplasm involves also the nonessential metal-ion chaperone CusF, from which Cu^+^ is transported via the PAP CusB to the Cu^+^-binding site in the periplasmic part of CusA.^360^

#### 5.1.8 Other Bacterial RND Transporters

Nodulation factor exporter (NFE) family members are supposed to act as a single component of nodulation factors as NolGHI, coding for a single gene,^361^ was identified in the Gram-negative root nodule bacterium *Rhizobium meliloti*.^362^ An NFE transporter of the plant pathogen *Pseudomonas syringae*, involved in the export of the lipodepsipeptides syringopeptin and syringomycin, and YerP from the Gram-positive bacterium *Bacillus subtilis*, involved in the secretion of lipodepsipeptide surfactin, are further examples of NFE family members.^274^ Members of the SecDF family are of bacterial or archaeal origin and have been shown to assist in protein translocation over the cytoplasmic membrane mediated by the SecYEG translocon. *Thermus thermophilus* SecD and SecF display a heterodimeric 2 × 6 TM setup with a typical monomeric RND motif.^363−366^ SecDF captures the SecYEG transported precursor protein on the periplasmic side with its soluble periplasmic domain and can independently finalize the complete translocation at the expense of the PMF, even in the absence of SecA-mediated ATP-hydrolysis (see section below).

### 5.2 Major Facilitator Superfamily (MFS) Transporters

**Figure 10:**
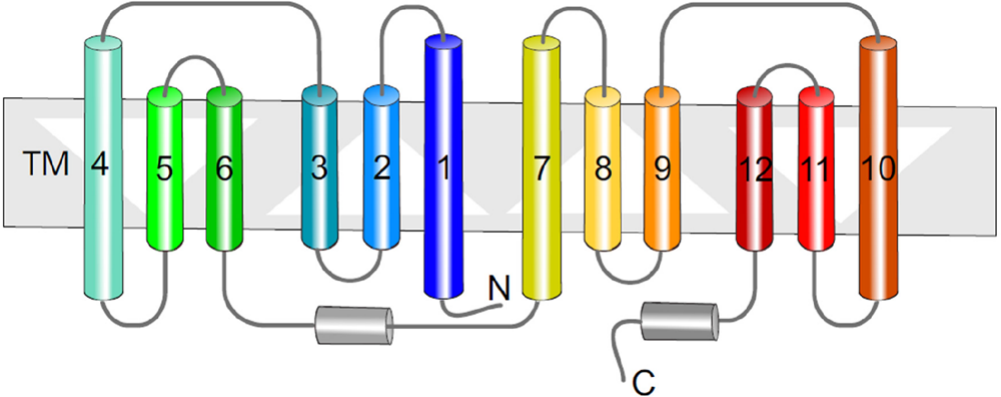
General topology of MFS transporters based on a representative of the structure of *E. coli* EmrD (PDB ID: 2GFP).^380^ The topological diagram highlights the inverted structural repeats, forming the N- and C-terminal lobes of the transporter, suggesting multiple gene duplication events.

The diverse major facilitator superfamily (MFS) is thought to be the largest group of membrane transporters found in all organisms.^367−371^ Members are uniporters, symporters, and antiporters comprising approximately 400 to 600 amino acids and 12 or 14 transmembrane helices (TMHs). Many MFS-members are involved in the uptake of nutrients, such as sugars and amino acids. This occurs in symport with H^+^ and depends on the substrate and stoichiometry, at the expense of the electrochemical gradient across the inner membrane. The general architecture of the prototypical MFS transporters (Figure 10) consists of a polypeptide chain that contains 2 lobes of 6 (or 7) transmembrane α-helices, which gives rise to a 12-TM or 14-TM “clam-like” assembly with a central symmetry between the two respective N- and C-terminal lobes,^372^ although 24 TM-spanners exist, such as NarK from *Paracoccus pantotophus*,^373^ which can be demonstrated to have evolved from the fusion of two homologous, but distinct 12-TM MFS permeases. The substrate pathway is thought to be formed between the two lobes of the permeases using an alternating access mechanism with a single substrate binding site.^374^ The high-resolution structures of the glycerol-3-phosphate:P antiporter GlpT^374^ and the lactose:H^+^ symporter LacY^375,376^ confirmed the existence of a pseudo-2-fold symmetry, which was predicted based on the sequence similarity of the two 6 TM-lobes of the prototypical MFS transporters, suggested to have arisen from a 3 TM helical element, known as MFS fold via two successive gene-duplication events,^377,378^ although this evolutionary scenario has been challenged more recently.^379^

**Figure 11:**
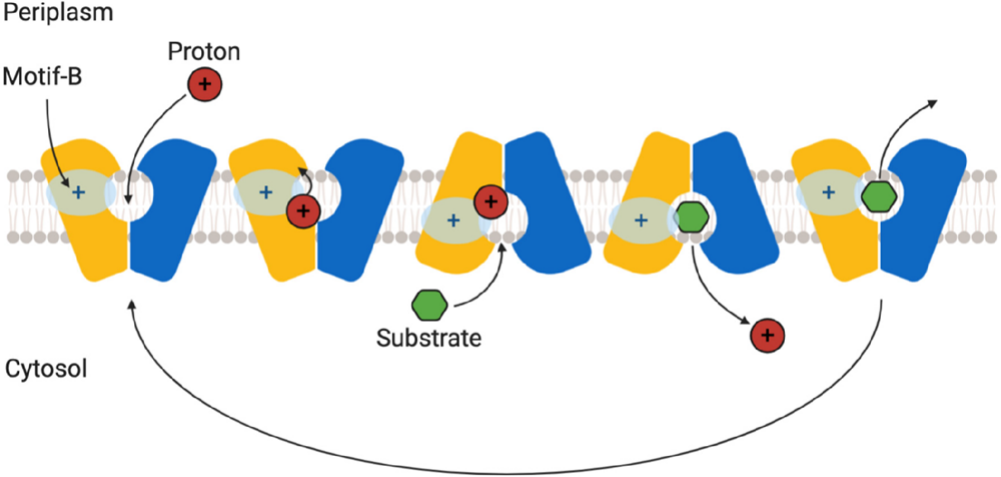
Alternating access mechanism by MFS transporters. The transport cycle of MFS proteins seems to involve ordered binding and release of proton and substrate; however, drug/proton stoichiometry varies between family members.

In MFS transporters four motifs, A–D, have been identified. Motifs A and B can be found in antiporters as well as symporters. In the multidrug efflux antiporter MdfA from *E. coli*, the sequence of motif A is ^73^GPLSDRIGRRP^83^ (mapping to the TM2–TM3 junction; numbers relate to the position in the *Ec*MdfA sequence)^381^ and has been proposed to stabilize the outward facing state,^382^ and that of motif B is ^108^FTLLRFLQG^116^ (TM4), with R112 as a pivotal residue involved in the alternative access mechanism of drug/H^+^ antiport (Figure 11). Motif-C (^154^PLLGPLVGA^162^; TM5) is unique for antiporters including MdfA^381,383^ and MdtM^384^ and because of this is called the antiporter motif.^385^ Preceding motif C is TM5, which shows a large deviation when superposing the inward and outward open structures of MdfA.^383^ Motif-D (^26^EFSTYIGNDMIQPGM^40^; TM1) is not very common in MFS transporters but conserved in MdfA orthologs. It includes the important titratable residues E26 and D34 involved in proton binding and release (see below).

The X-ray structures of the GlpT^374^ and LacY^375^ also provided a mechanistic model for the operation of the MFS transporters which has become known as the “rocker-switch” mechanism. In this model, the clamlike transporter opens up only to one side of the membrane at a time, preventing formation of a continuous aqueous conduit across it. In the case of LacY (symporter) this cycle involves binding of lactose and protons on the periplasmic side, causing closure of the “clam” to the periplasm but opening toward the cytoplasm releasing the cargo. The cycle for efflux pumps is suggested to be inverted. In terms of energy coupling, the MFS transporters catalyze uniport and symport of solutes using cation gradients (predominantly protons but occasionally Na^+^) and, in the case of exporters/efflux pumps, the antiport of solutes against protons,^369^ often in conjunction with the negative-inside membrane potential.^386^ Most MFS transporters exhibit a very distinct substrate specificity; however, MFS transporters involved in multidrug resistance, i.e. having a polyspecific substrate spectrum, have also been identified.^387^ Drug/H^+^ antiporters (DHA) from the DHA-1 (12 TM segments) and DHA-2 (14 TM segments) families are most commonly found in bacteria. MFS transporters of the DHA-1 family are arranged in 2 domains of 6 TMH bundles related in a pseudo-2-fold symmetry.^374,388^ The well-studied DHA-1 family protein *E. coli* MdfA transports a broad range of zwitterionic and uncharged drugs from the cytoplasmic side to the periplasm.^389^ Crystallographic structures indicate a central cavity at the interface of the two TM bundles containing exposed side chains involved in both drug and proton binding. In MdfA, drug binds to the central cavity, formed by TM1, TM4, TM7, and TM10. For MdfA, an inward open^381^ and an outward-open conformation was recently reported.^383^ Transport is mediated in an alternating access mechanism^381,390,391^ (Figure 11).

Two conserved acidic residues, E26 and D34, have been shown in MdfA to be involved in proton binding and release, and the negative charge on D34 is not essential for substrate binding as it could be replaced with asparagine without having an effect on *in vitro* substrate binding.^392^ MdfA is broadly flexible in recognizing electroneutral or monovalent cationic drugs and catalyzes the efflux of these drugs in exchange with a single proton, and there appears to be an indirect competition mechanism between protons and substrates. However, MdfA was shown to also transport divalent cations where the charges were separated by a long linker. It is anticipated that the export of these kinds of divalent drug molecules is in need of two consecutive transport cycles, thereby consuming 2 protons.^393^

In Gram-negative bacteria, this large superfamily comprises not only single component transporters but also tripartite efflux machineries. Tripartite multidrug pumps of the MFS family are encoded by chromosomal or plasmid genes, and usually PAP and MFS are coded on a single operon (e.g., *emrAB* in *E. coli*, *salAB* in *Rhyzobium leguminosarum*, or *emrKY* in *Shigella flexneri*), while the OMF is borrowed from another operon. However, in some cases all three pump components are encoded on one operon, such as the *emrAB-emrC* from *Chromobacterium violaceum*.^394^ As an example, a tripartite system is formed by the *E*. *coli* DHA-2 family MFS transporter *E. coli* EmrB, which interacts with the PAP EmrA, connecting it to the OMF TolC.^395^ While this is one of the first multidrug efflux systems to be identified,^396^ direct structural information about the complete assembly has not been available until very recently.^9^ The EmrAB-TolC system provides resistance to a number of antibiotics, from thiolactomycin (TLM) and cerulenin^397^ to nalidixic acid and nitroxolone and of hydrophobic proton uncouplers, including carbonyl-cyanide *m*-chlorophenylhydrazone (CCCP).^396,398^ EmrB contains 14 transmembrane helices, which sets it apart from other related MFS exporters such as MdfA and EmrD, which feature 12 TM helices. On TM1 and TM7, candidate aspartic acid candidates for H^+^ and/or drug binding are present. To our knowledge, no mutational analysis has been conducted on EmrB or EmrA, e.g. to determine residues involved in H^+^ or drug binding. Other identified tripartite MFS transporters are the VceABC system from *V. cholerae*(399) and the FarAB-MtrE system from gonococci,^79^ with the latter involved in fatty-acid resistance. The expression of the *farAB* genes is under the control of the same regulator protein (MtrR) as the genes encoding the RND-tripartite MtrCDE system, and for resistance, the FarAB system shares the OMF (MtrE) with the MtrCD system.

### 5.3 Multiple Antibiotic and Toxin Extrusion (MATE) Transporters

Based on phylogenetic analyses, bacterial MATE transporters can be divided into the NorM and DinF subfamilies of 12TM drug/Na^+^ or drug/H^+^ antiporters^400^ (for xenobiotic-transporting eukaryotic MATE transporters the reader is referred to Miyauchi et al.^401^). MATE family members transport polyaromatic and cationic compounds, such as rhodamine 6G, ethidium bromide, and tetraphenylphosphonium (TPP).^233,400^ Structures of Na^+^-dependent NorM subfamily transporters have been solved by X-ray crystallography at 3.6 Å resolution in cation- and substrate-bound states.^402,403^ X-ray structures (3.2 Å resolution) of the *Bacillus halodurans* DinF (DinF-BH) transporter of the DinF subfamily were solved in complex with rhodamine 6G bound to the central cavity via mostly hydrophobic interactions.^404^ From the same subfamily, structures of pfMATE from *Pyrococcus furiosus* in two distinct apo-form conformations, and in complexes with a derivative of norfloxacin and three thioether-macrocyclic peptides, were solved at 2.1–3.0 Å resolution.^405^ Despite MATE and MFS transporters having different topologies in their pseudosymmetrical 6 TM helix bundles, an alternating access mechanism with ordered binding and release of proton and substrate similar to that for MFS transporters has been proposed.^406^

### 5.4 Small Multidrug Resistance (SMR) Transporters

Members of the small multidrug resistance (SMR) transporter family are the smallest membrane transporters identified thus far.^407^ SMR transporters form parallel or antiparallel homo- or heterodimers comprising 4 TMs to transport drugs in dependence of the PMF.^408,409^ EmrE is the most studied SMR transporter and contains an interprotomeric hydrophobic drug binding pocket including a highly conserved Glu residue that is essential for transport. In *A. baumannii*, a homologue of EmrE, AbeS^410^ selects for the size and planarity of the conjugated aromatic moieties on drugs. Moderate changes in the property of side chains in the substrate binding site resulted in large phenotypic differences.^411^ SMR family members recognize and transport cationic lipophilic molecules such as ethidium, tetraphenylphosphonium (TPP), or acriflavine, which are extruded in an alternating access mechanism.^412^ An apo-structure of EmrE and in complex with various substrates was created by molecular dynamic (MD) simulations^413^ on the basis of a low-resolution EmrE X-ray structure in complex with TPP.^414^

### 5.5 Proteobacterial Antimicrobial Compound Efflux (PACE) Transporter Family

A transcriptomic study when *A. baumannii* was challenged with the disinfectant chlorhexidine, resulted in the discovery of AceI, which was the founding member of the proteobacterial antimicrobial compound efflux (PACE) transporter family.^415^ PACE family members are small membrane proteins comprising approximately 150 amino acids. Topology prediction revealed putative 4 TMs in a setup similar to members of the SMR family.^416^ AceI homologues were shown to confer resistance to additional biocides, including benzalkonium, dequalinium, proflavine, and acriflavine. For the AceI homologue from *Vibrio parahaemolyticus* resistance to proflavine and acriflavine was shown to be mediated by an active efflux mechanism. Recently, it has been reported that AceI of *A. baumannii* exists in a monomer–dimer equilibrium that is regulated by pH. The binding of chlorhexidine and an increase in pH was found to facilitate the functional form of the protein.^417^

### 5.6 ABC Transporter Family

The ATP-binding cassette (ABC) transporters are an extremely diverse superfamily of transporters with equally diverse functions within the cell that are widespread across both pro- and eukaryotic organisms. Classified as the TC# 3.A.1 by the transporter classification database, TCDB (http://www.tcdb.org),^418^ the ABC superfamily forms the largest functional superfamily of primary active transporters known, and in *Escherichia coli* alone 79 ABC proteins have been identified, making the ABC transporters the largest paralogous family of proteins in its genome comprising 5% of its total,^419^ forming 69 independent functional systems, with some 57 being ABC transporters.^129,420^ Several different classification systems have been proposed over the years to address this diversity within the family, see refs (421−423), taking into account both functional and topological data. Despite their considerable diversity across the family, in general organization, the ABC transporters are built around a common basic blueprint and in a first approximation can be seen as composed of a dimeric transmembrane porter section, typically in a 6 + 6 transmembrane helix configuration, and a pair of cytoplasmic nucleotide binding domains (NBDs), which provide energy coupling for the transport process by ATP-hydrolysis and could be found either as a part of a single polypeptide chain or as separate subunits.^424^ While the NBDs of the ABC transporters are relatively conserved across the superfamily and contain highly conserved nucleotide binding motifs, known as Walker A (GXXXXGS/T) and Walker B (hhhhD, or h_4_B where “h” is a hydrophobic residue),^425^ as well as the ABC signature sequence LSGGQ (aka the C-motif), that defines the family^419,422^ (see section 5.6.6 for detail and graphic), the transmembrane porter domains are rather diverse^426^ (see section 5.7.7 for details). Extensive bioinformatic analysis^427^ has suggested that the porter domains of ATP-binding cassette transport systems are polyphyletic and have evolved three times independently, forming three families which the authors designated ABC1, ABC2, and ABC3, upon which the monophyletic ATPases have been superimposed for energy-coupling purposes. It has been suggested that ABC1 porters have originated from intragenic triplication of a primordial two-transmembrane segment (TMS)-encoding genetic element, yielding six TMS proteins, while the ABC2 porters arose by intragenic duplication of a dissimilar primordial three-TMS-encoding genetic element, yielding a distinctive protein family, which is nonhomologous to the ABC1 proteins.^427^ ABC3 clade of porters on the other hand is suggested to have arisen via duplication of a primordial four-TMS-encoding genetic element, yielding either 8- or 10-TMS proteins.

Recent reclassification of the ABC transporters has established some order in this overwhelming diversity of ever-expanding structural and topological data, resulting in 7 main family “types”,^423,428^ and to avoid confusion the discourse below follows this latest classification. Of these newly defined seven classes, three types are importers, which are of limited interest to the topic of interest here, while the others include the following families: “type IV”, possessing 6 + 6 TM-helices membrane topology, includes both exporters (e.g., Sav1866 and MsbA floppase) and importers (e.g., YbtPQ; Rv1819c); “type V”, in prokaryotes a 6 + 6 TM topology of exporters/floppases including the Wzm-WztN O-antigen transport system;^429^ and the “extractor”, ABC transporters that remove compounds from the outer leaflet of the membrane, that includes members with a 6 + 6 TM membrane topology such as LptB_2_FG,^430^ which possess specialized β-jellyroll domains forming the type VI group, as well as the 4 + 4 TM members of the MacB/FtsX family constituting the newly defined type VII group.^423^ The known structures of the latter group include MacB itself,^24,272,431^ as well as the heterodimeric LolCDE lipoprotein sorting system (LolC, PDB ID 5NAA;^432^ LolE, PDB ID 5UDF(432)) and the FtsX family members (PDB ID 4N8N; 6HEE, 6HFX).^433,434^

In addition, the maintenance of phospholipid asymmetry (Mla) MlaFEDB complex,^435,436^ the structure of which has recently become available (PDB ID 7CGN; 7CH0; 7CGE; 6XBD),^437−439^ revealed unexpected structural organization of the MlaE transmembrane domain, which might merit its isolation into a new, separate topological group.

As the ABC transporters are subject to a number of reviews (see refs (423 and 440)), including in the current special issue of *Chemical Reviews* itself,^441^ to avoid redundancy, below we will focus on aspects of the organization of the ABC transporters that are specific to their function within the context of the tripartite assemblies. Indeed, despite the diversity of folds and architectures described above, only two groups of prokaryotic ABC transporters participate in tripartite assemblies—namely the transporters associated with protein secretion and type 1 secretion systems (belonging to the type IV [exporter] group) and the MacB/FtsX-like transporters (belonging to the newly defined type VII),^428,442^ with only the latter of them conclusively associated with efflux-function, despite some recent circumstantial evidence.^443^

#### 5.6.1 Type IV Family of ABC Transporters Involved in Tripartite Assemblies

As mentioned above the ABC transporters present a great structural variety, which corresponds to an equally vast diversity of transported cargoes, which vary from xenobiotics and dyes, to lipid and glyco-lipid molecules; lipoproteins and outer-membrane components to S-layer proteins; as well as an array of virulence factors, including large adhesins, toxins, and smaller antibacterial peptides.^422,428^ The latter two functions, that allow secretion of proteinaceous cargoes bypassing the Sec-pathway, are served by two related families of type IV ABC transporters, the first of which, often called the NisT/McjD family after its representative members,^444,445^ is solely dedicated to cargo transport and exclusively deals with smaller cargoes without changing them during transport, while the second family covers bifunctional transporters, which provide maturation as well as transport function, proteolytically processing their substrates, which are hence known as the peptidase-containing ATP-binding transporters (PCAT) or ABC transporter maturation secretion (AMS) family.^446,447^ These AMS/PCAT family transporters, apart from small antimicrobial peptides, are capable of transporting very large cargoes such as toxins and adhesins and participate in formation of type 1 secretion systems (T1SSs) in Gram-negative bacteria.^448,449^ A detailed description of these tripartite systems alongside a graphic overview is provided in section 10. In the following paragraphs, we will discuss both these transporter families in turn, focusing on the transporters from Gram-negative organisms, which contribute to tripartite assemblies. For comprehensive reviews of the ABC transporters associated with the antimicrobial transport in Gram-positive (Firmicutes) bacteria please consult refs (444 and 445)

#### 5.6.2 NisT/McjD Family of Bacteriocin Transporters

In their interspecies warfare, bacteria have developed a number of adaptive strategies, including the production of narrow-spectrum antimicrobial peptides, which they deploy against their competitors, which are generally termed *“bacteriocins”* (a term that also includes the heavily modified lantibiotics, which do not always perform bactericidal function), and which in Gram-positive organisms exert their action by disrupting the membrane potential.^450,451^ When applicable to Gram-negative bacteria these antimicrobial peptides are designated *“microcins”*.^447,452^ A large class of these are the so-called ribosomally synthesized and post-translationally modified peptides (RiPPs),^453,454^ and they are primarily secreted by dedicated ABC transporter systems, bypassing the Sec-pathway. Microcins are gene-encoded antibacterial peptides, with molecular weight below 10 kDa,^455^ and could be both modified and nonmodified, with the latter group encompassing a great variety of lasso peptides, siderophore peptides, nucleotide peptides, and linear azole(in)e-containing peptides.^452^ While the peptide antibiotics produced by Gram-positive organisms mainly act as ionophores, the microcins, which are secreted by the Gram-negative bacteria attack a diverse range of cellular targets including DNA gyrase (microcin B17),^456^ protein synthesis (microcin C7),^457^ and dissipation of the membrane potential (colicin V).^450^

#### 5.6.3 Structural Features of McjD and the Standard Cycle of the Type IV ABC Family

The best studied member of the family is the enterobacterial McjD transporter,^458,459^ which is involved in the transport of the eight-residue macrolactam-containing lasso-peptide microcin J25 (aka MccJ25).^460^ MccJ25 has a narrow spectrum and is principally active against *Salmonella* and *E. coli* and provides the cells expressing it self-immunity.^461^ McjD is related to the prototypical NisT ABC transporter from the Gram-positive *Lactococcus lactis* which is involved in the transport of the commercially important lantibiotic nisin,^444^ with both of them giving the name to the wider family. In *E. coli*, MccJ25 is secreted by a dedicated ABC transporter McjD; albeit, additional ABC transporters of the same group may be involved, including YojI.^63,462^ Both McjD and YojI require the action of the OMF TolC for the export of MccJ25, although it is not clear whether any of them participate in *bona fide* tripartite systems or there is a soluble intermediate of transport.^63,462^

**Figure 12:**
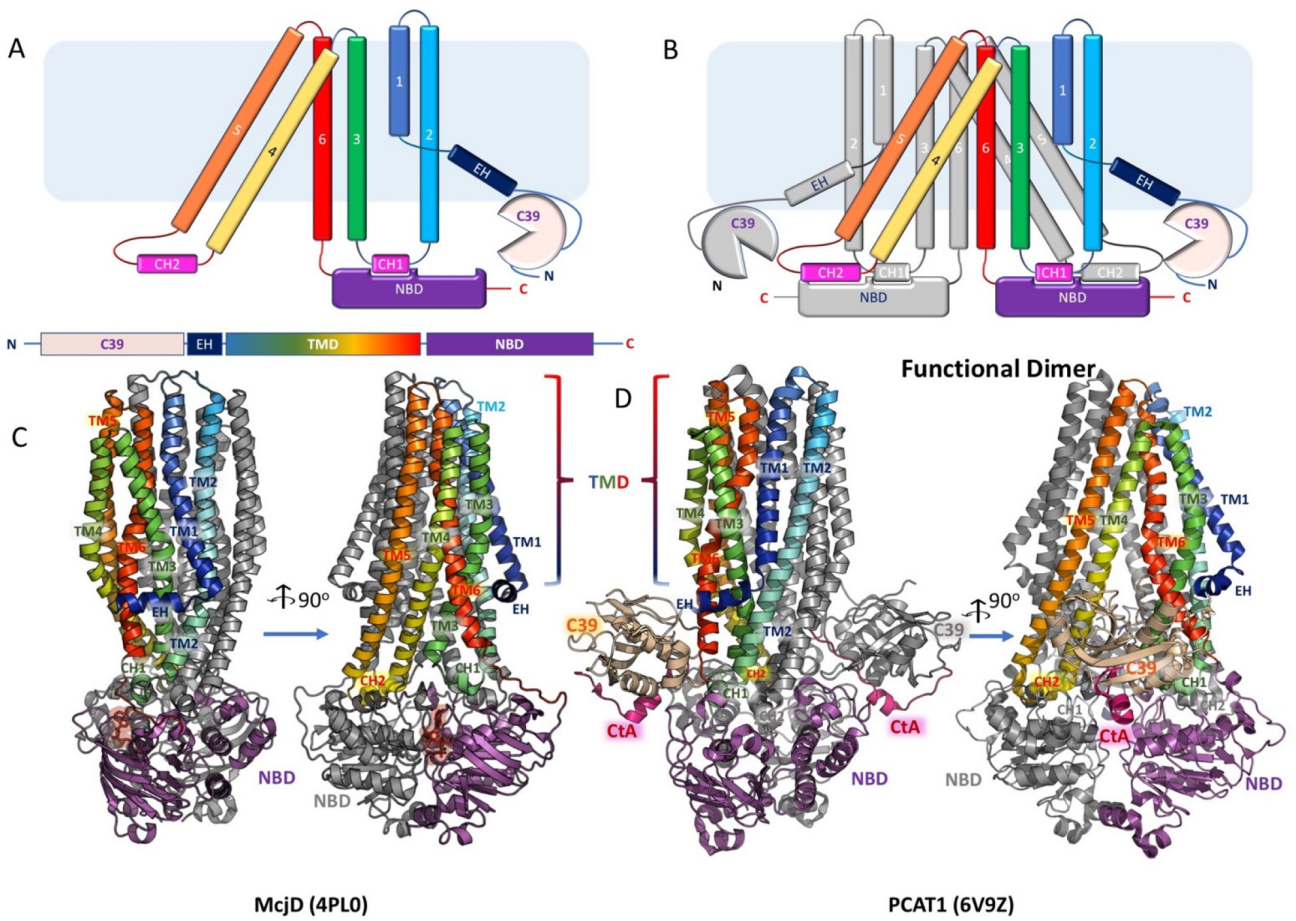
General view of the type IV ABC transporter topology and features of the typical type IV ABC transporter structure. Schematic representation of the topology of the isolated monomer of ABC transporters belonging to the type IV group (panel A) and the dimeric formation (panel B), highlighting the cross-protomer engagement of the nucleotide binding domains (NBDs) by the coupling helices 1 and 2 (CH1 and CH2, respectively). The optional N-terminal C39 or C39-like domain (CLD), which is present in a subset of the family, is shown as a “packman”. The TMD is presented as a rainbow from N- to C-terminal, and the EH represents the elbow helix. The second protomer of HlyB is presented in gray. Approximate membrane boundaries are represented by the blue rectangle. (C) View of a microcin-transport associated McjD based on the PDB ID 4PL0 (two side views 90° apart) highlighting the principal structural elements. Coloring as in panels A and B. (D) PCAT1 in complex with its transported peptide CtA, based on the PDB ID 6 V9Z. The C39-domain is colored wheat.

The structure of the McjD (PDB ID 4PL0)^459^ is representative of the type IV family of ABC transporters, sharing close topological and structural similarity to other canonical type IV transporters, such as Sav1866,^463^ MsbA,^464^ and Pgp/ABCB1^465^ (Figure 12). The structure of the biological unit is homodimeric (overall dimension of ∼125 × 55 × 50 Å), with each protomer composed of an N-terminal TMD composed of 6 TM-helices and a C-terminal NBD. The TM-helices are connected by extracellular and intracellular loops, with the intracellular loops between TM2 and TM3 and between TM4 and TM forming the “coupling helices” CH1 and CH2, respectively. Coupling helices interact with the NBDs transducing the conformational changes induced by ATP-binding and hydrolysis to conformational changes of the TMDs. In a configuration that is typical for the type IV ABC transporters,^463,465^ the CH1 contacts predominantly the NBD of its cognate protomer, while the CH2 interacts exclusively with the NBD from the opposing protomer, in a swapped-protomer manner^459^ (see topological schematic Figure 12).

The membrane protein structural biology revolution has dramatically increased our understanding of the mechanistic operation of ABC transporters, largely displacing the earlier models derived from kinetic and biochemical data, e.g. the “alternating catalytic sites”^466^ and the connected “two-cylinder engine” model.^467^ The “alternating access” allosteric model, first postulated for major facilitator transport proteins,^390^ has become a paradigm for ABC transporter action being used for rationalization of the available crystal structures of Sav1866, with the notable distinction being the suggestion that ATP-binding and hydrolysis rather than substrate acquisition are the main drivers of the conformational state transition.^463^ As its name suggests, the functional cycle of the transporter is based on the alternating inward- and outward-facing TMD conformations of the protomers. This rather straightforward model has undertaken a number of iterations and increased in complexity over the years as more diverse ABC transporters came to light. Some notable variations include the “Switch Model”, which proposes that hydrolysis in each protomer is sequential and that as the sites are freed of nucleotide, the protomers lose contact.^468−470^ In this model, the ATP binding and ATP-hydrolysis, respectively, induce formation and dissociation of an NBD dimer, as seen in a number of crystal structures, e.g., Sav1866^463,471^ and MsbA;^464,472^ furthermore, a derived model of the P-glycoprotein (Pgp, aka ABCB1) binding has been based on this approach.^465^

A second major group of models, known as “Constant Contact”,^473^ has evolved from the alternating catalytic site ideas,^466,474^ which were derived mostly from observations of the cooperativity between the two nucleotide binding sites in the P-glycoprotein. In that model, there is an alternating ATP-hydrolysis in each NBD, with one site opening at the point of ATP-hydrolysis, while the second site remains closed with ATP bound and occluded, with the process repeating itself in alternating cycles without the NBDs losing contact fully. This model has gained support from a number of structures of heterodimeric transporters, e.g., the TM287/TM288 from *T. maritima*,^475,476^ BmrCD,^477^ and TmrAB.^478,479^ This may not be restricted to heterodimeric transporters with degenerate sites; however, e.g., experiments by Siarheyeva et al.^480^ identified an asymmetric and catalytically active state of the Pgp/ABCB1 that did not show reduced binding of drug substrate, suggesting that ATP-binding does not drive transport, contradicting the “switch model”. It thus seems plausible that the mechanistic cycles of different ABC transporters may align closer to either one or the other model, or indeed by a tuned-up, blended version of each, giving rise to a considerable variety of allosteric modulation, and these possibilities are further explored in dedicated reviews (see refs (423, 473, and 481)

The crystal structure of McjD reveals the transporter in an outward “occluded TMD” conformation, presenting a substrate-binding cavity, which is sealed both from the periplasm and the cytoplasmic ends. This cavity, at ∼5900 Å^3^ is sufficiently large to accommodate the full microcin allowing for a single-step translocation, with several residues being specifically important for cargo recognition, notably F86, N134, N302,^459^ and TM3/TM6 lining the walls of the cavity. In comparison, the structure of the MsbA in an occluded, ligand-bound state^464^ presents a conformation of its TM3 and TM6, which bend toward the center of the TMD eliminating the corresponding cavity.

As the McjD has been co-crystallized with the ATP analogue AMP-PNP and MgCl_2_, both of its NBDs were found in a dimerized, ATP-bound state, with the P-loop and ABC-signature motifs involved in binding of the nucleotides, and correspondingly the TMD is also closed up.^459^ The TMD–dimer interface is formed by TM2 and TM5/TM6 from one subunit, the binding of which to the equivalent TM5/TM6 and TM2 from the opposite subunit buries a surface area of ∼7,100 Å^2^ per protomer. The structure of McjD suggested a model of its conformational cycling,^459^ where the inward facing open (IF_o_) apo-conformation allows binding of the transported substrate to the transporter, which facilitates the transition into an inward-closed conformation (IF_c_) which becomes ATP-binding capable. The binding of the ATP to the NBDs at this point was proposed to bring the transporter into a hypothetical substrate-engaged occluded conformation (Occ), and it is supposed to transit into a nucleotide bound outward-facing open (OF) conformation. Upon release of the substrate, the transporter was suggested to adopt an the outward occluded (Occ) conformation yet again, however this time in a substrate free state. The structure solved (PDB ID 4PL0)^459^ was associated with this post-transport occluded stated, and subsequent ATP-hydrolysis and release of the bound ADP and Pi eventually resets the transporter to the initial (IF_o_) configuration completing the cycle. Later the structure of the eukaryotic P-gp (ABCB1)^465^ has been used to further refine this cycle.

Further research, adding pulsed electron–electron double resonance (PELDOR) and single molecule Förster-resonance energy transfer (sm-FRET) measurements to probe the conformational changes and the dynamics of McjD along its transport cycle,^461,482^ has led to the suggestion that irrespective of the presence or absence of nucleotides, in the absence of MccJ25, McjD remains in an occluded conformation and that it requires both MccJ25 and ATP to open, presenting a mechanism for the substrate transport and selectivity that was dubbed the “occluded-mechanism with transient opening”. Such a mechanism would make it distinct from the other type IV ABC transporters discussed above, which are capable of adopting an outward open conformation in the absence of substrate.

#### 5.6.4 ABC Transporters with Double Functions—The Peptidase-Containing ATP-Binding Cassette Transporters (PCAT) Family

These peptidase-containing ATP-binding cassette transporters (or PCATs for short) are also sometimes designated in the ABC transporter maturation and secretion (AMS) family and also belong to the wider family of type IV exporters, which possess a 6 + 6 TM-helix topology^423,444^ and are present in both Gram-positive and Gram-negative bacteria. The transporters involved in protein secretion and maturation are unique among ABC transporters, due to the presence of extra domains required for substrate recruitment and processing. These domains typically have a proteolytic function and are a result of a gene fusion with peptidases belonging to the cysteine protease superfamily, classified as family C39, bacteriocin-processing peptidase, with their protease activity being dependent upon the catalytic cysteine, histidine, or aspartate residues that are conserved among papain-like cysteine proteases^483^ and are hence designated either as PEP or C39 domains. Crystal structures of C39 domains, e.g., ComA,^484^ have confirmed their association with the Cys-protease family. The substrates of the PCAT transporters contain an N-terminal “leader sequence” that typically ends in a double Gly-motif (variations G-G, G-A, or G-S), and mutational analysis, based primarily on the quorum-sensor pathway-associated transporter ComA, identified a consensus leader-peptide recognition sequence of Leu(-12)-(X)_3_-Glu(-8)-Leu(-7) located upstream from that double Gly-motif.^484^

In Gram-positive bacteria, PCATs function as both maturation proteases and exporters for peptides involved in quorum-sensing^485^ or antimicrobial peptides, including lantibiotic and nonlantibiotic bacteriocins, e.g., colicin V and similar class II bacteriocins, and various classes of RiPPs, which are extensively post-translationally modified over their C-terminal core peptide.^453,486−488^ The biosynthetic clusters of bacteriocins typically contain a dedicated PCAT exporter that directs the processing and secretion of their respective cargoes prior to membrane translocation at the conserved double Gly-leader sequence described above,^446,489^ removing a roughly 2.5 kDa N-terminal fragment, which is necessary for cargo secretion and activation of their antibacterial function.^490^

In Gram-negative organisms, PCATs, by pairing with respective PAPs and OMFs, form type 1 secretion systems (T1SSs) capable of exporting a range of virulence factors including ligases, scavenging molecules, and proteases,^157,491,492^ as well as gigantic adhesins with a mass over 1 MDa,^6,493^ e.g. in the export of hemolysin A the PCAT transporter HlyB pairs with HlyCD.^214,494^

#### 5.6.5 Unique Features of the AMS/PCAT Transporters—N-Terminal C39 Protease and CLD Domains

Many of the T1SS transporters, as well as the PCAT transporters involved in microcin/bacteriocin secretion possess unique N-terminal C39-protease domains,^3,447^ e.g., the *E. coli* MchF (UniProtKB: Q9EXN5), involved in the secretion of microcin H47 ref (495); RaxB from*Xanthomonas oryzae* (UniProtKB: Q5GWX4).^496^ These C39 domains are particularly associated with Gram-negative T1SSs involved in the secretion of so-called repeats-in-toxin (RTX) cargoes,^449^ which include adhesins,^497^ as well as hemolytic and cytolytic pore-forming toxins,^498,499^ which feature a noncleavable C-terminal secretion signal.^500^ As their name suggests, the RTX toxins are defined by the presence of repeated glycine- and aspartate-rich nonapeptide consensus sequences known as the “GG-repeats” (GGxGxDxUx; where x is any residue and U is a bulky hydrophobic residue).^501^ Depending on the transported cargo, the GG-repeat number can vary from less than 10 to over 40.^449^ Concomitant with the secretion of the T1SS substrate into the extracellular medium, the GG-repeats bind Ca^2+^ ions with high affinity, triggering cargo-domain folding.^500,501^

**Figure 13:**
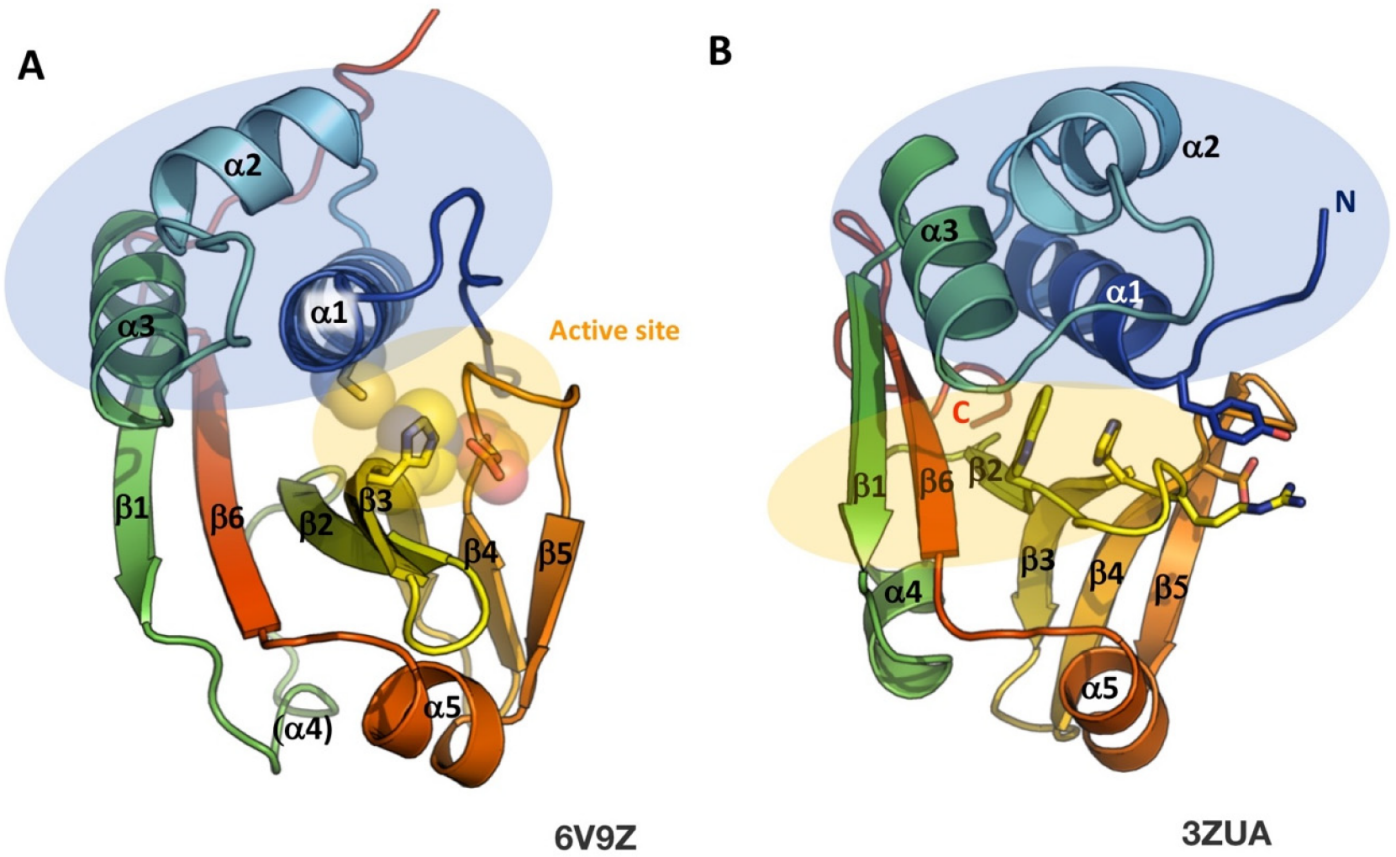
C39 and CLD domains in AMS/PCAT transporters. (A) Structure of the catalytically active C39 domain from PCAT1 (based on PDB ID 6V9Z).^505^ The main elements of the secondary structure, as well as the key catalytic residues are highlighted. (B) The structure of the C39-like domain (CLD) of the HlyB transporter in identical orientation based on PBD ID 3ZUA.^506^ While the general architecture is preserved, the active site cysteine residue is substituted by a tyrosine.

The C39-domain (Interprot ID IPR005074) belongs to the MEROPS^502^ peptidase cysteine-proteinase family CA,^483,503^ which includes proteins with a papain-like fold. This homology-based prediction was confirmed, when the C32 and H105-residues of the N-terminal C39-domain of CvaB transporter were shown to be critical for the calcium-dependent cysteine protease activity and the secretion of its native cargo colicin V.^504^ The crystal structure of the C39 domain belonging to the bacteriocin-associated ATPase ComA from *Streptococcus* provided a structural template to the family (PDB ID 3K8U).^484^ The structure revealed a domain consisting of two subdomains with the active site sandwiched between them. The smaller subdomain consists of a bundle of three consecutive N-terminal α-helices, with the catalytic C17 located at the end of the first of them, and is positioned on top of a larger subdomain, that presents a twisted antiparallel β-sheet core (β1−β6) supplemented by two small α-helices (α4 and α5), and is contributing the active site residues H96 and D112, consistent with a classical catalytic triad for CA cysteine proteinases.^484,503^ A fourth residue preceding the catalytic cysteine, in this case Q11, forms a part of an oxyanion hole that stabilizes the acyl-intermediate formed during catalysis (see Figure 13).^484^

The recent structure of the protease domain of the PCAT transporter LahT from a lanthipeptide biosynthetic operon in the Gram-positive *Lachnospiraceae bacterium* C6A11 in complex with a leader peptide (PDB ID 6MPZ)^507^ provides a model for the substrate recognition, where the helical region of the substrate peptide is oriented in a small groove located between the C39 domain and the NBD of the transporter, providing an effective means to lock these domains together. In addition, the leader peptide may serve as a backstop that prevents the cleaved cargo from sliding back into the cytoplasm and thus provides the directionality of transport upon binding of ATP to the NBDs.

**Figure 14:**
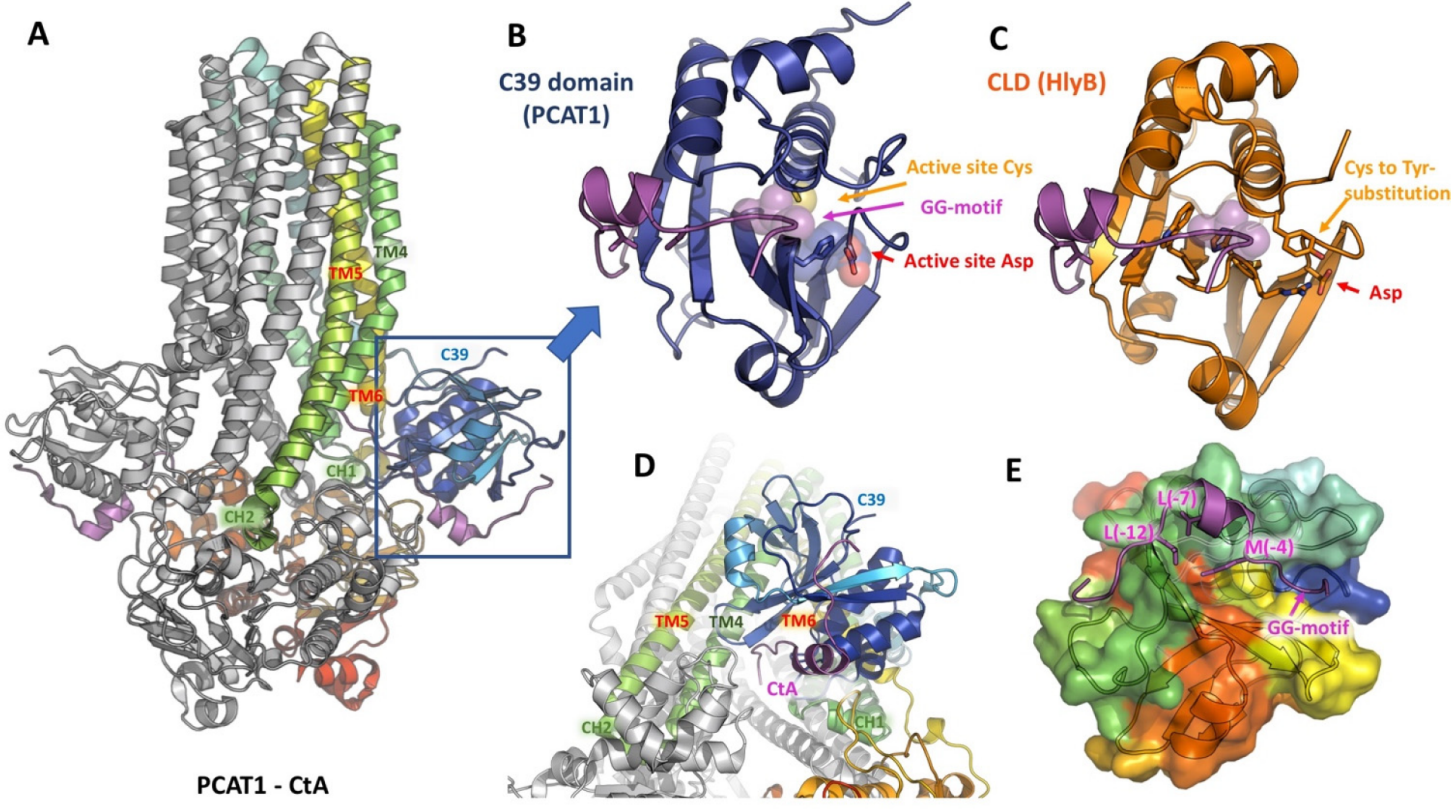
PCAT–cargo interactions mediated by their C39 and CLD domains. (A) Side view of the PCAT1-CtA transporter–cargo complex based on the PDB ID 6 V9Z (Kieuvongngam et al.^505^). (B) Zoomed-in view of the C39 domain highlighting the interaction with the secretion signal of the CtA. (C) Structure of the equivalent CLD domain in HlyB, with CtA superposed into the substrate binding groove. (D) View of the CtA-threading into the substrate binding cavity of PCAT reveals the large substrate access cavern delineated by TM4 and TM6. (E) Position of the cargo targeting motif of the CtA on the surface of the C39 domain, indicating the location of the conserved hydrophobic residues which provide the docking and the GG-motif where the proteolytic cleavage occurs.

C39-domains have also been revealed in the structures of the PCAT1 transporter both in the nucleotide free apo-state (PDB ID 4RY2), in the ATP-analogue occluded state (PDB ID 4S0F),^273^ and in complex with the cognate substrate CtA peptide (PDB ID 6 V9Z),^505^ all showing close correlation with the one seen in CvaB and confirming the earlier cargo-binding model derived from the isolated C39-domain^507^ (Figure 14). As predicted, the peptide signature sequence (Leu(-12)-(X)_3_-Glu(-8)-Leu(−7))^484^ makes a number of specific interactions based on the conserved residues in the C39-domain which facilitates its orientation for processing (Figure 14, panel E). However, intriguingly the peptide-bound structure of PCAT1 presented asymmetric positioning of the cargoes, with one being fully threaded into the transporter suggesting a functional asymmetry. The C39-domain appears to orient the cargo, feeding the C-terminus first into a cleft formed by the TM helices 4 and 6 leading into the large central cavity of the transporter (Figure 14, panel D). Consistent with the biochemical data, the cargo is not seen making specific contacts with the TMD, which may explain the apparent promiscuity of the PCATs.^505^ The energetic implications of the CtA-bound structure are discussed in some detail in the following sections.

A number of Gram-negative PCAT-type transporters seem to possess “C39-like domains” or CLDs, which lack the required proteolytic function,^3^ especially the ones associated with the export of the so-called “repeats-in-toxin” (RTX) cargos. These CLDs present different noncatalytic amino acids; e.g., in the exotoxin-translocating ATPase PaxB (UniProtKB: Q9RCG7), which is part of the PaxA secreting PaxBD complex, a Y13 is present *in lieu* of a catalytic cysteine. The leukotoxin translocation ATPase LktB from *Mannheimia haemolytica* presents a L10, while an N9-residue is found in place of the cysteine residue expected in the CLD of the hemolysin B (RtxB) transporter from *E. coli* O157:H7 (UniProtKB: Q46717). While this is generally the rule, not all RTX-repeat transporters have disabled C39-domains, e.g., CyaB involved in the transport of calmodulin-sensitive adenylate cyclase-hemolysin (cyclolysin) (UniProtKB: P0DKX5).^33^

One of the best-studied examples of a CLD-containing PCATs is HlyB (UniProtKB: P08716), which is part of the T1SS involved in the secretion of α-hemolysin (HlyA).^508^ The N-terminal domain of HlyB shares ∼40% homology to C39 peptidases, but critically, this “C39-like domain” or CLD displays a degenerated proteolytic site, with a catalytically essential cysteine^504^ being replaced by a tyrosine (Y9).^509^ Consistent with this, HlyA does not contain an N-terminal leader peptide or a GG motif and is not cleaved during or after the transport, suggesting an alternative mechanism is in action for its secretion. Compared to the catalytical histidine of ComA-PEP (H96), the equivalent histidine of the HlyB-CLD (H83) is flipped by 180° and the solution NMR structure of the HlyB-CLD (PDB ID 3ZUA)^506^ revealed a π–π stacking between the H83 and W77 residues, which might be a characteristic feature of CLDs, as the tryptophan residue in the equivalent position is conserved across the family. This π–π stacking requirement explains why reintroduction of Cys at position 9 (Y9C) does not restore the proteolytic activity of the CLD. Despite the apparent homology and the similar overall fold between the C39-domain seen in the Streptococcal peptidase ComA,^484^ and the HlyB-CLD, which results in an overall similar fold, the CLD of HlyB represents a particular protein class with a distinct binding site imposing specificity for the RTX-toxin HlyA^506^ with the binding pocket for the latter being located on the opposite side compared to the substrate-binding site of ComA-C39. Notably, all PCAT transporters containing an active C39-peptidase domain appear to transport relatively small substrates with an upper MW limit of ∼10 kDa, belonging to the bacteriocin family that possesses an N-terminal, cleavable leader peptide.^506^ In contrast, CLD-containing PCATs are associated with the transport of large cargoes of above 50 kDa, which contain a C-terminal secretion signal and, importantly, belong to the RTX (repeat in toxin)-toxin family. Thus, the existence of an RTX-domain in the transported substrate can be correlated to the presence of a CLD in the cognate PCAT transporter and *vice versa*.^506^

Importantly, despite the lack of proteolytic activity, the CLD of HlyB appears necessary for the recruitment and tethering of the unfolded substrate, playing chaperone-like.^506^ The CLD of HlyB was shown to tether its cargo HlyA, preventing its aggregation and/or degradation during secretion, playing the dual role of a cargo recognition as well as a chaperoning unit.^506^ Crucially, combining these data with more recent findings^507^ suggests that the CLD may also play a role in stabilizing the nucleotide-free state of HlyB by binding the substrate peptide, allowing it to optimally position its cargo for export upon ATP-binding.

In some cases, such as the multifunctional autoprocessing RTX-like (MARTX) (e.g., the *Vibrio cholerae* actin-directed RTX toxin (Q9KS12)^510^ and Clostridium sp. glucosylating toxin families the *Vibrio cholerae* actin-directed RTX toxin (Q9KS12)^510^), the cargoes themselves display an autoprocessing in cis mediated by an internal C80 peptidase domain, which becomes activated upon binding to the eukaryotic-specific small molecule, inositol hexakisphosphate (InsP(6)), present in the target cells.^511^

Intriguingly, there is also a sizable group of T1SS-associated PCAT-like ABC transporters that lack a CLD or a C39-domain altogether.^3^ One such example is HasD, which is part of the T1SS of *Serratia marcescens* which secretes the hemophore HasA.^153,512^ HasA is not cleaved during the transport and contains no RTX domain, and consistent with the observations of Lecher et al.,^506^ HasD does not possess an N-terminal domain. Furthermore, the cellular chaperone SecB is required for the productive HasA secretion by the T1SS by inhibiting its folding and maintaining the unfolded, secretion-competent state of HasA.^513^ Nevertheless, HasA contains primary recognition sites that interact with HasD prior to the secretion, likely within the substrate binding cavity of the transporter, although their exact identity remains obscure.^512^ Other notable members include LipB from the *S. marcescens* lipase T1SS (UniProtKB Q54456)^514^ and PrtD from the protease secretion system PrtDEF from *Aquifex aeolicus* VF5 (UniProtKB O67184),^515^ both of which are discussed below.

#### 5.6.6 NBDs of PCAT Transporters

**Figure 15:**
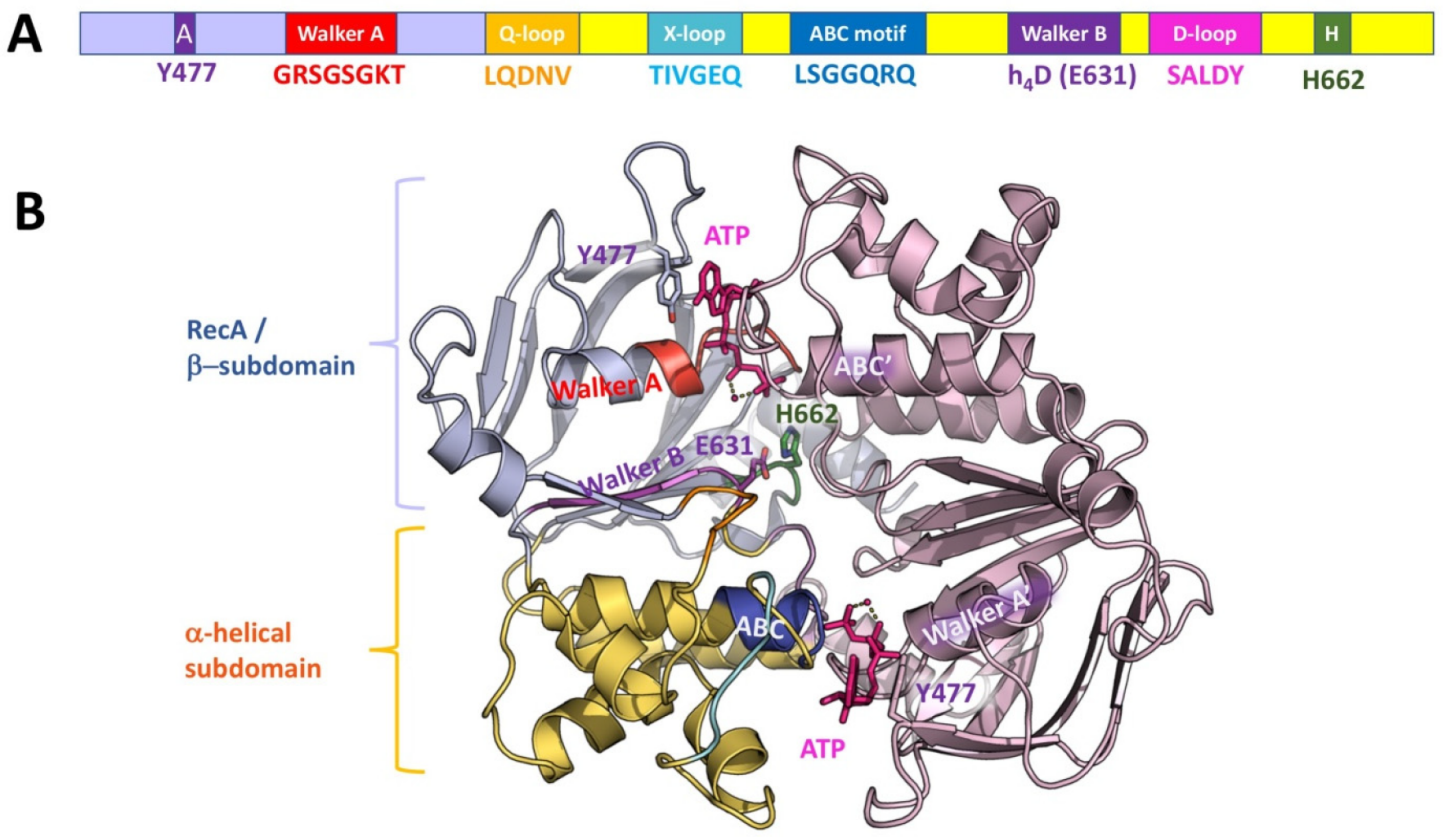
Structure of the ABC transporter nucleotide-binding domains (NBDs). (A) Linear graph from the N- to C-terminus of the typical NBD domain, using HlyB as a template. (B) View of the dimerized NBD based on the crystal structure of the ATP-bound HlyB monomer (PDB ID 1XEF),^516^ highlighting the positions of the conserved sequence motifs involved in nucleotide binding. In one subunit, the RecA-like and β-sheet domain is colored pale blue, while the α-helical subdomain, harboring the ABC-motif, is colored yellow. The second NBD protomer is colored wheat. Motifs are presented in colors matching the sequence boxes in panel A. For illustrative purposes, the side chain of the catalytic H662, which is substituted by an alanine in the above structure to stabilize the ATP-bound state, is being modeled in. The side chains of Y477 (loop A; coordinates the adenine ring of ATP); the E631 (the invariant Glu-residue following Walker B, which helps to coordinate the ATP) and H662 (H-loop) are presented as sticks. The Walker B motif usually features four hydrophobic residues that form a β-strand and is terminated by a conserved Asp (in this case D630), which coordinates the catalytic Mg^2+^ cofactor via water-molecule mediation. Two ATP molecules are colored in purple.

The NBDs of HlyB (PDB ID 1XEF,^509^ PDB ID 2FF7(516)), LipB (the ABC transporter involved in the T1SS that secretes the lipase LipA in *S. marcescens*) (PDB ID 5X7K),^514^ and PrtD (PDB ID 5L22)^515^ have been solved and present a structure that is generally identical to that of the canonical type IV ABC transporters. ATP-binding promotes dimerization of the HlyB-NBD.^517^ Indeed, the HlyB NBDs has been solved in an ATP-bound state (PDB ID 1XEF),^509^ presenting a dimer in which two intact ATP molecules are bound at the interface of the Walker A motif and the C-loop provided by the opposite protomers (Figure 15). Biochemical data suggests a mechanism of substrate-assisted catalysis, rather than that general base catalysis might operate in the HlyB-type ABC-ATPases. Two highly conserved residues, namely E631 and H662, form a catalytic dyad, with H662 playing the role of a “linchpin”, that coordinates a complex network of intermolecular interactions, and were found to be required for interprotomer communication, that covers ATP, water molecules, as well as Mg^2+^ ions and amino-acids both in cis and trans orientation (Figure 15).^509^

A couple of follow-up studies^516,518^ revealed the kinetics of ATP-driven NBD association alongside the structures of the ADP-bound form of HlyB NBD, in wild-type (PDB ID 2FF7), as well as in mutant forms of the transporter (PDB ID 2FFA; 2FFB) allowing us to complete the functional cycle of the transporter. Cavity analysis of dimeric ATP-bound NBDs indicates that inorganic phosphate should not be able to diffuse freely out of the binding site, and its exit requires an additional conformational change in the domain.^516^ Intriguingly, these structures also revealed a structural asymmetry within the ATP-bound NBD dimer in the form of one open and one closed phosphate exit tunnel. This tunnel gate is controlled by a salt bridge between D551 and a R611-residue, the side chain of which flips open to allow diffusion of the inorganic phosphate. Analysis of the structures, and the loss of cooperativity upon mutation of these conserved residues, strongly suggests a sequential ATP-hydrolysis is taking place;^516^ however, whether this sequential release of ADP and Pi may be connected to the processivity of the transporter remains unclear. ATP-hydrolysis (∼ –30 to −35 kJ/mol) and NBD dimerization (−33.4 kJ/mol) are comparable from an energetic point of view, and thus either step could represent the potential “power stroke” during the reaction cycle of the ABC transporters. In addition, both ATP binding and ADP-Pi dissociation could contribute to the power stroke.^516,519^ In the case of HlyB, the cycle has been suggested to take two distinct steps, with the *mechanical energy* being derived from the rigid-body motion of the α-helical subdomain and the rearrangement of the N-terminus of the Walker A motif associated with its binding of ATP and associated NBD-dimerization. This energy is transmitted to the TMDs likely used as a power-stroke. The second stage is suggested to employ the *chemical energy* of sequential ATP-hydrolysis, which is used for the recycling of the dimer by enabling the ADP dissociation. Analysis of the ADP-bound state of the HlyB-NBD revealed a distortion of helix-6, and it has been suggested that the tilting of this helix is used as an enthalpic energy storage device, with the elastic energy being possibly used for programmed release of ADP from dissociated NBD protomers and additional transmission to the TMDs to complete substrate translocation.

The archaeal ABC transporter MJ0796 *from Methanocaldococcus jannaschii*(520) has been linked to the LolD from the LolCDE system and is the closest NBD homologue to the *E. coli* LolD, with ∼50% identity and 70% similarity between the two. Due to the close sequence similarity, its NBD may be a representative of MacB-family transporters, and intriguingly the dissociation of the NBDs upon ATP-hydrolysis seems to differ from that observed in the HlyB-family. While in the HlyB no significant increase in negative charges accompanying ATP-hydrolysis was detected within the ATP-binding site of HlyB;^516^ the dissociation of the NBDs in the LolD/MacB family appears to be driven primarily by the active electrostatic repulsion.^520^ This discrepancy may reflect the different mode of substrate engagement between the two respective transporter families, with LolD/MacB operating a discontinuous, single-step extraction of the substrate, while the HlyB is required to progressively thread its substrate over multiple cycles.

#### 5.6.7 Structure of AMS/PCAT Family Transporters

Structurally, the most studied member of the PCAT-family is the PCAT1 from the thermophilic Gram-positive bacterium *Clostridium thermocellum (aka. Hungateiclostridium thermocellum)* (UniProtKB - A3DCU1), which is responsible for the secretion of the small cargo protein CtA. CtA is encoded in the same operon as the main transporter and represents a 90-residue polypeptide, containing an N-terminal signal sequence (leader peptide), which is cleaved by the C39 domain of the PCAT1, to produce a mature, transport-competent cargo of 66 residues^273,505^ (Figure 14).

Each PCAT1 protomer consists of an N-terminal C39-domain, a transmembrane domain (TMD) containing 6 TM helices, and a C-terminal NBD, containing the conserved signature sequence-motifs for ATP-binding and hydrolysis.^273^ The ATPase activity of PCAT1 was found to be low and rather insensitive to the presence of substrate, while its peptidase activity was found to be negatively impacted by the addition of ATP (ATPγS). Furthermore, at the same enzyme/substrate ratio, the isolated C39-domain appeared to cleave the substrate 80% more slowly than the nucleotide-free full-length PCAT1, while the addition of ATP inhibited the proteolytic activity of the full-length PCAT1 by 90%, to a level comparable with the isolated C39-domain.^273^

The structure of the PCAT1 shows that the two TMDs from the functional dimer form a large α-helical barrel, with a rhomboidal cross-section and an area of approximately 440 Å^2^ traversing nearly the entire lipid bilayer. Its size is large enough to allow for a small protein cargo such as CtA to be accommodated even in a folded state. Consistent with that, the inside of the translocation pathway is lined with charged residues creating a *hydrophilic* environment for the cargo protein.^273^ The two C39 cysteine-protease domains are positioned at the opposite ends of the helical-core at the entryway of the TM-barrel and are loosely associated with TM3, TM4, and TM6 and make weak contacts with the NBDs (Figure 12B, D and Figure 14).

The apo-structure of PCAT1, obtained in the absence of ATP, reveals an inward facing (IF) conformation, with its two NBDs in a loosely packed dimer primed for ATP binding, while the cargo-binding cavity is open to the cytosol and accessible to the cargo at the exact site where the C39-peptidase domain is docked.^273^ The structure of the catalytically inactive E648Q PCAT mutant bound to ATPγS, reported by the same group, shows an occluded TMD cavity and presents a supposed catalytic intermediate highlighting the conformational changes associated with ATP-binding. It reveals rearrangements of the TMDs that involve the movement of TM3 and TM6 toward the center of the α-barrel, resulting in disruption of the binding interfaces of the C39-domains and their displacement and disengagement from the translocation pathway.^273^ Importantly, the PCAT1 has been shown to be proteolytically active in the absence of ATP, suggesting that in the apo-conformation, while the C-terminal region of the substrate is threaded into the translocation pathway, the N-terminal leader peptide may remain associated with the C39-peptidase domain. Such orientation of the substrate is speculated to be optimal for proteolytic cleavage to free the cargo from the leader peptide. Once cleavage has taken place, ATP binding would induce closure of the cytoplasmic opening and dissociation of the peptidase domains. Consistent with such an interpretation, the lower protease activity in the ATP-bound form, in which the C39-domains are disengaged from the translocation pathway, could prevent cleavage of the substrate when it is not in position for translocation.^273^

#### 5.6.8 Models of the Mechanism of Cargo Transport of the PCAT Type ATPases

The cryo-EM structure of the PCAT1–CtA complex, solved to 3.4 Å resolution, reveals two copies of CtAs to be bound to the transporter C39-domains via their N-terminal leader peptides, but only one of these presents density extending into the TMD cavity of the transporter and hence is positioned for productive cleavage and translocation^505^ (Figure 14A, B, D). This structure has provided a possible mechanistic model of the complex assembly and cargo processing, which is consistent with the general “alternating access” allosteric model of ABC transporter action,^390^ exemplified by canonical type IV ABC transporters such as P-glycoprotein (ABCB1),^465^ TM287/288,^476^ and MsbA.^464^

In the model proposed by Kieuvongngam et al.,^505^ the substrate cargo-protein is recognized by the C39-domain of the transporter via specific interaction with its leader sequence with the transporter being present in an inward facing conformation. This allows the cargo-portion of the CtA to get recruited into the transporter cavity, following which the cleavage of the signal occurs, possibly concomitantly with the binding of the ATP, which causes the outward-facing conformation of the transporter to be stabilized, allowing the now cleaved cargo to leave the cavity. Following cargo release, the TMDs isomerize to create an “occluded form” of the cavity, which is sealed from both sides. Subsequent ATP-hydrolysis resets the transporter conformation to inward-facing (IF), releasing the prebound signal peptides and allowing the C39-domain to redock at the TMD–NBD interface where it can recruit another cargo.

Importantly, this newly proposed model^505^ implies that the substrate specificity of the transporter is conferred primarily, if not exclusively, by the interaction of the C39-domains with the leader peptide, and the transporter TMD essentially does not otherwise interact with the cargo. In that, the model of Kieuvongngam et al.^505^ differs from the ones described for other classical type IV ABC exporters, such as P-glycoprotein (Pgp/ABCB1), which show that the TMDs of the transporter clearly interact with their cargoes, e.g. as evidenced by the substate-bound structure of Pgp.^521^ It has to be noted that the model proposed by Kieuvongngam et al.^505^ has also been contradicted by the recent findings of Rahman and McHaourab,^522^ who used spectroscopic detection of the fluorescent (bimane) and spin-labeled protein substrate of the PCAT1 as it passes through the transporter. These results suggested that the cargo protein binds specifically to the core of the transporter in the inward-facing (IF) conformation and is modulated by ATP binding. This resulted in a different, “cargo-centric” model of transport by PCAT1,^522^ in which the recognition and binding of the leader peptide of the cargo-protein by the C39-domain enables interactions of the transported fragment of the cargo protein with the TMD of the transporter, which is subsequent to cleavage of the leader peptide. Importantly, binding of the cargo to the TMD was reportedly detected in transporter variants lacking C39 proteinase domains, suggesting that while the C39-domain facilitates cargo recognition and orientation the interaction of the cargo with the TMD is the main contributor to affinity. This alternative model of transport suggests that there is an ATP-dependent equilibrium binding of the cargo to the TMB domain, which is speculated to transition to an outward-facing (OF) conformation of the TMB leading to a release of the cargo.

**Figure 16:**
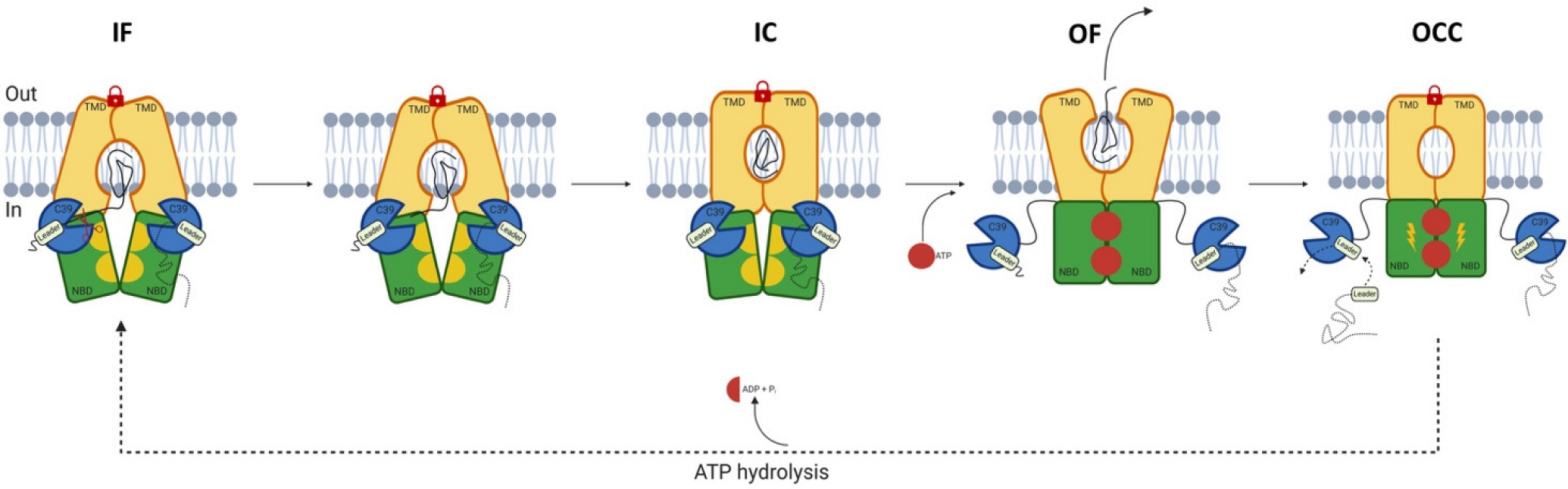
Modified alternating-access mechanism of PCAT1. CtA is recruited and cleaved in the *inward-facing (IF) conformation*. In the model by Kieuvongngam et al.,^505^ the substrate specificity of the transporter is conferred primarily, if not exclusively, by the interaction of the C39-domains with the leader peptide, and the transporter TMD essentially does not otherwise interact with the cargo. In a modification of the cycle proposed by Rachman and Mchaourab,^522^ the cargo is at the center of the interaction, as cargo interactions have been observed in transporter variants lacking C39-domains. In both cases, the interaction of the cargo proteins with the TMD leads to subsequent cleavage of the leader peptide, and a transient *inward closed conformation (IC)*, similar to that observed in the McjD,^459^ is created, which enables the ATP binding. ATP-binding drives conformational changes in the TMD leading to occlusion of the cargo protein binding chamber, known as *the outward occluded state* (*not shown).* ATP binding stabilizes the *outward-facing (OF)* conformation in which the PEP domains are disengaged. After cargo release, TMDs isomerize to form an *occluded state (OCC)* in the absence of cargo. The energy of ATP-hydrolysis resets PCAT1 back to the inward-facing conformation, allowing PEP to dock into the TMD–NBD interface. Padlock indicates the closure of the outer “gate” of the transporter.

We have attempted to summarize the above data and the proposed models into a noncontradictory general model for PCAT action below, which unites both viewpoints and also takes into account the possible role of the extracellular gate closure below (Figure 16).

The actual role and timing of the ATP-hydrolysis within the functional cycle of the ABC transporters remains an open question, with significant spontaneous conformational transitions from IF-to-OF being observed via an occluded intermediate, e.g., in unbiased molecular dynamics simulations.^476^ Recent data derived from the heterodimeric ABC exporter TM287/288 from the thermophilic bacterium *Thermotoga maritima* suggests that ATP binding alone is sufficient for the IF-OF conversion to occur, which is presumably associated with the active transport of one substrate molecule.^476^ Directionality of transport is suggested to be achieved by an affinity switch of the substrate binding site, which unavoidably undergoes a conformational transition due to rearrangements of TM-helices during the switch from an IF to an OF conformation. Using a synthetic single domain antibody as conformational probe and a combination of spin-label, it was shown that the ATP-occlusion firmly traps the transporter in the OF state, preventing transporter cycling.^476^ They further demonstrate that efficient extracellular gate closure, which is based on interactions of charged residues from TM1s of the opposing protomers, is required to dissociate the NBD dimer after ATP-hydrolysis to reset the transporter back to its inward-facing state. In that, the ATP-hydrolysis appears to be strictly required to initiate dissociation of the closed NBD dimer, and once the ATPs are hydrolyzed the mechanical force of this firmly sealed extracellular gate (termed closed D-lock) is required to dissociate the NBDs.^476^

In the extreme thermophile Gram-negative bacterium *Thermus themophilus*, only a single T1SS has been identified, which is based around the heterodimeric *Thermus thermophilus* multidrug resistance proteins A and B (TmrAB), in which the TmrB component has a degenerate NBD-binding site.^478^ E523 from the NBD of TmrA is essential for rapid conversion of the ATP/ATP-bound complex into its ADP/ATP state, whereas the corresponding residue in TmrB (D500) was found to play only a regulatory role.^478^ Swap of active residues between the NBDs did not result in rescue of the function, suggesting a built-in asymmetry of the complex. The prevalence of heterodimeric ABC half-transporters, especially among eukaryotic organisms, suggests an underlying functional principle of NBD asymmetry. TmrAB not only shares structural homology with the antigen translocation complex TAP but is also able to complement antigen processing in human TAP-deficient cells.^523^ Recent cryo-EM structures of the TmrAB in different states and conformations,^479^ which could be attributed to two inward-facing (IF), four outward-facing (OF), and two asymmetric posthydrolysis states, have provided significant insight into the general conformational cycling of the ABC transporters. Using single-turnover assays, Stefan et al.^524^ have been able to further analyze the cycle of the TmrAB reconstituted in lipid nanodiscs at single-liposome resolution. Strikingly, the analysis of the data suggests that a single conformational switch by ATP-binding drives unidirectional substrate translocation. Under this new model, the ATP-binding initiates the IF-to-OF transition and is stoichiometrically coupled to substrate translocation in a single step. Along with this “power stroke” caused by occlusion of two ATP molecules, the substrate-binding site is being remodeled, priming substrate release on the extracellular site. Following this “power stroke”, ATP-hydrolysis and subsequent phosphate release launch the conformational transition of OF-to-IF and hence the return to the resting state, which facilitates nucleotide exchange and thus in turn primes a new round of substrate binding and translocation.^524^ This model contrasts the previously proposed steady-state models, as the utilization of the single-turnover assays reveals a power stroke in substrate translocation and the tight chemomechanical coupling in these molecular machines. Furthermore, the recent structure of the lipid-linked oligosaccharide (LLO)-flippase PglK essential for asparagine-linked protein glycosylation in *Campylobacter jejuni*, in the outward facing conformation, strongly suggests that the release of LLO to the outside occurs before ATP-hydrolysis and is followed by the closing of the periplasmic cavity of PglK.^525^

While further details are needed to fully populate the conformational cycles of the ABC transporters, these results strongly suggest that ATP-hydrolysis is required to drive the transport cycle at the resetting step from the OF to the IF state. As we will in the later sections, such energy dependency may have parallels with the pump cycle of the RND-transporter-based tripartite assemblies.

#### 5.6.9 PCATs Associated with T1SS

The transport mechanisms discussed above, while possibly applicable to bacteriocin transporters in Gram-positive organisms, do not appear to be feasible for the PCAT transporters operating as part of a T1SS in Gram-negative bacteria, as these are often carrying cargoes in excess of 8000 amino-acid long and over 1 MDa in weight,^3,18,449^ while in *S. enterica*, the giant nonfimbrial adhesin SiiE (595 kDa), exported by the *Pathogenicity Island 4-encoded type I secretion system* (SPI4-T1SS), is the largest protein in the *Salmonella* proteome and consists of 53 repetitive bacterial immunoglobulin domains (Big).^526^ As known for other T1SS substrates, such as *E. coli* HlyA, Ca^2+^ ions bound by conserved D residues within the BIg domains stabilize the protein and facilitate secretion.^527,528^ Exporting such cargoes would require a departure from the classical “alternate access” mechanism discussed above in favor of some form of continuous threading. Furthermore, several lines of experimental evidence suggest that T1SS substrates must be unfolded in order to be translocation competent,^529,530^ resulting in the length of an unfolded T1SS substrate that would far exceed the length of the transporter TMD cavity itself. These observations imply that T1SS ABC transporters must operate by a distinct mechanism, where the unfolded polypeptide is likely progressively threaded through the TMD of the transporter over multiple ATP-hydrolysis cycles.^3,449,512^ These hypotheses were confirmed by the structure of the first ABC transporter associated with T1SS—namely the *Aquifex aeolicus* PrtD (*Aa*PrtD) coded by the *abcT5* gene (PDB ID: 5L22; UniProtKB: O67184).^515^ The PrtD is the ABC transporter within the protease secretion system PrtDEF, best known from the phytopathogen *Dickeya dadantii* (*formerly known as Erwinia chrysanthemi*),^531^ responsible for the secretion of a number of proteases encoded in the same operon—namely, PrtA, PrtB, PrtC, and PrtG.^532^ Secretion of the *Dickeya dadantii* protease DdPrtG across the plasma membrane requires a fully assembled PrtDEF complex, and secretion of PrtG *in vivo* requires ATP-hydrolysis by PrtD.^515^

**Figure 17:**
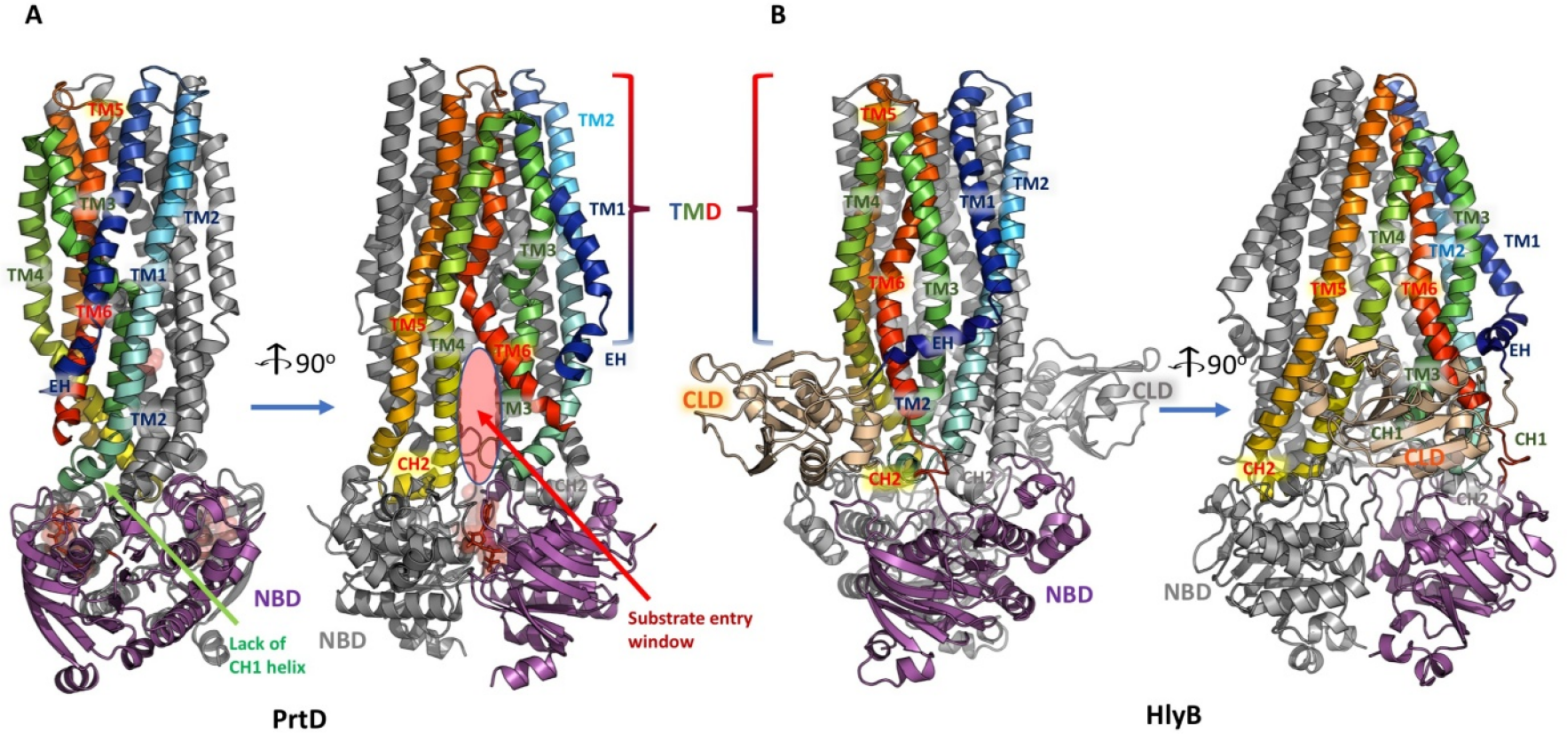
General organization of PCAT transporters associated with T1SS. (A) *Aa*PrtD based on the PDB ID 5L22. Note the highly kinked TM3 and TM6 and the supposed “substrate entry window”. Unlike the PCAT transporters discussed previously, the *Aa*PrtD does not present a wide substrate binding cavity and is suggested to transport unfolded cargoes. The ADP moiety (in red spacefill) is found bound to the protomers. Note the lack of CH1 helix at the end of TM2. (B) Homology model of HlyB, highlighting features common to both PCAT1 and PrtD. The CLD-domains are predicted to aid positioning of the cargo into the substrate entry window.

The *Aa*PrtD structure was revealed in an occluded, ADP-bound state,^515^ and despite lacking C39 or CLD-domains,^3^ the structure is significant as it provides a blueprint for the wider RTX-associated subfamily of T1SS transporters and it reveals an overall organization, which while similar to the standard PCAT transporters such as the PCAT1 discussed above, has a number of distinct structural features. In contrast to the other PCAT transporter structures available to date, e.g., PCAT1, the hydrophilic TM cavity of which allows at least partial folding of the cargo,^273^ the substrate channel of PrtD has been shown to be very narrow and to run throughout the structure for some 40 Å, spanning the entire membrane and ending just shortly before the periplasmic exit. In that it shows analogies to the SecY channel^533^ and due to its steric restrictions appears unable to accommodate folded substrate (Figure 17). The structure also suggests a “substrate entry window” just above the NBD domain formed by the TM helices 4 and 6, which is gated by a conserved R307 on the cytoplasmic side,^515^ while the periplasmic opening is gated by a hydrophobic seal, similar to the “pore-ring” of the SecY channel.^534^

*Aa*PrtD also shows a distinct architecture of its TMD, with uniquely kinked TM3 and TM6 helices near the cytosolic solvent–lipid interface. These kinked helices point toward the dimer interface, creating a highly basic concave “bowl” on the PrtD surface formed by TM1, TM3, and TM6. Furthermore, the coupling helix between TM2 and TM3 (CH1), which packs against the NBD of the same subunit, was found to be replaced by an extension of TM2 and an extended loop leading into TM3 presenting a unique architecture among type IV ABC transporters.^515^ It is notable that both the “concave bowl” and the aforementioned “substrate entry window” are highly basic, which is speculated to play a role in electrostatic stabilization of the T1SS substrates which tend to be fairly acidic. The absence of the coupling helix leading into TM3 is suggested to reflect substrate entering from the cytosolic window between TM4 and TM6. Thus, while the *Aa*PrtD architecture is similar to the classic 6 + 6 TMD dimer observed in the type IV transporters, the shape and the character of the transporter conduit channel differ markedly from the canonical peptide transporters discussed in the previous sections^515^ (Figure 17).

The structure of *Aa*PrtD also hints at specific adaptations that are relevant to the formation of the tripartite T1SS and shared with other such transporters, including the hemolysin A transporter HlyB and the related *Mannheimia* (formerly *Pasteurella*) *haemolytica* leukotoxin pump-associated ABC transporter LktB,^535^ both of which serve as prototypes pumps for a widely disseminated family of virulence factors, known as the RTX (repeats-in-toxin) cytotoxins. To facilitate the analysis, we have created a hybrid homology model of HlyB based on the *Aa*PrtD and PCAT1 structures (Figure 4 and Figure 17).

In comparison with the peptide transporters, the cargo pathway in the T1SS-associated PCATs is strikingly different and much more restrictive, which is achieved by the aforementioned kinks in the TM3 and TM6 leading to constriction of the substrate channel. These kinks are stabilized by conserved sequence motifs, which are shared by the T1SS ABC transporters but not found in peptide PCAT transporters—e.g., the TM3 helix is split into three sections, with the TM3a-TM3b kink being enforced by an “FxT-motif”,^515^ which in *Aa*PrtD is formed by F128 and T130, while in HlyB the corresponding residues are F264 and T266. A second conserved motif is present on TM6 in the form of a bulky hydrophobic (usually W; W295 in PrtD; W431 in HlyB), followed by a hydrophilic residue (R296 in *Aa*PrtD; Q432 in HlyB). These motifs stabilize the kinks via a network of specific inter- and intraprotomer interactions.

In addition, HlyB seems to share with the *Aa*PrtD a hydrophobic “gasket-ring”, on the periplasmic side (PrtD M43, Y47, I425; PCAT I190, F194, L425/L426; HlyB F174/F175, M179/L410/I411) that is speculated to prevent dissipation of the proton gradient during transport and the basic-residue gating (PrtD R307/HlyD R443) at the putative substrate entry window. The orientation of the CLD-domain of the HlyB positions its N-terminal tail in direct proximity to the entry of the predicted substrate entry window, which is consistent with the position of the threaded peptide seen in the CtA-bound PCAT1.^505^

In summary, the recent structural snapshots discussed above strongly suggest a common overall cargo-selection and substrate-feeding mechanism between the PCATs associated with peptide transport and those participating in the T1SS. Unlike the peptide-transporting PCATs, the latter do not allow folding of the substrate throughout the single transporter cycle and necessitate multiple processive steps to transport the whole cargo protein, in a mechanism that requires further elucidation.

### 5.7 MacB/FtsX Transporter Family

Initially identified via a bioinformatic search as one of five putative ABC transporter clusters in the *E. coli* genome,^536^ the *macAB* genes were originally designated *ybjYZ*. The genes were later renamed to *macAB* (macrolide-specific ABC-type efflux carrier) as the pump was reported to transport several macrolides.^60^ However, the relevance of this function *in vivo* remains somewhat questionable, as in *E. coli* it is only pronounced in cells with a deactivated primary AcrAB multidrug efflux pump, and the loss of the orthologous system in *Neisseria gonorrhoeae* did not lead to a pronounced decrease in resistance to azithromycin or erythromycin.^78^ In addition, the MacAB requires TolC for its function to form a tripartite system.^60^ Related tripartite systems have been identified in a number of Gram-negative organisms, such as MacB*sm* of *Stenotrophomonas maltophilia*(116) and the PseEF pump of *Pseudomonas syringae*.^537^

Strengthening the case for the MacB-family members as *bona fide* multidrug transporters is the *E. coli* heterooligomeric YbhFSR, which appears to function with the PAP coded by the *ybhF* gene (P0A9U1).^538^ This tripartite system can efflux cephalosporins, including the third generation cefoperazone. Recently, Feng et al.^62^ characterized the YbhFSR system and found it to export tetracycline, oxytetracycline, chlortetracycline, doxycycline, EtBr, as well as the Hoechst 33342 dye. Furthermore, uniquely for the ABC-family, the findings of Feng et al.^62^ suggested that YbhFSR exhibits a dual function both as a MDR efflux pump and as a Na^+^ (Li^+^)/H^+^ antiporter, although this requires further investigation, especially in light of the rather high homology of the YbhFSR protomers with MATE transporters such as NorM.

#### 5.7.1 Biological Functions

**Figure 18:**
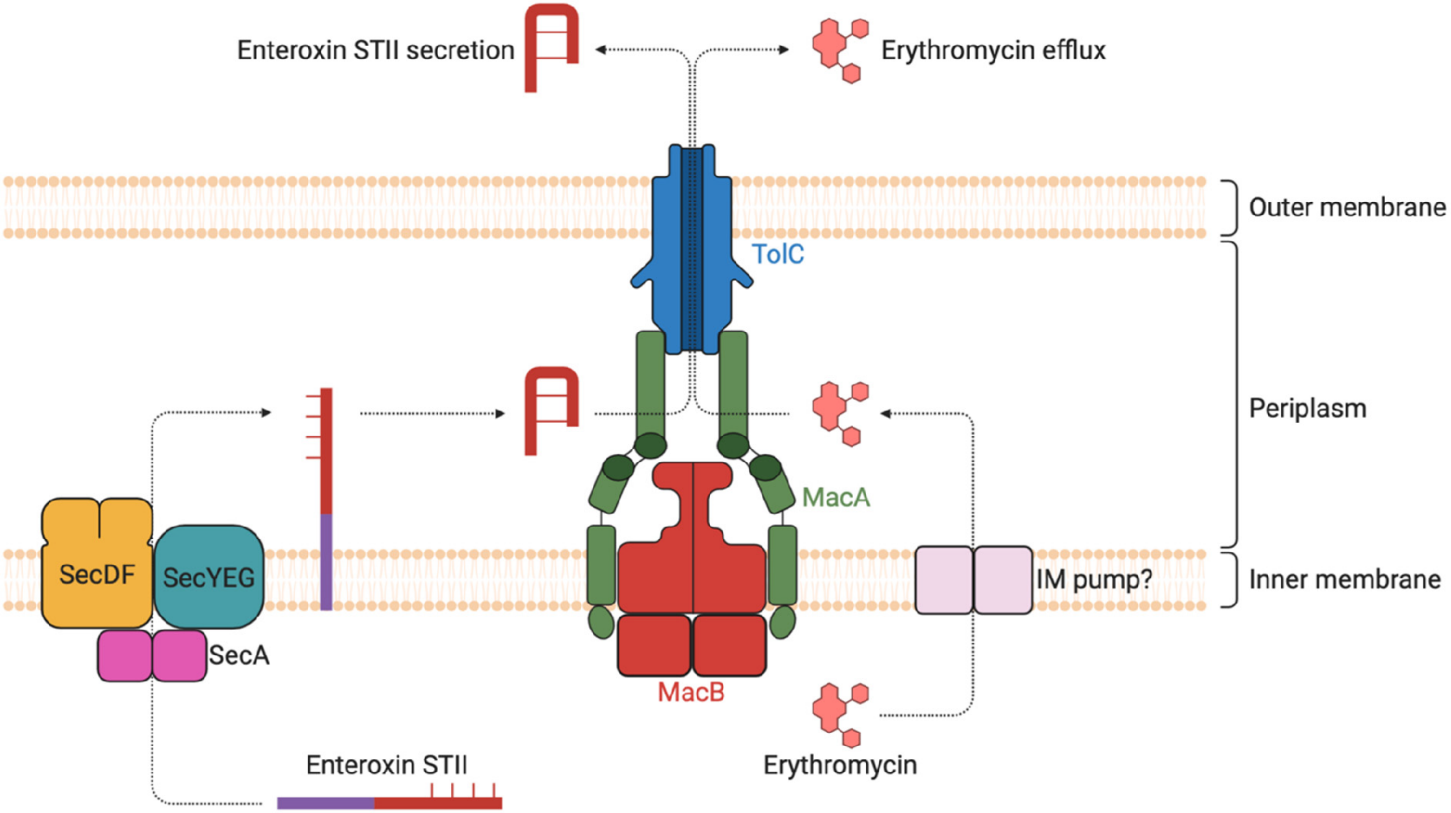
The function of the tripartite pump MacAB-TolC in toxin secretion and macrolide resistance. MacAB secretes ribosomally synthesized and post-translationally modified peptides (RiPPs), such as enterotoxin STII, that are targeted and exported to the periplasm by the SecY-secretion signal (purple). Following cleavage and disulfide bond formation in the periplasm, the toxin is then secreted out by the MacAB-TolC. Figure modified based on Crow et al.^431^

In addition to xenobiotics, the MacB-family pumps are associated with the secretion of a wide range of nonantibiotic targets, including the heat-stable enterotoxin II in *E. coli*(154) and the cyclic lipopeptide entolysin in *Pseudomonas entomophila*.^539^ The cargoes range in size from ∼500 Da for macrolides to ∼10 kDa for dispersins,^540^ suggesting low selectivity of the MacB pump. Notably, and unlike the typical T1SS which it resembles, the secretion of the heat-stable toxins by MacB-related pumps appears to be Sec-dependent in the first stage and requires a Sec-secretion signal.^154^ MacB copurifies with and binds specifically to phosphatidylethanolamine, in an interaction that survives native mass spectrometry in the gas phase,^541^ and the related LolCDE system is differentially modulated by different phospholipids^542^ indicating possible lipid transport and/or modulation. MacAB appears to be capable of binding to the core lipopolysaccharide, although its exact role is unclear^543^ (Figure 18).

In a related system in *Pseudomonas syringae*, PseEF was found to secrete the lipodepsinonapeptides syringomycin (*syrB1*) and syringopeptin (*sypA*),^537^ and it was required for the full virulence of the bacterium. The MacAB-TolC pump has also been implicated in the efflux of protoporphyrin IX (PPIX) in *E. coli*, and mutants of *macAB* or *tolC* were reported to accumulated PPIX and become sensitive to photoinactivation.^155^ Also, in *Pseudomonas*, an orthologous system known as PvdRT-OpmQ is involved in the secretion of the primary virulence factor pyoverdine, which is a siderophore, acting as both iron-carrier and a virulence-related signaling molecule.^544,545^ In enteroaggregative *E. coli* (EAEC), an export system for dispersin, a 10.2 kDa protein which promotes bacterial dispersal across the intestinal mucosa^540^ able to convert plasminogen into plasmin,^546^ is encoded by a virulence plasmid pAA and has been linked to an MacB-like pump AatPABCD where the AatP is a permease, AatC is an NBD, and AatA is a TolC-like OMF.^547^

In enterotoxigenic *E. coli* (ETEC), the predicted tripartite complex CexPABC, which consists of the MacB homologue CexP (permease)/CexC (NDB), the PAP CexB and the OMF CexA^548^ appear to be associated with the export of the coat protein and virulence factor CexE, later found in *Yersinia enterocolitica*, *Providencia alcalifaciens*, and *Citrobacter rodentium*.^549^ Intriguingly, despite similarities with the T1SS, this exporter was shown to accept its cargo from the periplasm, where CexE accumulates, and to rely on the general secretory pathway.^548,549^ At the time of writing of this review, the roles of the auxiliary transmembrane proteins CexD and AatD are yet to be established. In *Salmonella* Typhimurium, the nonribosomally synthesized siderophore enterobactin is produced in response to iron starvation and can be processed into salmochelin and several linear derivatives.^550^ Trimers of such linearized enterobactin have been shown to protect *S*. Typhimurium against peroxide-mediated killing in a MacAB-dependent fashion.^550^ Furthermore, in a mouse model of *Salmonella* infection, the deletion of MacB impacted virulence,^101^ while MacAB was found to be required for survival of *Salmonella* in cultured macrophages which produce reactive oxygen species (ROS) but was not essential in macrophages which did not generate ROS.^138^ Consistent with a stress-response interpretation, expression of *macAB* in *E. coli* and *Salmonella* is inducible and regulated by the two-component system PhoPQ,^101,551^ which among other things, senses antimicrobial and pH stress, e.g., responding to acidification of the phagosome.

A large group of MacB-like transporters have been described in the multicellular cyanobacteria such as *Anabaena* and *Nostoc* (for recent reviews please consult Shvarev and Maldener^552,553^) that have been found to associate with diverse functions covering heterocyst development and diazotrophic growth (e.g., HgdBCD system, DevBCA system,^554^ All0809/8/7); iron metabolism (All2652/1); as well as drug resistance (Alr4280/1/2 and Alr3647/8/9).^553,555^ These systems seem to utilize a single OMF HgdD^556^ (see section 7 and associated Figure 33A for details). One of the better studied examples includes the DevBCA-HgdD.^557−559^ The pump component genes are *devB (alr3710)*, coding for the PAP protein; *devC (alr3711)*, coding for TMD; and *devA (alr3712)*, coding for the NBD, respectively, and they are positioned on a single operon. The *Anabaena* could be considered a multicellular organism, as it forms filamentous nonbranching strings of cells called “trichomes”, which, in the presence of combined nitrogen cells are composed mostly of vegetative cells, but in its absence, the *Anabaena* develops specialized cells, called heterocysts,^560,561^ which provide N_2_-fixation capability and transport nitrogen assimilation products to neighboring vegetative cells in exchange for sugars produced by photosynthesis.^562^ These heterocysts build an additional envelope around the outer membrane, which is itself composed of two layers: an external heterocyst exopolysaccharide (hep) layer and an underlying heterocyst glycolipid layer (hgl). The hgl layer is made of heterocyst-specific glycolipids (HGLs) and is airtight, restricting oxygen from penetrating into the heterocyst, creating a microoxic environment which is essential for dinitrogen fixation by nitrogenase.^553,559,561^ DevBCA forms a heterocyst-specific glycolipid exporter that is essential for the export and development of the hgl layer. To transfer glycolipids through the Gram-negative cell wall, DevBCA has to recruit a TolC-homologue protein called HgdD, that is encoded by the gene *alr2887*.^556,563^ The *Anabaena sp. hgdD* mutant, like the *devB, devC, and devA* mutants, does not form the hgl layer and hence cannot grow diazotrophically; that is, they are unable to grow without external sources of fixed nitrogen.^556^ The HgdD OMF protein is central to secretion and efflux in *Anabaena*, as it has been shown to pair with at least seven different DevBCA paralogues, with varied functions. While some of these have partially overlapping functions and are involved in heterocyst-specific roles, at least some of these DevBCA paralogues such as the tripartite pumps formed of the gene products of *alr4280/alr4281/alr4282* and *alr3647/alr3648/alr3649* that code for the components of putative MacB-like ABC exporters have been shown to be essential for resistance to a range of different drugs and efflux of ethidium bromide, while having no demonstrable role in heterocyst development.^564^

In addition, the YbbAP is a poorly characterized member of the MacB-family in *E. coli*,^419^ where *ybbA* encodes the predicted NBD subunit and *ybbP* encodes the predicted permease subunit of this putative exporter complex. Although association with heavy metal export has been suggested,^418^ this member of the family remains poorly understood.

#### 5.7.2 Gram-Positive Homologues of the MacB Transporters

Within the wider family of MacB-like transporters, there are a number of members from Gram-positive organisms; e.g., in *Bacillus subtilis* the permease YxdM, forming part of the ABC transporter YxdLM, appears to be involved in resistance to antibacterial cationic peptides.^565^ Also, in *B. subtilis*, YtrF appears to be a permease forming part of the YtrBCDEF complex that plays a role in acetoin utilization.^566^ While very little is known about the structure of these transporters, our bioinformatic analysis suggests that they possess significantly more expansive PCDs than the rest of the MacB-family, possibly presenting a duplicated PD-module/LolC-like extension.

The *yknzWXYZ* operon in *Bacillus subtilis*(567) has been found to code an unusual four-component transporter system, which is built around the principal YknY (NBD)/YknZ (permease) ABC transporter unit which is homologous to MacB. The system also associates with a cognate PAP (YknX) and a four-TM additional membrane protein (YknW)^568^ to provide protection against sporulation-delaying-protein-induced killing by the 42-residues long disulfide-linked SDP toxin.^569^ The pump appears to protect *B. subtilis* against both endogenous and exogenous SDP, and YknW is critical in the assembly and functionality of the complex, as YknXYZ complex alone does not protect against SDP, while overproduction of YknW alone provides limited protection.

An investigation of the response of *Streptococcus pneumoniae* D39 gene-expression patterns to three antimicrobial peptides—namely nisin, bacitracin, and the human cationic peptide defensin LL-37 identified upregulation of a hitherto uncharacterized operon (*Sp0785–0787*), the deletion of which increased the cell-susceptibility to LL-37 and lincomycin.^570^ Similarly, the PAP, NBD, and translocase coded by the *spr0693-spr0694-spr0695* genes belonging to the same operon in *Streptococcus pneumoniae*(570,571) show high similarity to MacAB, with Dpr0693 having 21% identity with the PAP MacA, whereas the Spr0694-0695 complex is 37% sequence identical to MacB and presents a very similar structure.

#### 5.7.3 Topology and Structural Organization of MacB Family Members

The first investigation of the structural organization of the MacB was undertaken by Kobayashi et al.,^572^ who found that the protomers of the MacB/FtsX family are distinct as their NBDs are found at the N-terminus of the transporter, while their TMDs contain four TM helices thus presenting a unique topology. Parallels in the organization of the known tripartite assemblies suggested that MacB-type transporters may organize into higher stoichiometries; however, cross-linking, atomic-force microscopy, and native mass-spectrometry confirmed that the functional unit of the transporter is organized along the universal dimeric pattern characteristic of the wider ABC-superfamily.^573^

In the monomer of the prototypical family member MacB, the NBD is fused to the N-terminal part of the permease domain of the transporter^572^ and it presents a dimeric architecture; however, a number of other family members have the permease and NBDs coded by separate genes. Examples include the FtsEX complex, which gives the name to the wider MacB/FtsX family. It assembles with a FtsE_2_FtsX_2_ stoichiometry, with a porter domain formed by the FtsX (UniProtKB P0AC30), while the NBDs are provided by the FtsE (UniProtKB P0A9R7).^574^ It has to be noted that despite the close structural homology, and the apparent ATPase cycles,^575^ this complex does not seem to perform a transporter function but is rather engaged in peptidoglycan remodeling, a role which it exerts by activating the extracellular and periplasmic amidases, as demonstrated by the direct recruitment of the EnvC by the periplasmic domains of FtsX.^576^ Similarly, the connected lipoprotein extrusion complex LolCDE, while having a heterodimeric permease portion (LolCE), engages a dimeric NDB LolD, to give a general stoichiometry of LolCED_2_.^432^ All of the permease domains of the proteins described above contain the 4 TM-helix architecture of the MacB. However, more exotic architectures have also been described, e.g., the bacitracin-sensing and resistance-associated BceAB-type transporters^577^ that appear to have 10 TM helices arising from a duplication of the 4-TM FtsX fold over TM2–4 and 7–10 and featuring a central helical insertion over TM5–6. These transporters presumably function as monomeric units. Some other members of the family feature this arrangement, e.g. YbbP-YbbA_2_ complex;^442^ however, these have been poorly characterized.

The first glimpse of the 3D structural organization came from the periplasmic domain of the *Aggregatibacter* (previously *Actinobacillus*) *actinomycetemcomitans* (PDB ID 3FTJ),^578^ which revealed a structural organization distinct from the then available ABC transporter structures. In the past decade, the structural and conformational space for type VII transporters has expanded dramatically to include the complete MacB from several different species of Gram-negative bacteria,^24,272,431^ as well as the Gram-positive homologues Spr0694–0695 from *Streptococcus pneumoniae* (PDB ID 5XU1)^571^ and YknWXYZ from *Bacillus amyloliquefaciens* (PDB ID 5F9Q).^579^ In addition, the related heterodimeric LolCDE lipoprotein sorting system (LolC, PDB ID 5NAA, 6F49;^432^ LolE, PDB ID 5UDF (Abendroth et al., to be published)) and the FtsX family members (PFAM family FtsX (PDB ID 4N8N, 6HEE, 6HFX)^433,434^ have also been determined. Importantly, the complete assembled structure of the tripartite MacAB-TolC from *E. coli* has also become available (PDB ID 5NIL, 5NIK),^272^ allowing a direct comparison with the RND-based assemblies.

#### 5.7.4 General Topology of MacB/FtsX Family and TM Domains

The recent determination of a number of MacB/FtsX family transporter structures^24,272,431,571^ has revealed a number of unique features within the TM region of these transporters, and these have been the subject of several excellent reviews (see Greene et al.^442^ and Murakami et al.^580^).

**Figure 19:**
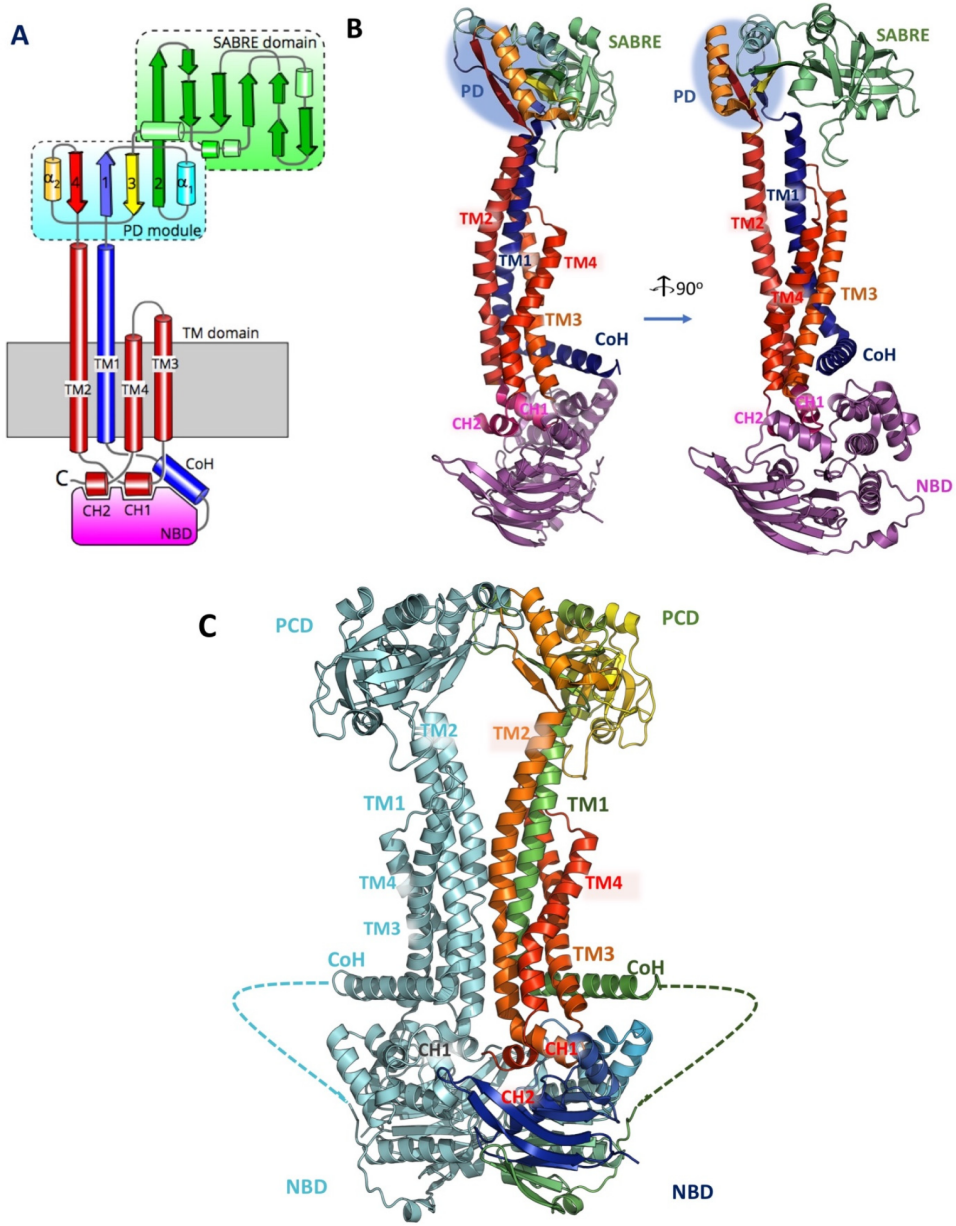
Structural organization of MacB. (A) General topology of the single MacB protomer highlighting the N-terminal nucleotide binding domain (NBD; magenta), the connecting helix (CoH), the transmembrane domain (TM domain) with its four helices, as well as the periplasmic core domain (PCD), which is composed of a PD-module and a SABRE subdomain (green). CH1 and CH2, coupling helices 1 and 2, respectively. (B) Two orthogonal orientations of the single protomer of MacB from *A. baumanii* based on PDB ID 5WS4,^24^ highlighting the principal secondary structure elements. The blue oval delineates the PD. (C) View of the complete MacB-dimer.

First and foremost, the TM-region is composed of only 4 TM helices, with a dimer forming a 4 + 4 helical bundle, which is a stark contrast from the typical 6 + 6 architecture seen in the prototypical ABC family members, such as the type IV family (e.g., Sav1866 and MsbA).^428,581^ Second, TM helices 1 and 2 are longer than the TM3 and TM4 and protrude above the membrane plane into the periplasm, where they are extended into a “stalk-like” structure crowned with a sizable globular periplasmic domain (Figure 19), known as the periplasmic core domain” or PCD,^578^ formed from the large periplasmic loop which is spliced into the periplasmic extensions of the stalk-helices TM1 and TM2, and in this respect resemble somewhat the RND transporters.^572^

The TM3 and TM4 are notably shorter than the stalk-helices and pack outside of the helical bundle formed by them. They do not protrude above the membrane plane and are linked by a short periplasmic loop, which is sometimes referred to as a “shoulder”.^431^ The cytoplasmic, N-terminal part of TM1 is linked to the NBDs by an amphipathic helix, that runs parallel to the membrane surface and a “skirting loop” (Figure 19). The former helix is equivalent to the “elbow helix” seen in the type IV transporters but is commonly referred to as a connecting helix (CoH)^24^ due to its linkage with similar helices in type V ABC transporters, e.g. ABCG5/ABCG8^428,582^

Finally, the NBD domains are located much closer to the membrane than in the typical type IV ABC transporters, which allows close association of the connecting helices with them, providing additional allosteric control. Each MacB TMD provides two coupling helices (CH1 and CH2) for TMD-NBD interdomain communication. The major coupling helix (CH1) is located on the cytoplasmic loop connecting TM2 and TM3 and provides a means of communicating conformational changes from the NBDs to the TMDs, while a second, “minor coupling helix” (CH2) is located after the TM4 at the extreme C-terminus. Deletion of the CH2 in *A. baumannii* MacB had a notable impact on the macrolide resistance^24^ but only a minor effect on the pump activity in the *Ec*MacB.^431^

In a further departure from the architecture of type IV transporters where CH1 and CH2 from one-half-transporter contact the NBDs of the opposing protomer, the coupling helices (CH1 and CH2) are not shared between the protomers; i.e., the coupling helices of MacB/FtsX only engage the NBD of their cognate protomers^24,431^ (see Figure 12 in the PCAT section and Figure 19).

#### 5.7.5 Periplasmic Core Domains (PCDs)

**Figure 20:**
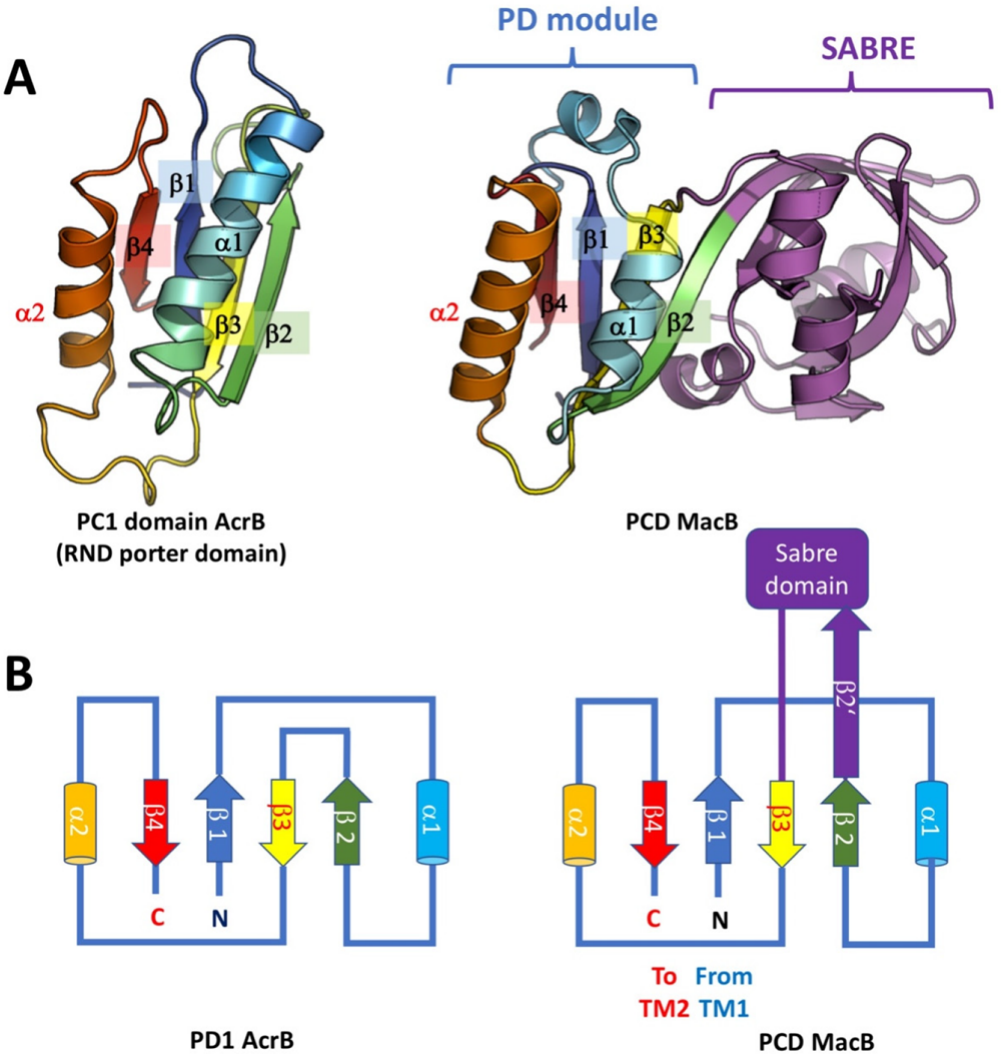
Comparison of the porter domain of RND transporters and PCD of MacB. (A) The lower part of the periplasmic domain (PCD) of MacB shares structural homology with the porter domains (PD) of the RND transporters, as can be seen on the example of the *Aa*MacB (PDB ID 3FTJ)^578^ (right) and the PC1 PD module of AcrB (PDB ID 2DHH)^288^ (left). (B) The topology diagram of the domain indicates that the SABRE subdomain is spliced within the core PD module between the β2 and β3 strands.

As mentioned above, the bulk of the periplasmic portion of the type VII ABC transporters is contributed by a globular “periplasmic core domain” or PCD.^578^ It forms a conserved module, which is presented across the MacB and also FtsX-type transporters^574,575^ allowing to unite them into a separate PFAM group (PF02687) and giving the name to the whole family, so it can often be referred to as MacB/FtsX. The PCD domain could be split into an “upper” and “lower” subdomain,^578^ which have also been named periplasmic subdomain C (PSC), and periplasmic subdomain N (PSN), respectively^272^ (Figure 20), with the lower portion presenting a mixed α-β sandwich of a general (β–α–β–β–α–β) configuration, sharing high structural homology to the porter (PD) domains of the RND transporters,^578^ while the “upper subdomain”, which has become known as the “SABRE” (an acronym for s*mall, alpha/beta rich, extracytoplasmic*), is unique to the wider FtsX family and can vary significantly in structure. In some of the recent literature, the PD and SABRE subdomains have also been referred to as periplasmic subdomain N (PSN) and periplasmic subdomain C (PSC), respectively,^272^ but to avoid confusion, we will stick to the PD and SABRE nomenclature (Figure 20).

Due to its variability, the SABRE subdomain can be used as a differentiator within the wider MacB/FtsX family, and based on this it is possible to subdivide the group into several cohorts. It is notable that in the prototypical members of the MacB family, including the *Aggregatibacter actinomycetemcomitans Aa*MacB,^431,578^*Acinetobacter baumannii Ab*MacB,^24^*E. coli Ec*MacB,^272^ and the Gram-positive transporters belonging to the family, such as the *Streptococcal* permease Spr0695 (PDB ID 5XU1),^571^ the SABRE domain presents a continuous entity, which is spliced into the middle of the “lower subdomain” (PD), splitting it evenly into two β–α–β motifs. Within this “canonical” MacB group, the SABRE forms a pseudocontinuous extension of the PD β-sheet, presenting a semirolled β-barrel.

A number of MacB-related proteins share the extended architecture above, with their PD modules superposing with low RMSD,^431^ and they differ predominantly in the organization of their SABRE subdomains which display considerable diversity; however, as they do not form tripartite complexes, we will not discuss them at length, highlighting some key members and respective PCD-domain peculiarities.

The second group of proteins that give the joint-name to the MacB/FtsX family, the aforementioned FtsEX complex,^574^ is central for peptidoglycan remodeling, a role which it exerts by activating the extracellular and periplasmic amidases, via direct recruitment of the EnvC by the PCD of FtsX.^576^ FtsEX shares similar TM-architecture to MacB, featuring 4 TM-helices and a large periplasmic loop, and it appears to function as a dimeric assembly of (FtsE)_2_(FtsX)_2_ stoichiometry.

In *Streptococcus pneumoniae*, FtsEX interacts with modular peptidoglycan hydrolase PcsB (PBD ID 4CGK),^583^ and the activation of the ATPase activity by FtsE triggers trans-membrane activation of PcsB.^584^ PcsB is a dimer in which the V-shaped coiled-coil (CC) domain of each protomer acts as a pair of molecular tweezers locking the catalytic domain of each dimeric partner in an inactive configuration. It has been suggested that the release of the catalytic domains likely requires an ATP-driven conformational change in the FtsEX complex, and FtsX PCD (referred to as extracellular loop 1 or ECL1) has been shown to interact directly with the coiled coils of the PscB.^434^ The PCD of FtsX reveals an upper extended β-hairpin and a lower α-helical lobe, with each extending from a mixed α–β core. The helical lobe mediates a physical interaction with the peptidoglycan hydrolase PcsB via the coiled-coil domain of PcsB.

**Figure 21:**
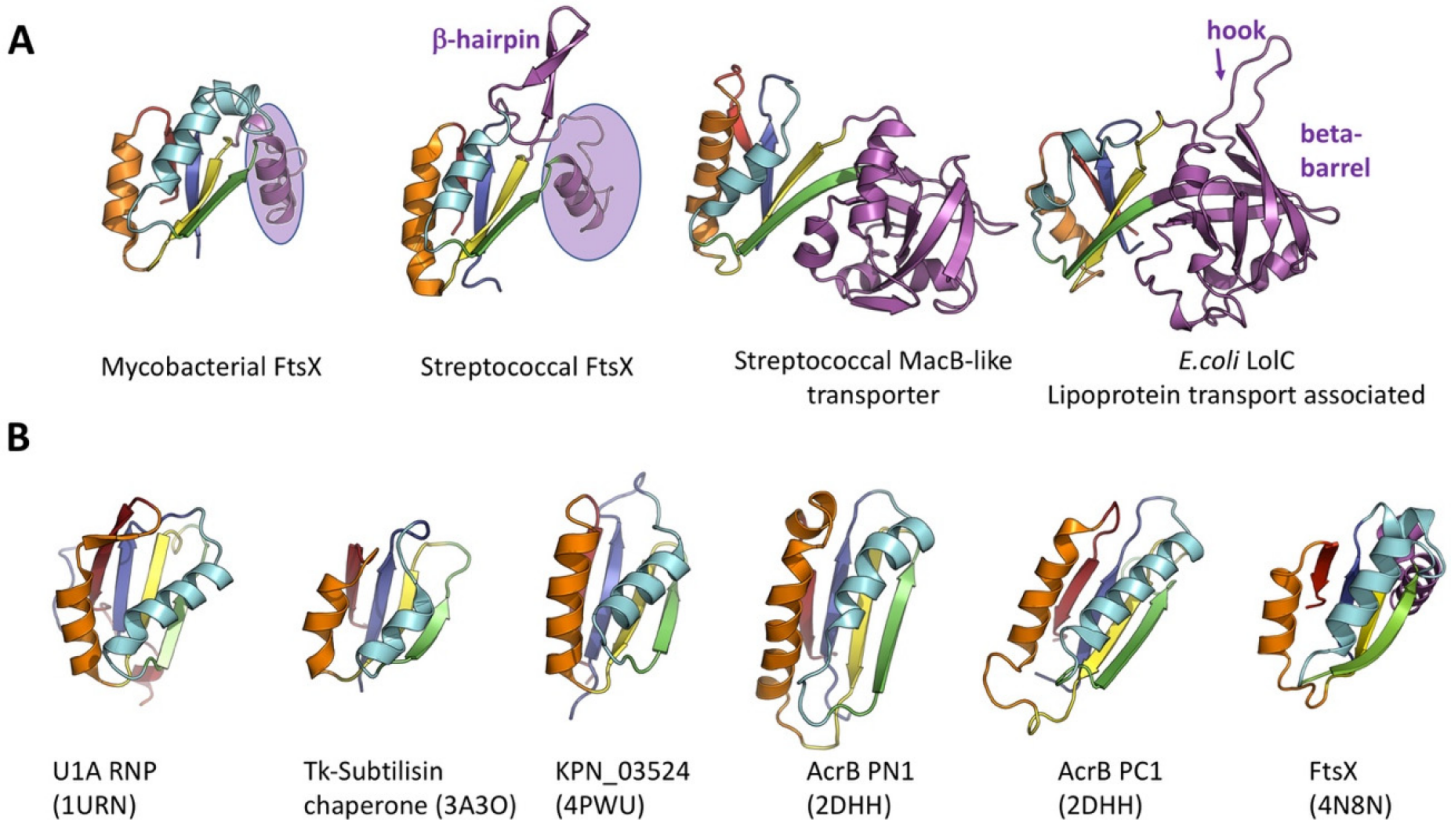
Diversity of PCD domains in the FtsX/MacB family and porter domain connections. (A) The basic PD-module of the PCD is conserved across the MacB/FtsX family; however, there are multiple variations on the theme. The architecture of the module is expandable, but intriguingly, it features conserved regions of insertion, which are shared not only between Gram-positive and Gram-negative bacteria but also with eukaryotic organisms. The PDB codes for presented structures are as follows: Mycobacterial FtsX (4N8N); SpFtsX (6HE6); Spr0695 (5XU1); LolC (5NNA); NPC1 (5U74); DISP (6XE6). (B) PD domains present in different proteins outside of the MacB-family (see text for more details).

Within the classical FtsX family transporters the mycobacterial homologues (e.g., PDB ID 4N8N)^433^ could be considered a basal group, and there the SABRE domain is presented by a simple helical extension, while the FtsX from *Streptococcus pneumoniae* (PDB ID 6HFX; 6HEE; 6HE6)^434^ presents an additional β-hairpin insertion between β1 and α1 (see Figure 21 for a comparison of SABRE domains). Intriguingly, exactly the same expansion of the basic PD-module can be found in the C-terminal domain (CTD), the PD1-domains of the eukaryotic RND-related cholesterol transporter NPC1 (e.g., PDB ID 5U74,^585,586^6W5R,^587^ and 6UOX(588)), as well as the second extracellular domain (ECD-II) of the Hedgehog-signaling associated Patched1 (PDB ID 6OEU)^589^ and the recently resolved Dispatched (DISP1) (PBD ID 6XE6)^590^ showing the topological versatility of the basic PD-fold.

The recent structure of the EnvC bound to the periplasmic domain of FtsX (PDB ID 6TPI)^591^ reveals an EnvC-FtsX complex with 1-to-2 stoichiometry with two distinct FtsX-binding sites located within an antiparallel coiled-coil domain of EnvC, showing that the FtsX-PD modules could be used not just to provide lipid- and PG-binding but can mediate protein–protein interactions.

In Gram-negative bacteria the inner-membrane-associated LolCDE ABC transporter, the LolA chaperone, and the LolB outer-membrane receptor form an essential system for membrane extraction and transportation of the newly produced lipoproteins from the outer leaflet of the plasma membrane to the inner leaflet of the outer membrane.^592^

The permease unit of the transporter is formed by the heterodimer of LolC and LolE, both of which have MacB/FtsX-like structure, and in *E. coli* the LolC-LolE heterodimer cooperates with the NBDs of the ATPase LolD, which provides energy for the lipoprotein extraction. A very similar system is present in *A. baumannii*, where a homodimeric LolF binds to a dimer of LolD.^593^ The structures of the PCD of LolC have been revealed, both in isolation (PDB ID 5NAA(431) and 6F49(432)) and bound to the periplasmic chaperone LolA (PDB ID 6F3Z)^432^ which reveals how a solvent-exposed β-hairpin loop (termed the “Hook”) and trio of surface residues (the “Pad”) of LolC are essential for recruiting LolA from the periplasm and priming it to receive lipoproteins (PDB ID 6F49).^432^

The PCD of LolC presents a departure from the prototypical MacB PCD fold, and its SABRE domain has a distinctive β-hairpin structure formed by residues P167–P179 (full-length LolC numbering), which has been named the “hook”, which is not observed in the MacB, and which, along with a trio of charged residues from the external side of the SABRE β-barrel, named the “Pad”, participates in the LolA recruitment^432^ (see Figure 21 above). A very similar arrangement is seen in the structure of the related A. *baumannii* LolF (PDB ID 5UDF) generated from a structural genomics effort

*Bacillus subtilis* and *B. amyloliquefaciens* produce sporulation-delaying protein (SDP), which is a peptide toxin capable of killing cells within a biofilm to support continued growth, thereby delaying the onset of biofilm sporulation. SDP producing cells protect themselves against the endogenous SDP via the action of the four-component transporter YknWXYZ which acts as a major SDP efflux pump.^568^ Within this assembly, the YknYZ forms a noncanonical ABC transporter, where YknZ is the permease, while YknY is the NDB. Heterodimeric assembly of YknY_2_Z_2_ forms a transporter remarkably similar to MacB,^579^ while the PAP YknX assembles into a hexameric pattern very similar to MacA.^594^ Despite the sequence identity of around 20%, the extracellular domain (ED) of the *Ba*YknZ reveals a structure which is virtually identical to that of the PCD of the *Aa*MacB with DaliLite server bringing a Z-score of 20.1 and structural superposition returning an RMSD of just 1.6 Å over the C-α backbone of the corresponding domain.^594^

Similarly, the structure of the related Gram-positive pump Spr0694–0695, where Spr0695 is the permease and Spr0694 provides the ATPase function, is surprisingly similar to the Gram-negative equivalent (PDB ID 5XU1)^571^ and presents PCDs which are virtually identical to those of MacB, suggesting communality of operation, despite the lack of OMF engagement in the respective complex. One small exception is the presence of a well-defined helical loop inserted in the SABRE domain (residues 202–216), which seems to form specific contacts with the TM helices of the transporter and could provide additional conformational coupling or, as suggested by the authors, plays the role of a gating loop.^571^

From the data available on the PCD domains, it is clear that they are predominantly engaged in peptide binding and protein–protein interactions, and furthermore, they display major conformational changes between different structures. It is a bit surprising that the PCD domains do not present high dimerization propensity on their own.^578^ However, in the available apo-*Ec*MacB and the nucleotide bound structures, as well as the PCDs of the ADP-bound structures of *Ab*MacB,^24^ they appear to undergo significant conformational rearrangements, which have been suggested to arise from the NBD-liked allostery.^442^

#### 5.7.6 Versatility of PD Fold outside of the MacB/FtsX Family. Modularity of Assembly, Phylogenetic Connections, and Topological Linkage between Different Transporter Groups

**Figure 22:**
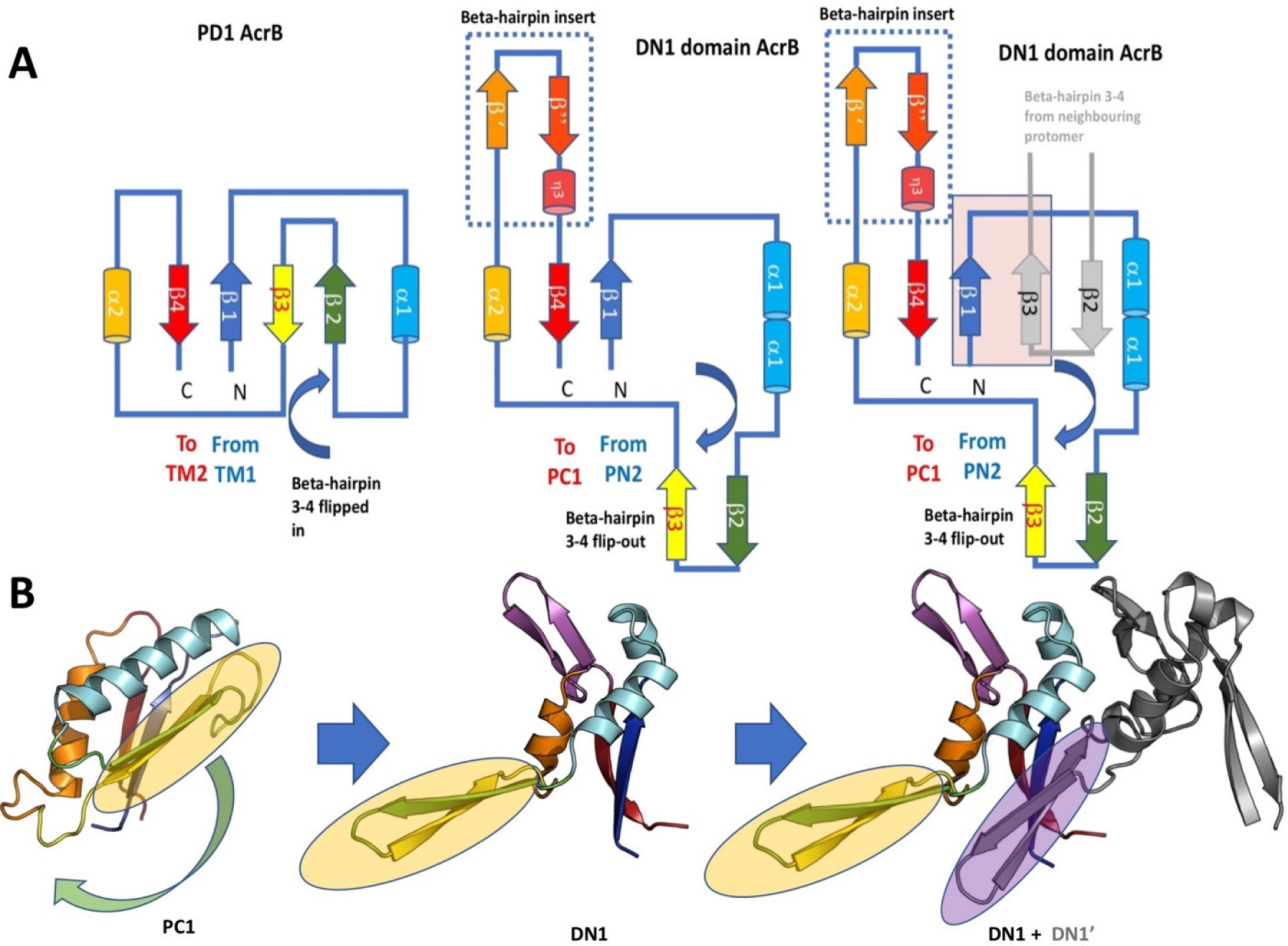
Topological connections of the basal RND PD-module and the docking/funnel subdomain in RND transporters. (A) The upper panel presents the topological diagrams, demonstrating the DN domain could be derived from the basal PD-module by flipping out the β2/β3 hairpin which forms part of the central β-sheet. This disrupted cental sheet is complemented by the corresponding β2/β3 hairpin of the neighboring subunit by β-augmentation. Note that the antiparallel character of the central sheet changes as a result. PD1 AcrB denotes the organization seen in PN1/PC1 subdomains. (B) The crystal structures of the corresponding modules on the example of the AcrB PC1 subdomain (PDB ID 2GIF) and the DN1 subdomain of CusA (PDB ID 2DHH).

The porter domains are the central building blocks of the periplasmic part of RND transporters. Numbering these for an isolated PD domain from the N- to the C-terminus, by the respective secondary structure elements, provides a β_1_–α_1_–β_2_–β_3_–α_1_–β_4_ configuration, which we refer to as the “standard PD” domain, which is observed in the AcrB PC1 domain^279^ (Figure 22).

The standard PD domain of RND transporters is a version of the ferredoxin-like fold,^595^ which is composed of 2 α-helices and 4 β-strands, in an α-β sandwich configuration, with the α-helices being spliced between the β1/β2 and β3/β4, respectively, and the 4 β-strands forming an antiparallel β-sheet. The fold has been initially described from the activation domains of pro-carboxypeptidases (e.g., PDB ID 1NSA(596) and 1AYE(597)) and a number of RNA-binding proteins, including small nuclear ribonuclear proteins, e.g. U1A small spliceosomal protein (PDB ID 1URN)^598^ and ribosomal protein S6 (PBD ID 1RIS),^599,600^ with the latter showing a β-roll transition of the central sheet.

The pore domains of prototypical HAE-1 type transporter such as AcrB,^279^ as well as the HME group transporters, such as CusA,^354^ contain four variants of the PD fold, namely the PN1, PN2 and PC1, PC2 domains (Figure 5 and Figure 6), with PN1 and PC1 being continuous folds, while PN2 and PC2 harbor insertions between their respective β2 and β3 strands, comprising the funnel (FN) domains.^279^ Furthermore, while PN2 and PC2 present a 4 stranded β-sheet, the PN1 and PC1 show an extended 5-stranded sheet provided by β-augmentation *in trans* from the Cβ13 and Nβ13 strands, respectively^279^ (Figure 5, Figure 6, and Figure 22).

Our analysis of the fold of the globular subdomains that form the funnel or docking domain of the RND transporters reveals that they could be derived from the “standard” PD domain topology by an additional β-hairpin insertion following α2-helix and a flip of the β-hairpin formed by the β2 and β3 strands of the central β-sheet, leading to its disruption. The central β-sheet is then supplemented during oligomerization, by the equivalent β2−β3 hairpin from the neighboring protomer, providing interconnectivity between the subunits of the functional trimer. As a result of this “hairpin swap”, the central continuous β-sheet of the “standard PD domain” is replaced, e.g., in the DN1 domain of AcrB, by a pseudocontinuous β-sheet, where, due to the outward flip of the β2−β3 hairpin, the antiparallel interface between the cognate β1- and β3-strands is substituted by a parallel β1- and β3′-strand arrangement (Figure 22).

**Figure 23:**
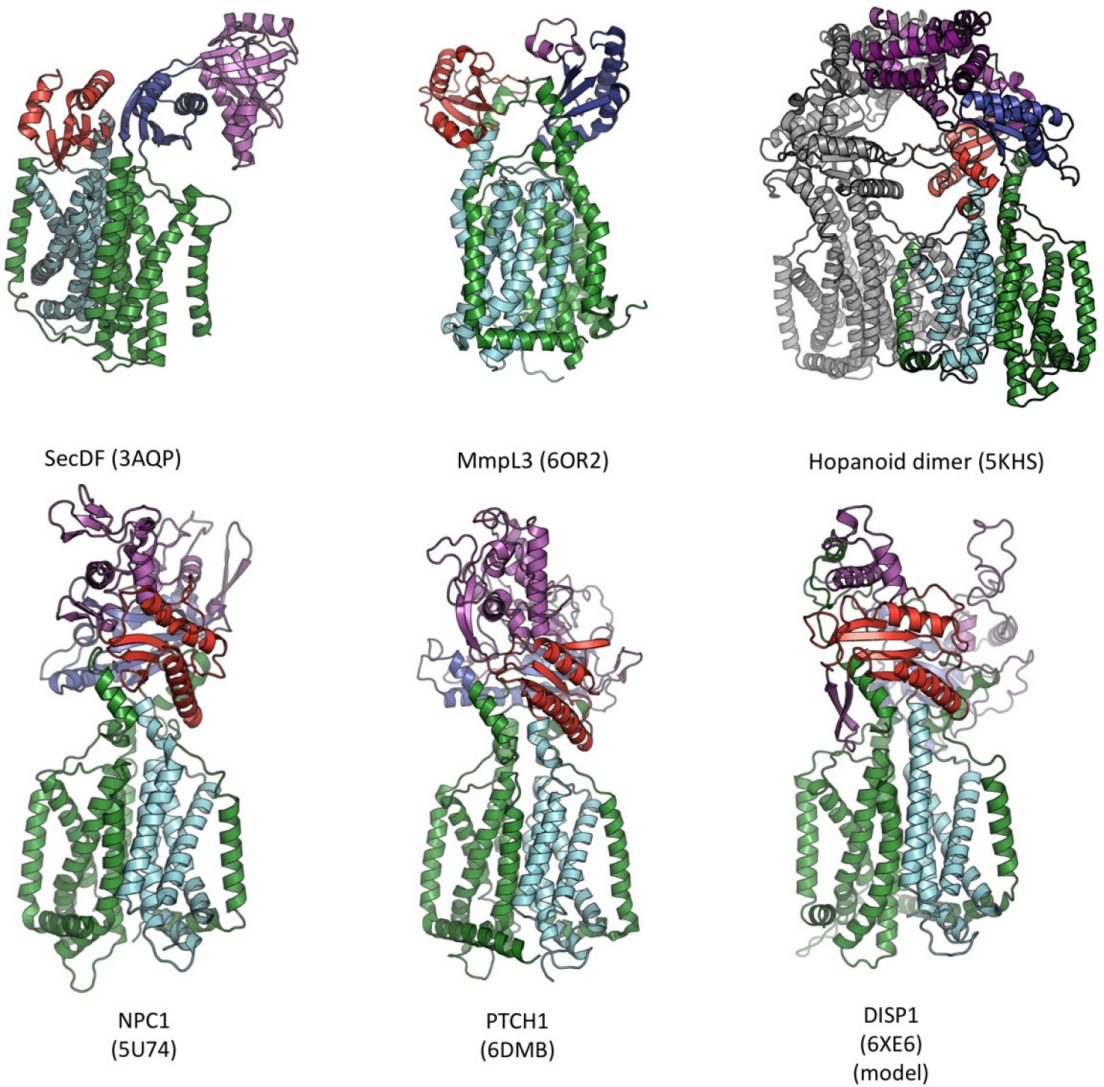
Different families of RND transporters and related proteins. The representatives of the SecDF family, as well as the prokaryotic MmpL and Hopanoid transporter groups are shown, as well as the known structures of eukaryotic RND transporters. The PDB ID codes are given in parentheses. Due to the poor resolution and disorder, the missing loops of the DISP1 have been modeled for completeness. See text for more details.

A number of RND and related transporters present variations on such an architecture including the HAE-2 family, with representatives such as MmpL3 (PDB ID 6AJF);^277^ HAE-3 HpnN (PDB ID 5KHN);^278^ and the more distantly related SecDF translocases (e.g., PDB ID 3AQP(364) and PBD ID 5YHF^366^), as well as representatives from eukaryotic groups, such as the human cholesterol transport associated Niemann–Pick disease type C protein 1 (NPC1) (PBD ID: 5JNX(601) and 6W5R(587)), mouse cholesterol translocator Patched (PBD ID 6MG8),^602^ and the human Patched 1 (PDB ID 6OEU(589) and 6DMB(603)) (Figure 23).

##### 5.7.6.1 MmpLs

**Figure 24:**
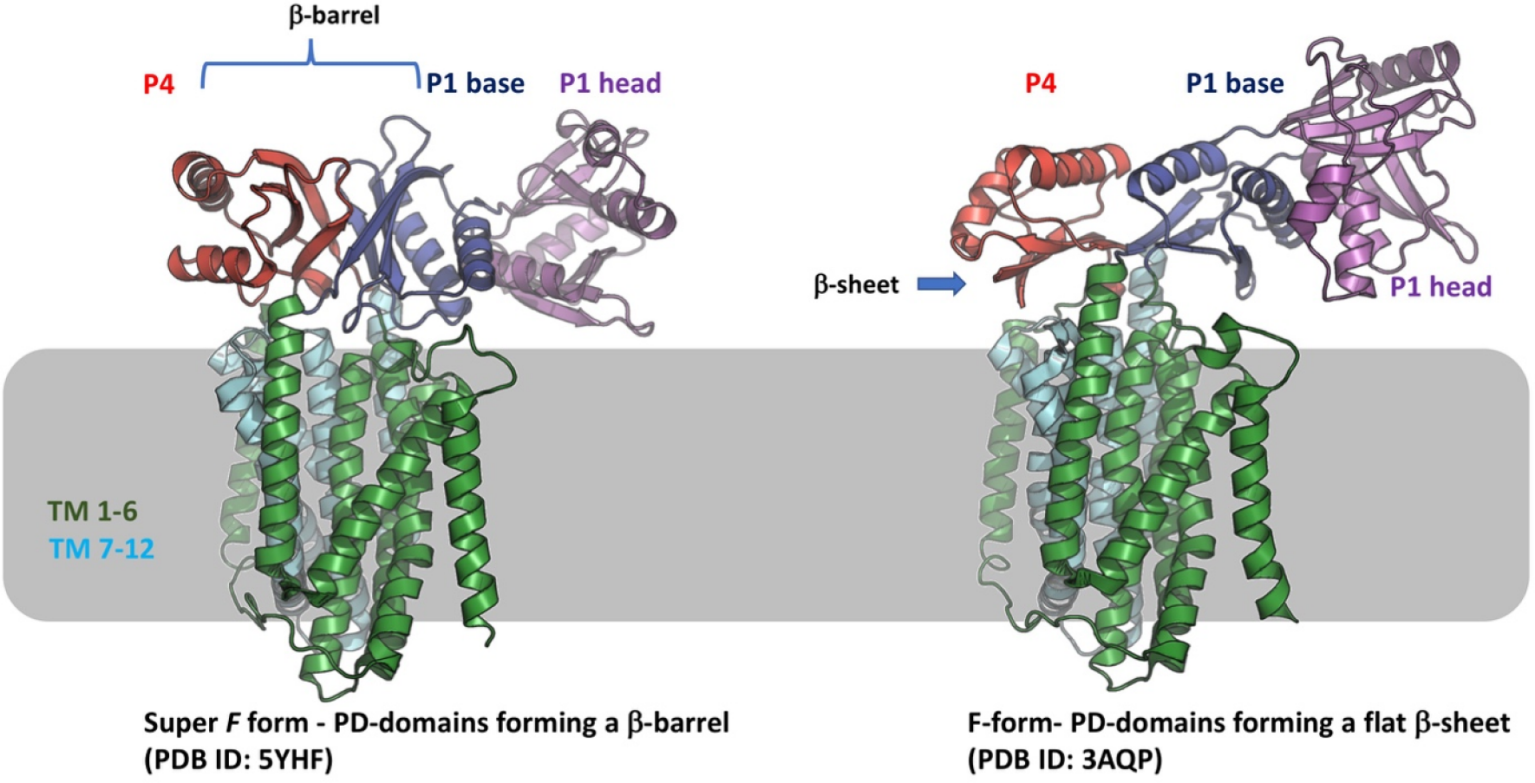
Structural transitions within the PD-domains of SecDF are linked to the functional cycle of the transporter. The PD modules in the periplasmic domains of SecDF (P4 and P1-base) undergo a conformational transition from β-barrel to β-sheet between the Super *F* form (as seen in the PDB ID YHF) and the F-form (PDB ID 3AQP). Modified based on Furukawa et al.^366^ These are linked to the substrate engagement by the P1-head domain of the transporter, and similar transitions may occur within other PD-containing transporters, e.g., the PD-domains of the MacB/FtsX family.

**Figure 25:**
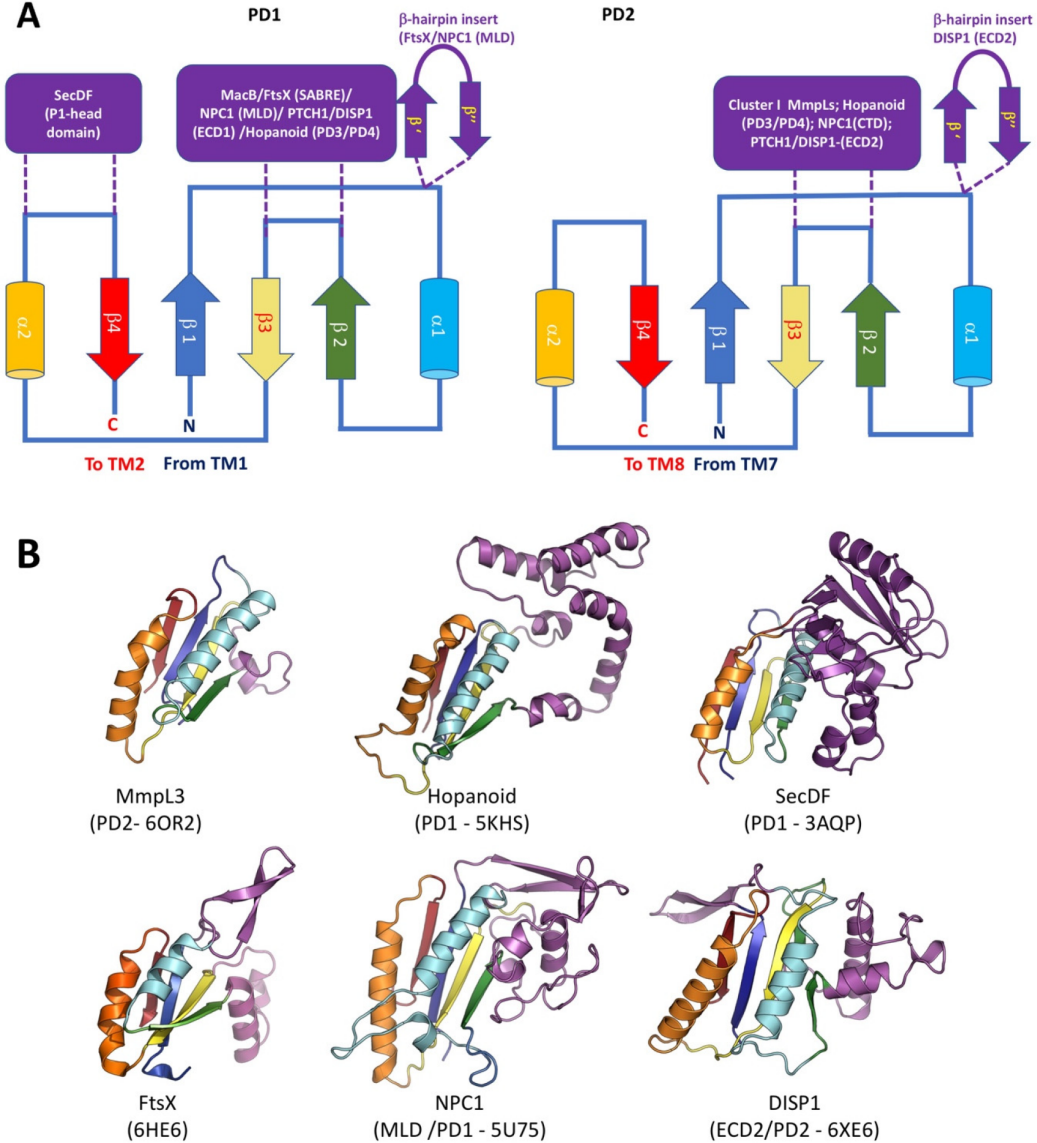
PD domains provide a flexible modular architecture which is shared across a number of different transporters. (A) Topological diagram showing the decoration of PD domains in different transporter classes. Where the transporters are not homooligomers with a single PD module per domain, the PD1 and PD2 domains of respective subunits are presented. The insertion points are limited and conserved across the known PD-domains, with notable splicing events taking place between β2 and β3 of the central β-sheet. Minor β-hairpin decorations are seen in the FtsX family and eukaryotic RND transporters. In addition, the SecDF family displays a unique insertion between α2 and β4. (B) Gallery of the PD-domains of the notable members of the transporter families discussed above. The domain abbreviation and PDB ID code of the respective structures are provided in parentheses. Abbreviations: MLD, middle luminal domain; ECD1/2, extracellular domain 1/2; CTD, C-terminal luminal domain or Cys-rich domain.

In *Mycobacterium tuberculosis* the 14 members of the MmpL (mycobacterial membrane protein large) transporter family form a group of proton-driven carriers, that translocate complex (glyco)lipids and siderophores across the cell envelope.^604,605^ Unlike the RND transporters of the HAE-1 and HME families, these transporters operate as dimers, although some earlier data on the orthologous Corynebacterial CmpLs,^606^ namely CmpL1, has suggested a possibility of a trimeric assembly.^605^ As these transporters are subject to a detailed review in the current issue of *Chemical Reviews*,^607^ we will give only a cursory note about them here. Phylogenetic analysis has suggested that the group shares high homology to the classic HAE/HME RND family and similarly presents an internal gene duplication with resulting 12 TM-helices,^608^ with extracellular porter-domains D1 and D2 homologous to the PD-domains being spliced between the TM1/TM2 and TM7/TM8, respectively^609^ (see Figures 23 and 24 and Figure 25A). Two broad clusters of MmpLs can be derived based on the topology of their PD domains, a more basal group, called cluster II, including MmpL 3, 11, and 13, which possess two standard PD domains (with a β_1_–α_1_–β_2_–β_3_–α_1_–β_4_ configuration), “cluster I” which includes the rest of the family that have a standard N-terminal PD domain, and an expanded C-terminal D2 domain featuring an insertion between the β_2_–β_3_ (β_1_–α_1_–β_2_–X−β_3_–α_1_–β_4_),^609^ thus presenting a PD-architecture equivalent to that observed in the MacB/FtsX family discussed above. Consistent with the prediction, the structure of the isolated D2 domain from the MmpL11 (PDB ID 4Y0L)^609^ showed high homology to the PD domains of classical RND transporters. Cross-linking experiments suggest that the orientation of the PD domains in MmpL 11^609^ is similar to the PC1/PN2 domain orientation on the AcrB, e.g. (PDB ID 2GIF)^318^ or CusA (PDB ID: 4DNT).^610^ However, later structures of the MmpL3 from *M. smegmatis* (PDB IDs 6AJF-6AJJ,^277^6OR2^611^) display a distinct domelike arrangement. In addition, the PD2 domain of MmpL3 (PDB ID 6OR2) does appear to have a small insert, after β2 (see Figure 25B). At present there are no experimental structures of PD domains belonging to cluster I MmpLs, although our bioinformatic analysis suggests their insertions are not related to the ones observed in the funnel domains of RND transporters and may be closer related to the α-helical domains observed in the HAE-3.

##### 5.7.6.2 Hopanoid Transporters

The hopanoid transporters, which are representatives of the HAE-3 group and are responsible for the transport of the eponymous pentacyclic triterpenoids with a sterol-like function in the bacterial membrane,^278^ also exhibit high similarity to the RND transporters, consistent with that present for PD-like domains. The crystal structure of the HpnN (PDB ID 5KHN)^278^ (see Figure 23) revealed a dimeric assembly of the transporter, which presented four PD domains, with PD1 and PD2 being equivalent to the classic PD domains observed in HAE-1 and HME RND transporters, but in addition, they present helical inserts forming additional PD3/PD4 domains, with no relation to any hitherto known domains. The splicing of the PD3/PD4 into the PD1 and PD2, respectively, produces the same topology (β_1_–α_1_–β_2_–X−β_3_–α_1_–β_4_) as discussed above for MmpL and the MacB/FtsX proteins suggesting a general pattern for expanding the architecture (see Figure 25, panel B).

##### 5.7.6.3 SecDF

Working in conjunction with the major secretory complex SecYEG/SecA^612^ that is driven by SecA ATP-hydrolysis, the proton motive force powered SecDF complex facilitates the threading of the unfolded protein cargoes and plays a chaperone-like function.^365,613,614^ In *Thermus thermophilus* SecDF, the SecD and SecF are fused into a single polypeptide chain, and its structure (PDB ID 3AQP)^364^ revealed that similar to the group I MmpLs discussed above, SecDF presents a pseudosymmetrical, 12-helix transmembrane domain belonging to the RND superfamily and two homologous major periplasmic domains, P1 and P4 (Figure 24 and Figure 25). While the P4 domain shows a classic β_1_–α_1_–β_2_–β_3_–α_1_–β_4_ organization, similar to the PD-domains in HAE-1 PD and MmpL3, the P1 domain presents a β_1_–α_1_–β_2_–X−β_3_–α_1_–β_4_ organization, where “X” is a large insert forming the so-called “head” subdomain, while the core PD β-strands form the cores of both the P1 and P4 form a pseudosymmetrical 8-stranded antiparallel β-sheet that covers the TM region of SecDF and is planar in the most structures, including P1-membrane facing “*F*” forms and P1-outward facing “*I”*-forms;^364,615^ however, unexpectedly, the PD-domains of the transporter were also revealed in a β-barrel form (known as Super *F*-form) (PDB ID 5YHF).^366^ The β-barrelled super *F-*form is speculated to engage with the emergent preprotein coming out of the SecYEG on the periplasmic side of the membrane, which is bound to the P1-head. The conformational transition from super *F*-to-F and planification of the β-sheet is speculated to be associated with substrate dragging, and consistent with such interpretation, fixing the β-sheet by cross-linking between the interface of the P1-base and P4 abolished the translocation activity of SecDF, suggesting that β-sheet rearrangement occurs during protein translocation.^615^ Further transition of the *F* to *I*-form is associated with the formation of a proton-accessible tunnel in the TM-part of the protein (as seen in PDB ID 5XAN) and subsequent release of the cargo, leaving the P1-head outward facing in the cargo-disengaged “*I* without tunnel form”^366^ (Figure 24).

In a possible analogy to the efflux RND transporter cycle, the substrate binding causes conformational transitions, that allow the access and binding of the protons to the TM-domains, with actual release of the protons on the cytoplasmic side being associated with the cargo release and thus, recycling of the complex. Despite the similarities, the proton relay network in SecDF does not appear to function the same way as in canonical RND transporters such as AcrB. Notably, while there is still a tight interplay between the residues from TM4 (esAspIV; “es”—standing for “essential”) and TM10 (Tyr), unlike the AcrB, the proton-relay is supported by critical residues located on TM11 (esArgXI).^366^

##### 5.7.6.4 PD-Domains within the Eukaryotic RND Transporters

Members of the eukaryotic sterol transporter family include the Niemann–Pick type C protein 1 (NPC1) and the Niemann–Pick type C protein 1 like 1 (NPC1L1). The first is a lysosomal membrane protein essential for cholesterol homeostasis. Defects of NPC1 are associated with hereditary Niemann–Pick disease leading to a fatal accumulation of low-density lipoprotein (LDL)-derived cholesterol.^616^ Active as a monomer with 13 TM helices and an extended N-terminal domain, NPC1 presumably acts as a receptor/transporter for the cholesterol-loaded NPC2 soluble carrier via the N-terminal soluble loop.^274,586^ It is also the target of the Ebola virus acting as an intracellular receptor. Structures for the full-length NPC1 and in complex with the cleaved glycoprotein of the Ebola virus have been published (PBD ID: 5JNX,^601^6W5R^587^). NPC1L1 is an intestinal membrane protein involved in the absorption of cholesterol and a target of the therapeutic compound ezetimibe, for which recently cryo-EM structures have been solved.^617^ Patched (PTCH1) and Dispatched (DISP) belong to the eukaryotic dispatched RND transporter family. Both are involved in the hedgehog signaling pathway, during embryogenesis and tissue homeostasis, where Dispatched exports the N-terminal portion of sonic hedgehog, with C-terminally linked cholesterol,^274,618,619^ and PTCH1 acts as a receptor of the lipidated sonic hedgehog protein, binding of which releases the downstream suppression of Smoothened (SMO). Cryo-EM structures of monomeric human PTCH1 resemble the architecture of NPC1 (PDB ID 6OEU, 6OEV;^589^6DMB^603^), presenting an RMSD of ∼2.5 Å, and recently the structure of the human Dispatched-1 (DISP1) homologue has been obtained (PDB ID 6XE6.^590^ These eukaryotic homologues possess two separate PD domains, which are decorated in a similar fashion to the ones discussed above for the Cluster I MmpLs and Hopanoid transporters. Notably, the two PD-like domains of NPC1, named the middle luminal domain (MLD) and C-terminal luminal domain or Cys-rich domain (CTD), present large insertions between the β_2_–β_3_ (PDB ID 5U74),^586^ an organization which is also shared by the Patched structure.^589,603^ In addition, the NPC1 MLD-domain (PD1) contains a β-hairpin insertion between β1 and α1, which mimics the ones observed in the FtsX members such as discussed previously (Figure 25 and Figure 21). A very similar insertion is seen in the extracellular domain 2 (ECD2/PD2) of the DISP1 (PDB ID 6XE6)^590^ but not in the PTCH1.

Taken together, the analysis of the PD domains of the RND-like transporters presented, as well as the FtsX/MacB family, displays a clear pattern of usage and modification of the PD modules, with two “hotspots” of insertion being utilized—the first one is located at the tip of the β_2_-β_3_ hairpin of the central PD-β sheet and allows for splicing of large subdomains, e.g., the SABRE domain in the case of the FtsX/MacB family, or the large helical subdomain inserts in the case of PD3/PD4 domains of the hopanoid transporters and the eukaryotic RND-like transporters (NPC1/DISP1/PTCH1). The second insertion point accommodates smaller splice-ins in the form of an additional β-hairpin insertion, as seen in the *S. pneumoniae* FtsX (PDB ID 6HE6); the ECD2 of DISP (PDB ID 6XE6), and the MLD domain of NPC1. Finally, the SecDF presents a unique insertion in its first PD-domain, with the P1-head domain being spliced after α2 (see Figure 25).

Notably the architecture seen in the PD domains is also present in a number of additional periplasmic proteins which lack transporter function, e.g., the two-component signaling modulator family of MzrA proteins (PDB ID 4PWU),^620^ as well as more distantly related proteins including the archaeal Tk-subtilisin chaperone pro-peptides (PDB ID 3A3O) and,^621^ similarly, chaperoning activation domains of carboxypeptidases (PDB ID 1PBA),^622^ as well as acylphosphatases (e.g., mtAcP PDB ID 1APS)^623^ and the aforementioned small nuclear ribonuclear proteins such as U1-SNRP A (PBD ID 3G8T,^624^3UCU^625^) (see Figure 21B).

#### 5.7.7 Relations of the Membrane Porter Domains of MacB/FtsX to Other ABC Transporter Families

**Figure 26:**
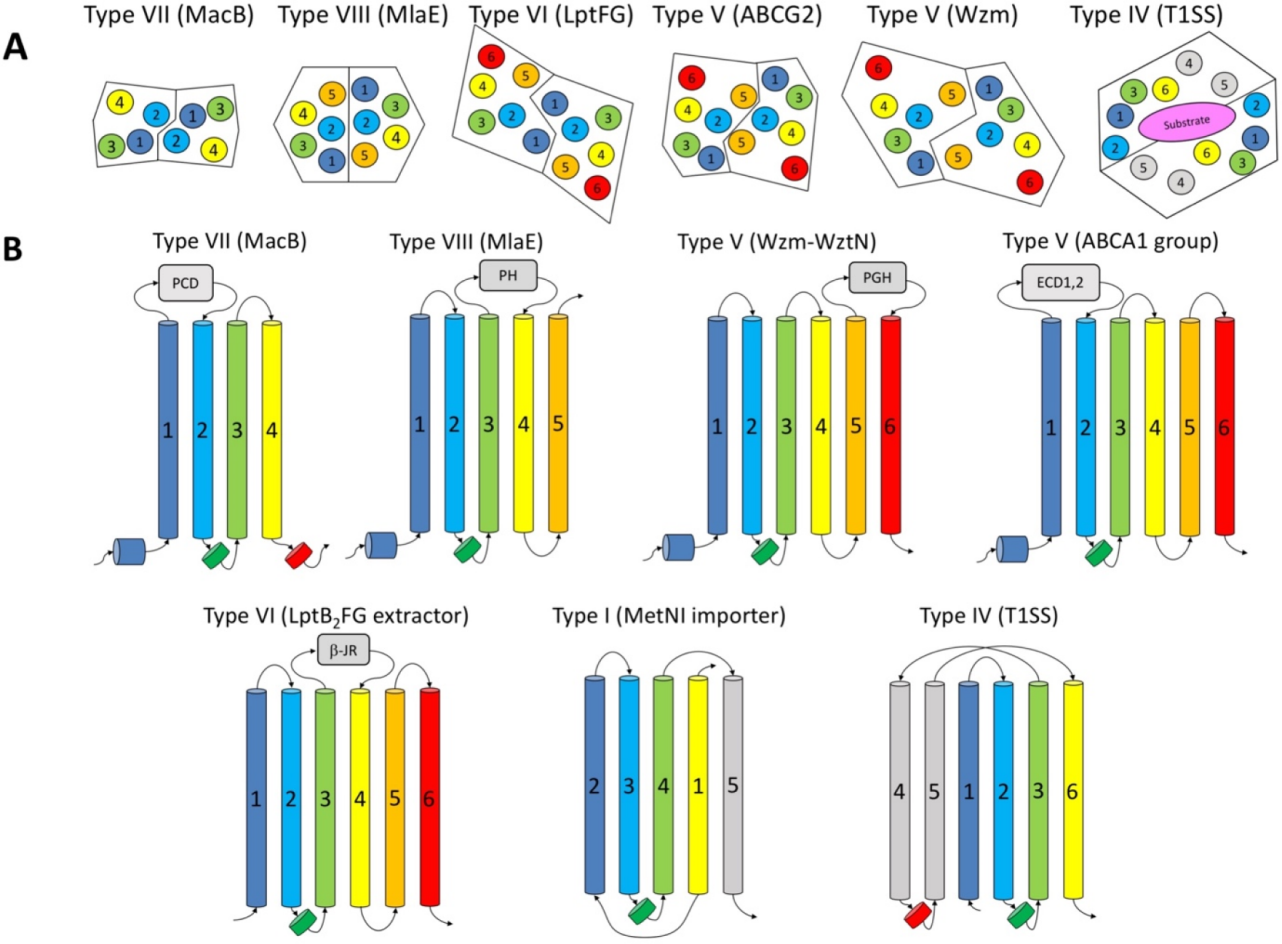
(A) Schematic representation of the TM helix packing at the dimer interface between different classes of ABC transporters shows close connections of the MacB architecture to the type VI and type VIII (MlaEF) transporters. Topologies are derived from the structures of MacB (PDB ID 5GKO),^24^ MlaE (PDB ID 6XBD),^439^ LptB2FGC complex (PDB ID 6S8N),^635^ Wzm-WznT (PDB ID 6M96),^429^ and ABCG2 (PDB ID 5NJG).^632^ Top-down view from the periplasm. Note: due to the intertwisting of the helices in the type IV transporters, the diagram depends on the level at which the slice is cut through the TM; the provided view is representative to the middle of TM segment, based on the ABCB1 structure (PDB ID 6QEX).^521^ (B) Topological connections within the ABC transporter families. The ABC transporter families share a common core architecture, which can be derived from the four TM helices seen in the type VII (MacB/FtsX) family. The other main families of exporters are presented, which could be seen as derived from MacB by addition of C-terminal helical fragments. Homologous helices are colored consistently from the N-terminal to the C-terminal. The intracellular coupling helices (CH1 and CH2) are presented in green and red. CoH = connecting helix. Substrate binding domains are shown as gray boxes (PCD, periplasmic core domain; PH, pore helix; PGH, periplasmic gate helix; ECD1,2, extracellular domains 1, 2; β-JR, β jelly roll domains). Adopted with modifications based on Schaeffer et al.^634^

At first sight, there appears to be limited similarity between the porter domains of MacB/FtsX containing 4-TMs and the more typical ABC transporters, which contain 6-TMs as standard. However, closer inspection of the MacB-TM regions utilizing DALI pairwise fold-comparison software^24^ and comparison against different members of the type IV family, such as ABCB1 (PDB ID 4M1M,^626^3G60^627^), heterodimeric TmrAB (PDB ID 5MKK), and the ADP-bound form of the lipid-linked oligosaccharide flippase PglK (PDB ID: 5NBD),^628^ reveal unexpectedly high similarity of the MacB/FtsX family to this family, with the ABCB1 returning a Z-score of 8.7 (PDB ID 3G60), based on the superposition of the first three N-terminal helices (TM 1–3) of the type IV transporters showing high structural similarity to the MacB TM helices 1–3. Despite this similarity, it has to be noted that the dimer interface of MacB is not matched with the dimer interface presented by the structures of type IV ABC transporters, e.g. in the MacB/FtsX TM helices 1–3 do not form part of the substrate binding cavity and are hence more likely to play a role in energy transduction, rather than substrate interactions, while in type IV helices 1–3 face away from the central cavity toward the lipid bilayer^24^ (Figure 26, panel A, showing the packing in ABCB1 6QEX^521^).

An expanded topological analysis of the type VII ABC-family topological relationships, which in addition to the type IV transporters discussed above also included type I importers (e.g., MetNI(Q) (PDB ID 3DHW),^629^ YjgP/YjgQ (LptBFG; type VI family)),^630,631^ as well as the members of type V exporters, such as the ABCG^582,632^ and ABCA^633^ involved in eukaryotic lipid export, revealed unexpected similarities, suggesting that they might be evolutionarily related.^634^ Based on these similarities, an alternative classification of ABC transporter topologies has been proposed, with the core of these transporters’ structures consisting of the four-TM core segments (1–4), as observed in the MacB/FtsX family (e.g., PDB ID 5WS4), and it has been proposed that MacB/FtsX (Group VII) may be a basal group of transporters from which the other topologies covering groups V, VI, and even IV are derived. Uniquely, the MacB/FtsX family additionally possesses a second coupling helix (CH2) at the C-terminal end of the fourth core TM segment (Figure 26, panel B; TM-segment is colored red), which is not present in the TMDs of LptBFG (YjgP/YjgQ; type VI) or in group V (ABCG and ABCA) transporters.^634^

Intriguingly, the complete topology of type IV floppase/exporters (formerly type I exporters) could also be derived when using MacB/FtsX as a basis, as TM segments 1, 2, 3, and 6 can be structurally aligned to the TMs 1–4 of MacB. Insertion of TM helices 4–5 carrying a canonical coupling helix between TM3 and 4 of MacB/FtsX produces a topology equivalent to the type IV floppase/exporters. Consistent with this, DALI superposition of MacB (PDB ID 5WS4) with type IV heterodimeric exporter TmrAB (PDB ID 5MKK),^523^ which is a homologue of the antigen translocation complex TAP, returned a Z-score of 7.7.^24^ While intriguing and appealing in its simplicity, such systematics do not automatically suggest that the MacB/FtsX family is a basal group, as it could be a product of degenerative evolution.

More recently, Murakami et al.^580^ also extended their DALI analysis to the type V family members ABCG2 (PDB ID 5NJG)^632^ and ABCA1 (PDB ID 5XJY)^633^ that returned high Z-score values of 7.6 and 7.5, respectively. While the overall score is lower, these transporters allow better superposition, covering all four TMs of MacB, and importantly, they provide a dimer interface matching that of the MacB.

Our initial analysis of MacB-structures has also suggested a connection of the MacB/FtsX with type V floppases/exporters such as Wzm-WztN and the ABCG/ABCA family but also with the yet-to-be classified Mla-system members due to the overall topology of TMDs, the presence of the amphipathic connecting helices (CoH), linking the NBDs to the TM helices, and the close association of the NBDs with the membrane. In Figure 26 (panel B), we have expanded the topological classification discussed above,^634^ incorporating the data from the recently solved structures of the yet-to-be classified MlaEF-ABC transporter system,^437−439^ the structure of the permease subunit of which, MlaE displays a number features linking it with the MacB and LptB_2_FG families, plus a set of unique elements, that in our view justify classifying it as a separate group of ABC transporters, which we tentatively assign to type VIII for clarity of presentation below. Notably, the MlaE topology can be readily derived from a MacB/FtsX family basal topology by addition of C-terminal helix 5 (Figure 26, panel B). Furthermore, the TM5-helices of MlaE pack analogously to the similarly derived helix 5 in the LptFG complex, and crucially, the packing is dominated by close bundling of TM2 which is only observed in the MacB family, setting these two groups into a close sister grouping within the ABC-superfamily, reaching similar conclusions from the independent topological analysis by Coudray et al.^439^ Helical packing diagrams (Figure 26, panel A) provide further evidence of common patterns across the ABC-groups V–VII.

#### 5.7.8 TMD and Energy Coupling

As mentioned earlier, the analysis of the topologies of the available ABC transporter structures revealed that there are at least seven structurally distinct ABC transporter folds,^428^ of which MacB is the most-recently determined representative. MacB can therefore be considered the “holotype” for the whole of the type VII ABC transporter superfamily^428,431^ and has become the first structurally characterized member of the ABC3 superfamily of the ABC transporters as defined by Wang et al.^427^ Its unique architecture suggests a mechanistically distinct functional cycling mechanism.

The *Ec*MacB has been revealed in a nucleotide free form (PDB ID 5NIK), with the NBDs apart and unable to bind ATP.^272^ However, an ATP-bound (PDB ID 5LJ7) and nonhydrolyzable ATP-analogue bound form (PDB ID 5LJ6, 5LOL)^431^ have also been reported. For *Aa*MacB and *Ab*MacB, an ADP-analogue bound form (e.g., adenosine-5′-(β-thio)-diphosphate (PDB ID 5WS4)) and a nucleotide-free form (PDB ID 5GKO) have been obtained, respectively.^24^ In addition, the structure of the Streptococcal MacB-like transporter Spr0694–0695 (PDB ID 5XU1)^571^ was revealed in a nucleotide-free state and the comparative analysis of these structures has allowed us to reconstruct a plausible energy-coupling cycle of the MacB/FtsX-like transporters.

**Figure 27:**
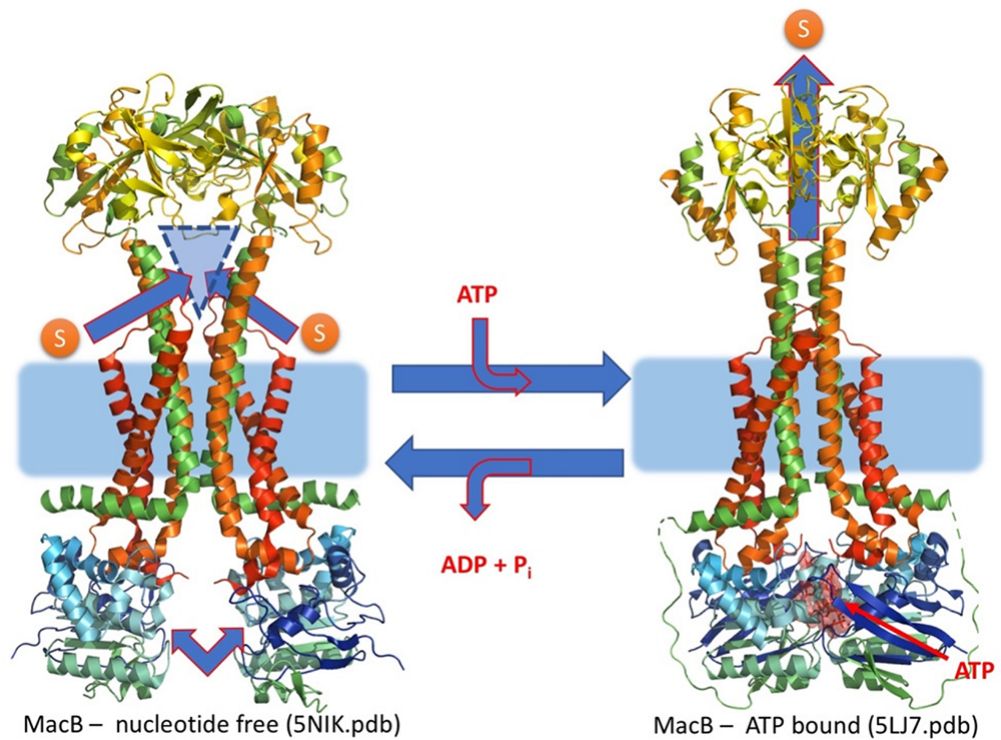
Mechanotransmission mechanism of MacB. Crystal structures suggest a possible cycling mechanism for MacB. In the nucleotide-free structures, the NDBs of the transporter are separated and appear unable to bind ATP. This coincides with the separation of the PCDs and lack of helical-bundle formation in the stalk helices. To the contrary, the ATP-bound forms show significant rearrangement of the PCDs, which also coincides with a helical bundle formation in the TM-domain, suggesting that the ATP-binding is communicated long-range from the NBDs across the membrane to the periplasmic domain through the TM-stalk helices. This has been dubbed “mechanotransmission”, as the stroke from ATP-binding is not used to transport the substrate across the membrane in which the ABC transporter resides but rather to cause conformational changes in the periplasm. (Modified, based on the model by Crow et al.^431^)

In none of the structures does the tightly packed dimer interface of MacB present any credible space for a transmembrane cargo to be accommodated. However, the nucleotide-free, as well as the ADP-bound *A. baumannii* structures clearly resemble each other with the extra-membrane helical protrusions of the TM1 and TM2 being bent away from the central axis of symmetry, creating a “Y-shaped” crevice, which appears accessible to potential periplasmic cargoes.^24,272^ The PCDs in the nucleotide-free forms and in the ADP-bound form appear to be separated. In the presence of ATP, the NBDs are brought together, which causes the major coupling helix CH1 to push up the TM2, causing contraction of the interhelical gap, which results in a transformation of the loose helical arrangement into a rigid four-helical bundle,^431^ and this “zipping-up” of the interhelical interface causes a marked contraction of the PCDs, which are squeezed together. Such long-range communication from the NBDs through the TM-stalk helices all the way to the periplasmic domain has been dubbed “mechanotransmission”, as the stroke from the ATP-binding is not used to transport the substrate across the membrane in which the ABC transporter resides but to cause conformational changes into the periplasm, or extracellular space, respectively. Consistent with such an interpretation, disulfide cross-linking of the stalk-helices has been demonstrated to severely impact MacB function *in vivo*(431) (see Figure 27).

Thus, in MacB, the tight coupling between the TMD and the NBDs via CH1 and CH2 is likely used not solely for conformational changes of TM helices but is also translated into long-range communication with the PCDs, which is in turn converted into substrate transport via the PAP- and OMF-interactions.

Under the currently prevailing paradigm, ATP-binding and hydrolysis on the inside of the membrane cause large, transmembrane conformational changes in the MacB structure, leading to “zipping-up” of the central stalk-helices, and this has been suggested to drive the PCD-domain rearrangement which leads to cargo expulsion, although the exact binding sites and extrusion mechanism are not fully clear. It is clear, however, that rather than transporting substrates across the inner membrane, MacB-family transporters remove their substrates from the periplasm, utilizing long-distance conformational coupling of the NBD dimerization, in a mechanism which was dubbed “mechanotransmission”. Members of the MacB/FtsX family participating in tripartite complex formation may use the mechanotransmissive conformational coupling of the stalk helices to the PCDs to efflux substrates across the outer membrane via OMFs ducts, while the MacB/FtsX homologues that function alone can utilize such motions for exporting across the peptidoglycan layer in the Gram-positive organisms, as well as for a related function in lipoprotein trafficking and transmembrane signaling.

#### 5.7.9 Substrate Binding Site

In the MacB/FtsX family, the extra-membrane extensions of TM1 and TM2 protrude out of the membrane forming a kinked, “Y-shaped” conformation with a small semioccluded cavity, that is accessible from the periplasm. In the cryo-EM structure of MacB (PDB ID 5NIL), an unidentified electron density, occluding this region between the periplasmic extensions of TM1 and TM2, has been reported.^272^ Although, the low resolution of the associated cryo-EM map (at 5.3 Å) did not permit a clear interpretation, it has been speculated that it might represent a putative substrate binding site.^272^ While there is no direct structural data on substrate binding, structure-guided mutagenesis was used to narrow down the potential substrate binding residues,^431^ showing a plausible erythromycin binding site, covering predominantly bulky hydrophobic residues (including Y315, F320, F320, F503, and M507), mapping mostly to the PD-subdomain facing the interdomain interface. Their impact on the activity of the pump was also validated using bacitracin and colistin. Given their distribution across the PCD, it appears plausible that the PD subdomain is primarily engaged with cargo processing, while the SABRE domain, which shows significant conformational transitions and has been implicated in protein–protein interactions, is central to coupling to the PAP and the transmission of the conformational changes outward to the OMF, although future research is still needed to validate this hypothesis.

As mentioned above, the structure of the related Gram-positive pump Spr0694-0695, despite overall similarity with MacB, reveals a departure from its blueprint in the TMD portion of the protein.^571^ Specifically, there is divergence in the SABRE domain, where there is a short extra helical insertion (helix 6 of the PCD or rather in that case the extra-cellular domain or ECD) with the extra-membrane protrusions of the TM helices 1–3. Several charged residues are seen on this helix (e.g., K207-R208, K210), and it has been suggested that these may form a “guard helix” protecting a putative substrate-entrance tunnel. Mutation of the charged residues of the helix reduced the ATPase activity, suggesting it may also play a role in energy coupling.

In *Lactococcus lactis, the hrtB* gene (UniProt A0A2N5WEC6) codes for the permease, and the *hrtA* gene (A0A0H1RJ19) codes for the NBDs of a MacB-like heme transporter, which was initially suggested to remove it from the cytoplasm or the membrane-solubilized pool,^636,637^ although the exact mechanism remains unclear. Newer work suggests that the heme may instead be removed from the periplasmic side of the membrane in *S. aureus*(638) and consistent with that mutation of residues in the HrtB of *L. lactis* mapping to the SABRE domain of the PCD (Y168) and the upper part of stalk-helix 2 (Y231), speculated to coordinate heme-ligands, impacted function,^639^ suggesting this region may be involved in ligand binding. Despite this circumstantial evidence, the detailed characterization of the binding sites of MacB-like transporters must await another day.

In summary, the unique structural organization of the MacB/FtsX family suggests a distinct mode of operation for the binary PAP transporter pairs and for the tripartite assemblies in which they participate. We will discuss that in the context of the structure of the complete tripartite complexes with their participation below.

## 6 Outer Membrane Factors (OMFs): TolC and Others

### 6.1 Introduction to the OMFs

Ubiquitous in Gram-negative bacteria, proteins from the TolC family were initially characterized based upon their pleiotropic roles: their ability to induce tolerance to colicin E1,^196^ a role of the surface receptor for bacteriophages,^197^ resistance to antibacterial agents,^640^ or secretion of virulence factors.^61^ Several structural descriptions of TolC^641^ and its homologues have emerged over the last 20 years, notably OprM,^269,642,643^ OprN, and OprJ from *P. aeruginosa*,^644,645^ MtrE from *Neisseria gonorrhoeae*,^297^ VceC from *Vibrio cholerae*,^646^ CmeC from *Campylobacter jejuni*,^647^ and CusC from *E. coli*.^648^ These structures build upon an important number of (rational or random) side-directed mutagenesis, biochemical and biophysical studies that have shed light on the way OMFs open and assemble with their partners. The purpose of the following paragraphs is to try to confront structural and functional data obtained over the years and highlight the breakthroughs and paradoxes that have emerged.

### 6.2 Overall Organization of OMFs

OMF structures share a very similar architecture although their percentage of sequence identity is relatively low. OMFs are trimeric proteins, which form a long tubular channel one end of which is inserted in the outer membrane with the other end extending into the periplasmic space and interacting with the peptidoglycan layer.

**Figure 28:**
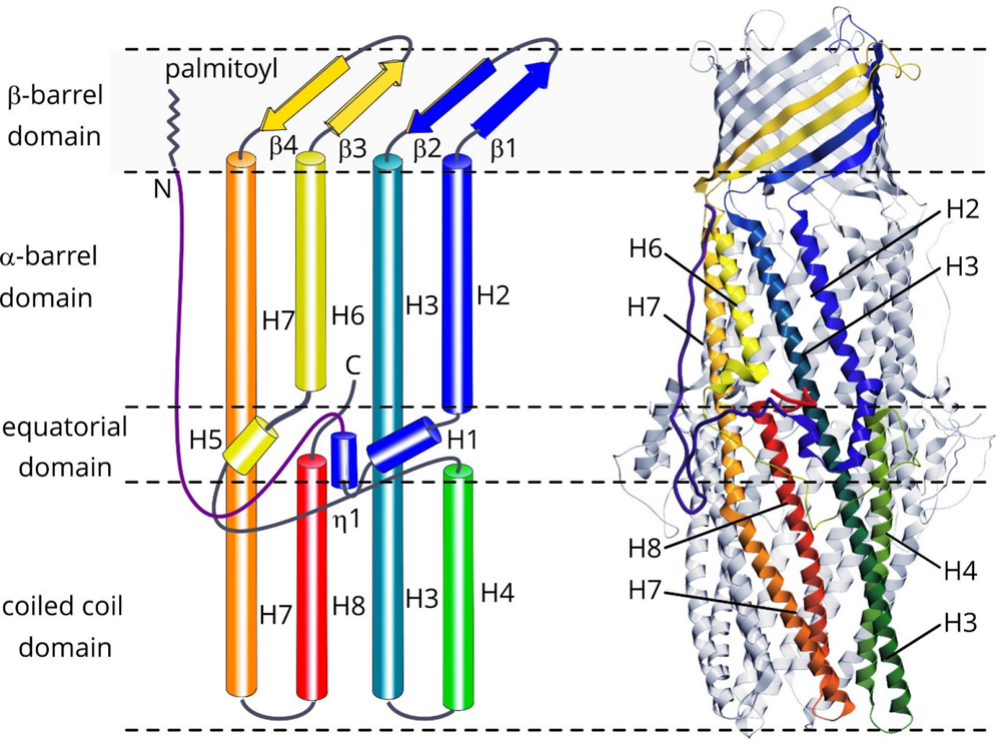
General organization of the typical OMF channel on the example of OprM. (A) Topological diagram. Principal helices (H) and β-sheets (β) are denoted. The chain is colored rainbow from the N-terminus to the C-terminus. Pseudosymmetry within the protomer provided by the structural repeats, arising from a gene duplication event, is evident. (B) 3D structure based on the palmitoylated OprM structure (PDB ID 4Y1K).^643^ See text for more details.

They share a characteristic channel-like organization.^641^ TolC from *E. coli* is a protein consisting of 471 residues whose protomers assemble into a homotrimer. This homotrimer of approximately 150 kDa forms a 140 Å long channel organized in two main domains: a transmembrane domain that adopts a porin-like fold consisting of 12 β-strands organized in a β-barrel of approximately 40 Å inserted into the outer membrane and a periplasmic domain of about 100 Å long. This second domain presents a unique “α-barrel” architecture that consists of 12 α-helices organized into three subdomains: the α-domain, the equatorial domain, and the coiled-coil domain. Each protomer is formed by the repetition of a conserved motif made of two transmembrane β-strands, of a long helix (H3 in repeat one; H7 in repeat two), and finally of two helices aligned along their axis (H2/H4; and H6/H8, respectively), with each having one of their ends at the equatorial domain, thus forming a pseudohelix of the same length as the long helix. These pseudocontinuous helices give the trimer extra flexibility. The equatorial domain (or mixed α/β domain) consists of a mixture of secondary structures (α-helices and β-strands) that forms a belt around the medial part of the α-barrel (see Figure 28).

OprM in *P. aeruginosa* is a large, slightly conical cylinder about 140 Å long, 40 Å wide at the top, and 70 Å wide at the bottom.^269,642^ OprM protomer consists of four transmembrane antiparallel strands (β1, β2, β3, and β4) and eight periplasmic helices (H1 to H8) (Figure 28). The β-strands are composed of a dozen residues which combine to form an antiparallel β single sheet curving at a remarkable angle of ∼60°. Each β-strand is inclined by about 45° relative to the axis of symmetry of the trimer. The H1 helix consists of 13 residues, H2/H6 of 23 residues, H4/H8 of 36 residues, and H3/H7 of 67 residues. The smaller helices join together with the larger ones to form a large, curved sheet of antiparallel helices. Each helix bundle tilts approximately 20° from the axis of symmetry of the trimer. The three protomers come together to form a transmembrane β-barrel about 40 Å long and wide which extends toward the periplasm by a large β-barrel 100 Å long and 70 Å wide. The structure of OprM can be separated into three domains: the transmembrane domain which forms a β-barrel, the periplasmic domain which forms an α-barrel, and the equatorial domain located at the level of the middle part of the α-domain. The latter is a mixed structure consisting of two α-helices (H1 and H5), loops, and 3_10_ helices. The equatorial domain forms a ring around the α-barrel.

Although the structures are very similar, there are some differences between TolC and OprM. First, the N-terminal extremity of OprM is anchored to the outer membrane via a palmityl moiety that originates from the equatorial domain.^642,643,645^ The palmitate anchor provides a second attachment point of the protein to the outer membrane.

The equatorial domain also presents significant variations, particularly in the C-terminal, probably reflecting interaction with structurally diverse PAPs in the tripartite efflux pump. As will be discussed in more detail below, proposed models of assembly between TolC and AcrA show that this loop is involved in the recruitment and interaction of the OMF porin with its PAP.^649,650^ The importance of the equatorial domain has been shown by loss-of-function mutants of TolC.^651−653^ It was shown that TolC requires a leucine residue at position 412, located in an equatorial domain that protrudes from the main body of TolC into the periplasm, to be functional.^651^ Similarly, residues located at the base of carboxy-terminal elongation in the equatorial domain of AatA, homologous to *E. coli* TolC in enteroaggregative *Escherichia coli*, are required for the export of its substrate, Aap.^654^ Finally, the inability for TolC to form a functional complex with CusAB and to confer resistance to copper and silver ions *in vivo* was also suggested to be caused by the seemingly different equatorial domains of CusC and TolC.^648^ In VceC, part of the loop is not visible in the crystallographic structure probably because of its great flexibility.

The C-terminal tail also seems to be of importance for OprM as it is involved in its interaction with MexAB.^655^ The role of the C-terminal tail of OprM has also been studied for the hybrid complementation between OprM and VceAB in order to highlight its role in the specificity of recruitment in the assembly. OprM can no longer function with VceAB when its 20 C-terminal residues are truncated,^656^ but when a modified VceA with mutation in the hairpin domain is used, the function with OprM-Δ20Cter is restored.^657^ As will be discussed below, this region has long been thought to be involved in the interaction with the partner PAP. Overall, the equatorial domain takes on a unique shape for each porin, which is not surprising since it is one of the least conserved regions. Accordingly, major structural differences between OprM, OprN, and OprJ also lie at the equatorial domain and at the periplasmic end of the β-barrel.^645^ However, the H1/H5 helices and the large L1 loop appear to share redundant patterns. In TolC, it was shown that exchanging the H4–H5 loop of TolC with that of other OMFs decreases susceptibility to novobiocin, suggesting that this loop affects the transport activity of AcrAB–TolC; hence, part of loop L1 (N188–K214) is essential for the function of the channel.^658^ Compared to its homologues, TolC is the only one to have an additional β-strand in this domain. Unlike the α- and β-domains, the equatorial domain does not appear to be a duplication of the same structural pattern.

**Figure 29:**
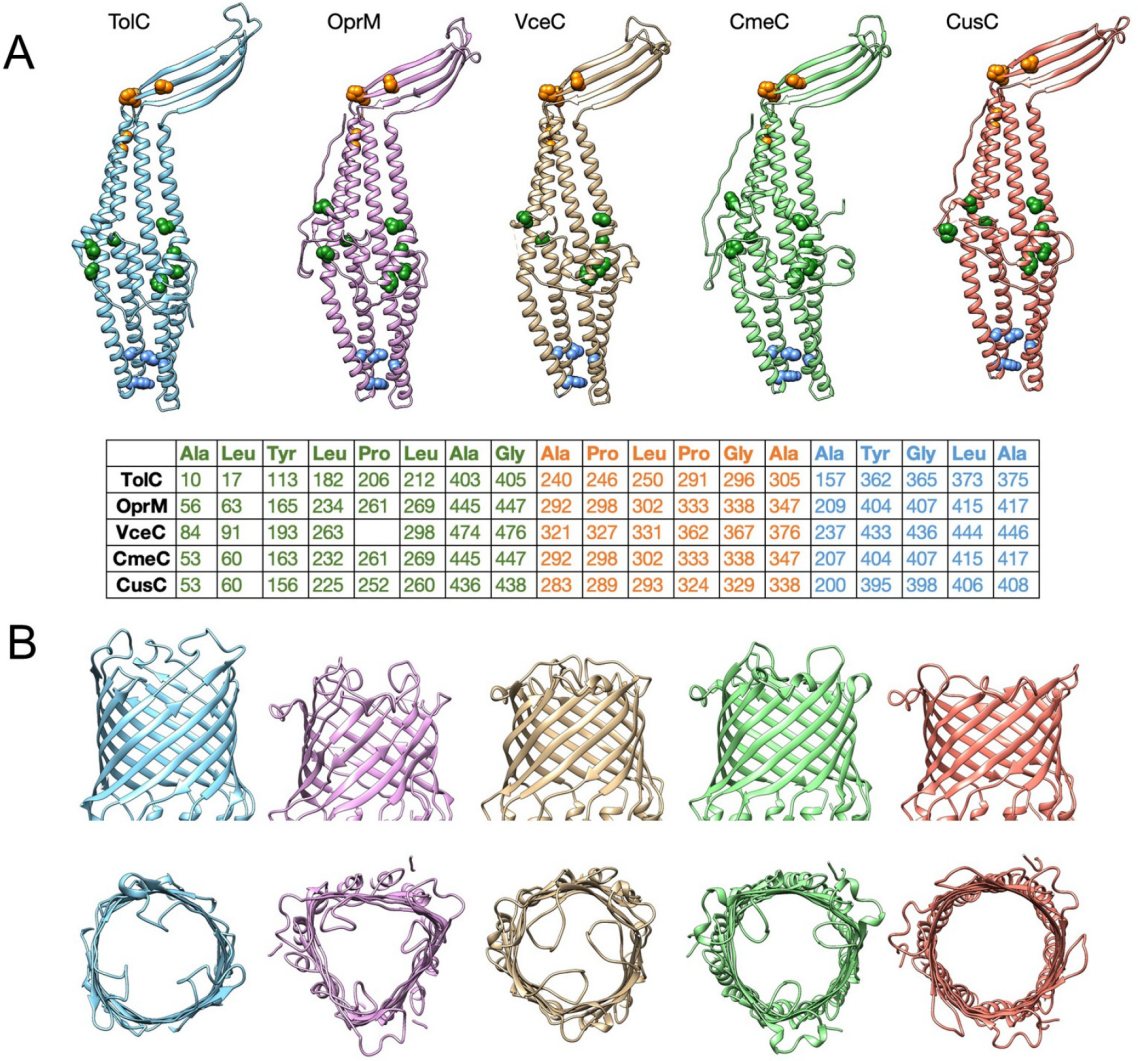
(A) Highlight of the conserved amino acids among 5 OMFs. The protomeric structures of TolC (1EK9, blue), OprM (3D5K, purple), VceC (1YC9, ochre), CmeC (4MT4, green), and CusC (3PIK, red) are shown in the upper part of the panel. The side chains of the preserved residues are represented as spheres of different colors according to their intrinsic location in the protein: orange for the β-barrel part, green for the equatorial part, and blue for the periplasmic part. The position of each conserved amino acid within the primary sequence is shown in the table in the lower part of the panel. (B) Visualization of the β-barrel domains within the quaternary structures of 5 OMFs. Each porin retains the same structural color as in panel A. Two views are shown: a side view first and a top view second.

Despite the strong structural analogy of OprM (PDB code: 3D5K) with its counterparts (TolC, PDB code: 1EK9; VceC, PDB code: 1YC9; CmeC, PDB code: 4MT4; CusC, PDB code: 3PIK), the identity of their primary sequence is rather low (19% for TolC and 23% for VceC). A multiple alignment of OprM and OMPs from different species highlights the residues conserved during evolution (Figure 29A). These residues, which probably play a key role in the structure or function of OMPs of the TolC family, have a preferential distribution at the ends of the α-helices and at the level of the connections between the α-domain and the β-domain. Furthermore, the majority of the conserved residues are located on the C-terminal motif which could be an evolutionarily ancestral sequence.

Gram-negative bacteria encode multiple RND transporters which pair with a number of PAPs and OMFs forming a variety of efflux systems with different substrate profiles (Table 1). TolC from *E. coli* can be associated with several export machineries located in the inner membrane including the α-hemolysin HlyBD-TolC secretion system^61^ and antibiotic efflux pumps (e.g. AcrAB-TolC, MacAB-TolC, EmrAB-TolC). CusC is an exception in the versatility of association among efflux pumps as it is specific for the CusBA CusC metal efflux pump.^10^ OprM from *P. aeruginosa* also shows some promiscuity as it is the common OMF for MexAB,^659^ MexXY,^660^ and MexJK.^661^ In addition, it can replace OprJ and form a complex with MexCD.^662^ However, OprN is only able to interact with MexEF.^82,663^ Electrostatic distribution at the periplasmic entrance regions may be important for formation of functional tripartite efflux pumps. OprM is fully functional in complex with MexAB, MexCD, and MexEF, and OprJ is partially functional in complex with MexAB, whereas OprN is not.^659,662,664^ Crystal structures of the OMFs allowed the suggestion that the electrostatic distribution at the periplasmic entrance regions may be important for formation of functional tripartite efflux pumps containing MexAB. Indeed, electrostatic distribution of OprM at the interprotomer and intraprotomer grooves of the periplasmic entrance of the α-barrel domain is more similar to that of OprJ than that of OprN.^645^ The substrate profile also seems to be a determinant of assembly as it was shown that MexY forms a complex with OprA when it effluxes β-lactams, carbenicillin, or sulbenicillin and with the OprM when aminoglycosides are transported.^665^

Apart from direct involvement in efflux, OMFs play additional roles, notably as adhesion molecules and phage receptors. In *Burkholderia*, the gene for the outer membrane collagen-like protein 8 (Bucl8)^666^ colocalizes with the downstream fusCDE genes encoding fusaric acid resistance leading to the suggestion that ift forms a tetrapartite efflux pump.^667^ The recent characterization of the Bucl8 is an unexpected unique architecture, where the canonical TolC-like N-terminal portion of the protein is followed by two C-terminal regions, one of which is a collagen-like (CL) region composed of an uncommon repeating (Gly-Ala-Ser)_n_ sequence followed by a noncollagen low-complexity C-terminal tail (Ct). The CL-region has been shown to form a triple helix, whereas functional assays screening for Bucl8 ligands identified binding to fibrinogen.^667^ While little is known about this protein, it significantly expands the structural gallery of OMF proteins and suggests additional adhesion and host-invasion-associated functions.

### 6.3 Gates within the OMFs

OMFs of the TolC family display a mean internal diameter of 20 Å (reaching 26 Å at the widest) along the channel. When not engaged with the cognate transporter-PAP pair, both ends of the channel are suggested to be occluded, to prevent uncontrolled diffusion of solutes, despite the fact that some crystal structures (see e.g. CusC, pdb 3PIK, Figure 29B) have been found to have external loops open. We discuss the molecular explanations for the channel gating and the locks that exist along the channel path and how recruitment of protein partners allows for a long-range, allosteric, active, and controlled opening.

#### 6.3.1 Gates within the OMFs: Outer Membrane Loops

Similar to other OMF family members known to date, the transmembrane domain of OprM is a β-barrel formed by the association of 12 antiparallel strands.^668^ In transverse view, the β-barrel of OprM appears to be rather triangular, unlike that of TolC, VceC, or CusC, which are cylindrical (Figure 29B). As pointed out by Wong et al.,^669^ the β-barrel of OMPs is delimited by two rings of aromatic residues, on either side of the membrane, which ensures the stabilization and the vertical positioning of the channel within the phospholipid bilayer. The aromatic residues involved in the stability of the β-barrel are predominantly phenylalanines. They are mainly located at the level of the lower crown which also corresponds to the junction zone with the α-domain. Furthermore, the hydroxyl of the tyrosine residues logically points to the polar groups of the phospholipids. The nature and number of these residues is very variable from one porin to another. Unlike OprM and VceC, aromatic residue crowns are not well-defined in TolC. In the case of VceC, the side chain of residues F340, Y131, and Y143 is localized inside the porin and therefore does not interact with the cell membrane. It is possible that these residues reorient outward to stabilize the β-barrel when the canal is opened.

The cavity of porins is generally capped by a large loop which regulates the flow. In the case of TolC and its counterparts, there is no such loop, and the interior of the channel is therefore completely free. However, extracellular loops, topping their upper end, were postulated for the closed state observed in crystallographic structures. Short molecular dynamics simulations initially suggested that large motion of the extra cellular loops indeed plays a gate function over the β-barrel like a lid.^670^ However, simulation performed in the presence of NaCl ruled out this outer gate hypothesis and concluded that TolC is locked at the periplasmic side only.^671^ Differences most probably arose because it was shown that at physiological values of ions, cations of the solvent are seemingly able to constitute ionic bonds in place of the removed salt bridges, which inhibited the opening of the aperture in simulations.^672^ Similar studies performed for OprM showed that extracellular loops open and close freely in the simulations, suggesting that in the conditions of the simulation (isolated protein, absence of peptidoglycan and LPS), there is no gating mechanism on the extracellular side.^673^

Studying the influence of the composition of the outer membrane on porin is a difficult experimental challenge, and examples of reliable *in vitro* models of envelopes of Gram-negative bacteria are scarce in the literature.^674,675^ Remarkably, major advances in the field of molecular dynamics now allow for more and more accurate models of very complex lipid environments.^676−678^ In the latter study, a fully assembled OM bilayer of *P. aeruginosa* was built *in silico* in order to understand the molecular determinants contributing to the rigidity and stiffness of LPS-containing bilayers, e.g the low permeability of cell envelopes of Gram-negative bacteria. In addition, OprM was inserted, and its conductivity was calculated in a model OM by comparison to a pure 1-palmitoyl-oleoyl-*sn*-glycero-phosphocholine (POPC) bilayer. The authors conclude that the presence of LPS modulates the conductivity of the channel, increasing its conductance (from 600 pS to 890 pS) and increasing the selectivity toward anions in an LPS-embedded OM bilayer by comparison to what is found in pure POPC bilayers. Accordingly, LPS was found to interact with the residues of the extracellular loop of the protein through the interaction of a network of hydrogen bonds. Opening of the outer gate was not affected by the presence of LPS *per se* but had a more pronounced interaction with the loops which is in good agreement with a direct effect on the function of the porin, as already shown for other porins.^679−681^ A cryo-ET structure of AcrAB-TolC in *E. coli* intact cells^682^ suggests a critical role of the loop in keeping the outer cavity open: according to this structure, upon interaction with LPS at the vicinity, the outer diameter is kept 10 Å wide, which is enough to expel substrates.

#### 6.3.2 Gates within the OMFs: Knob-into-Holes and Bistable Switching between Opening and Closing of the α-Helical Bundle

Behind the structural similarities of the α-barrel domain of OMFs of the TolC family is hidden interhelical interaction geometries that differ according to the porin, in particular at the level of the upper α-cylinder. They have been described through geometrical principles derived from the definition of coiled coils by Francis Crick.^683^

**Figure 30:**
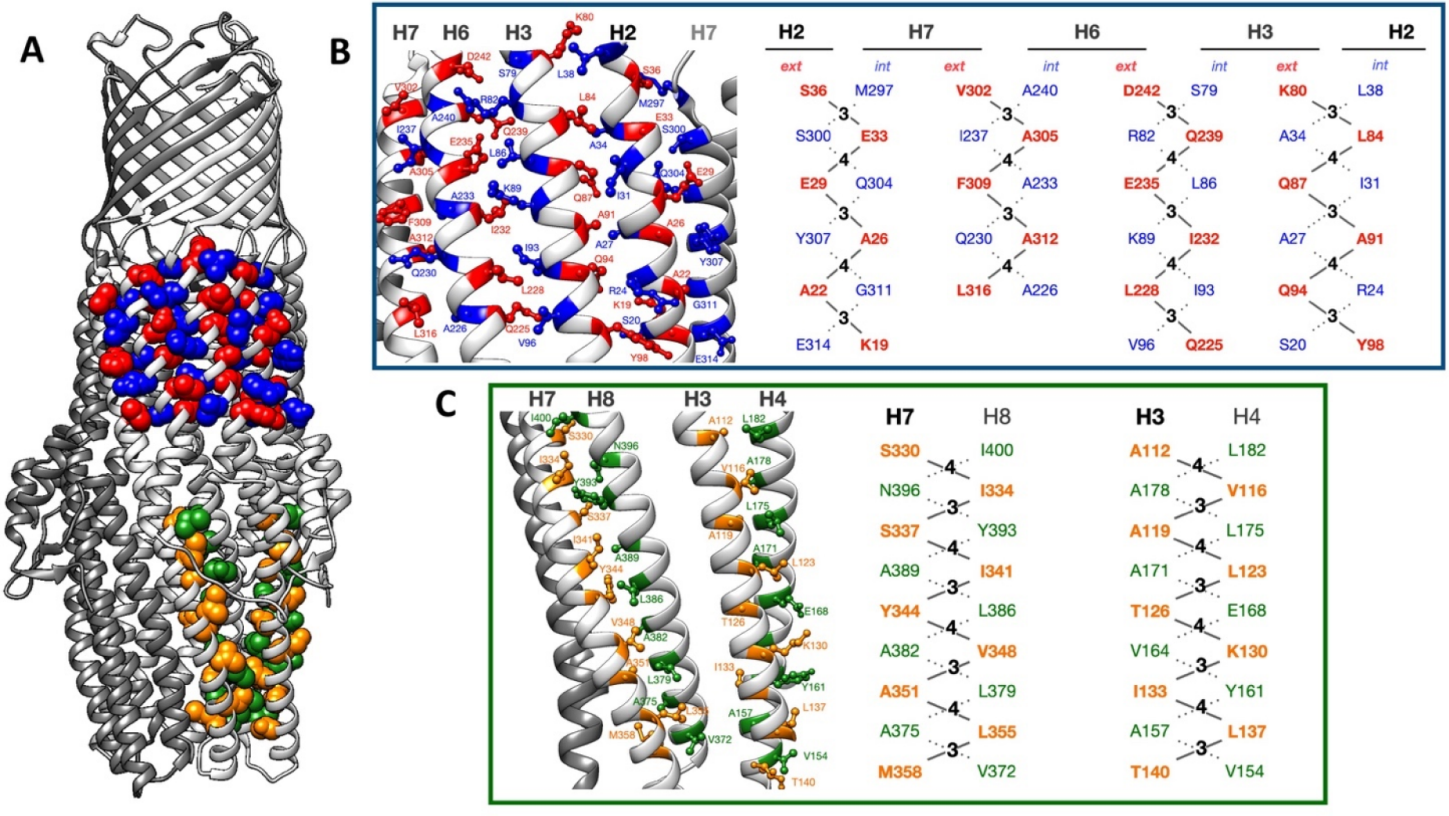
“Knobs into holes” packing analysis of the helix–helix interfaces of TolC. (A) The structure of TolC (PDB ID: 1EK9) is visualized as a ribbon, with residues forming canonical coiled-coils (yellow and orange spheres) and residues forming noncanonical coiled coils (red and blue spheres). (B and C) A closer look into the knobs-into-holes packing patterns at the interfaces between α-helices, which are also depicted in “ladder” representation. Helical IDs are listed at the top, and amino acid residues are specified by the one-letter code. Heavy lines and bold helix identification numbers correspond to α-helices viewed from the exterior, while dotted lines and light identification correspond to α-helices viewed from the interior. (B) The layout of the four interfaces between α-helices participating in the construction of the 12-helix tube forming the α-cylinder. (C) The layout of the two interfaces between α-helices in the construction of the four-helix bundle forming the canonical coiled-coil restricting the periplasmic extremity of the TolC channel.

In TolC, the α-barrel is separated into two subdomains, on either side of the equatorial domain. In its lower part, H3 and H7 initiate supercoiling with H4 and H8, respectively, hence forming two canonical *coiled coils* which, in turn, result in a curvature of the α-helices of about 20° with respect to the axis of the channel curl and constrict to close the lower end of the porin. Formed with a periodicity of seven residues over two helical turns and a supercoil in the opposite direction of the helices, these structures are stabilized by the interlocking of side chains, most often nonpolar (dubbed “knobs”), in small concavities (dubbed “holes”) formed by four residues of the opposite helix. The *packing cutoff* is the distance between the “knobs” residue and those which form the “holes”, which is classically 7 Å.^684^ The periodicity of the lattice line that contains the hydrophobic residues determines the “helix crossing angle”, e.g. the hand of the supercoiling^685^ (Figure 30).

The upper part corresponds to an organization called the α-cylinder,^686^ that can be imagined to be like an assembly of coiled-coils. An α-domain, corresponding to a large barrel formed from hydro soluble α-helices encompassing half of the periplasmic space, is the signature of TolC-like proteins. The interacting helices do not coil or adopt the canonical “knobs-into holes” configuration because they have to adjust along the curved surface of the cylinder to maintain a perfectly uniform diameter. Two distinct deformations are needed: the bending of the helices in a curve and the untwisting along the axis so that they arrange like a sheet with the α-cylinder formed by the association of three curved leaflets. By contrast to helices found in classical two-stranded coiled-coils, the axis of each helix is now tilted and translated, and α-barrel helices untwist and distort so as to make their knobs approach closely one end of the complementary holes. In addition, each helix packs laterally with two neighbors to form parallel or antiparallel sheets. As a consequence, this arrangement must fulfill geometrical constraints on two separate interfaces simultaneously, on either side of the helix. The periodicity of amino-acid side chains is crucial because of the mechanical requirements for interfacial compression and expansion on either side.^685^

#### 6.3.3 Gates within the OMFs: Primary and Secondary Periplasmic Gates

The periplasmic end is a key region for channel opening^641,668,687^ as the constriction at the periplasmic end has to allow the passage of cargoes as large as α-hemolysin (160 kDa). The coiled-coil-derived “hydrophobic core” does not have a sufficient stabilizing effect for the tight maintenance of the bundle, and additional specific salt bridges and/or hydrogen-bonding links between adjacent α-helices are necessary to stiffen the interactions between the internal and external helices or between protomers. Reciprocally, the positioning of an intertwined coiled-coil on top of the α-cylinder leads to an intrinsically tensed edifice, so that opening movement can spontaneously arise upon the mere breaking of the periplasmic gate(s): removal of the hydrogen bonds and the salt bridges between these residues causes a reorientation of the internal helices (H4 and H8) and the external helices (H3, H7) of the trimer linked to the relaxation of the torsional stress and to a widening of the periplasmic pore. An additional constriction, located higher in the channel, is constituted by an anionic ring formed by the carboxylate groups of the side chains of the Aspartate residues present in this region. This ring forms a cation-selective filter. These two obstacles do not allow the efflux of substrates and need to be removed before a productive efflux event.

**Figure 31:**
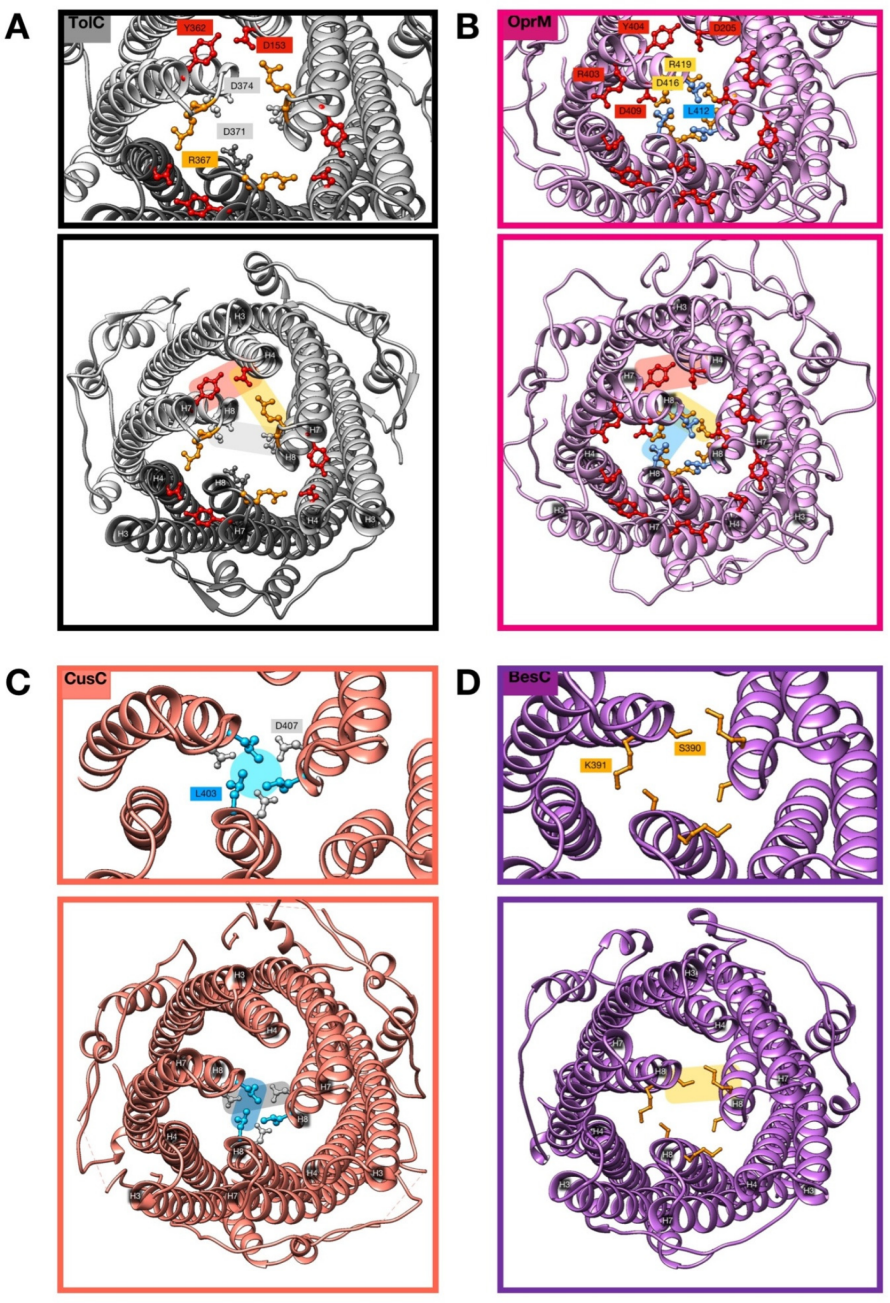
View of the periplasmic tip of representative OMFs: TolC (1EK9), OprM (3D5K), CusC (3PIK), and BesC. (A) TolC. (B) OprM. (C) CusC. (D) BesC. Structures are shown as cartoons, and residues involved in the channel gating are shown as ball and sticks representation, colored depending on the type of interactions between them. In the resting state of the channels, the trajectories of the H7/H8 helices are bent and they are prevented from adopting a relaxed superhelical trajectory by anchoring interactions with the H3/H4 helices, known as “primary gates”. These are suggested to be disrupted upon interaction with the helical-hairpins of their PAP-binding partners. Intraprotomer interactions within primary gates are colored red; interprotomer interactions are shown in orange; van der Waals interactions forming the hydrophobic gates in, e.g., OprM and CusC are colored blue. The aspartate ring forming the “secondary” gate is shown in gray. The predicted primary gates of BesC (homology model), from the *B. burgdorferi* pump BesABC, which operates with the PAP BesA, that lacks the helical hairpin domain, are significantly less stable.

The residues involved in the formation of the salt bridges and intra- and interprotomer hydrogen bonds at the extremities of the periplasmic helices differ between OMFs (Figure 31). In TolC, the key interaction providing the obstruction of the OMF periplasmic end is the intraprotomer bond Y362–R367 that tethers the inner coiled-coil H7/H8 to the outer H3/H4 coiled-coils. There, two major locks were described: the D153–Y362 intraprotomer hydrogen bond between helices H4 and H7 and the D153–R367 interprotomer salt bridge between helices H4 and H8. In addition, T152 also provides coordination to R367.

Mutational inactivation of the charged interactions that anchor the H7/H8 to the H3/H4 helices results in spontaneous relaxation of the superhelical trajectories of the former, leading to a “leakiness of the channel” that could be detected by electrophysiology essay,^687^ and the resulting relaxation was also trapped in a crystal structures of the TolC channel.^688^ That led to the suggestion that these residues are targeted during the formation of the tripartite complex not the partner proteins, and they were dubbed “primary gates”.^689^ The exact mechanics of these interactions are discussed in the following sections, but it is also notable that in the TolC D371 and D374,^690^ a double concentric ring is formed above the primary gates, which also prevents the passage of any cargo beyond cations, which is often referred to as “the secondary gate”, as it has to be disrupted to allow passage of the cargo.

In order to validate the importance of these residues, site-directed mutagenesis and electrophysiology experiments were carried out. On TolC, by electrophysiology measurements carried out on the wild-type form and on the D371A-D374A mutant, it was shown that porin was a selective channel with respect to cations and this via a ring of negative charges formed by D371 and D374.^687,690,691^ Breakdown of three inter- or intramolecular bonds (D153A–Y362 between H4 and H7 intraprotomer, R367S–D153A and R367S–T152 V between H4 and H8 interprotomers) induces a 6-fold increase in conductance (500 pS instead of 80 pS) within the channel compared to the wild-type TolC protein, reflecting a greater opening. Combining mutations R367S and Y362F even made it possible to increase the conductance further. Inactivation of the ionic bond network at the R367S and Y362F mutants relaxes the protein accompanied by an increase in conductance at 205–370 and 800–1000 pS, leading to an approximately 16 Å opening at the periplasmic end.^687^ A reverse experiment, aiming at blocking the movement of the helices thanks to the introduction of cysteines, confirmed, both by electrophysiology and complementation in a strain of *E. coli*, the importance of the opening of the helices for the efflux of antibiotics. Indeed, if a disulfide bridge between the H4 and H7 interprotomer helices (A159C–S350C) reduces the export of hemolysin by a factor of 5, it is completely abolished when the H4 and H8 helices or Asp374C is cross-linked.^692^ However, these mutants retain the ability to interact with their partners, which suggests that the movement of the helices is essential for the efflux of molecules. The importance of residues D374 as well as D371 was later confirmed by a biochemical and structural study of the porin in the presence of hexaamminecobalt which has a 20 nM affinity for TolC and forms a plug at the entrance to the periplasmic pore upon interacting with aspartate D374.^693^ The TolC periplasmic end is tightly sealed, and several laboratories have provided insights into how the association of the channel with its inner membrane partners may cause its opening,^694−698^ as will be discussed with more details below.

In OprM, the equivalent residues are not all conserved (Figure 31B). Surprisingly, only one aspartate ring is present at D416 (corresponding to D374 in TolC), contributing to the closure by creating three interprotomer salt bridges with R419. Other salt bridges were also described as important for the opening mechanism: between D205–Y404 (equivalent to D153–Y362 in TolC) and D209–-R403 (D153–R367 in TolC). In addition, L412 adds additional “glue” at the smallest constriction of the tunnel via van der Waals interactions. Note that X-ray crystallography under xenon gas pressure located xenon trapped in a sandwich between the three symmetry-related corresponding leucines in OprN (L405) forming a tight hydrophobic constriction of the channel. Xenon was further coordinated by the three-symmetry related D409 (corresponding to D416 in OprM), part of a salt bridge with R412 (R419 in OprM).^644^ Recent cryo-EM structures of the complete MexAB–OprM pump^270,306^ have allowed for a detailed reconstruction of the trajectories of the gating helices, which are discussed in section 8.2 (Figure 42).

Electrophysiology recordings were similarly used for the characterization of OprM^669,699^ and EefC from *Enterococcus aerogenes*.^700^ Similarly, D371 and D374 are conserved in TolC of *E. aerogenes* (D365 and D368) but are replaced by a serine (S401) and a threonine (T404) in EefC. Both TolC from *E. coli* and *E. aerogenes* have similar ion selectivity. EefC was found approximately 10-fold lower than that of *E. aerogenes* TolC. Interestingly, single channel conductance studies performed at acidic pH showed that conductance decreases when pH decreases,^691,700^ probably because protonation of D371 and D374 reduces the cation selectivity and the like charge repulsions that help to expand the tunnel entrance. This hypothesis was supported by systematic molecular dynamics simulations of WT versus mutated TolC that showed that the closed state is favored at low pH.^701^ Along similar lines, MD simulations suggest that cations of the solvent may form ionic bonds when salt bridges are removed, thereby inhibiting the opening of the aperture.^672^ More specifically, TolC seems to be locked in a sodium-dependent manner: the inner periplasmic bottleneck region at D374 is kept closed unless all NaCl is removed from the simulation.^671^

The resolution of the X-ray structure of VceC^646^ suggested that opening occurs by changes at the interfaces between helix H4 of one protomer and the helical pair H7–H8 of the adjacent protomer concomitant to changes between helices H3 and H7–H8 of the same protomers. VceC is structurally closer to OprM than to TolC, and comparison of the structures revealed that hydrogen bonds and salt bridges are not conserved between VceC and TolC (Figure 31C). Although we do not have structures of the open VceC channel, it has been supposed that it is stabilized in its closed conformation by a different set of interactions than those present in TolC. The two intraprotomer links D153–Y362 and Q136–E359 from TolC are not conserved in VceC. The TolC D153 corresponds to S233, and the serine side chain interacts with a glutamine rather than the corresponding tyrosine.

TolC Q136 and D359 correspond in VceC to T216 and T430, respectively. The side chains of these threonines are too distant to form a hydrogen bond. None of the TolC interprotomer salt bridges (D153–R367 and R367–T152) are possible in VceC where the D153, R367, and T152 are respectively replaced by S233, A438, and G232. In CusC, the surface of the periplasmic end is relatively hydrophobic. The channel is gated by van der Waals contacts between L403 residues from the three protomers. An adjacent ring of negatively charged residues (D407) provides an additional layer of closure.^648^ TolC is not able to functionally replace CusC in the CusABC-mediated resistance to copper and silver,^357^ probably because the α-helical subdomain of CusB is very different from that of all other known PAPs.^702^ For the sake of systematic comparison, we have computed a model of BesC from *Borrelia burgdorferi*(703) based on the structure of VceC. It shows a gating merely located on H8, through interprotomer salt bridges between K391 and S390 (Figure 31D). This rather simplistic lock is consistent with the fact that its corresponding PAP has no actual hairpin and may in fact not be involved in maintaining the OMF open.

Opening of the channel is necessary during the translocation process, and it was long thought that the other two partners were needed to open. Deletion of efflux pump genes gives rise to various sensitivity profiles,^56^ and use of drug sensitive mutants to monitor the effect of pump deletion, sequence insertion, and site-directed mutagenesis and to select suppressor mutants has long been a method of reference^311^ to depict what could be the residues involved in the opening of the OMFs. In the following we describe how this strategy that allowed understanding of the reversible assembly of the pump acts as a switch for opening of the periplasmic gate. The role of the PAP in the bridging and in the stabilization of a tripartite pump has been well documented. For instance, Mokhonov et al.^704^ showed that there is tight association between MexA and OprM in the absence of MexB, whereas the expression systems lacking MexA failed to copurify MexB or OprM. The same conclusion was raised from *in vitro* experiments where MexAB OprM was reconstituted in respective liposomes and subjected to pull-down *in vitro*.^705^ But the PAP is certainly much more than a simple connector and could play an active role in channel opening by transmitting the work liberated at the level of the RND transporter, in the inner membrane. The interplay within the tripartite assembly is of definitive complexity. The pump is described as a highly allosteric system in which conformational changes associated with ligand binding to the RND are communicated to the OMF^305^ and, reciprocally, OMF induces activation of the RND via its PAP (see below), indicating that a mutual interplay exists between the respective partners of the pump.

For instance, it was shown that the PAP is mandatory for the activity of the pump.^311,706−708^ Mutual functional interactions have been thoroughly investigated using genetic screens for AcrAB-TolC.^689,695,709−712^ Lower ion bridges can be destabilized by direct interaction with transporters with large periplasmic domains, such as the RND family.^711^ However, the Asp-rings were believed to be too far up the channel to be directly affected by the transporter and likely “unlocked” via interaction with the tip of the PAP. Successful unlocking of these bridges designated “primary” and “secondary gates” respectively, is a requirement for productive transport.^689^ The open structure of TolC presents extensive interaction interfaces between the periplasmic and outer membrane proteins, defined by the helices H3–H4 and helices H7–H8 from TolC. Site directed mutagenesis and docking simulation of the coiled-coil domain of AcrA in these grooves were performed and suggested that a functional salt bridge could form in the groove and the equatorial domain, accounting for effects of mutations in this exact region on activity,^709^ in line with mutagenesis,^652,711,713−715^ cross-linking studies,^650,716^ and mapping of the gain-of-function suppressor alteration of an inactive TolC mutant^710^ or mutations in the α-helical region of AcrA allowing cross reactivity within the nonfunctional heterologous TolC-AcrA-MexB pump.^717^ It has been proposed that PAP–OMF interaction can also be dependent on the substrate.^84,661^ For the first time, substrate specificity was also found within AcrAB-TolC after analyzing the effects of site-directed mutations targeting the α-barrel of TolC on substrate transport substrate specificity.^718^ Marshall and Bavro^718^ showed that those residues affecting the electrostatic properties of the channel caused hypersusceptibility to all efflux-tested substrates and affected cells’ ability to grow even in the absence of antibiotics. Interestingly, mutations were shown to be substrate specific.

Understanding the energy requirements for the assembly has also been the subject of a wide range of converging studies. For instance, the energization of the transition from the closed to open state of MtrE from the efflux pump MtrCDE of *N. gonorrhoeae* was evaluated by assessing the sensibility of the cell to vancomycin. This antibiotic is a very large molecule that does not penetrate the Gram-negative membrane unless the OMF is sufficiently open to passively diffuse into the cell.^689^ Cells expressing the wild-type MtrE or a mutant (E434 K) which opens more easily than the wild-type remain resistant to vancomycin, but if MtrC is now expressed, cells become more sensitive. This means that MtrC is required by MtrE to open.^719,720^ In addition, the full-length PAP is required to open MtrE since a truncated version of the PAP, consisting of the α-helical hairpin domain only, is not able to open the OMF. When the RND, MtrD, is expressed with MtrCE, cells are resistant to vancomycin whereas when expressed with MtrCE^E434K^ cells are susceptible to vancomycin.

**Figure 32:**
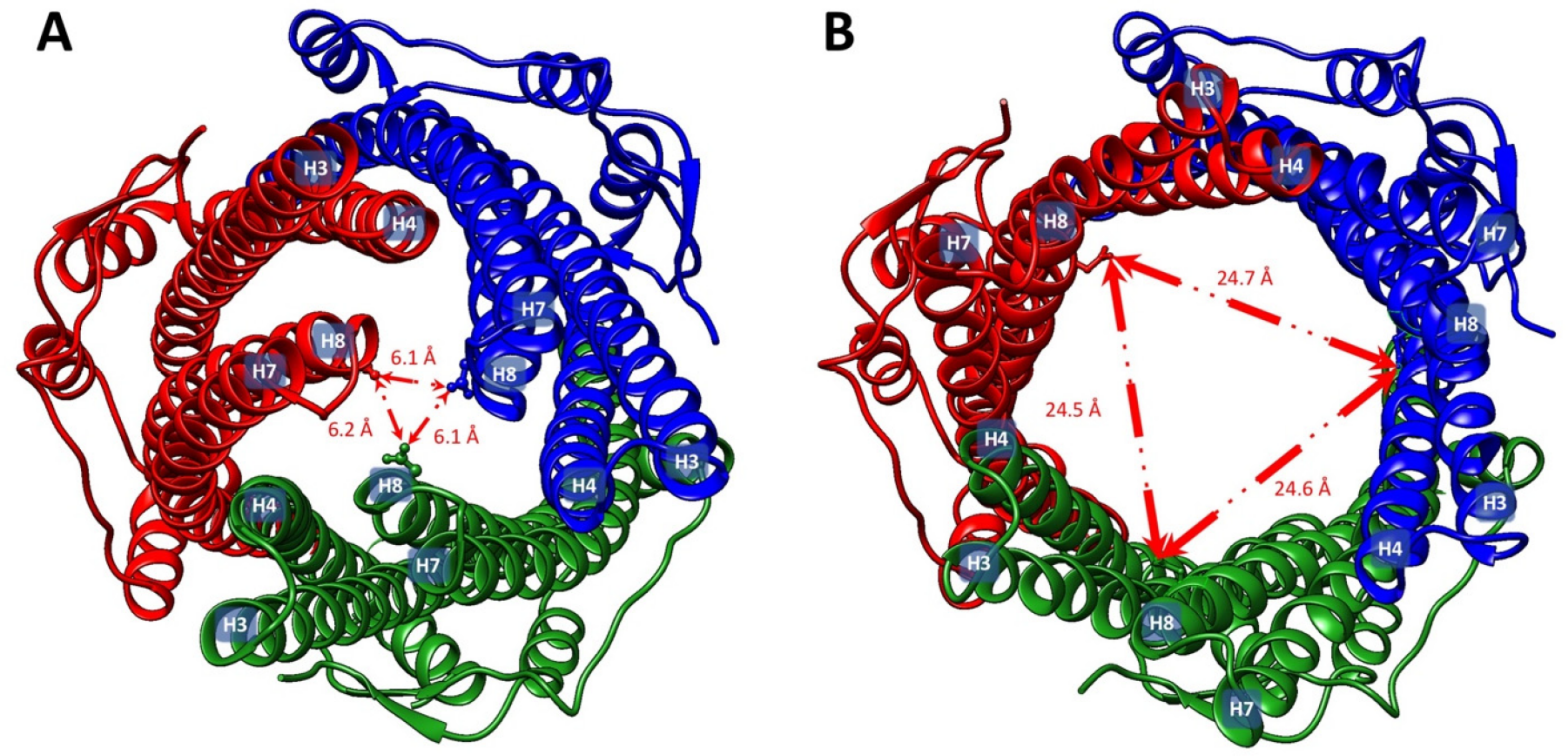
Periplasmic view of TolC in its resting, closed conformation (PDB ID 1EK9) and after full dilation of its aperture upon engagement with the PAP protein as seen in the assembled tripartite complex PDB ID 5NG5. Structures are shown as cartoon view, and aspartate D374 is shown as ball and sticks. Distances between hydroxyl groups of the carboxylic acid of D374 are measured.

The energy dependency of the pump assembly was investigated, again using nonfunctional mutants of MtrD, mutated in one or more residues involved in the proton-transducing pathway (D405, D406, and K948). If these mutants are expressed with MtrCE or MtrCE^E434K^, cells remain insensitive to vancomycin. As discussed above, the TolC^YFRE^ variant had significantly lower affinity to AcrB,^698^ suggesting that opening of the channel could lead to destabilization of interactions, disengagement of the complex, and channel closing. Thus, the integrity of the assembled tripartite pump seems to be transient, with a proton-independent assembly and a dissociation that is probably performed upon closing of the OMF, which is allosterically coupled with the active transport of proton across the RND transporter. This claim was later confirmed *in vitro* upon reconstitution of the various partners of the MexAB OprM pump into proteoliposomes.^705^ This is also in accordance with what was shown for the hemolysin secretion system, in which energy consumption is not necessary for the assembly of the type 1 secretion system^721^ and *in vivo* cross-linking copurification and ITC experiments showed that the AcrAB-TolC does not require the proton motive force to assemble.^694,696^ Ultimately, structural determinations allowed for a detailed description of the periplasmic opening (Figure 32).^688,689,693^ Opening of the periplasmic end is believed to occur in a comparable manner to a photographic diaphragm where the “internal coiled-coils” undergo twisting to align with the “external coiled-coils” H3/H4.^690^ Structural and molecular dynamics studies^641,642,689^ later confirmed the photographic diaphragm-style opening mechanism to untwist the helices at the level of the coiled-coil domains.

## 7 Periplasmic Adaptor Proteins (PAPs)/Membrane Fusion Proteins (MFPs)

**Figure 33:**
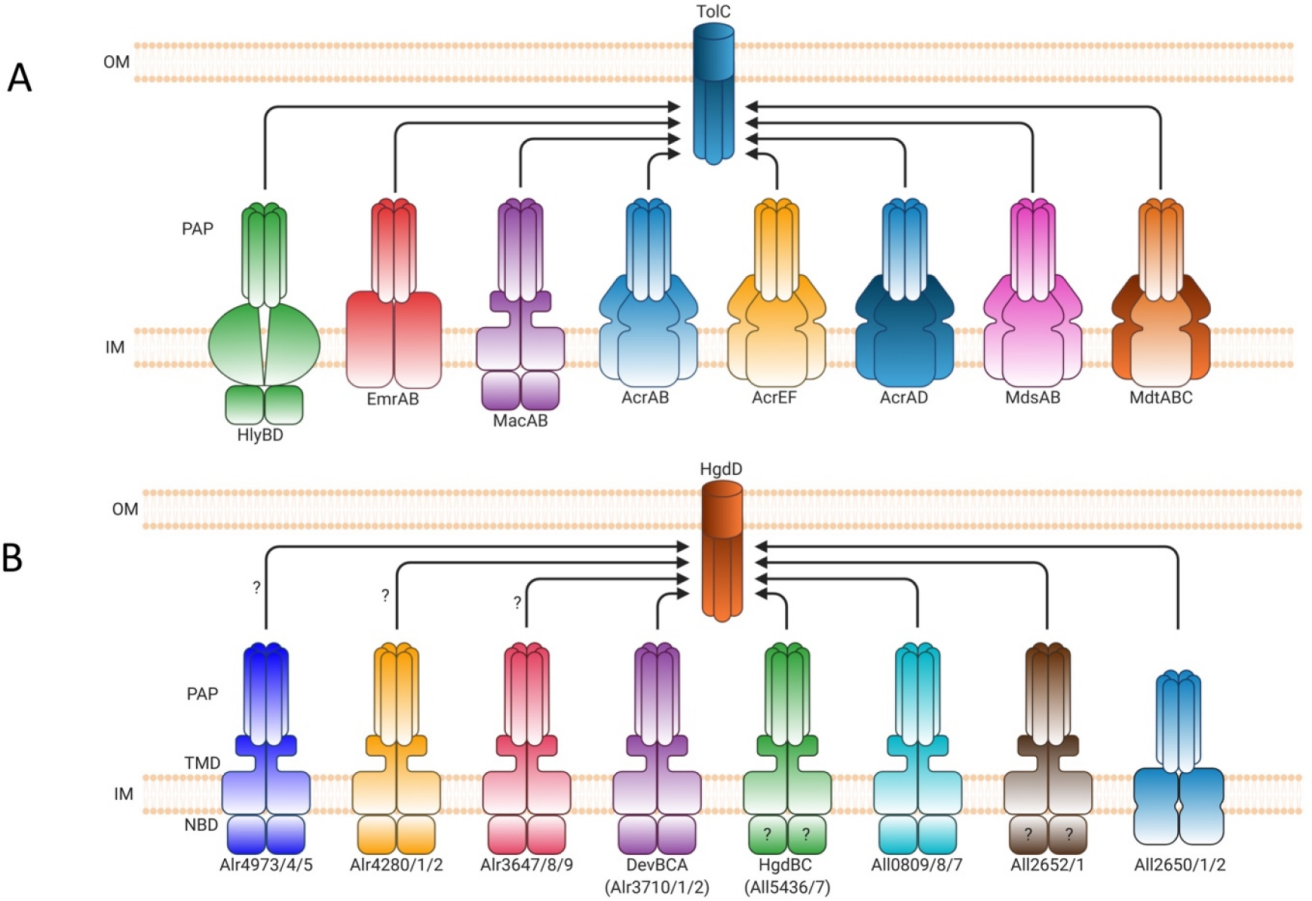
Diversity of PAPs suggesting promiscuity of assembly. (A) In *Salmonella*, at least eight different tripartite assemblies are formed with the participation of the OMF TolC. (B) In *Anabaena* sp. PCC 7120,^553^ the OMF HgdD partners with at least eight different PAP–transporter pairs. Notably, while the tripartite assemblies in *Anabaena* are dominated by MacB and ABC transporters, the RND-based assemblies are predominantly represented in *Salmonella.* The established roles of the ABC transporters in the lower panel are indicated.

The periplasmic adaptor proteins (PAPs), or as they were previously referred to, “membrane fusion proteins” (MFPs),^722^ are a wide and diverse family of transporter-associated proteins the primary function of which is to allow pairing of a limited set of outer membrane factor (OMF) proteins to a wide range of inner membrane transporters ensuring high-fidelity and selectivity of assembly, thus contributing to the combinatorial diversity of the tripartite efflux pumps and greater plasticity of the response to xenobiotic stress.^100,723^ The unique feature of PAP proteins that makes them central to the assembly of the trans-envelope pump complex is their ability to interact simultaneously with both the inner-membrane transporter and the OMF using distinct protein–protein interfaces. In this way PAPs allow for “promiscuity” of the OMF relative to the inner membrane transporter, allowing the docking of a number of different transporter types to a single OMF. In *Salmonella* at least seven tripartite complexes are formed, including the five RND complexes (AcrAB, AcrAD, AcrEF, MdsAB, and MdtABC), MFS EmrAB, as well as the ABC transporter bases MacAB that include the TolC as an OMF,^724^ which is made possible by the range of PAPs (Figure 33A). Similarly, at least eight separate PAPs appear to dock to the OMF HgdD in *Anabaena* sp. PCC 7120^553^ (Figure 33B).

The existence of a wide range of PAPs that are able to interact with a single OMF indicates that the primacy of the determinants of substrate specificity resides with the specific PAP–transporter pair, although the OMF may provide some limited substrate differentiation.^718^ Despite their central importance for the transport process, the role and function of PAPs within the assembly remains least-well understood. In the pages below we will try to summarize the recent advances in our understanding of their function. Previous systematic reviews from recent years have focused in more detail on the diversity and phylogenetic grouping of PAPs^725^ and structural organization and overall mechanisms of PAP–transporter coupling,^726,727^ respectively.

### 7.1 Historical Note

PAPs participate in the formation of tripartite systems with a number of different transporter classes including ABC transporters (HlyD,^728^ MacA^60,206^), MFS transporters (FarAB-MtrE^79^), and RND transporters; however, at the time of writing there have not been any reports of tripartite assemblies formed with the PACE, MATE, or SMR systems. Tripartite efflux systems built around the ABC transporters forming the RTX-toxin secretion systems, which later became known as T1SSs, were the first tripartite systems to be identified and provided the blueprint of the organization of the other such systems, including the multidrug efflux pump assemblies. The first PAP genes were cloned in association with the hemolysin A (HlyA) secretion system from *E. coli*(729) and the leukotoxin secretion systems from *Pasteurella haemolytica*.^535^ The HlyBCD system built around the ABC transporter HlyB was one of the first secretion systems to be characterized, and its homology to P-glycoprotein allowed some of the first models of efflux function in the context of multidrug resistance to be proposed.^730^ The first indication of the periplasmic localization and organization of the PAPs has consequently also come from topological mapping of the HlyD, part of the ABC-based T1SS HlyBCD responsible for hemolysin export.^728,731^ It also provided the first insight into the PAP function and linkage to TolC.^732^

Subsequent bioinformatic analysis of the available PAPs suggested that they form a novel, clearly defined group, which was ascribed “membrane fusion” capability, based on the superficial resemblance of hairpin domain architecture to the coiled-coil domains observed in *bona fide* membrane fusion proteins, e.g., paramyxoviral SV5.^722^ This inferred fusion function has never been demonstrated in any PAP since; however, the old-name association still persists, and they are sometimes referred to in the literature as “membrane fusion proteins” or MFPs.

### 7.2 Structural Organization of PAPs

Periplasmic adaptor proteins display significant structural diversity; however, they are built around a common blueprint. Topological analysis of the PAP-fold reveals a rather unique nonlinear architecture, with each of the domains being constructed with the participation of both N- and C-terminal halves of the protein, due to the fact that the polypeptide chain is effectively folded in the middle, similar to a “hairpin” or “safety pin” arrangement.^733,734^ While both N- and C-termini of the proteins are either associated with, or held close to, the inner membrane, the elongated body of the molecule extends away from it into the periplasmic space, eventually reaching the OMF.^725,726^ This body is composed of three to four “core” domains but is often augmented by additional N- and C-terminal modules, which are specific to the different families of PAPs and are dictated by the cognate transporters.

Rapid advances in the structural characterization of the PAP proteins over the past decade have shed light on all of the main group members, although the RND-associated proteins were the first to be solved; for exampel, MexA (PDB ID 1T5E;^733,734^ PBD ID 2V4D(716)) and AcrA (PDB ID 2F1M(735)) remain the best-studied examples due to the plethora of structures of assembled tripartite complexes with their participation.^270,287,305,306^ The structures of the heavy-metal efflux (HME) RND pump-associated PAPs have also been resolved both in isolation, e.g., the copper-efflux associated CusB (PDB ID 3H943OOC, 3OPO, 3OW7)^702^ and ZneB from *Cupriavidus metallidurans* (PDB ID 3LLN),^63^ and in complex with cognate transporters, e.g. CusBA (PDB ID 4DNR, 3T51, 3T53, 3T56, 3NE5, 4DNT, 4DOP).^359,610^

The structures of MacB-associated PAPs from *E. coli Ec*MacA (PDB ID 3FPP)^736^ and *Aggregatibacter (Actinobacillus) actinomycetemcomitans Aa*MacA (PDB ID 4DK0)^737^ and the structure of the full MacAB-TolC pump have been reconstructed from cryo-EM data (PDB ID 5NIL-5NIK).^272^ The structures of the PAPs associated with the MacB-type Gram-positive efflux systems have also been resolved, including the *Bacillus* YknX^594^ and the Spr0693 from *Streptococcus pneumoniae* R6 (PDB ID 5XU0),^571^ both of which assemble into a hexameric pattern very similar to MacA.

The first MFS transporter-associated PAP was EmrA from *Aquifex aeolicus* (PDB ID 4TKO).^738^ The last group of PAPs to reveal their structure was the PAPs associated with T1SS. The structure of *E. coli* HlyD (PDB ID 5C21, 5C22)^739^ was followed by the Serratia *marcescens* LipC PAP (PDB ID 5NEN).^514^

**Figure 34:**
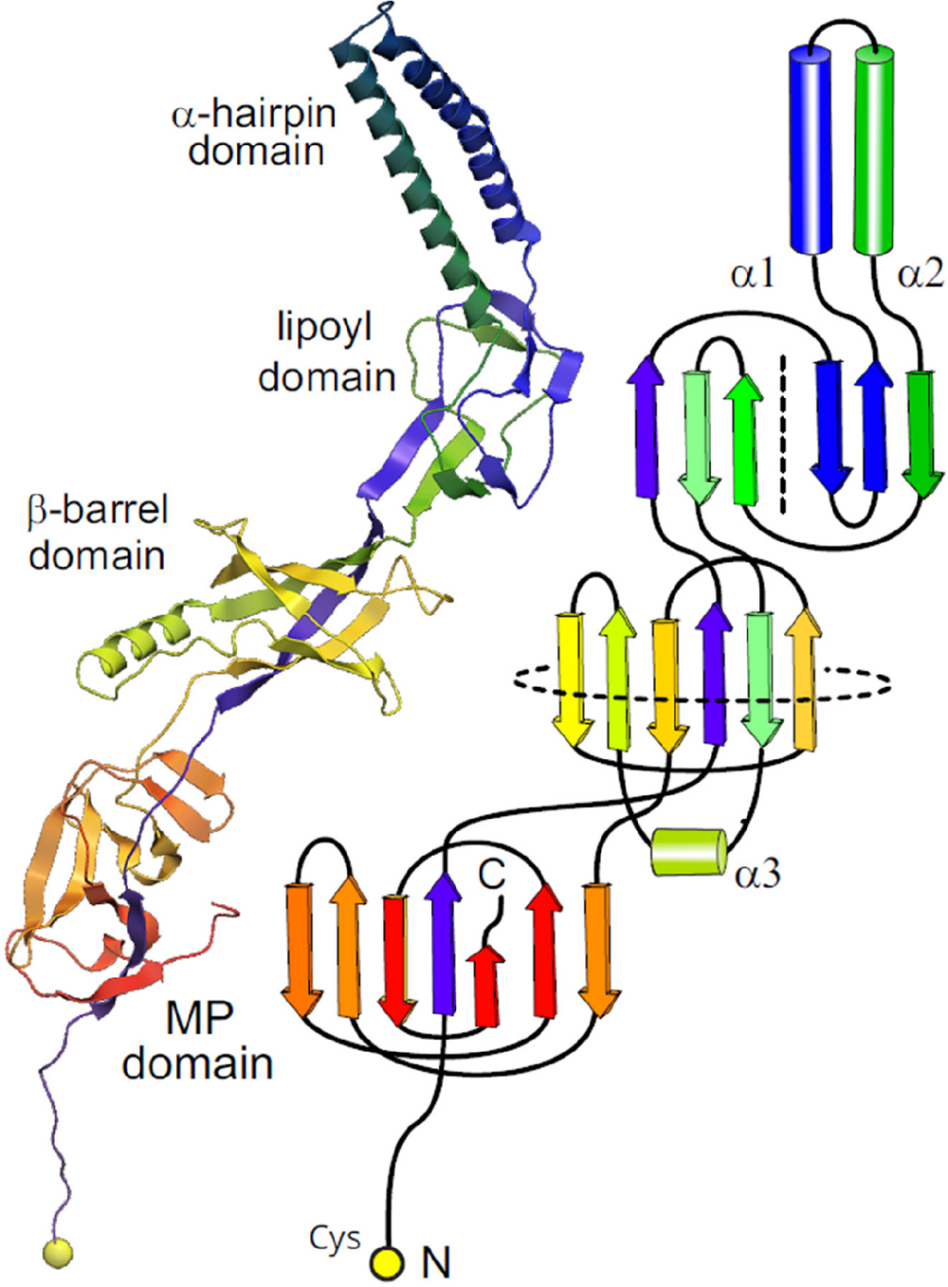
Structural organization of a typical PAP based on the structure of the MexA from *Pseudomonas aeruginosa* (PDB ID 6TA5).^270^ The topological organization of the principal PAP domains is shown on the right. The polypeptide chain of the PAP has a form with a hairpin-like fold with both N- and C-termini being close to the membrane and both the N- and C-terminal parts of the protein contributing to each domain. The N-terminal cys-residue that is lipidated in the RND-associated PAPs is highlighted in yellow.

In addition, the structures of some aberrant PAPs have become available, such as the PAP BesA from the Lyme disease associated spirochete *Borrelia burgdorferi* (PDB ID 4KKS-4KKU),^703^ which lacks the helical hairpin domain altogether. In addition, some orphan PAP structures could also be found in the database, as a result of structural genomics efforts, e.g., BACEGG_01895) from a putative efflux pump from *Bacteroides eggerthii* DSM 20697 (PDB ID 4L8J); however, its association with HAE-1 or HME pumps remains to be established. Figure 34 presents the organization of a typical PAP on the example of the MexA which functions with the RND transporter MexB and the OMF OprM in *P. aeruginosa*.^704^

In the paragraphs below, we will “walk” along the structure of the typical PAP from the N-terminal to the C-terminal end, describing briefly the central features and the domains involved in building its unique architecture.

Many RND transporter associated PAPs in their mature form, following processing by signal peptidase 2, are modified by an N-terminal cysteine lipidation (e.g., triacylation or in the case of AcrA, S-palmitoylation), which anchors the protein to the inner membrane. The role of this anchorage is not fully clear, as truncated delipidated AcrA carrying a His-tag at the C-terminal is functional and has been used for biochemical studies.^740^

The PAPs associated with ABC and MFS transporters, as well as those involved in T1SSs, are typically anchored to the inner membrane by an N-terminal transmembrane helix and have sizable cytoplasmic domains;^726^ however, very little is known of their structure at present. These regions, however, are essential for correct substrate recognition and complex assembly.^206^

### 7.3 Domain Organization of the PAPs Associated with RND and MacB-Assemblies

The core domains, which are found in all RND-associated PAPs, including MexA, present a beads-on-a-string arrangement; starting with the closest to the membrane are the membrane proximal domain (MPD), the β-barrel domain, the lipoyl domain, and the α-hairpin (coiled-coil) domain. Some modules are universal, while others are only shared within a subset of the family, and this arrangement correlates with the type of cognate transporter with which the PAP functions^726^

The closest domain to the membrane, as the name suggests, is the membrane proximal domain, abbreviated from here on as MPD. It is flexibly linked to the core of the protein and is composed of both N-terminal and C-terminal elements. Its N-terminal β-strand provides the direct link to the inner membrane in the TM-containing PAPs, such as those associated with the T1SS, MacB, and MFS-type transporters. The rest of the domain is composed of the C-terminal part of the protein and presents a β-roll domain topology^716^ loosely related to the downstream β-barrel domain, from which it may have originated via an early gene duplication event.

Notably MPDs are missing in the PAPs operating with transporters that lack periplasmic domains, suggesting that their function could be associated with supporting the periplasmic assembly and substrate selection and presentation.^726^ This role has been clearly demonstrated for PAPs associated with HME RND transporters, such as CusB,^360,741^ and hinted at by mutagenesis and transporter studies of chimeric HAE-1 transporter assemblies.^717,742^ Furthermore, MPDs are essential for the activation of ATPase function in the MacB-class transporters,^573,743^ and they have been associated with substrate binding as will be discussed further in the context of their tripartite assembly. A possible break from the rule is observed in the specialized MacB-like transporter DevCA and related pumps in *Anabaena*, the PAPs of which (e.g., DevB), appear not to possess MPDs.^206^

The MP-domain of RND-associated PAPs was shown to be essential for the assembly and function of the AcrAB-TolC in *E. coli*(744) and more recently, in a range of *Salmonella* PAPs.^742^ In RND-based assemblies, PAPs play a significant role in stimulation of the efflux activity and consumption of the proton gradient as exemplified by the liposome-reconstituted studies of AcrAB^706^ and MexA–MexB studies.^707^ While in both cases the pump activity was increased in the presence of the cognate PAP, the MexA dramatically increased the activity of MexB only when the substrate was also present, suggesting that substrate engagement plays a significant role in driving the assembly.

The next domain up from the membrane is the self-explanatorily named β-barrel domain, which presents a β-barrel consisting of six antiparallel β-strands capped by a single α-helix^733,734^ (Figure 34). The β-barrel domain presents similarity to enzymatic ligand-binding domains such as the flavin adenine nucleotide-binding domain of flavodoxin reductase and ribokinase and also to domains with odorant-binding properties. The roles of this domain appear to be predominantly connected with self-association of the PAPs into hexameric assemblies,^737^ as well as in forming contacts with the periplasmic domains of respective transporters.^272,287,359^

Among the MacA-family PAPs the residues equivalent to the *Ec*MacA E231, Y276, and T293 residing within the β-barrel domain are strictly conserved. These three residues are involved in the interactions between the β-barrel domains, forming a closed six-membered ring in head-to-tail fashion.^737^ Notably equivalent residues are conserved in PAPs from Gram-positive organisms, e.g., the *B*. *amyloliquefaciens* YknX and Spr0693,^571,594^ and similar conservation of equivalent residues in RND-associated PAPs is also observed, highlighting the functional importance of this hexamerization.

At least in some subsets of PAPs, e.g., HME-associated ZneB, the β-barrel domain also participates in ligand binding.^46^ We will discuss these functions further in the context of the specific tripartite assemblies in the sections below.

**Figure 35:**
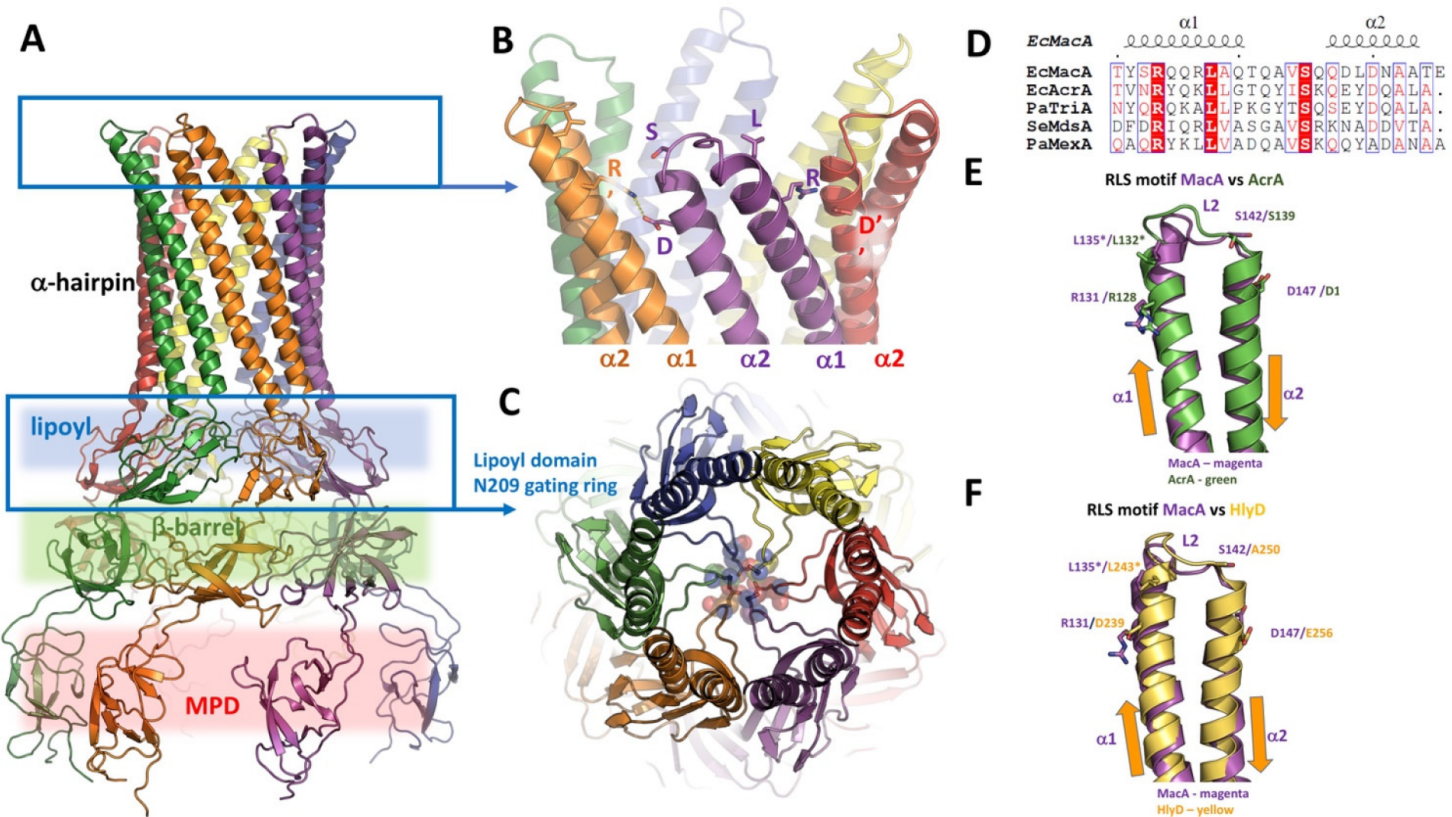
Hexamerization of PAPs and the formation of the helical–hairpin nanotube. RLS-motif of PAPs and its conservation across different groups. (A) General view of the MacA hexamer from PDB ID 5NIK, highlighting principal domains. (B) Close view of the hairpin domains presenting the RLS signature motif. (C) Top-down view of the lipoyl-ring of MacA as seen in PDB ID 3FPP.^737^ The N209 from the “gating ring” is restricting the channel in the hexameric form. (D) Canonic RLS-motif as seen in RND- and MacB-associating PAPs. (E) Overlay of the tips of the hairpin domains of MacA and AcrA showing close conservation of the RLS-motif. (F) Overlay of the tips of the hairpin domains of MacA and T1SS-associated HlyD shows deviation from the canonic sequence.

The next domain up from the β-barrel domain is the lipoyl domain, named due to the homology it exhibits to the biotinyl/lipoyl-carrier domains found in dehydrogenase enzymes.^733,745^ These domains consist of a β-sandwich of two interlocking motifs, each consisting of three β-strands. The role of these domains also appears to be in stabilizing the oligomeric self-assembly of the PAPs, and mutations targeting the domain have a destabilizing effect, often obliterating function altogether.^206^ However, unlike the β-barrel domains, the lipoyl domains do not appear to provide contacts with the transporters, at least in the studied RND and MacB assemblies.^272,305,359^ The lipoyl domains also provide substrate-vetting and, at least in the case of MacB-based systems, the lipoyl-domains of the MacA-family PAPs create a “gating ring” restricting access to the α-helical tunnel formed by their hexamerised hairpin domains, which is suggested to prevent backflow of substrates^272,737^ (Figure 35, panel D).

The most-distant from the inner membrane and facing toward the OMF is the α-hairpin domain, which in its “canonical” form presents an antiparallel coiled-coil, formed of two helical fragments, divided in the middle by a loop. The distal end of this hairpin is suggested to be responsible for the interaction with the reciprocal coiled-coils of the OMF; however, the level of this interaction is either extensive, by forming four-helical bundles in the so-called “deep-interpenetration” or “wrapping-up” model,^649,716^ or strictly restricted to the tip of the hairpin and the loop that connects the two helices in the so-called “cogwheel” or “tip-to-tip” model.^737,746,747^ Nestled within the lipoyl domain, the α-hairpin domain is an extension from the very loop that contains the lysine that undergoes lipoylation (covalent attachment of lipoamide, via an amide bond) in the corresponding domain in the dehydrogenase subunits.

The α-helical hairpin domains of the “canonical” PAPs associated with RND assemblies, such as AcrA and the MacB-associated MacA, resemble inverted versions of the helical hairpins forming the structural repeats within the OMF.^716^ Indeed, the Cα-backbone of the PAP α-helical hairpin domain can be superimposed on both coiled-coils of TolC, when inverted and viewed in an equivalent orientation. The coiled-coils within this group of PAPs are formed upon a classical heptad repeat (HxxHCxC) principle, where H is a hydrophobic and C is a charged residue,^748,749^ and they contain a variable number of repeats. The heptad is also usually depicted as abcdefg, with the core of the hairpin being formed by the residues in the a and d-positions, which are conserved throughout the PAP family and contain bulky hydrophobics, while the exposed helical faces, directly opposite the core of the hairpin are also conserved, with hydrophilic serine/glutamate occupying the c-position, and alanine predominantly found in the f-position^733^ (Figure 35 and connected figures in section 8.2). The hairpin domains show considerable diversity in organization, differing in length from four heptad repeats per helix in MexA to 11 heptad repeats in EmrA;^738^ however, there are some aberrant forms, such as the metal-efflux pump associated CusB,^702^ which features a folded hairpin, with only 2 heptad repeats in its longest helices, as well as the spirochetal BesA,^703^ which lacks an α-helical extension altogether.

Sequence alignment of the distal part of the hairpin domains of the canonical PAPs reveals high conservation of the tip-region of α1 and α2-helices, presenting a consensus sequence RxxxLxxxxxxS/T, which became known as the RLS-motif,^737,750−753^ which has been implicated in OMF binding, which was subsequently confirmed by the cryo-EM structure of the complete assembles. The leucine of the RLS-motif is the most conserved residue within PAPs, mediating strong tip-to-tip interactions between PAPs and OMFs. However, the last position of the motif shows higher variability and can be occupied by a serine or a threonine residue (Figure 35).

The role of the PAP hairpin-domain may not be limited to the RLS-based pairing with the OMF, as witnessed by a number of suppressor and compensatory gain-of-function mutations which map to the stalk of the hairpin.^709,711,717,754,755^ The structures of the MacAB-like transport complex from Gram-positive bacteria^571^ composed of the ABC transporter Spr0694–0695 and the associated PAP Spr0693 (UniProt ID: Q8DQF9) revealed that despite the strikingly similar architecture to that of the MacAB complexes seen in the Gram-negative bacteria and notable preservation of the hexameric tube-like assembly of PAPs creating a tight gasket-like structure over the transporter, the tips α-helical hairpin is mobile and disordered in the structure. Consistent with a lack of OMF-association, the tips of the Gram-positive PAPs examined to date show little conservation and no obvious RLS-like motif.^571,594,725^ These findings highlight the functional importance of PAP–hairpin domain oligomerization. However, they also pose a question regarding their role in these organisms, and while the definitive answer to that is still not known, an increasing amount of circumstantial evidence suggests that they are involved in peptidoglycan binding;^756^ for example, both AcrA and YknX were found to bind to peptidoglycan.^594,751^ Notably, *E. coli*, but not *S. aureus* peptidoglycan was found to facilitate AcrA-TolC interaction.^751^ Furthermore, the reconstruction of the assembled AcrAB dimers from the *in situ* cryo-tomography of AcrAB-TolC complexes is also compatible with the hairpin of the PAP being in direct contact with the PG layer.^307^

The α-helical hairpin of PAPs of the EmrA-family^738^ and T1SS-associated PAPs such as HlyD^739^ features an “extension subdomain”, representing a departure from the classical heptad packing, which can be seen as an additional helical insertion into the proximal part of the hairpin, which is highly similar to the untwisted pairs of α-helices in the OMF α-barrel.^726^

The coiled coils of the α-hairpin domain show significant flexibility and propensity for self-association, which has been linked to their functional role.^735,736,757,758^ Apart from soluble dimer and trimer formation that could be detected in the periplasm, a particular subset of PAPs form hexameric tubular structures, which match remarkably well the outer diameter of the OMF channel.^573,737,759^ The stability of these oligomeric assemblies differs between different PAP types and generally correlates with the length of the hairpin domain, resulting in a differential affinity to the OMF.^697^ These differences in hairpin domain organization appear to be associated with the different modes of engagement of the PAPs with their respective transporters, and we will discuss in further detail the context of specific assemblies, but here we will highlight some specifics of the PAPs participating in T1SS and MFS transporter assemblies.

### 7.4 PAPs Associated with MFS Transporters and T1SS

Unlike the “canonical” group of PAPs discussed above, which function with transporters that feature large periplasmic domains and only shuttle cargoes from the periplasm, the PAPs from this group associate with transporters that transfer cargoes directly from the cytoplasm and lack pronounced periplasmic domains. Correspondingly, these PAPs present a different architecture, as they face dual challenges—on one hand they have to provide extensive sealing of the relatively small transporters to avoid leakage of substrate in the periplasm, and on the other hand, they are required to reach further out into the periplasm to bridge the OMF as they are located much closer to the inner membrane.

**Figure 36:**
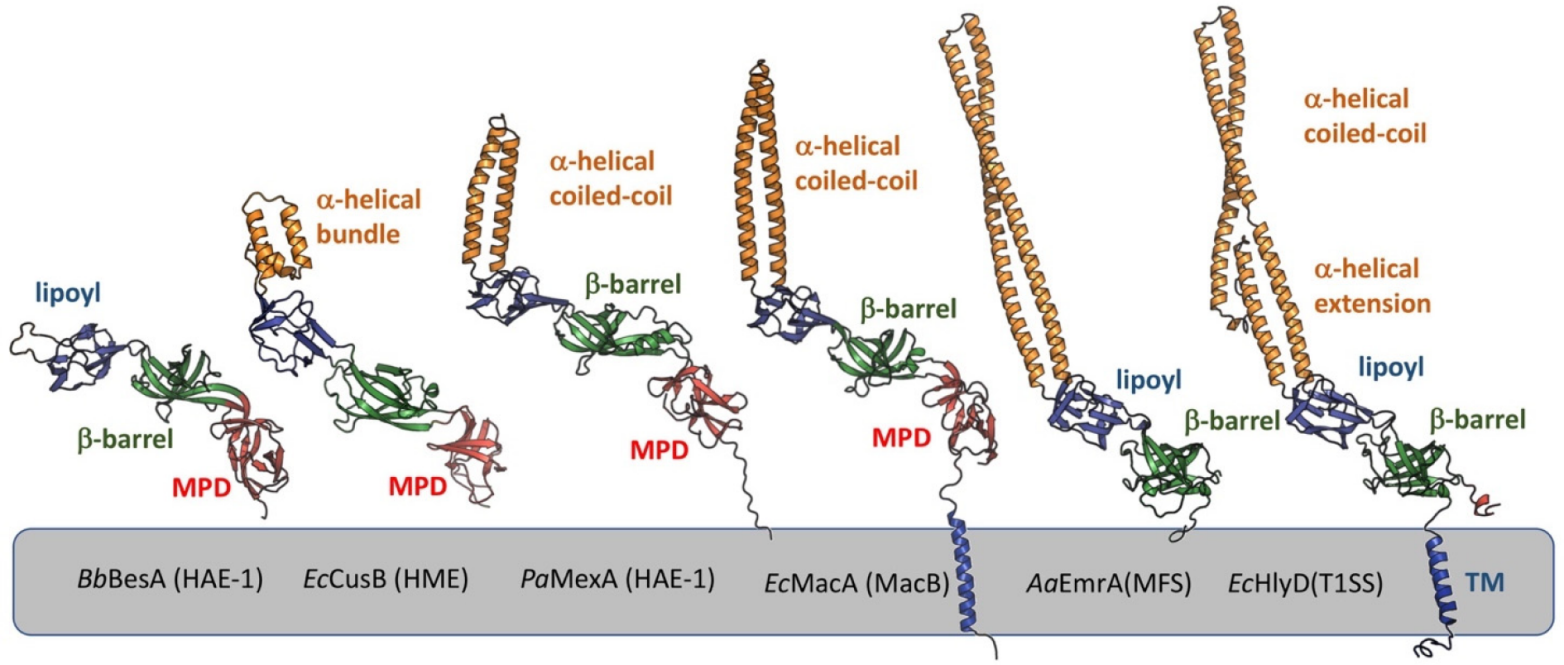
Different types of PAPs associated with different efflux systems. Comparative structural gallery of PAPs of known 3D structure, colored by domain. Membrane proximal domain (MPD) in red; β-barrel domain in green; lipoyl in blue; α-helical-hairpin in gold. From left to right: BesA (PDB ID 4KKS); HME-associated CusB (PDB ID 3OW7); HAE-1 associated MexA (PDB ID 6TA6); MacB-associated MacA (PDB ID 5NIK); MFS-associated *Aa*EmrA (PDB ID 4TKO); and T1SS-associated HlyD (PDB ID 5C21). The missing loops and transmembrane fragments of MacA/EmrA and HlyD have been modeled for illustrative purposes. Note that only RND- and MacB-associated PAPs appear to contain MPDs, while the spirochaetal BesA lacks the α-helical–hairpin domain altogether.

*Notably, PAPs from this group lack a MPD domain and present a very long helical extension although they have slightly different solutions to the common problem discussed above (*Figure 36*). The MFS-associated group is exemplified by the structure of the* EmrA *from the hyperthermophile Aquifex aeolicus* (*Aa*EmrA from here on).^738^ The lipoyl and β-barrel domains are rather conserved and match those seen in the rest of the family, with the exception of a notable loop (*P*321-V343) in the β-barrel domain, which is both unique to and highly conserved across the EmrA family, suggesting it may play an important functional role. The periplasmic part of the protein binds the transported drugs, and in addition, the *E. coli* homologue, *Ec*EmrA, is anchored in the membrane by an N-terminal helix, which contains a leucine-zipper-like dimerization domain and is involved in oligomerization and transporter association of the PAP.^760^

The crystal structure of the *Aa*EmrA revealed an antiparallel, two-stranded, α-helical coiled-coil domain of 168 residues and 127 Å long with 11 heptad repeats per helix. Thus, the hairpin-domain in *Aa*EmrA is over twice the length of that in RND-type PAPs, e.g., MexA and AcrA (47 and 58 Å, respectively), although in its *E. coli* counterpart *Ec*EmrA, that is estimated to be around 80 Å in length^738^ (Figure 36). Notably, the coiled-coil in EmrA appears nonideal due to disruption in packing caused by positively charged residues in its hydrophobic core, i.e., K84, K98, R130, R182, and K119 which occupy the normally hydrophobic a and d heptad positions. In addition, the heptad repeat pattern is disrupted by a four-residue insertion in the N-terminal helix (residues 112–115) and a three-residue insertion in the C-terminal helix (residues 210–212), approximately 70 Å from the tip of the structure and close to the center of the coiled-coil.^738^ These insertions disrupt the knobs-into-holes packing, resulting in a weakened interhelical interaction in this region of the coil. Heptad shifts, known as “‘stutters’” and “‘stammers’”, are commonly found in fibrous proteins with an extended coiled-coil structure,^761^ and it has been long hypothesized that such regions serve as points of flexibility for extremely long coiled-coils.^762,763^

### 7.5 T1SS Associated PAPs

Similar to the PAPs associated with the MFS assemblies, the structure of the representative T1SS-associated PAP, namely *E. coli* HlyD (referred to as *Ec*HlyD henceforth) from the α-hemolysin secretion system, presents an architecture lacking an MPD and is characterized by an oversized α-helical hairpin domain measuring ∼115 Å in length, thus being second only to the MFS-associated EmrA, which is almost 130 Å long (PDB ID 5C21-5C2).^739^

Similar to *Aa*EmrA discussed above, the α-helical hairpin domain of *Ec*HlyD is divided roughly in the middle, into an N-terminal and C-terminal part, by a loop at its distal tip (L2), which is supposed to contact the OMF. Unlike the MFS-associated PAPs, however, which have two continuous α-helices forming each half of the α-hairpin domain, the HlyD hairpin presents a complex, discontinuous architecture, which deviates from the standard coiled-coils observed in the classical RND-associated transporters and is composed of three helical fragments (α1 to α3), linked by two connecting loops L1 and L2.^739^

While the C-terminal part of the hairpin domain is formed by a continuous α3-helix (residues 251–325), which runs the entire length of the hairpin, the N-terminal part of the hairpin domain is discontinuous and formed by two shorter helices, α1 and α2, connected by the long and flexible loop L2. While the exact role of the long loop L1 is unclear, it provides a hingelike arrangement for the α1- and α2-helices, allowing them to form independent antiparallel-coiled coils with the α3, and such an arrangement may purposefully allow for allosterically controlled destabilization of the PAP α-barrel, providing greater flexibility, and it may facilitate the disassembly of what otherwise may be a too-rigid conduit. Thus, the breakage of helices observed in the T1SS-associated HlyD likely serves the same purpose as the nonheptad insertions in the EmrA, allowing to alleviate structural stress, and furthermore, helical breaks allow the helical trajectories to be corrected, providing both more flexible lateral packing and easier disassembly of the complex. Intriguingly, a similar solution to the cladding of the alpha-barrel problem is presented by the OMF-proteins, the inverted repeats of which follow the same discontinuous hairpin arrangement.

Oligomerization of HlyD appears to be prerequisite for TolC recruitment,^492^ and consistent with that forced hexamerization of a HlyD fragment (covering 81–383) fused to an Hfq-hexamer ring was able to recruit a TolC-MacA chimeric protein, while the same HlyD fragment was unstable on its own and was not capable of TolC-MacA binding.^739^ Indeed, formation of a helical bundle via lateral oligomerization, similar to those observed for MacA and AcrA, has been suggested based on the crystal structure of the hairpin and lipoyl domain of HlyD,^739^ and modeling of hexamer creates a plausible PAP assembly, with an internal diameter of ∼40 Å. Providing support to the plausibility of the model, packing of the helices within the model brings into proximity two critically important residues, mutation of which abolishes HlyD function *in vivo*, as established from functional studies (R186 from α2-helix and D309 from α3-helix).^752^ Superposition of the distal parts of the α-helical hairpins of HlyD and MacA provides a good structural match (Figure 35E); however, the RLS-motif is misaligned relative to the earlier sequence-alignment-based predictions, which designated the R186, L190, and T197 as the signature triplet.^752^

Furthermore, the RLS-motif as positioned at the L2-loop appears to be significantly degenerated relative to the other members of the PAP family which interact with TolC and present a sequence which in the *Ec*HlyD is formed of D238, L243, and A250. The lack of RLS conservation suggests higher promiscuity at the tip of the assembly than might be expected by the limited interface that it presents. This unique organization of the hairpin in T1SS-PAPs has also been confirmed from the structure of the *Serratia marcescens* LipC (PDB ID 5NEN), a PAP that forms a T1SS with the ATPase LipB and the OMF LipD in the transport of the lipase cargo-protein.^514^ The “RLS” sequence of the LipC is further divergent from the canonical one, with S183 (in place of R128 AcrA/D239 HlyD respectively); L187 (L132/L243 being the only fully conserved residue within the motif); P194 (S139/A250); and E200 (D144/E256) in place.

### 7.6 PAPs Associated with the Heavy Metal Efflux (HME) Family

PAPs associated with the heavy metal efflux (HME) family of RND transporters often contain additional N- and C-terminal domains, e.g., SilB from *Cupriavidus metallidurans* CH34,^764^ the C-terminal domain of which has been shown to provide metal-chaperoning function. These domains have arisen from gene-fusions with metal-binding proteins and could also be found as stand-alone polypeptides (e.g., CusF of *E. coli; PBD ID 3E6Z*), which display a unique metal-binding β-barrel fold.^765,766^

CusB protein is part of the CusCBA periplasmic Cu^+^/Ag^+^ efflux system in Gram-negative bacteria,^767^ and it was the first HME-associated PAP whose structure was solved (PDB ID: 3H9I)^702^ and presented several unexpected structural features; notably, its hairpin domain was significantly shorter than the ones of “canonical” HAE-1 associated PAPs and was folded into an antiparallel, three-helix bundle. In addition, CusB was found to directly bind to Cu^+^ ions via an intramolecular relay of conserved methionine residues (e.g., M49, M64, M66, and M311), undergoing a conformational transition, implicated in the transport cycle of the pump.^768−770^

The structure of ZneB (PDB ID 3LNN), a PAP associating with the second branch of HME transporters, responsible for efflux of divalent cations, has also been solved; it presented an architecture very similar to the canonical HAE-1 PAPs.^46^ Similar to CusB, it also has a specific metal-binding site, which however is much more localized and located in the junction between the β-barrel and the MP-domain. While the PAP ZneB presents an architecture very similar to the PAPs associated with the HAE-1 type RND transporters, it also acts as a specific metal binding site, and the crystal structure revealed Zn^2+^ ion coordinated by H220, H284, E328, and a water molecule at the interface between the β-barrel and the MPD domain.^46^

The demonstration of active substrate-binding and presentation function, as exemplified by the metal-pump associated PAPs, showed that the PAP-proteins are more than just passive adaptors and has shifted the attention to their active role in organization of the tripartite assemblies, which we will discuss in the following paragraphs for each of the main systems.

### 7.7 Fellowship of the Rings—The Role of PAP β-Barrel Domains in Assembly and Processivity of Tripartite Efflux Pumps

**Figure 37:**
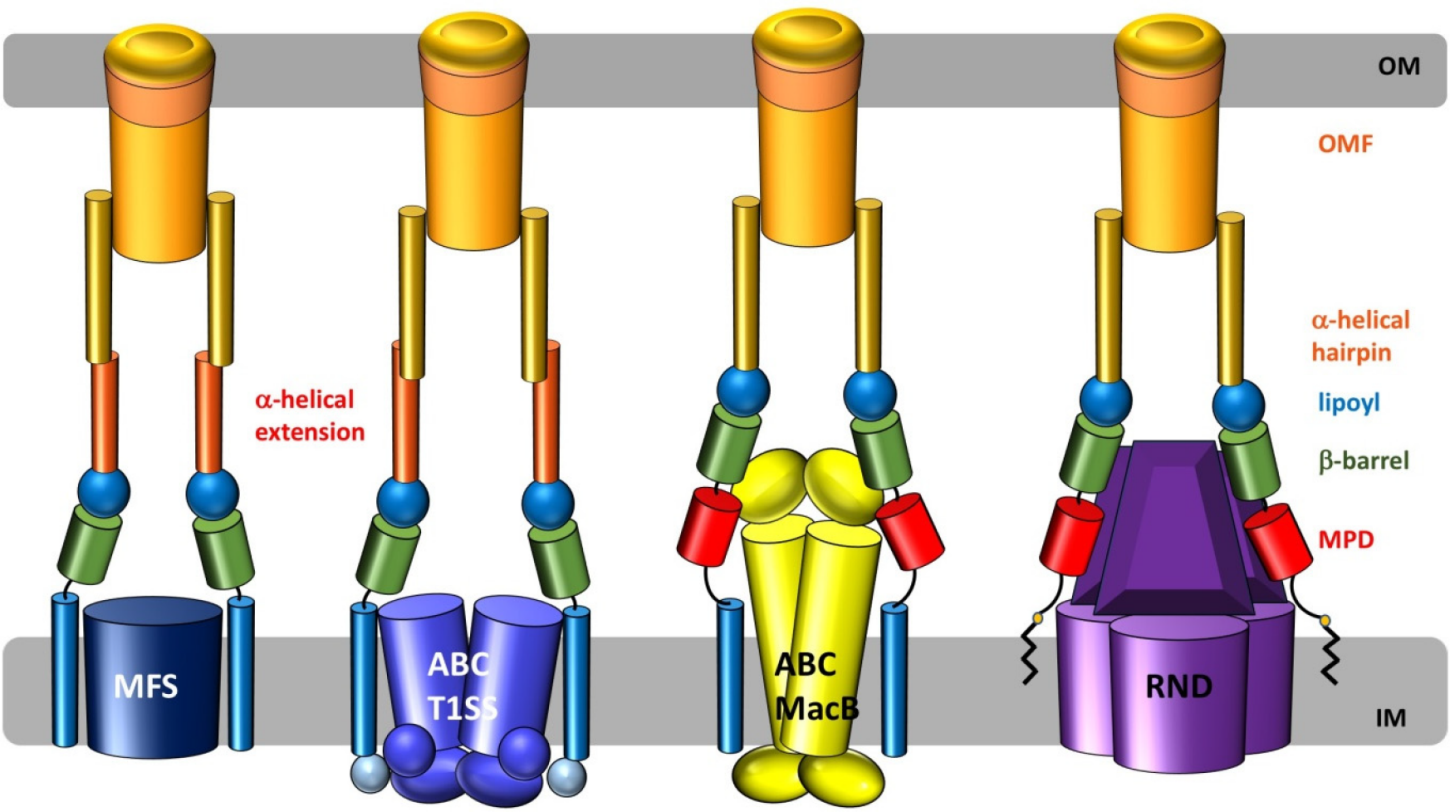
Modularity of the assembly of tripartite complexes. A schematic representation of the different tripartite assemblies based on their transporter types reveals striking correlation with the domain composition of the PAP proteins associated with them. PAPs of the MFS- and T1SS ABC transporter-based complexes lack MP domains, which is also correlated to the transport of their cargoes across the inner membrane (IM). On the other hand, the MacB and RND transporter-based assemblies pick their cargoes from the periplasmic side of the membrane associated with PAPs that have MPDs, hinting toward their role in presentation of the cargo to the transporters. Notably, the β-barrel domains of the PAPs are universally present and allow adaptation to diverse transporter stoichiometries.

In the preceding sections we described a wide variety of transporter families, PAPs and OMFs, which combine to create a motley crew of tripartite assemblies. This leads to the logical question, as to what the unifying feature of these assemblies is and what brings them under the same umbrella. Linked to the common use of the OMFs, the unifying feature of these assemblies is the use of the PAP-family for bridging the transporters. As noted previously,^726^ the domain utilization of the PAPs is associated with specific transporter families (Figure 37), with the presence of MP domains being restricted to transporters which have large periplasmic domains, i.e. the MacB- and RND-families, which correspondingly take their cargoes predominantly from the periplasm.

The examination of the remaining tripartite complexes suggests that the PAP domains minimally required for forming a functional assembly are the β-barrel and lipoyl domains, both of which have been shown to form homooligomeric assemblies. Structures of binary PAP–transporter complexes^610^ and fully reconstituted pumps^9,272,305^ indicate that while lipoyl domains never contact the transporter, the β-barrel domains form a critical hexameric ring-seal around the transporter in the periplasm, which is a defining structure of the tripartite assemblies, shared across all transporter types.^726^ This gasket-like assembly allows seamless adaptation of the different transporter stoichiometries and sizes to the trimeric OMF and has some unique features, which we will discuss below.

The PAP β-barrel domain shares the topology of the barrel seen in ribokinase enzymes and lipid-binding proteins,^733^ and our previous work linked the β-barrel domains of the PAPs to the ribokinase-like domains seen in some flagellar basal-body associated proteins.^726^ The bacterial flagellar motor consists of a number of conserved components which include the inner-membrane spanning MS-ring; a cytoplasmic C-ring and its associated export apparatus; a periplasm-and-outer membrane spanning rod structure; and associated with the latter, the periplasmic (P-) and outer-membrane embedded L-rings; plus, the optional H- and T-rings which are present in some organisms such as *Vibrio*.^771^ The C-terminal domain of the flagellar protein FlgT (FlgT-C; residues 256–356),^772^ which associates with the outer membrane H- and T-rings in *Vibrio*, is comprised of a six-stranded β-barrel, nearly identical to the N-terminal domain of the β-subunit of F_1_-ATPase (e.g., bovine mitochondrial F1-ATPase PDB ID 1BMF),^773^ the catalytic subunit of the ATP-synthase complex.^772^

**Figure 38:**
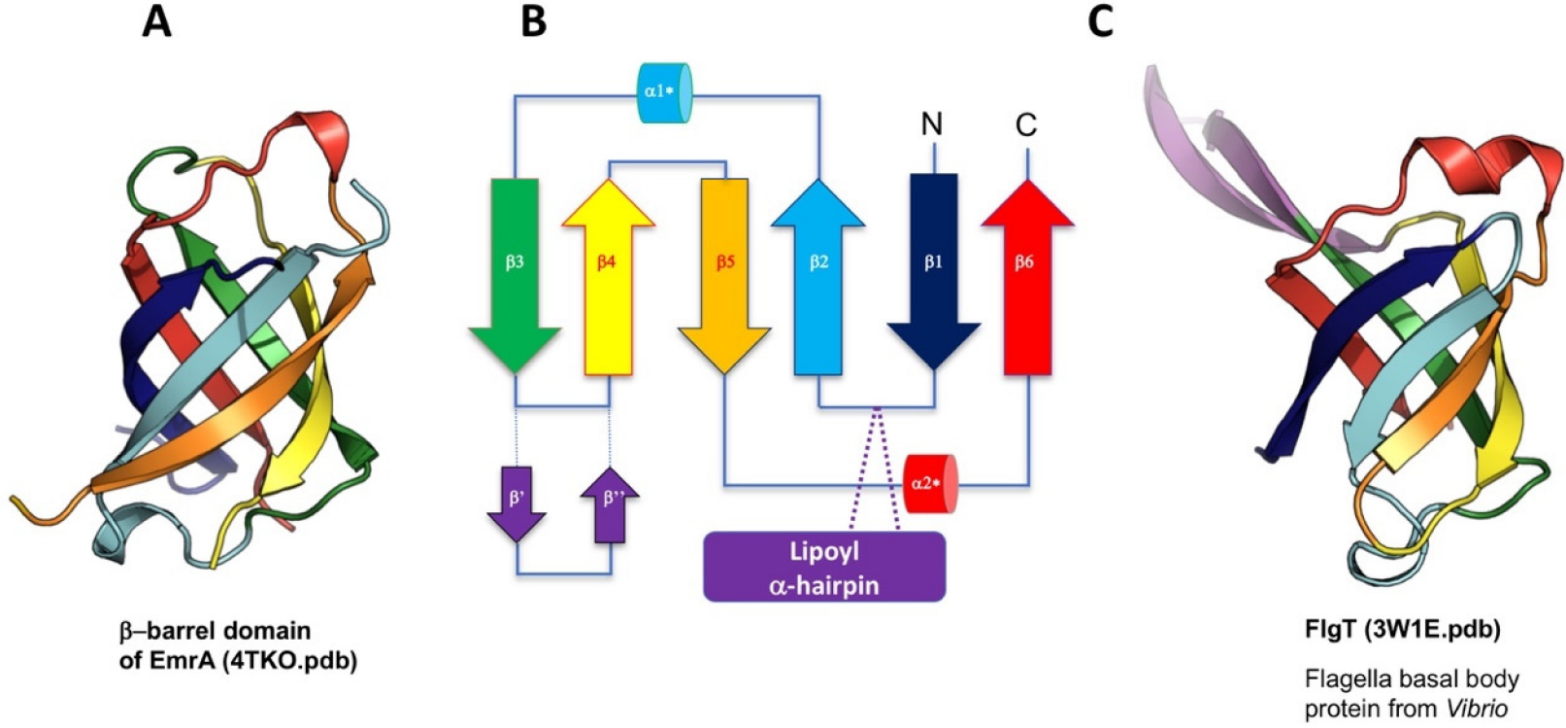
Structural organization of the PAP β-barrel domains links them with β-barrel domains in flagellar basal body proteins. (A) Structure of the isolated β-barrel domain of the MFS-associated PAP EmrA, based on PDB ID 4TKO. (B) Topological diagram. The lipoyl and α-hairpin domains can be seen as inserted between the β1- and β2-strands of the β-barrel. (C) Structure of the C-terminal domain of the flagellar basal body protein FlgT from *Vibrio*.

This topology is strikingly similar to that observed in the PAP β-barrel domains (Figure 38), which only differ by the lipoyl domain being spliced into the loop connecting the β1-strand and β2-strand forming the core of the barrel.^726^ Consistent with that, despite lacking discernible sequence homology, FlgT-C superposes on the Cα-backbone of the β-barrel domain of EmrA (PDB ID 4TKO), with an RMDS of ∼3 Å.^726^ Our new expanded analysis, provided below, expands this association of PAP β-barrel domains across a number of the type III secretion system proteins as well, including the cytoplasmic flagellar assembly export apparatus.

Away from the outer membrane, the cytoplasmic *flagellar type III export apparatus* used for the secretion of the flagellar components consists of a transmembrane PMF-driven export gate and a cytoplasmic ATPase complex composed of FliH, FliI, and FliJ.^774^ There, the FliI is a Walker-type, homohexameric rotary-ATPase, oligomerization of which is required for ATP-hydrolysis;^775^ FliJ is the stalk unit, and the FliH is a regulatory/stator subunit.^776^ This system is also evolutionarily connected to the virulence associated *“injectisome”* apparatus,^777,778^ e.g. the one coded by the *Salmonella* pathogenicity island 1 (SPI-1), where the corresponding components in the so-called “sorting platform”, forming the base of the SPI-1 injectisome, are the ATPase InvC (aka SctN under the unified nomenclature),^779^ its stalk subunit InvI (SctO), and the regulatory/stator subunit OrgB (SctL).^780^

The FliI_6_FliJ complex was shown to be structurally similar to the α_3_β_3_γ complex of F_1_F_0_-ATPase,^781,782^ with the N-terminal domains in the FliI-type ATPases being highly homologous to the N-terminal domains of the F1/V1-ATPases (e.g., bovine mitochondrial F1-ATPase (PDB ID 1BMF)^773^ or the *E. coli* F1F0-ATPase (PDB ID 6OQR)),^783^ in agreement with earlier bioinformatic predictions.^784,785^ This has been further confirmed by the recent cryo-EM structure of the homohexameric T3SS ATPase EscNin complex with its central stalk subunit EscO,^786^ which revealed a rotary catalytic mechanism analogous to that of the heterohexameric F1/V1-ATPases despite its homohexameric nature and, furthermore, elucidated the N-terminal domain of the EscN (residues 35–101) (PDB ID 6NJO; 6NJP), which was missing in the earlier structures. Comparison of these newly resolved N-terminal domains of the InvC-class ATPases, with those belonging to the FliI-type, shows that they are closely connected to each other (overlaying with an RMSD of ∼1 Å over the Cα backbone) and form a closed β-barrel with Greek-key topology.

**Figure 39:**
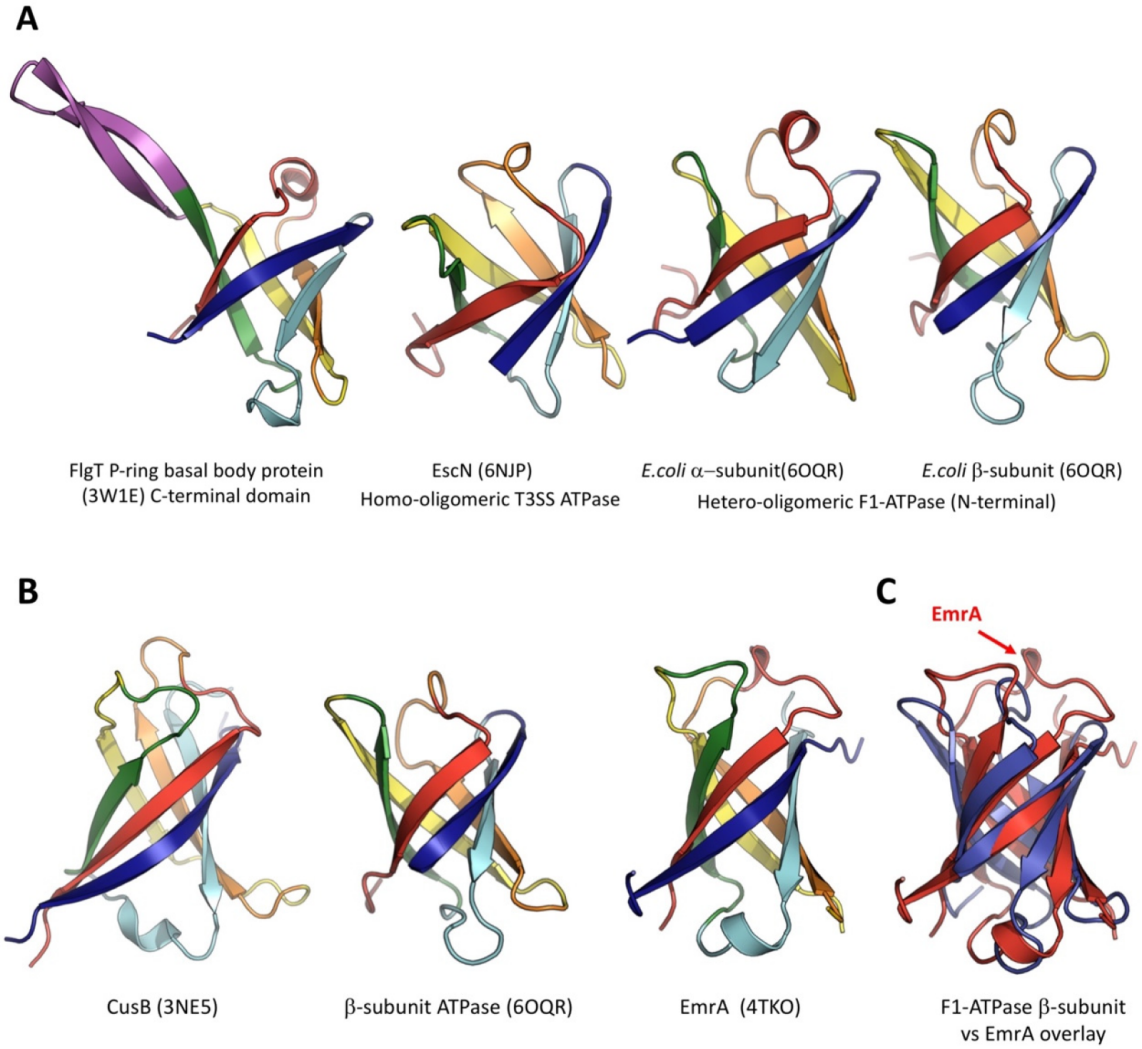
(A) Structural connections of the β-barrel domains in the rotary ATPases and the T3SS-associated ATPases. (B) The β-barrel domains of PAPs structurally homologous to those found in the rotary ATPases. Secondary structure elements are color coded identically to the topological diagram in Figure 38. (C) Overlay of the C-alpha backbone between EmrA and F1-ATPase β-subunit highlights their close structural homology.

Startlingly, these N-terminal β-barrel domains of the F1/V1- (e.g., the β-subunit of F1-ATPase PDB ID 6OQR (residues 1–76))^783^ and T3SS-associated ATPases (e.g., FliI residues 25–97 (PBD ID 2DPY))^781^ are also closely connected to the β-barrel domain of the PAPs, presenting an equivalent topology to the one discussed above for the FlgT but lacking the additional β-hairpin insert between β3 and β4 observed in the latter (Figure 39; color scheme is the same as Figure 38).

**Figure 40:**
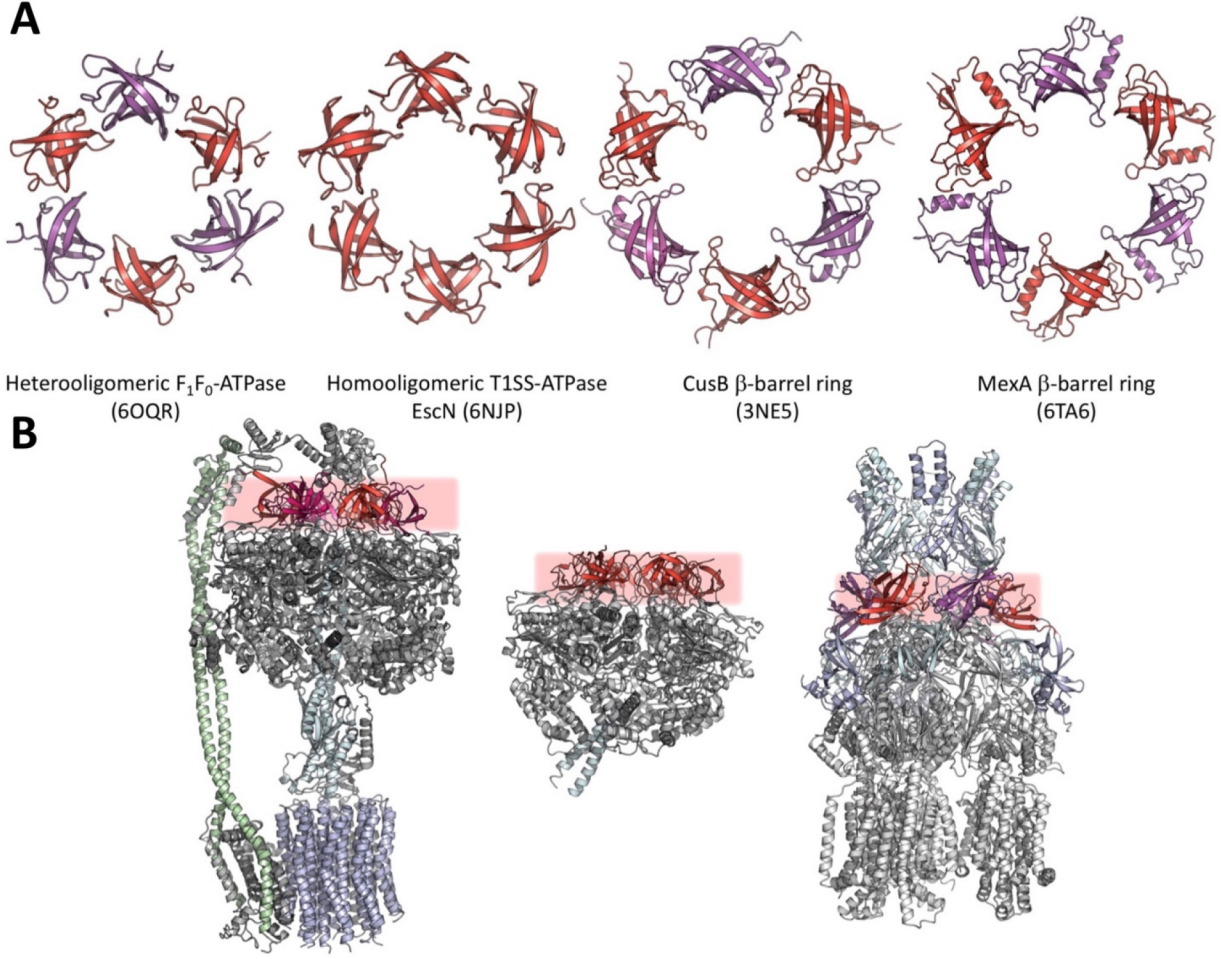
Connection between β-barrel domains and rotary ATPases. (A) Upper panel. A view of the hexameric assemblies presented by the rotary ATPases and PAP β-barrel domains. (B) Left, side view of a hetero-oliomeric F1F0-ATPase from *E. coli*; Center, type 3 secretion system associated ATPase EscN from *E. coli*. Right, CusBA subcomplex from *E. coli.* The hexameric β-barrel rings are highlighted by the red parallelogram, with individual subunits being red and magenta.

Within the ATPase assemblies, the N-terminal β-barrel domains form a head-to-tail hexameric ring assembly, which could be formed by alternating α- and β-subunits, e.g., in the case of F1-ATPases,^773,783^ or can be presented by a homohexameric ring in the case of T3SS ATPases, e.g. EscN.^786^ As seen in Figure 40, panel A, these rings share a striking resemblance to the ones observed in RND, MacB, PCAT, and MFS assemblies and are maintained by conserved residues as discussed in the preceding PAP section.^571,737^

In the type III SS associated ATPases, the N-terminal domain is essential for stable assembly of higher oligomers and activation of the ATPases;^776,787^ it interacts with effector proteins (e.g., the *Salmonella* negative regulator of the ATPase OrgB)^788^ and has intrinsic phospholipid binding affinity.^789^ Similarly, in the N-terminal β-barrel domains of F_1_F_0_, ATPases have been shown to play a role in stabilization of the complex^790^ and providing contact of stator subunits b and delta.^783^

Extending the functional analogy, mutations targeting the β-barrel of PAPs impact complex formation and can be detrimental to assembly of the complex presumably by compromising the stability of the hexameric ring formation in both RND- and MacB-based assemblies.^571,594,711,737^ Regulation of hexamerization and the modulation of the stability of these hexameric rings in response to substrate binding and OMF-engagement provide flexibility to the efflux and secretion machineries employing PAPs.

Paradoxically, the currently accepted model of the peristaltic cycle of the RND pumps^318^ has been inspired by the rotary mechanism of the F1-ATPase^773^ and the structural connection of the β-barrel rings of PAP and ATPase assemblies presented here, which allows us to extend this analogy to actual functional homology between the components of these complexes.

## 8 Assembly and Function of the RND-Based Tripartite Complexes

### 8.1 Cryo-EM Structures of Fully Assembled RND Complexes

PAPs appear to play a crucial role in the assembly of the trans-envelope complexes. Due to their unusual topology, early studies have speculated that PAPs are able to bridge not just the transporters and OMFs but also the opposing inner and outer Gram-negative membranes directly,^740^ which has partially led to their rather misguided assignment as “membrane fusion proteins” or MFPs,^722^ a designation which could still be found in some literature sources.

Until relatively recently our understanding of the structural arrangement of the tripartite pumps came from speculative models based on molecular docking, *in vivo* cross-linking, and mutagenesis studies.^269,649,650,693,713,716,759,791^ While these produced some convincing models, even such aspects as the stoichiometry of the assembly remained debatable in the absence of an experimental structure of a complete tripartite complex. A quantum leap in our understanding of these systems has been achieved by the maturing of the cryo-electron microscopy (cryo-EM) technology. The first to be addressed were cryo-EM structures of AcrAB-TolC, which were generated by coexpressing AcrA-AcrB fusion proteins^287,792^ or after stabilization by *in vivo* disulfide bridges.^305^ Later, reconstituted tripartite pumps could be prepared from purified proteins without additional stabilization in the presence of native lipids^270,304,306^ or even resolved by *in situ* by cryo-electron tomography (cryo-ET)^307^

A crucial insight has been provided by the higher resolution structures obtained by the group of Ben Luisi,^305^ in the presence and absence of substrate and inhibitors revealing different conformational states of the pump. The apo-state (PDB ID 5V5S) revealed a closed tip of the TolC-channel while the AcrB adopts a symmetric LLL conformation (resting state). In the presence of puromycin (PDB ID 5O66), the aperture of the TolC channel is dilated and while the AcrB is seen adopting an asymmetric LTO conformation, while the inhibitor in the presence of pyranopyridine inhibitor MBX3132,^341^ the pump was found in a pseudosymmetrized TTT state (PDB ID 5NG5).^305^ Importantly, the PAP, AcrA, was found to form a hexamer wrapping around the RND transporter through the trimerization of two conformationally distinct protomers. These were found bound to two nonequivalent interfaces on the surface of the transporter and are referred to here as PAP1 (with binding fully restricted to one RND-protomer and hence also referred to as intraprotomer-PAP) and PAP2 (which binds between the respective RND-protomers and is referred to as interprotomer-PAP) (see section 8.3 and associated figures below for details). The conformationally distinct RND-PAP interfaces between the protomers are suggested to play a key role in the long-distance allosteric coupling between AcrB and TolC. In the apo-state (PDB ID 5V5S), a gap is present at the interfaces between adjacent PAP dimer pairs, so that the PAP helical-hairpin, lipoyl, and β-barrel domains do not pack tightly to seal the channel from the periplasm.^305^ Based on these structures, it was suggested that AcrAB–TolC initially forms a complex in the closed state and that TolC opens upon rearrangement of the AcrA-hexamer induced by a conformational change in AcrB.^305^ In turn, the AcrA helical bundle would repack, imposing a contraction of the pump along its long axis by approximately 10 Å, and consequently seal gaps that would otherwise cause the substrate to leak out.

Two structures of the nonstabilized wild-type multidrug efflux pump, MexAB–OprM from *P. aeruginosa*, were also resolved at near-atomic resolution.^270,306^ In these structures OprM is found in two different orientations relative to MexB, related by a 60° rotation along the long pump axis. In contrast to the AcrAB-TolC structure described by Wang et al., both structures are found with OprM open. In order to account for the activation from a resting to an activated state, Tsutsumi et al.^306^ proposed a model where the resting state corresponds to the tripartite structure where MexB is under an LTO conformation but with a closing of the gating loop in its T protomer. Only in the presence of substrate is the gating loop within MexB suggested to experience a slight conformational change, allowing the substrate to access the distal-binding pocket.^306^ The conclusion is slightly different in the structure described by Glavier et al.^270^ for which it is described that transition from the resting to activated state is mediated by the binding of both MexA and OprM, allowing MexB to adopt an activated LTO conformation, hence corroborating *in vitro* functional results. In the sections below we will look at the PAP-OMF and PAP-RND interfaces elucidated by these structures in more detail.

### 8.2 PAP-OMF Interface

Due to their “adaptor” function, the PAP family plays a central role in the assembly of the tripartite complexes, effectively bridging the outer membrane (OMF) and inner membrane (energized transporter components). In the absence of direct structural data, a number of studies pointed out the primacy of the α-helical hairpin domain in engagement of the OMF and provided specificity of interaction between different pump complexes. For instance, swapping of the hairpin domain of MexA for that of MexE makes the MexA-E hybrid compatible with the OprM-MexF pump. Conversely, the chimeric construct is not able to function with OprN-MexF, and MexA bearing the hairpin from MexE does not form a functional complex with its cognate components OprM-MexB.^793^ Similarly, swapping the AcrA α-hairpin domain for that of MexA makes the chimeric OprM-AcrAB assembly functional.^713^ Cross reactivity among heterologous pumps was also tested, further highlighting the major role of PAPs in the specificity of recognition of assembly. For instance, TolC does not assemble with MexAB in *E. coli*(652) but does interact with and functionally complements both the AcrA-MexB^794^ and VceAB.^714^ Conversely, while VceC is able to interact with AcrAB in *E. coli* it does not provide functional complementation for the loss of TolC.^714^

While the central role of the PAP hairpin domain in OMF recognition and binding was never disputed, the nature of this bridging interaction has been a matter of much debate, with two competing models of assembly being proposed, which can be straightforwardly distinguished by the extent of the respective interaction interface between the OMF and the PAP α-hairpins. The first one, known as the “deep-interpenetration” model of assembly, as the name suggests, predicts an extensive binding interface between the PAP α-helical domain with the OMF helical-hairpin repeats, with stable four-helical bundles being formed on the inter- and intraprotomer grooves of the OMF. This model was the first to emerge historically and was primarily derived from biochemical data based on *in vivo* functional studies and cross-linking, as well as *ab initio* docking taking into account the propensity of coiled-coils to form helical bundles.^649,650,713,716,759,791^

**Figure 41:**
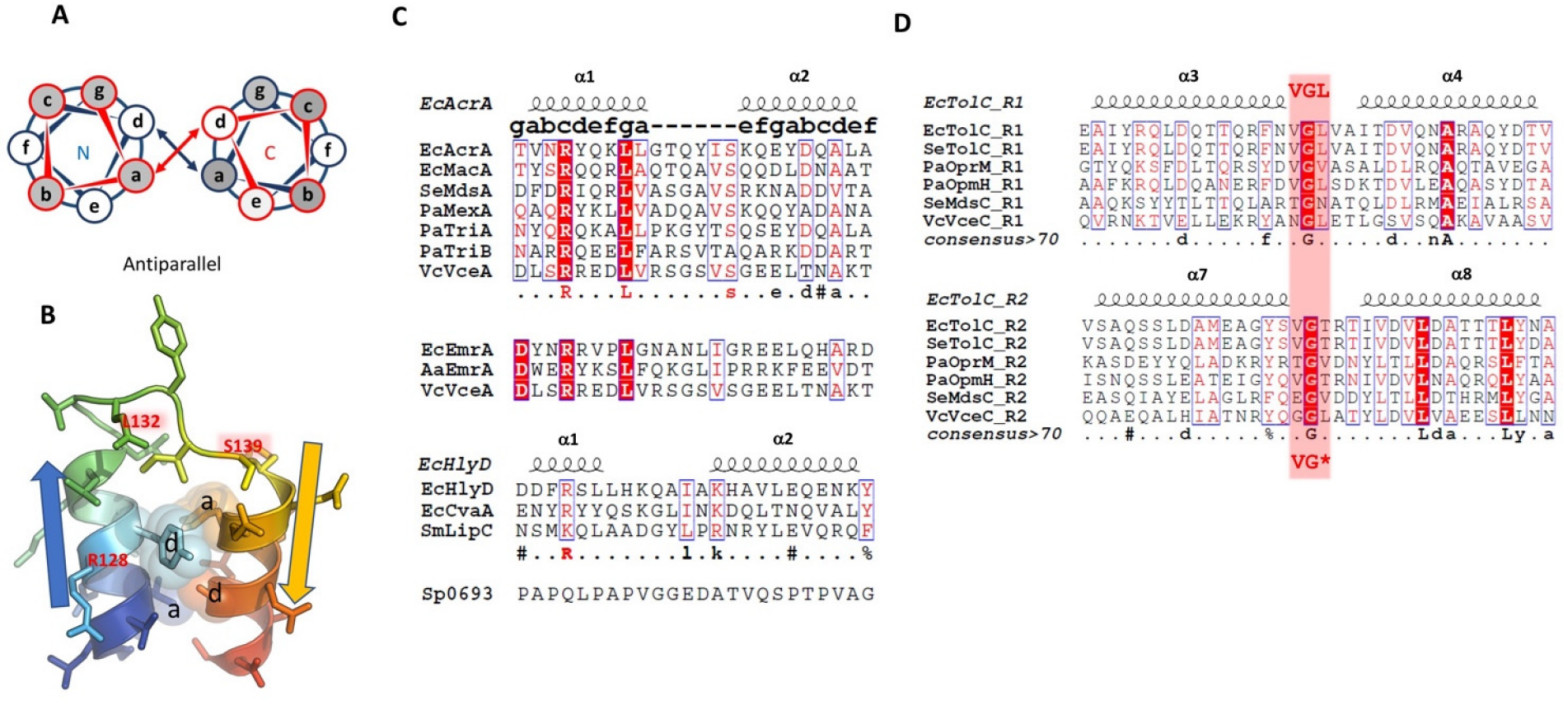
Helix–turn–helix motifs in PAPs and OMF that are supposed to mediate their interaction. (A) Schematic view of the coiled coil packing within the α-helical hairpin of the PAP showing its antiparallel interface. Conserved hydrophobic residues are interacting within the a and d positions of the respective N- and C-helices (projection view looking down the hairpin helices outward from the lipoyl domain). (B) A view of the α-helical hairpin tip of AcrA (based on PDB ID 2F1M),^735^ highlighting the interaction positions a and d; V126 (a) and A146 (d); and Y129 (a) and Y143 (a) form heptad-pairs. (C) The RLS-motif is present at the tip of the HTH motif of the PAP hairpin. Presentation of the classic-RLS-motif as observed in the (predominantly) RND- and MacB-associated assemblies, while MFS-associated PAPs show a deviation in the second half of the motif, and the T1SS-associated PAPs only retain a positively charged residue in the front of the motif, suggesting a different association with the OMF. Consistent with the lack of cognate OMFs, the PAPs in the Gram-positive organisms do not share any recognizable RLS. (D) R1 and R2 HTH-motifs at the tip of the static (H3/H4) and mobile (H7/H8) helices of the OMFs present a loosely conserved VG(L) motif, which is suggested to interact with the connecting loop of the RLS in the PAPs. Alignments in this figure have been visualized with Espript3.^795^

The second model, which has become known as “tip-to-tip” or “cogwheel”,^756^ is predominantly based on the structural information derived from crystallographic,^737,750^ cryo-EM,^270,287,305,306,747^ and *in situ* cryo-ET data.^307^ The tip-to-tip model initially came to existence from the observation of the crystallographic arrangement of the PAP MacA from the *Actinobacillus actinomycetemcomitans* (PDB ID 3FPP),^737^ which revealed a tight hexameric assembly, within which the α-helical hairpins of the PAP have been organized into a tubular structure, with a diameter strikingly similar to that of the fully dilated OMF channel.^687^ Furthermore, similar tip-to-tip assembly is formed by the OMF in isolation (PDB ID 2VDD),^689^ where two asymmetric TolC trimers were observed to pack crystallographically in an interpenetrative fashion. This similarity led to the suggestion that the two helical α-barrels of the OMF and the hexameric PAPs could interact in a much more limited way, resulting in the “cogwheel”, aka “adapter-bridging” model,^737,750^ something that has been further supported by cryo-microscopy of engineered MacA-TolC chimeric proteins.^746^ Consistent with the critical role of the RLS-motif (the canonical form of which is RxxxLxxxxxxS/T) and taking the form of a helix-turn-helix (HTX) motif at the tip of the α-helical hairpin domain, its targeting in different PAPs, including AcrA, MacA, and HlyD, has a detrimental effect on TolC-binding.^750−753^ The leucine of the PAP RLS-motif in MFPs is the most conserved residue, while the last position of the motif shows higher variability and can be occupied by a serine or a threonine residue (Figure 41, panels A–C).

Similar to the PAPs, the OMFs belonging to the TolC subfamily present conserved HTH-motifs at the tips of their H3/H4 and H7/H8 α-helical hairpins of the coiled-coil domain, referred to as *R1 and R2 motifs*(751,796) or *VGL-motifs*.^747^ These motifs contain a Val-Gly-Leu/Thr consensus sequence, which has been suggested to interact directly with the RLS-motif of the PAPs (Figure 41, panel D).

Strong support to the tip-to-tip association as a main driver of PAP-OMF interactions was provided by studies using chimeric PAPs, e.g. a MacA-TolC chimera, with only the R1 and R2 regions of the TolC transplanted onto the tips of the α-hairpin domain of MacA which was able to engage with MacA,^746^ while a MacA-MexA chimeric protein, containing the *P. aeruginosa* MexA α-helical domain (PDB ID 4DK1)^751^ was able to bind a similarly engineered MacA carrying the tips of the H3/H4 (Repeat 1 or R1) and H7/H8 (Repeat 2 or R2) helices forming the periplasmic tip of the α-barrel of OprM, showing that these are sufficient determinants for interaction.

**Figure 42:**
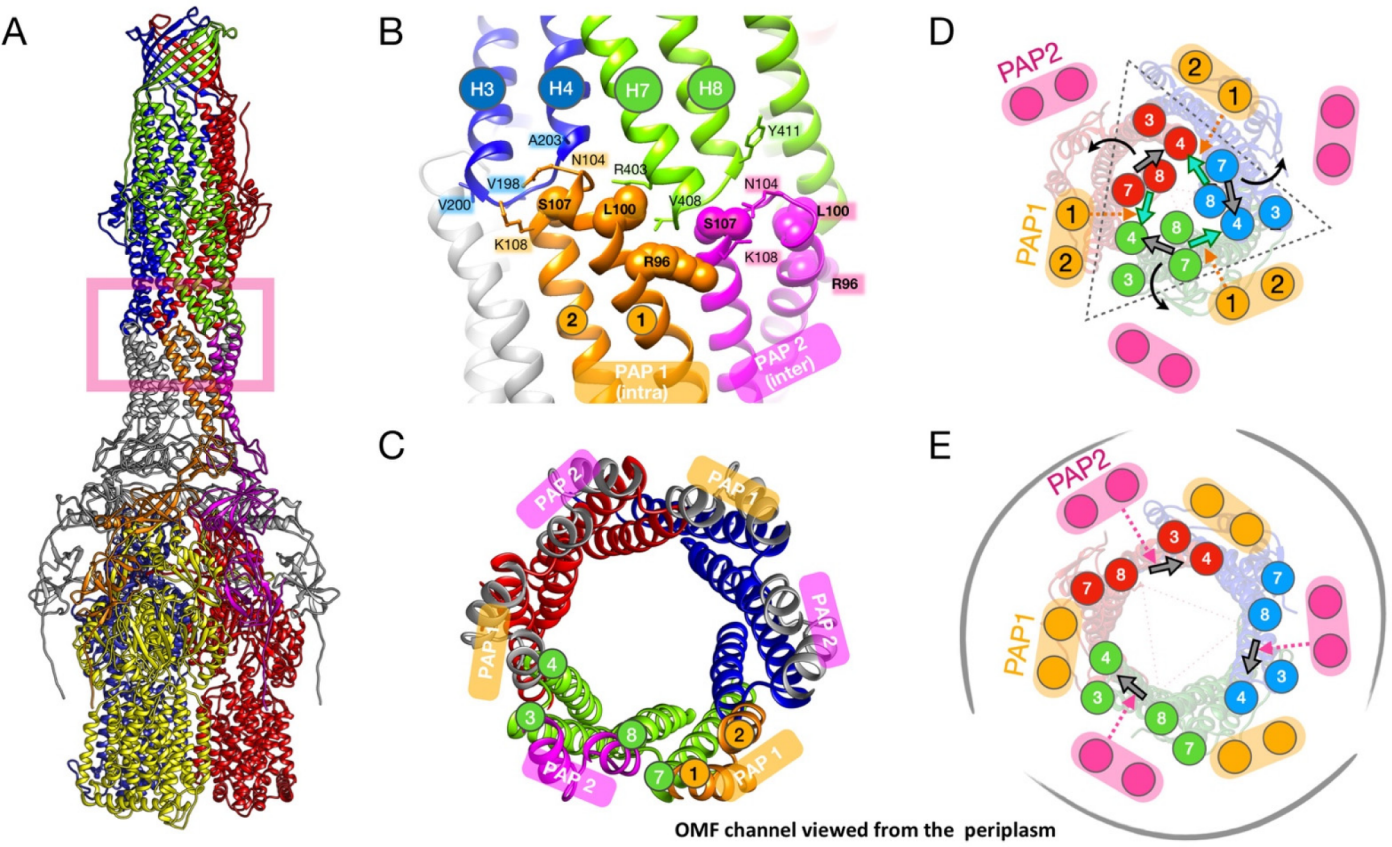
Interaction between the tip-regions of OMFs and PAPs on the example of OprM and MexA. (A) Side view of the MexAB–OprM complex as seen in the cryo-EM structure (PDB ID 6TA6(270)). The zone of PAP-OMF interpenetration is indicated by the rectangle. Helices of the respective OMF protomers are shown in red, green, and blue; while the α-helical hairpins of PAP1 and PAP2 are colored orange and magenta, respectively. (B and C) Zoom on the tip-to-tip interaction between OprM H3/H4 and H7/H8 helical tips (shown in blue and green, respectively) and the docked α-hairpins of MexA (shown in orange (PAP1) and magenta (PAP2)). Principal interacting residues are shown (proposed RLS-motifs are in bold). (D and E) Schematic view of the MexA channel and associated PAP protomers looking up the channel in closed and open (MexA-MexB-engaged) form, respectively. The binding of PAP1 protomer to the interprotomer interface is suggested to result in destabilization (orange dotted arrow in D) of the interprotomer gates (indicated by the cyan arrows), resulting in an outward swing of the mobile helices H7/H8 (black curved arrows), which are still attached to the H4 via intraprotomer interactions (gray arrow). The initial relaxation of the H7/H8 allows the docking of PAP2, which in turn destabilizes the intraprotomer gates (see the pink dotted arrow in E), leading to full dilation of the OMF aperture. While the exact gating residues differ between the specific PAP–OMF pairs, the general mechanism of PAP docking and relaxation of H3/H4 helices is suggested to be conserved. See the text for more details.

One of the expected outcomes of such models is a level of promiscuity and interoperability of the PAP-OMF pairs, and indeed there is some experimental evidence in their support; for example, the *Vibrio vulnificus* TolC orthologues TolCV1 and TolCV2 appear able to interact with the *E. coli* PAP MacA, forming functional complexes^753^ despite only having 51.3% and 29.6% identity with the *E. coli* TolC. Song et al.^797^ have also investigated the interaction between the putative tip-regions of the PAP MdsA, belonging to the *Salmonella*-specific tripartite transporter MdsABC, and its cognate OMF MdsC, as well as TolC, in the formation of functional MdsAB-mediated efflux pumps. Comparative sequence analysis revealed that despite low overall sequence homology between MdsA and MdsC with the endogenous *Salmonella* cognate PAPs (only 15% identity with MacA and 18.7% identify with AcrA) and OMFs, respectively, there is clear conservation of key residues in the putative tip regions of both the PAPs (MdsA has a clearly conserved RLS-motif consisting of R135, L139, and S146) and the OMFs, that suggests allowing interactions and formation of hybrid tripartite systems. The predicted promiscuity was indeed observed in complex formation, suggesting that the productive complex formation is restricted by a small number of conserved residues and interactions at the respective tips of the hairpin domain of the PAP and the H3/H4 (R1) and H7/H8 (R2) of OMF,^797^ further enhancing the RLS-R1/R2 interaction hypothesis (Figure 42).

Based on these observations it has been postulated that the RLS-motif is conserved across PAPs engaged in RND pumps, MacB-family ABC transporters,^746,750^ as well as PAPs involved in T1SS such as HlyD.^752^ However, the predicted RLS-motif in the HlyD family of PAPs appeared to be significantly degenerated, and later crystallographic studies revealed that the predicted tip of the HlyD is misaligned,^739^ with the tip sequence differing dramatically from the canonical RSL-signature. Similarly, the structure of the LipC from the lipase-secreting T1SS of *S. marcescens*(514) does not present a recognizable RLS-motif, suggesting that the T1SS systems may employ a slightly different mode of OMF-unlocking and/or stabilization and indicating that the role of the motif in OMF-recognition is not as clear-cut as previously thought. In addition, the hairpins of PAPs from Gram-positive organisms have recently come to light, e.g., Spr0693 from *Streptococcus pneumoniae*,^571^ and perhaps unsurprisingly they do not show any significant conservation of the hairpin tip, while maintaining the α-helical domain, raising further questions to its actual function (Figure 41, panel C).

This predicted PAP–OMF interaction was indeed directly observed in the cryo-EM structures derived from chimeric^747^ and covalently fused AcrAB-TolC assemblies,^287,792^ as well as the later cysteine-cross-link stabilized AcrABZ-TolC structures.^305^ More recently conclusive evidence has been provided by the complexes reconstituted without any additional stabilization,^270,304,306^ and thus the tip-to-tip model has become a dominant paradigm of PAP–OMF interaction.^756^

The cryo-EM structures reveal an OMF–PAP interface consistent with tip-to-tip interactions between their α-helical hairpins, with participation of the conserved motifs from the respective proteins discussed above; for example, within the AcrAB–TolC complex,^305^ the conserved VGL/T R1-motif of TolC is located at the concave region of the TolC cogwheel in close proximity to the conserved R128 and L132 of the AcrA RLS-motif. There, the G365 from the TolC R1-motif is found to form backbone to backbone interaction with AcrA-K140 and the side chain of AcrA-S139.^287,747^ Furthermore, consistent with earlier *in vivo* site-specific cross-linking experiments, N145 and T366 of TolC are found to contact L132 from the RLS-motif of AcrA.^736^ Due to the quasi 6-fold symmetry of the TolC interfaces, similar interactions are seen between the R2-motif of the OMF and the second copy of the PAP. Homologous contacts can be seen in the high-resolution structures of MexAB-OprM^270,306^ as illustrated in Figure 42, lending further support to the tip-to-tip model of interaction. Molecular dynamics simulations in POPC model-lipid bilayers confirmed that this fully assembled tip-to-tip pump is stable over microseconds. In addition, a sequence covariation analysis confirmed that interfacial contacts do involve residues consistent with those identified from the simulations.^798^

Despite the overwhelming structural biology support for it, there are certain issues with the “tip-to-tip” model, stemming from the limited interaction interfaces and the limited use of strong side-chain specific interactions between the predicted PAP-OMF binding surfaces, which limits the ability of each protein to differentiate their cognate binding partners. Furthermore, some experimental data is difficult to reconcile with such models, including functional complementation of concatenated pumps with enforced 3:3:3 stoichiometries,^799^ SPR measurements,^698^ and a range of direct *in vivo* cross-linking and mutagenesis studies.^650,652,711,717,718,791^ While some of these may be dismissed as artifactual, the existence of PAPs without discernible α-hairpin domains, such as BesA,^32^ which are able to form functionally active, channel-forming tripartite assemblies could not be readily explained by a classical tip-to-tip model.

### 8.3 RND–PAP Interface: Structural Data Suggests Asymmetry of the Assembled Tripartite Assembly and Different Roles for the PAPs

While earlier models of tripartite assembly gave preference to the 3:3:3 stoichiometry,^649,716^ a number of conflicting data have been accumulated that shifted the consensus toward 3:6:3 assembly. These included the discovery of RND transporters which partnered with two separate PAPs, e.g., TriABC-OpmH,^88^ as well as the demonstrable functionality of the tandem-fusion AcrA constructs.^736^ Furthermore, *in vivo* cross-linking to both inter- and intraprotomer grooves of the OMF MtrE supported the existence of a 3:6:3 stoichiometry of the OMF-PAP-RND, leading to the suggestion that such an arrangement would inevitably result in a nonequivalent binding of the PAPs per protomer.^759^ Subsequent structural studies have provided conclusive evidence of this nonequivalent binding from both HAE and HME pumps.^270,287,305,306,359^ Correspondingly, the nonequivalent binding sites on the surface of the RND pump create two functionally nonequivalent PAP-conformers, which are referred to as PAP1 and PAP2,^270,305,306^ as seen inFigure 43. PAP1 is exclusively bound within the limits of a single RND transporter protomer with which it makes extensive interactions and is hence referred to also as an *“intraprotomer-PAP”* relative to the RND subunit. PAP2, on the other hand, contacts both the RND-subunit to which PAP1 is bound and the following anticlockwise protomer (looking from the top down along the axis of the pump from outer cellular space), straddling the RND-interprotomer gap, and hence in the text below is also referred to as an *“interprotomer-PAP.*

**Figure 43:**
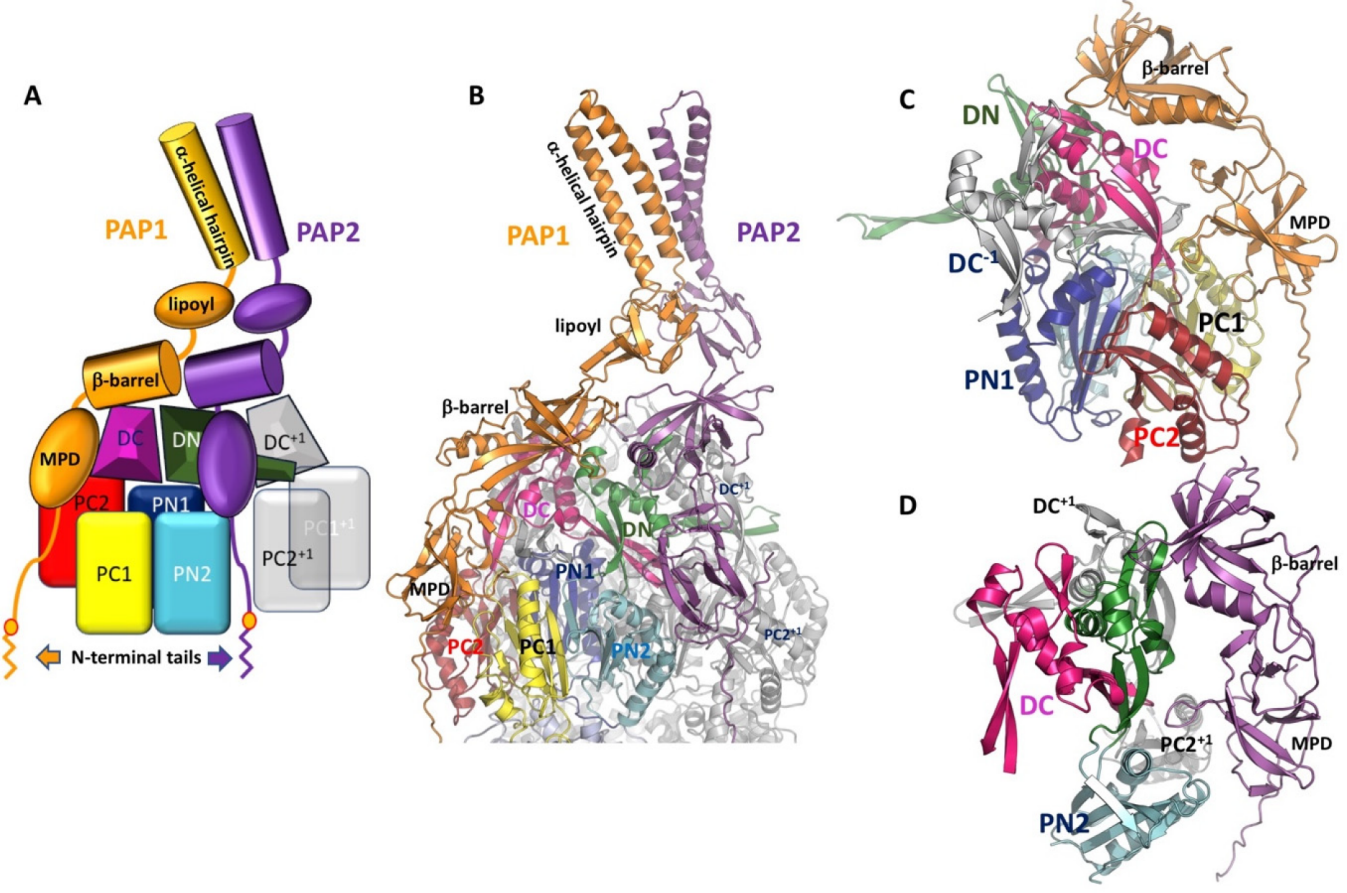
Two nonequivalent binding sites on the surface of the RND transporter accommodate two distinct conformers of the PAP, which serve distinct functions during the cycling of the pump. (A) Side view of the MexAB-OprM assembly (PDB ID 6TA6)^270^ showing the binding of the intraprotomer-PAP subunit (PAP1) (orange) and the interprotomer-PAP subunit (PAP2) (magenta). The different RND subdomains are colored, and neighboring RND protomer is colored gray. (B) Cartoon representation of the same angle of view labeling the different subdomains of the PAPs and RND. (C) Isolated views of the contact areas of the PAP1 and PAP2 relative to the RND; for clarity the lipoyl and hairpin domains which do not contact the transporter have been removed.

The PAP1 β-barrel domain docks onto the DN- and DC-subdomains of the RND-porter domain (Figure 5 and Figure 6 RND transporter section and Figure 43, showing the arrangement seen in the MexAB–OprM complex), while its MP domain interacts with the PC1 subdomain, as well as with the linker region between PC2 and DC, and an extended loop of the DN subdomain from the follow-up RND (Figure 43, panels A–C). PAP2 on the other hand forms fewer contacts, which are focused predominantly on the crown of the DN-subdomain, which is contacted by the β-barrel domain of PAP2, plus its MP-domain appears to be relatively loosely associated with the surface of the RND protomer, corresponding to the PN2-DN linker region (Figure 43A, B, and D). Despite the big discrepancy of the PAP binding sites relative to the transporter and their complex 3D arrangement, a closer analysis of the interacting PAP residues revealed that the contacts provided by both PAP1 and PAP2 are closely overlapping and restricted to several discrete linear sequences, which we have previously dubbed “binding boxes”.^742^ These are restricted to the β-barrel (boxes 1–5) and MPD domains (6–9) and are highly conserved within specific PAP families; however, not all of them appear to have a pronounced functional role. Disruption of a few key residues within boxes 1, 4, and 5, mapping to the exposed β-barrel loops, abrogates transport, and hence, these have been suggested to play a role in assembly of functional complexes and the differentiation of cognate PAP–transporter pairs.^742^ Notably, while PAP1 and PAP2 share the same β-barrel binding sites, there are discrepancies in the MPD attachment, and the domain is seen to be undergoing significant reorientation akin to “rolling” between the two protomers, resulting in PAP1 and PAP2-specific binding boxes, highlighting their differential modes of engagement and, likely, function.^742^

Furthermore, comparisons of the structures of RND transporters in isolation (also referred to as “mono”-structures below) vs their structures within tripartite assemblies in the presence of PAPs revealed by cryo-EM demonstrate significant differences, suggesting allosteric changes are enhanced and perhaps actively mediated by the PAPs,^305^ e.g., spatial orientation of the DN-domain β-hairpin motifs forming its crown, which have been implicated in the assembly of the pump,^712^ was noted to be impacted, and it was suggested that this conformational rearrangement facilitates the interactions between the RND-protomer and the β-barrel domain of PAP2, as well as the short “crown” α-helix of the DC-subdomain from the next-RND protomer.^305^ Identical contacts are also seen in the MexAB-interface of the assembled MexAB-OprM pump,^270,306^ and similar interaction is revealed by the binary CusBA complex,^359^ although the orientation of the β-barrel and lipoyl domain differs from the HAE1 pumps and the PAPs are rather loosely fitted to the crown of the transporter. The PAP1 MP-domain locates to the entry of the proximal binding pocket and thus has been suggested to play a role in substrate presentation and/or sensing of the cargo-load state of the transporter, which is also supported by the location PAP1 MPD observed in the CusBA complex, where such a role has been clearly shown.^359^

A significant new insight into the allosteric effects of the PAP-association came from the recent works on the MexAB-OprM,^270,306^ which highlighted the special role of the short helix (formally known as Nα2′),^279^ which is found on the loop connecting the α2-helix and the β4-strand of the central β-sheet of the PD module of the PN1 subdomain of the transporter (Figure 6 and Figure 43, panel C). This short helix, which has been dubbed “helix gate”,^270^ participates in formation of the exit gate, which controls the exit of the substrates into the PAP-OMF funnel and in the case of MexB is formed by the Q124, Q124 belonging to the helix gate plus a critical residue (Y758) from the DC domain of the RND.

Comparison of isolated RND transporters (aka “mono”-structures) vs the assembled RND tripartite complexes available shows that the interprotomer groove between T and O protomers is wider than the one observed at the LL-, LT-, or OL-interfaces. Intriguingly, this interface widens even further in the PAP2-engaged fully assembled “trio” state.^270^ A closer inspection of the mono structures of MexB revealed that despite the seeming L-T-O cycle being present and the corresponding transformation of the deep binding pocket to outward-facing conformation, the drug exit gate remains occluded, and thus the drug cannot leave the pocket. This “closed” transition state was dubbed the C-state.^270^ The closed (C) intermediate appears to be a state between the T and O states. Only after C converts to O is substrate released. Of note, the process from the apo-state (L) over drug bound state (T) to the release of the drug in the O state is postulated to be PMF-independent.

**Figure 44:**
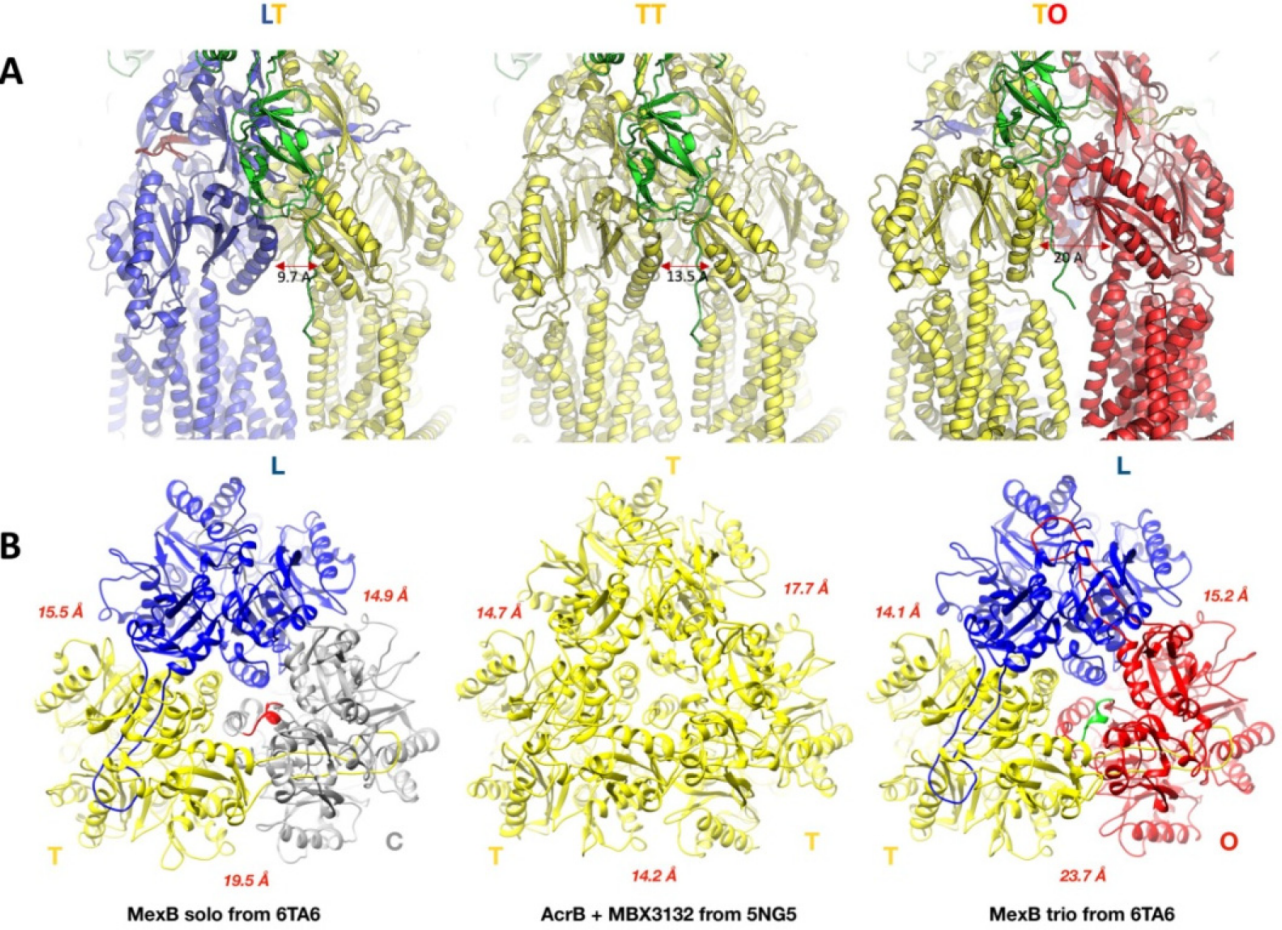
Comparison of interprotomer interfaces of RND transporters in isolation and upon PAP binding. (A) Side view of the superposition of the T-protomers of AcrB (as seen in asymmetric LTO and symmetrized TTT structures) and MexB (LTO) as seen in tripartite complex as observed in PBD ID 6TA6.^270^ RND-conformers are colored: L, blue; T, yellow; O, red; PAP2 confomer, green. (B) Top-down view of the trimeric assemblies of MexB solo (PDB ID 6T7S),^270^ MexB “trio” from the tripartite MexAB-OprM structure (PBD ID 6TA6),^270^ and the AcrB (TTT-form stabilized by the presence of the MBX3132 inhibitor) (PBD ID 5NG5).^305^

In the “trio”-structure, the PAP2 MPD appears to act as a wedge, which is slotted into the T-O interprotomer crevice, which is associated with a concerted movement of the PN2 subdomain of the T protomer and the PC2 domain of the neighboring O-protomer sidewise (Figure 44). These domain reorganizations have been suggested to translate to the underlying PN1 domain and its “helix-gate” mentioned above, allowing for the transition of the closed C-form to the open O-form of the transporter,^270^ emphasizing the specific role of the PAP2 in the efflux-vetting process.

There is a general consensus that PAP and the RND transporter initially form a binary complex, as corroborated by *in vivo* evidence,^696,800^ as well as by biochemical investigations,^694,698^ and that subsequently this binary assembly recruits the OMF to form a functional tripartite complex. It was suggested for a long time that PAPs play an active role in transducing conformational energy from substrate/proton binding in the RND transporter to the OMF to help in transitioning its channel from its closed to open state allowing for the creation of a functional efflux conduit, via mediation of the helical hairpins of the PAPs.^288,318,649,689,801^ Substitutions in the R1 motif targeting the loop connecting H3 and H4 in TolC result in antibiotic hypersensitivity despite the fact that their physical interaction is maintained as judged by *in vivo* chemical cross-linking. Vancomycin sensitivity assay revealed that AcrA-mediated suppression of this antibiotic sensitivity was achieved by dilating the aperture of the TolC channel in an AcrB-dependent manner,^711^ allowing suggestion that the allostery of the overall RND-cycle mechanism substrate binding by AcrB is linked to proton transport, whereby conformational movements occurring within AcrB would be captured by the AcrA MP- and lipoyl domains (which at the time were believed to be in contact with the RND transporter) and transferred to TolC via the α-helical hairpin of the PAP.

While later structural studies did not support the direct contact of the lipoyl domains of the PAP with the transporter and the deep-interpenetration models of PAP–OMF interaction have fallen out of fashion, the idea of the conformational transitions in the RND being conveyed by allosteric PAP mediation was proven largely correct. The cryo-EM data of the AcrAB-TolC complex obtained in the presence or in the absence of puromycin by the group of Ben Luisi allowed us to further elaborate on an allosteric, concerted, opening^305^

There, conformational changes in the periplasmic headpiece of the RND transporter upon substrate binding are suggested to instigate repacking of all four of the PAP-domains, which in turn leads to reorganization of the coiled–coiled domain of the PAPs, which is implied to be the key step in organization of the RLS-motifs and opening of the OMF channel,^305^ which is engaged in a tip-to-tip fashion. Upon opening of the OMF, the 3-fold symmetry in the apo-complex transitions to a quasi-6-fold symmetry of the interface between PAP α-hairpin and open-state OMF. This leads to rearrangement of the helical bundle, creating a tightly sealed nanotube, which in turn leads to rearrangements further down, resulting in repacking of the lipoyl and β-barrel domains of the PAPs and sealing of any the gaps to the periplasm,^305^ allowing for productive efflux even to take place. A similar mechanism has been suggested for the MexAB OprM pump in the presence of novobiocin.^306^

There is also a visible reorganization of the MPD domains between the PAPs in apo- and asymmetric, substrate engaged state, which is likely associated with their changing functional role through the cycle;^305^ however, due to the interoperability of the PAP1 and PAP2 in such pumps as AcrAB-TolC and MexAB-OprM, narrowing the specific roles of PAP1 and PAP2 has proven difficult.

### 8.4 Heterooligomeric Complexes Allow Unambiguous Assignment of PAP1 and PAP2 Highlighting Their Distinct Roles during Assembly and Cycling of the Pump

In *P. aeruginosa*, the tripartite pump TriABC-OpmH, which is associated with triclosan resistance,^88^ has a distinct organization, as its transporter component, unlike most of the RND transporters discussed above, which function with a single PAP, creates a heterooligomeric complex with the participation of two different PAP subunits: TriA and TriB. The two PAPs only share 36% identity and are not interchangeable, suggesting specialization of function, which is consistent with each of them occupying a specific binding interface in respect to both the RND transporter TriC and the OMF protein OpmH.^802,803^ Due to these unique characteristics, this system has provided a valuable insight into the distinct roles played by PAP1 and PAP2, allowing assignment of each of the PAPs to a specific functional role. A recent cryo-EM structure of the TriABC subcomplex (PDB ID 6VEJ)^302^ confirmed the locations of the TriA and TriB relative to the transporter and unambiguously assigned the PAP1 and PAP2 roles to them, respectively. The subcomplex was resolved in its unliganded “drug sweeping” state, which displays high mobility of the PAPs, with the hairpin domains being in disarray. However, the MP-domains could be resolved in their respective positions, and as expected the PAP1 (TriA) was found to have high-contact area and occupy the intraprotomer interface between the PC2 and PC1 subdomains, just above the substrate entry point to the proximal drug binding pocket, while the MPD of PAP2 (TriB) was found bound closer to the interprotomer interface between the RND-protomers. Notably, however, while the PAP1 MPD is fully resolved, the electron density for the PAP2 MPD is patchy, corresponding to low occupancy. Furthermore, intriguingly, in this substrate-free state of the pump, the MPD of PAP2 seems to be primarily localized to the PN2 domain of the RND protomer and is very loosely attached to it,^302^ in stark difference to the snug-fit observed in the recent MexAB-OprM structures, where it is also bridging the interprotomer gap.^270,306^

Mutation of the RLS-signature motif within the TriA/TriB seems to have different effects. While the TriA-R130D mutant fails to assemble a functional TriABC complex with the OMF, the alteration of the corresponding R118D in TriB does not significantly affect the assembly and activity of the complex.^802^ At the same time, mutation of the conserved MPD residue G350C (TriA) and G339 (TriB) had the opposite effect, with the first one being tolerated and the second being detrimental to pump function,^802^ suggesting that it only plays a significant role in the PAP2. Intriguingly, the mutation of the corresponding residue, which maps to “binding box 9”, also obliterates efflux in the *Salmonella* AcrA/AcrE PAPs.^742^

Antimicrobial susceptibility, site-directed mutagenesis informed by homology modeling, alongside with *in vivo* disulfide cross-linking and azithromycin-sensitivity assays allowed further differentiation of the PAP1 and PAP2 roles relative to the cognate OMF (OpmH).^803^ It was further confirmed that while PAP1 (TriA) is required for OMF recruitment, the activation of the transporter (which, as mentioned above is linked to the MPD of PAP2) is coupled to the opening of the OMF aperture.^803^ Data compiled from azithromycin sensitivity studies, which indicate leakiness of the OMF-channel, as it does not normally penetrate the channel and is not a substrate of the pump, suggested that TriB is essential for keeping the channel dilated, which is consistent with it associating with the mobile H7/H8 helices of the R2 motif.^803^ Such interpretation is compatible with the position of the PAP2 observed in the “zero-degree” structures of the OMF (PDB ID 5NG5,^305^6IOK,^306^6TA6^270^).

Further, *in vivo* proteolysis suggested that both TriA and TriB are conformationally flexible, causing susceptibility of their α-hairpin domains to digestion. However, while TriA (PAP1) is stabilized by the presence of the OMF and the transporter, the proteolysis of TriB (PAP2) is dependent on the presence of transporter, further suggesting that it may be a more dynamic and loosely associated member of the complex.^302^ As the TriA and TriB are noninterchangeable, these studies allow us for the first time to correlate the rotationally symmetrical PAP–OMF interface with the underlying and already established PAP–RND interfaces, giving a unified view of the tripartite assembly and the roles of respective components which we will present in the following sections.

### 8.5 Conformational Cycling of the Protomers within the AcrB/MexB Trimer

Before we proceed with a unified model of the assembly, we would like to recapitulate a few key elements of the conformational cycling of the RND transporters. The majority of the current understanding of the RND-transport to date has been derived from the study of the inner-membrane transporters in isolation. The conformational LTO cycling hypothesis suggested an allosteric bisite activation^270,321,335^ and is not necessarily suggesting that the three protomer conformations (LTO) are constantly present within the trimer at all times during the cycling. This notion is supported by cross-linking data,^321^ suggesting that the LLT, LTT, and TTT trimeric conformations (next to LLL and LTO) are possible trimeric conformations. Structural support for this conformational flexibility came recently by observations via single-particle cryo-EM as this variety of different conformational setups was adopted by trimeric AcrB in the AcrABZ-TolC complex in complex with the inhibitor MBX-3132.^305^ Experimental support for bisite activation (allostery) came from molecular dynamics studies and kinetic β-lactam transport studies in whole cells, which were in dependence of the substrate concentration non-Michaelis–Menten kinetics with Hill-coefficients >1 measured.^346,804−806^ All these conformational cycling models did not include a systematic role for the PAPs thus far, despite them being clearly implicated in the initial opening (and closing) of the OMF channel.

In the original LTO cycle,^288,292,318,335^ the cycle starts with the symmetric metastable LLL conformation and the efflux substrate is anticipated to bind to the proximal binding pocket of one of the L protomers (to the AP) (Figure 5). Upon conformational change to T (thus forming an LLT trimer), the substrate is suggested to be further transported to the DBP (comprised by the PN2 and PC1 subdomains). In this drug-bound conformation, TM2 transduces the binding energy toward the TMD of the transporter allowing protons to enter the TMD and to bind to the titratable residues (D407 and/or D408 in the case of AcrB),^328^ which causes a change in the electrostatics within the TMD. This results in a conformational change, and the transduction of the energy of proton binding is mediated via TM8 to the rigid PN1/PC2 subdomain unit^292^ of the periplasmic domain. This results in the closing of the AP and DBP and the opening of a substrate exit gate. However, in order to anticipate a bisite activation in analogy to the functional rotation cycle of the F_1_F_o_-ATP-synthase,^320^ the neighboring protomer has to be in the T state as well. In the presence of substrates or inhibitor,^305^ any L protomer (and T protomer) can accept substrate and reach the conformational transduction state that allows protons to enter from the periplasm to the protonation sites.

Under the current models, the LLL to LTO transition appears to only require substrate binding, which has been corroborated by MD-studies,^806^ and indeed, the very existence of the LTO conformation in crystals and EM-resolved complexes reconstituted from purified components suggests that the conformational transitions could be obtained in the absence of PMF energy. However, the pre-existence of the LTO in a resting apo-state has been subject to considerable debate,^338^ and recent studies suggest that while LTC transitions could indeed happen in the absence of PAPs, the substrate binding alone may not be sufficient for the transit of the transporter from an LTC to the LTO configuration *in vivo*.

Earlier FRET studies of CmeB transporter reconstituted into liposomes have shown that it could cycle, albeit inefficiently, in the absence of the PAP or OMF components.^299^ While individual protomers have been detected undergoing L-T-O transitions, there seems to be little evidence of cooperativity and directionality of cycling. These findings raise the question on whether the role of the PAPs may be associated with both the facilitation of the conformational transitions between the RND protomers and enforcing the directionality of the cycle. In the section below we try to integrate the latest structural advances in understanding of the RND assembly and the roles of the PAPs within it into an integrated model of assembly of the complex, which takes into account both the PAP–OMF and PAP–RND interactions.

### 8.6 Toward the Unified Model of RND Pump Assembly—An Integrated Model of PAP Participation in the Efflux Cycle and Resetting of the RND Assemblies

Corroborating the recent results on the MexAB-OprM^270,306^ and TriABC-OpmH systems,^302^ with the earlier allosteric model derived from the AcrAB-TolC discussed above,^305^ we would like to suggest a modest modification of this model, incorporating a simple mechanism based on varying the affinities of the interprotomer binding sites created by the peristaltic cycling of the RND transporter toward the PAP2.

The key to pump stability and processivity during these cycles and detachments is the continuous association of the hexameric β-barrel gasket with the transporter, which allows for bespoke regulation of the engagement of their MP-domains with the different conformers of the transporter. In essence, we propose that while the intraprotomer (PAP1-binding site) remains high-affinity throughout the pump cycle, the affinity of the PAP2 binding site changes due to the conformational changes of the RND-protomers depending on drug-binding and proton-occupancy, with the L/L, L/T, and L/O presenting low-affinity sites, while the PAP2-binding site formed by the T-protomer is strongest. This applies specifically to the MP-domain, which could possibly detach fully between the different transitions.

The flexibility and the strategic position of the PAP2 straddling the interprotomer interface of the RND make it an ideal candidate for both sensing and communicating the state of the preceding RND-conformer to the next. It seems therefore possible that the PAP2 MPD acts as molecular “latch” or “ratchet” to prevent backsliding of the conformations during cycling, thus stabilizing the metastable form C/O conformer and allowing the exit of the transported cargo.

We also suggest a two-way communication between the RND transporter and the PAPs. The first stage is an upward signaling from the RND toward the OMF via PAP1, forming the molecular basis for OMF recruitment, which in turn enables the second, downward signaling stage, which commences with the association of the PAP2 with the OMF, resulting in the stabilization of its dilated aperture. This translates into a rearrangement of the α-helical hairpins of the PAP2 sealing the PAP-OMF drug-export conduit and is communicated back to the RND via allosteric transitions within the PAP2 allowing the MP-domain to slot into the T/C interprotomer crevice, that ultimately leads to movement of the “helix-gate” in PN1 (see Figure 43). This latter retrograde communication would correspond to the “activation signal” described as a switch from LTC to LTO in the cryo-EM structure of MexAB OprM.^270^

**Figure 45:**
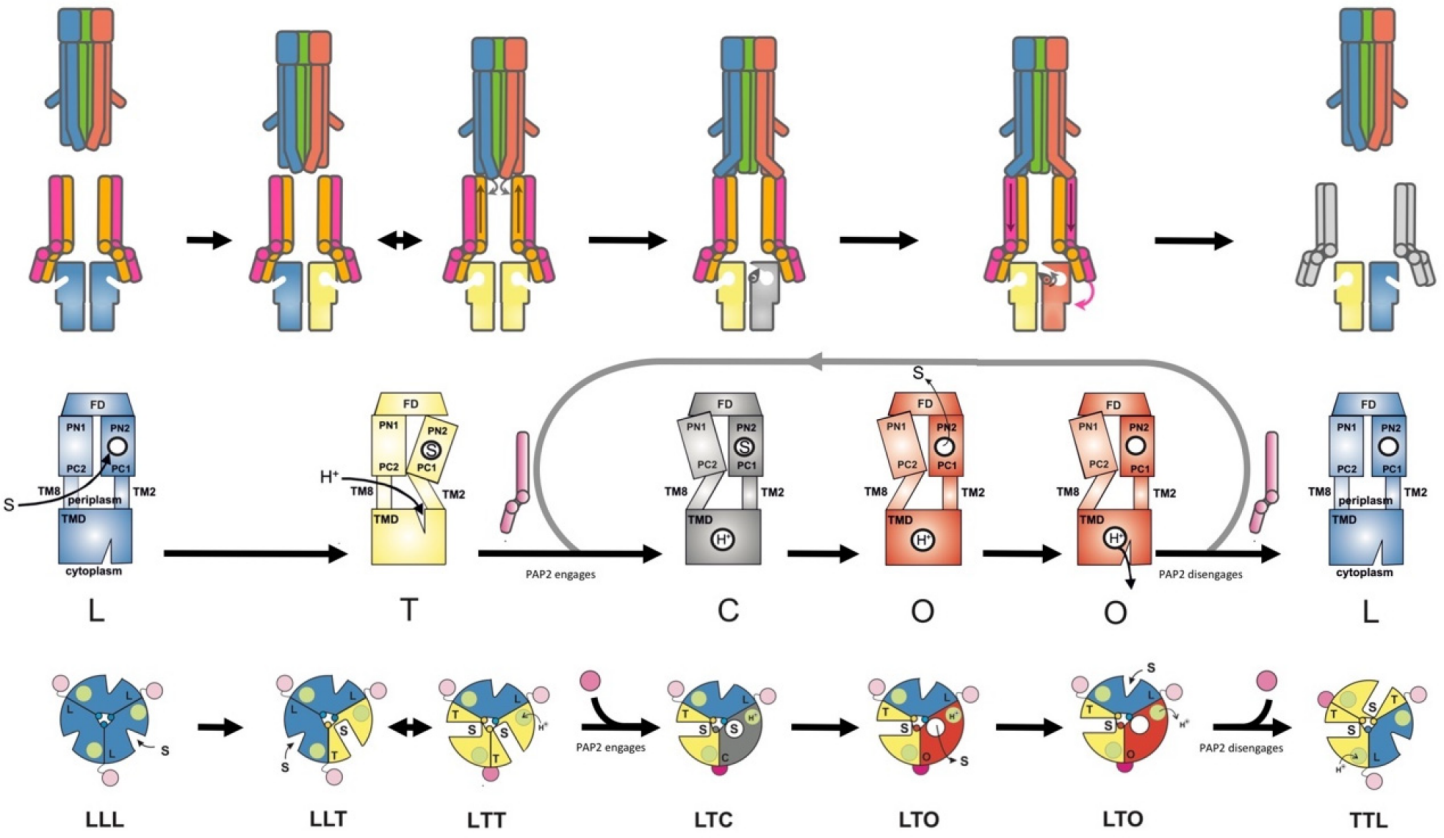
Conformational cycling of AcrB/MexB protomer and trimer in dependence of PAP engagement and disengagement. The tree panels present different aspects of the interaction focusing on the PAP-OMF (top) and PAP-RND (middle and bottom). In *the upper panel* the engagement state of the periplasmic adaptor protein conformer 2 (PAP2) is indicated based on the studies by Tsutsumi et al.^306^ and Glavier et al.^270^ Moreover, as an extension of the previous models,^288,318,335,807^ the closed state structure (denoted “C”) found by Glavier et al.^270^ is implemented, as well as the structural interpretation by Glavier et al.^270^ concerning the engaging role of PAP2 at the AcrB/MexB interprotomer interface. In *the middle panel*, the conformational cycling of a single RND protomer is shown (l-protomer, blue; T, yellow; C, gray; O, red). Whereas all PAPs (PAP1, PAP2) form a stable hexameric arrangement via their β-barrel and lipoyl domains, the membrane proximal domains (MPDs) are anticipated to be in a disengaged or engaged state. The binding of the MPD-domain of the PAP2 is suggested to take place at the interprotomer interface of the RND. The interprotomer crevice is closed between L and L (LL) as well as in LT and OL. Between TT this interface crevice is widened, and in TO, the gap is large. In *the lower panel* the conformational state of the protomer shown in the upper panel is given in relation to the states of the other protomers in the trimer (“wheel” representation). The role of the PAP2 MPD engagement is postulated to be supportive for the direction of the cycling (L → T → O → L), preventing the backward sliding of the O-to-T state by acting as a molecular latch. The complete conversion from L to O in the presence of substrate (S) including the protonation of the TMD and the release of the substrate from the O protomer is suggested to be PMF-independent and might explain why the LTO conformations are observed in isolated protein samples upon structural analysis. The return from the O state to the L state, however, is a PMF-dependent step in analogy with the ABC transporter cycle shown in Figure 16. FD, funnel domain; PN1, PN2, PC1, and PC2 are the subdomains of the AcrB/MexB porter domain; TM2 and TM8 are the transducing helices coupling energy transduction from the porter domain to the TMD and *vice versa*); TMD, transmembrane domain.

A summary of the key points of the model is presented in Figure 45, which provides three slices focusing on the PAP–OMF interface (top), the transporter–PAP interface from a side-view (middle), and a top-view rotary diagram (lower panel), taking account of the substrate and proton occupancy.

Under this model, the two PAPs within the RND assembly have clearly delineated and distinct functional roles. PAP1 is primarily associated with the first phase of the efflux cycle and is involved in presentation and feeding of the cargo to the transporter’s proximal binding pocket and sensing the successful transition of the drug onto the deep binding pocket, which is associated with the L-to-T transition, while PAP2 is predominantly associated with the second phase, namely detection of the successful docking of the OMF and vetting the release of the drug toward the OMF-funnel by facilitating/stabilizing the T-to-C transition and actively enabling the C-to-O switch, which leads to productive efflux of the drug. The first function requires a close association of the transporter-PAP, as the conformational change associated with the drug-binding and L-to-T switch needs to be communicated “upward” toward the OMF, via reorganization of the β-barrel and lipoyl domains, leading to the stitching-up of the helical-hairpin assembly to form a tube and recruit the OMF, which in turn enables the OMF opening and stabilization of the OMF-PAP assembly by PAP2 by completing the interlocking of their respective helical hairpins as suggested by Tsutsumi et al.^306^ Indeed, studies of binding of hairpins in isolation to the WT OMF MtrE vs mutationally stabilized open state (MtrE E434 K) indicated approximately 100-fold increase in affinity of the interaction.^719^ The significant realignment and tightening of the helical bundle of the PAPs upon OMF binding is associated with a subsequent compression of the lipoyl and β-barrel domain gaskets by ∼10 Å completing the formation and the sealing of the tubular conduit.^287,305^ The conformational trigger from this compression and realignment is propagated through the PAP2 enabling its MPD to engage with the DN/PN2 of the T-state RND protomer.

Via these protomer-specific interactions, PAP1 and PAP2 provide outward and inward communication from the RND and from the OMF, respectively, utilizing their conformational changes for coupling remote allosteric sites, which are consistent with the models suggested previously.^270,305,306,698,757,758^ Intriguingly, we have identified significant differences between the different RND-conformers in the engagement of the DC domain’s β-hairpins with the PAP2 (box 6) in the MexAB-trio structures (PBD ID 6TA6),^270^ specifically involving the MexB R764 and the main chain of the PAP D278. This interaction may provide an additional level of allosteric communication, as the critically important Y757, which forms part of the MexB drug-exit gate, is located at the base of the same β-hairpin that provides the PAP2-attachment point, and conformational changes as a result of PAP2 binding may be communicated via it to the drug exit gate. Furthermore, the box6-interaction mediated by the PAP2 R277 and the RND transporter E244, which maps to the short helix at the base of the DN-domain hairpin, may convey conformational changes from one RND protomer to the other. While the significance of these interactions needs to be investigated further, they could help explain the C to O transition trigger and forward communication between the PAP2 and subsequent RND-protomer. It is therefore tempting to suggest that the hexameric PAP β-barrel ring not only provides increased processivity but also enables the one-way PAP2 “latch system”, which in turn gives directionality to the cycle. This system can elegantly explain the lack of cooperativity or directionality observed in individual transporters.^299^

In respect to the PAP–OMF interface our model allows for the first time to tentatively assign a specific role to each PAP conformer within the cycle. As described above in the closed, resting state of the OMF the coiled-coil α-helical hairpins at its tip are stabilized by a network of predominantly ionic interactions, aka “primary gates”, that keep the inner coiled-coil helices (H7/H8) in a tensed supercoiled state relative to the relaxed superhelical trajectories of the outer coiled-coil helices (H3/H4). The disruption of the “primary gates” has been demonstrated to result in a spontaneous relaxation of the super helical trajectories of the “mobile” inner (H7/H8) helices relative to the “static” H3/H4 helices, resulting in dilation of the OMF aperture following an “iris-like” mechanism.^687−689^ This interaction is induced by the PAP–OMF interaction and does not require energy input from the transporter.^720^ Analysis of the positions of the PAP1 and PAP2 in the zero-degree structures of MexAB-OprM (PDB ID 6IOK,^306^6TA6)^270^ and the corresponding AcrAB-TolC^305^ suggests that PAP2 is the main interacting conformer with the OMF-R2 (H7/H8) motif, which is consistent with it being the final recipient (and stabilizer) of its relaxed helical trajectory, the release of which could plausibly be deduced to be triggered by an initial docking of the PAP1. While in the case of MexA and AcrA it is difficult to assign a specific role to each PAP due to their ultimate interchangeability and the quasi-equivalence of the 60° structures, the roles of PAP1 and PAP2 can be established to be distinctly different.

On the example of the TriABC-OpmH system it has been established that PAP1 (TriA) is detrimental to recruitment of the OMF and hence likely makes first contact with it, which is supported by mutagenesis,^802,803^ while the MPD of the PAP2 (TriB) is responsible for the activation of the transporter, which, in light of the latest findings of Glavier et al.,^270^ is likely mediated via the conformational changes that propagate to the “exit-gate”.

We therefore propose that the PAP1 plays the primary role in sensing the drug-bound state T-state of the transporter and the recruitment of the OMF. Due to the differing helical trajectories of the R1 (H3/H4-associated) and R2 (H7/H8-associated) motifs of the OMF, the primary interaction of PAP1 (based on the TriA data) is suggested to be the R1-motif, although in single-PAP assemblies such as MexAB and AcrAB the interface is quasi-6-fold symmetrized, and 60° rotation of the OMF may possibly produce functional complexes as suggested by the existence of the alternative structures (e.g., PDB ID 6IOL,^306^6TA5^270^). In the case of a binary AcrAB complex which is transitioning from an LLL state, the drug binding and conversion on L to T would serve as an allosteric signal from PAP1 toward the OMF enabling its attachment.

**Figure 46:**
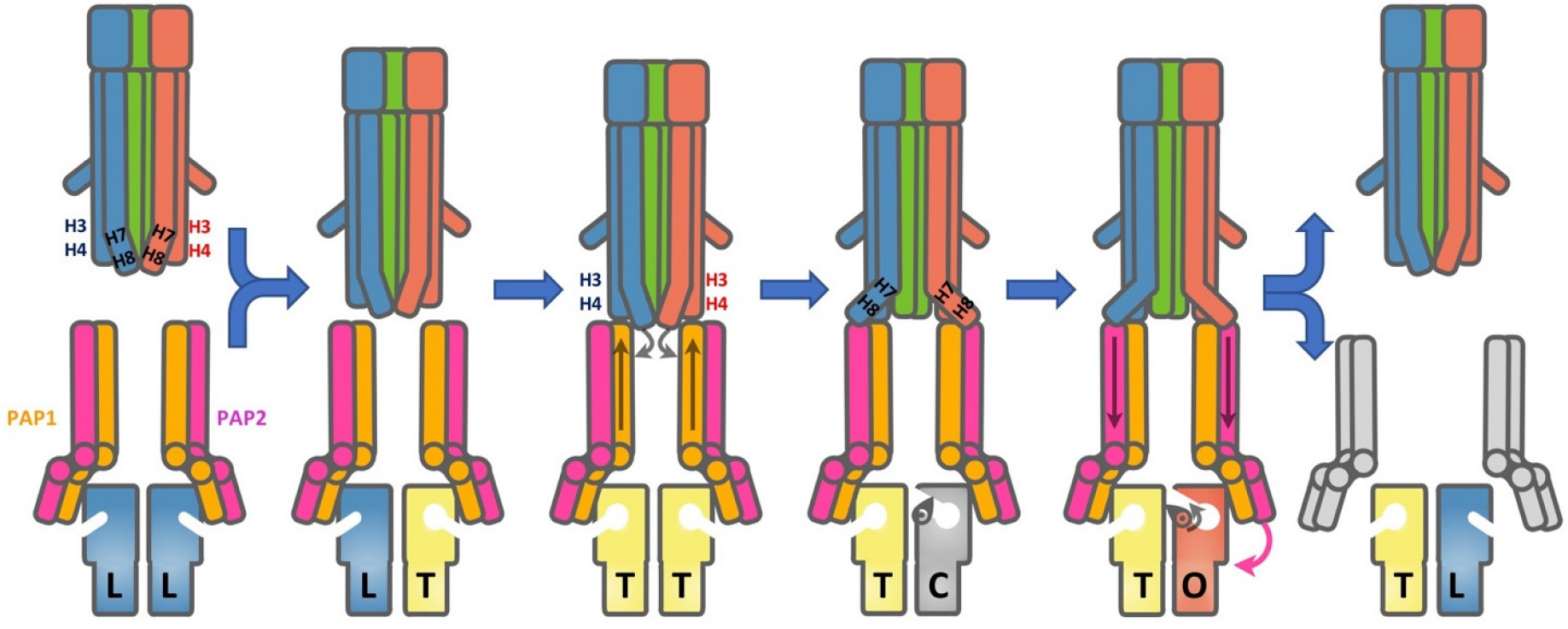
Proposed model for concerted tripartite assembly along sectional views of the OMF-PAP-RND interaction surface. RND protomers are colored according to their adopted conformation (Loose in blue, Tight in yellow, Closed in gray and Open in red). Six PAPs protomers bind as pairs to the RND trimer. The resting, closed OMF trimer interacts with the PAP hexamer (one pair of PAPs is omitted for clarity). Upon interaction with the OMF periplasmic end, PAP1 destabilizes the lock anchoring the H7–H8 helices to the static H3–H4 helices, resulting in a spontaneous relaxation of the superhelical trajectories of H7–H8 helixes as depicted by arrows. Engagement with the OMF is communicated down to the RND via PAP2, allowing the positioning of the MP-domain at the T/C crevice (indicated by the pink arrow), which in turn results in a rearrangement in the underlying PN1 domain leading to the opening the helix gate, denoted by the rotation of the flap symbol, thereby enabling the Closed to Open transition of the protomer.

The initial docking of the PAP1 to the H3/H4 helices of the OMF, which serves as an upward communication signal, triggers at least partial release of the H7/H8-helices, possibly by attacking the interprotomer H8–H4 attachment gate, the full relaxation of which is achieved by breakage of the intraprotomer H7–H4 gate (containing a conserved Y-residue, e.g., Y404 OprM or Y362 TolC) upon subsequent PAP2 docking (see Figure 42D, E and Figure 46). The relaxed trajectory of H7/H8 is then stabilized by the PAP2 which ends up being attached to the tip of H7–H8 (R2 HTH-motif) with its own RLS-motif, as seen in the recently assigned 0-degree OMF-structures (PDB ID 6IOK,^306^6TA6^270^) (see Figure 42B and Figure 46). Indeed, the analysis of the tip-to-tip interactions and the possible starting and finishing trajectories of the H7/H8 helices as seen in the 0-degree structures suggests that these are the only arrangements which are compatible with the cross-linking data presented by Ntreh et al.,^803^ strongly indicating that TriB binds in a compatible fashion to the one presented by PAP2.

### 8.7 RND–PAP Interface and Energetic Considerations

**Figure 47:**
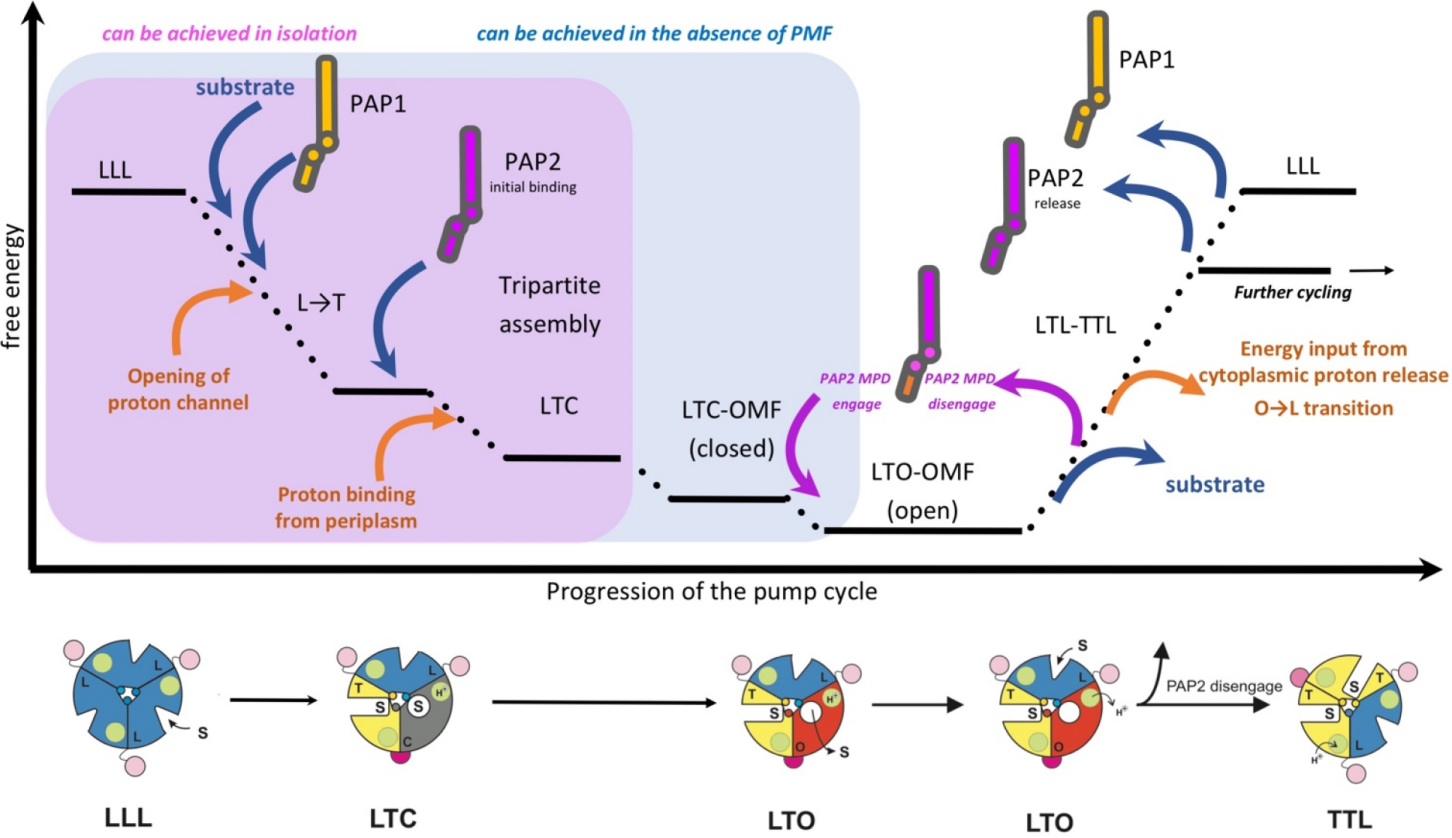
Qualitative energy diagram of the assembly and cycling of the RND-tripartite assemblies taking into account the role of the PAPs and the newly discovered C-to-O transitions. The symmetrical LLL state is suggested to be a metastable high-energy state of the RND trimer and is prone to spontaneous collapse upon substrate binding. The L to C transitions are suggested to be driven solely by the binding energy between the components and can occur in isolated RND transporters, as witnessed, e.g., by the “mono” structures of MexB.^270^ The C-to-O transition does not happen spontaneously, suggesting that the C-to-O is an upper energy transition and is offset by the binding of the PAP2 MPD (represented by the lower part of the magenta protomer being colored in orange). PAP2 MPD cycling provides directionality of the cycle and prevents backsliding of the C/O-to-T which in its absence is suggested to be in an unstable equilibrium. The energy from proton release allows for the upper-energy level O-to-L conformational transition, which enables repeat of the cycle.

Bridging of the RND transporter and the OMF by the PAP does not appear to require energy as the stable complex assembly can be achieved *in vitro*. Bipartite interactions analysis showed tight association between MexA and OprM in the absence of MexB, whereas the expression systems lacking MexA failed to copurify MexB or OprM.^704^ Furthermore, binary interactions between purified proteins probed by surface plasmon resonance (SPR) also suggest a sequential assembly of the RND complex in the absence of energy input,^697,698^ with PAP-RND subcomplexes creating a tight formation which appears to be independent of substrate presence. Consistent with this, pump substrate novobiocin and a broad-spectrum pump inhibitor Phe-Arg-β-naphthylamide (PAβN; MC-207,110) were able to bind to the immobilized AcrB but did not affect interactions between the components of the complex.^698^ Crucially, the pump assembly, including the recruitment of the OMF channel, and its opening were found to be energy independent *in vivo*(719,720) as well. The energy input from cytoplasmic proton release is likely required for resetting the protomer to the initial L-state as evidenced from both *in vivo*(720) and in an isolated controlled system *in vitro*.^705^ Based on the above considerations we postulate that the process from the apo-state (L), over drug-bound state (T), to the release of the drug in the O-state is PMF-independent (Figure 47).

Thus, the only energy involved is the binding of the substrate, plus the binding energy of PAP-association, alongside with and the binding of proton(s) to the transporter, with its occurrence in dependence of their concentrations present in the periplasm. On the other hand, the “reset” of the O conformation into the L conformation is suggested to be PMF-dependent. This might explain why the LTO conformations are observed in isolated protein samples upon structural analysis. The reset of the structure from the O-state to the L-state is suggested to take place upon the substrate release from the drug-binding pocket and the associated release of the proton from D407 and/or D408, which causes a large conformational transition.^352^ Upon proton release, the O conformation returns to the L conformation and the TO interface changes into a TL interface which closes the gap between the protomers, and rearrangement of the RND-surface leads to the disengagement of the PAP2 MPD domain.

Our model suggests that the LLL state of the pump is metastable and does not require additional input of energy to undergo L to T transition other than that provided by the substrate binding. Concomitantly, the allosteric transitions associated with the L-to-T transition upon the substrate binding are transmitted to PAP1, which initiates OMF-recruitment (Figure 47).

The binding of the substrate opens the proton access channel in the T-conformer, and the occupation of the proton relay causes the T to C conversion (as demonstrated by a recent MD study).^808^ This in turn recruits the PAP2 MP-domain to the interprotomer groove, which by acting as a “door stop” or “latch” stabilizes the C-state, preventing backsliding to the T-conformation, and enables the C-to-O transition. While the occupation of the proton relay facilitates this C-to-O transition *in vivo*, it can be achieved in the absence of PMF in the isolated transporters. Indeed, the energy barrier of the T-to-C transition appears to be relatively small, as additional binding energy provided by the interaction of the MBX3132 inhibitor over native substrate appears to be sufficient to overcome it and stabilize the pump in a quasi-symmetric TTT-state, as seen in the PDB ID 5NG5.^305^ The C-to-O step does not occur spontaneously, and in isolation it is likely to represent a higher energy step, which is offset by the binding energy provided from the allosteric association of the MPD of PAP2 with the RND-protomer as suggested by Glavier et al.^809^ In agreement with that a peak force of ∼8 kcal/(mol Å) was required for the indole to overcome the closed gate barrier,^808^ while a smooth energy profile is seen when the gate is open.^810^

In the next step of the cycle the substrate release from C- to the O-state causes the exit gate to collapse, leading to the TM-conformational transition that leads to release of the protons on the cytoplasmic side, which also causes the consumption of the proton gradient. It has to be noted that the MexB-solo incorporated in liposomes^270^ in the presence of its substrate Meropenem was not able to achieve any proton transport, which is consistent with the requirement of PAP2 association for proton release. The collapse of the exit gate, which initiates the O-to-L transition, is also translated to the change of the surface of the PN2 subdomain of the RND-protomer, which lowers the binding affinity of PAP2 to it, leading to its release. Thus, substrate release is sufficient to disengage the molecular “latch” that is the MPD of PAP2, the release of which causes the proton release on the cytoplasmic side, closing the cytoplasmic access (Figure 45, Figure 46, and Figure 47).

The preference of the MP-domain of PAP2 to the substrate-bound T-protomer provides a straightforward control of the pump activity in response to the substrate concentration. However, in the discussed scenario this on–off cycle of PAP2 MPDs is enabled by the β-barrel ring that keeps the tripartite pump assembled through repeated cycles. However, a complete disengagement of the PAPs might be required under certain conditions. It has been previously suggested that a drop in the drug concentrations in the periplasm may drive the adoption of a symmetric LLL-state of the AcrB trimer,^807^ which under the current model (Figure 45) could shift the equilibrium toward pump disassembly. While future research will need to provide a definitive answer to the actual mechanism of disassembly, the following findings may provide circumstantial evidence. The PMF is linked to the pH levels in the periplasm,^811^ and lower periplasmic pH at ∼6.0, which is associated with high PMF, was found to favor pump assembly, while elevated pH at 7.5, corresponding to the decreased PMF, promotes pump disassembly in a PAP-dependent fashion.^698,757,758^ Similarly, Ip et al.^812^ using site-directed spin-labeling EPR demonstrated that AcrA undergoes not just significant pH-induced conformational transitions but also oligomerization. Thus, the PAPs may also play the role of a PMF-sensor for the assembly.

Such interpretation is consistent with the observation of two-liposome reconstituted MexAB-OprM complexes, where substrate transport was associated with a rapid PMF-consumption^813^ followed by a subsequent dissociation of the MexAB and TolC complex.^705^ Furthermore, in *E. coli*, the rapid spiking (∼1 Hz) of the PMF coincided with the efflux of the potential AcrB substrate tetramethyl rhodamine methylester (TMRM)^814^ allowing the suggestion that the transient AcrAB–TolC complex operates in a discontinuous fashion.^807^

In summary, while further functional validation is required to fully confirm the roles of PAP1 and PAP2 at the level of specific OMF-gate opening, this model provides a noncontroversial unification of both PAP–OMF and PAP–RND interfaces, allowing us to rationalize the available data, and furthermore provides a testable platform for future studies that we believe will benefit the wider community in the field.

### 8.8 Tripartite Complexes of HME-RND Family

The active role of PAPs in the efflux pump assembly has also been demonstrated in the HME-RND transporters, which are closely homologous to the drug-efflux associated HAE-1 family discussed above and are thus anticipated to form similar tripartite assemblies.

One of two main branches within the HME-family specializes in pumping out divalent ions, e.g. CzcABC, CznABC, and NccCBA involved in Cd^2+^, Zn^2+^ and Ni^2+^ and Cd^2+^, Co^2+^ and Ni^2+^ detoxification respectively^45,67,815,816^ and with ZneCAB from *Cupriavidus metallidurans* CH34 being a relatively well-studied system.^46,358^ In contrast to the HAE-1 RND pumps discussed above, the ZneA transporter does not appear to undergo dramatic LTO conformational transitions, which can be expected as its ion-cargoes do not require such major reorganizations in order to pass through. There is also a strong indication that unlike other RND transporters discussed here, it may actually be capable of moving the Zn^2+^ from the cytoplasm directly to the extracellular space, in an electrogenic fashion suggesting a proton to Zn^2+^ ratio of greater than 2:1.^358^ However, little is currently known of the higher order complexes with its participation, although, as mentioned earlier, its cognate PAP ZneB presents an architecture very similar to the PAPs associated with the HAE-1 type RND transporters. ZneB, however, has a well-defined specific metal-binding site, and the crystal structure revealed Zn^2+^-ion coordinated by H220, H284, E328, and a water molecule at the interface between the β-barrel and MP-domain,^46^ yet again highlighting the important substrate-binding role for the PAPs.

The active role of PAPs in the assembly of the tripartite efflux complexes is probably most clearly demonstrated on the example of the Cu^+^/Ag^+^ tripartite transporter CusCBA,^359,702^ where CusA is an RND-pump belonging to the HME-family, CusB is a PAP, and CusC is an OMF.^817^ The system is complemented by a periplasmic chaperone, CusF (which is homologous to the C-terminal domain in SilB),^764^ whose role is to transfer the metal ions from the transmembrane ATPase CopA to CusB.^818^ This CusB–CusF interaction functions as a switch for the entire Cus-efflux system and facilitates the transfer of copper to the CusA component.^360^ Binding of copper is coordinated by a relay of conserved methionine residues, which stretch from the membrane-proximal domain to the helical hairpin of CusB, and importantly, binding to these sites causes CusB to undergo substrate-linked conformational changes.^768^ Electron paramagnetic resonance (EPR) distance measurements revealed that CusB exists as a dimer in solution, which undergoes major structural changes associated with Cu^+^ binding,^819^ resulting in a compactification of the apo-structure, to produce a “holo-CusB”. Furthermore, CusB has been demonstrated not just to be the key mental sensing element in the pump but to actively drive pump its assembly in response to metal stress.^820^ CusB switches the pump on, once excess metal ions are detected, but once the periplasmic ion-concentration goes down, CusB transfers the bound ions back to the periplasmic chaperone CusF, leading to deactivation of the pump and maintaining copper homeostasis.^820^ Thus, through its interplay CusF, CusB not only plays a central role in substrate binding and pump activation but also functions as a metal sensor controlling the whole system and is directly responsible for complex assembly.

### 8.9 Could the Structure of the Metal Pump CusBA Suggest a Conformation for a Pre-engagement Apo-State for the Transporter–PAP Complex?

Crystal structures of the binary CusBA complex^359^ revealed a hexameric assembly of the PAP forming the typical β-barrel and lipoyl domain gaskets on the top of the transporter; however, CusB forms a clearly delineated trimer of dimers, with each pair being relatively loosely associated with each other. Notably, PAP2 is fully engaged and resolved in the structure, and the electron density is particularly good around the N-terminal segment of PAP2 which appears to be in contact with PAP1 (PDB ID 4DNT,^610^3NE5^359^).

The positions of the different PAP protomers, and especially their respective MPDs, are diverging from the one seen in the cryo-EM structures of the HAE-1 assemblies. The orientation of the MPD domain of PAP2 in the CusBA complex is notably different from that in PAP2 seen in HAE-1 assemblies, where it is firmly associated with the interprotomer crevasse and PN2/DN domains of the RND protomer. The PAP2 features an extended N-terminal tail (in PDB ID 4DNT it is resolved from residue 79 to 96), making extensive contact with the surface of the RND transporter, engaging with the PC1 and PN2 domain of the protomer in an unfolded state. Notably this N-terminal tail of PAP2 also contacts PAP1 directly, making specific contact with the equivalent N-terminal tail of PAP1, which however presents a strikingly different configuration, specifically in the contact range (residues 84–93), where if it forms an α-helix, that contrasts the unfolded region of PAP1.

Importantly, the helical trajectories of the α-hairpins of CusB are pointing away from the center of the pump axis, without participating in helical-bundle tubular formation (PBD ID 3T53).^610^ Along with a loose association of the lipoyl domains, such a hairpin orientation of the PAP is inconsistent with sealing of the OMF-channel.

While this may be explained away by functional differences between transporter types, several points suggest that the conformation trapped by the crystal structure of the CusBA complex is one of a pre-engagement LLL, and indeed the complex is trapped in a symmetrized conformation in all the available structures, even those which are supposedly in an extrusion state (e.g., PBD ID 3T53).^610^ This symmetrized, pre-engagement state may explain the hairpin domain orientations, as after all, effective sealing of the gasket between the OMF and the short hairpins of the CusB is even more important as it only expels Cu(I) ions, and any leakiness would be nonpermissible.

**Figure 48:**
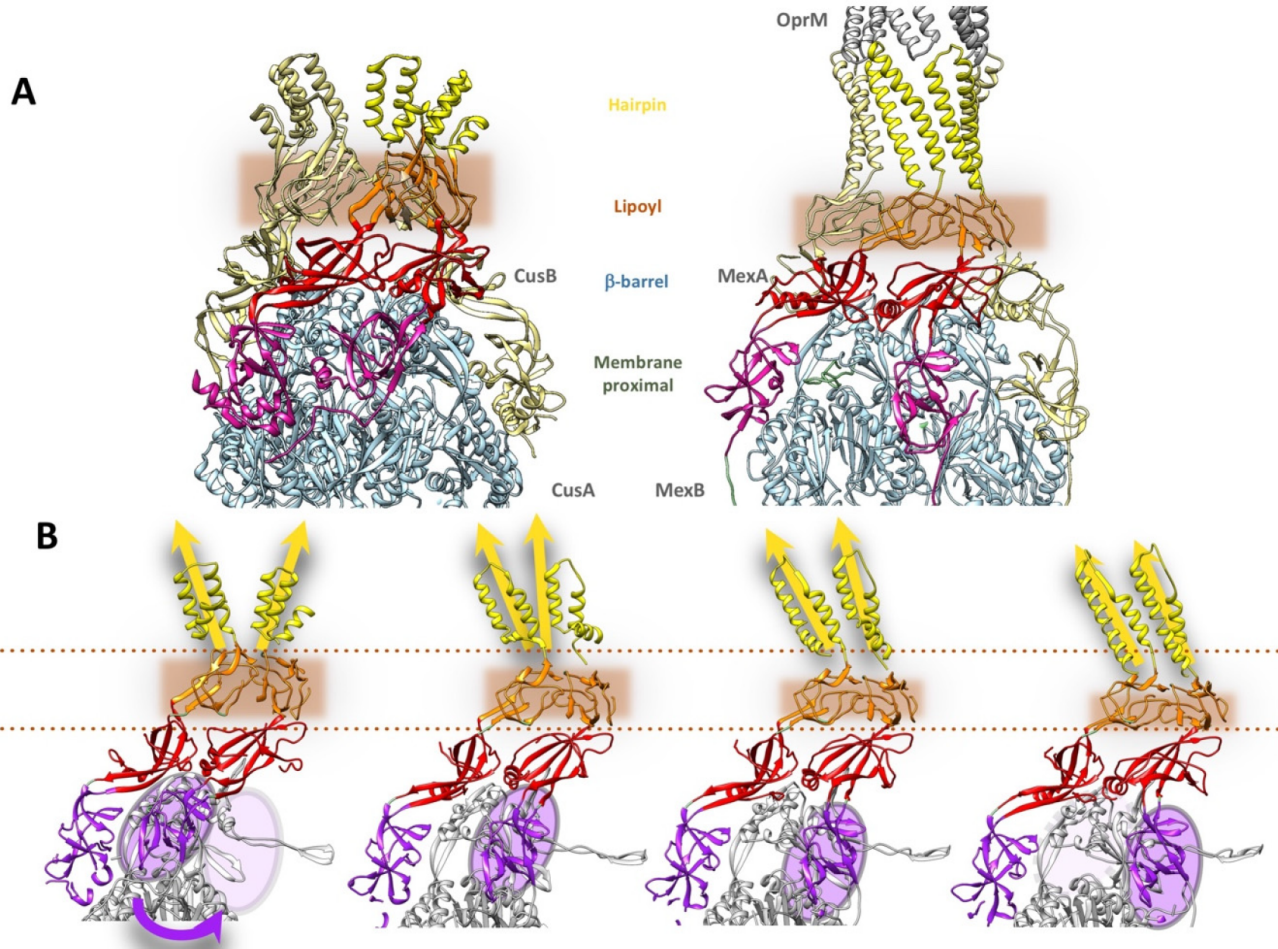
Comparison of the binary CusBA complex and MexAB-trio suggests a possible pathway of conformational reorganization of the PAPs upon engagement with the OMF. (A) Crystal structure of the CusBA subcomplex as seen in the PDB ID next to the cryo-EM MexAB-OprM structure (PDB ID 6TA6), showing significantly different orienations of the helical hairpins and the difference in packing lipoyl domains of their PAPs. Also, while PAP1 conformer binds at the approximately equivalent interface of the RND surface, the PAP2 conformer, and particularly its MPD domain, appears divergent. (B) Several snapshots from a molecular morphing movie illustrating a possible transition from the OMF-free state to the OMF-engaged state, suggesting possible reorganization of alignment of the helical bundle between the two PAP forms (shown in yellow arrows), as well as the proposed dislocation of the PAP2 MPD within HAE1-systems, which may reflect the engagement of PAP2 in the apo- and OMF-engaged state of the complex.

A speculative morph of the PAP1/PAP2 transitions between the ones observed in the CusBA complex and the MexAB trio complex^270^ is provided in Figure 48, and the associated video can be found as Supporting Information. The morph highlights the realignment of the hairpin vectors upon engagement of the incoming OMF (shown as yellow arrows), allowing effective sealing of the efflux conduit. Second, the discrepancy of the position of the MPD domains of the respective PAP2 protomers between the HME (left) and HAE1 (right) systems is suggestive of possible structural changes during the peristaltic cycling experienced by the HAE1 transporters, which appears to be lacking in the HME family. It has to be noted that there is no experimental suggestion of unfolding of the hairpin of the HME-PAPs during such a cycle and that a local variation of the height of the periplasmic space could not be excluded.

### 8.10 Additional Level of Regulation of RND Pumps May Be Provided by the Transmembrane Modulators of the AcrZ-Family

**Figure 49:**
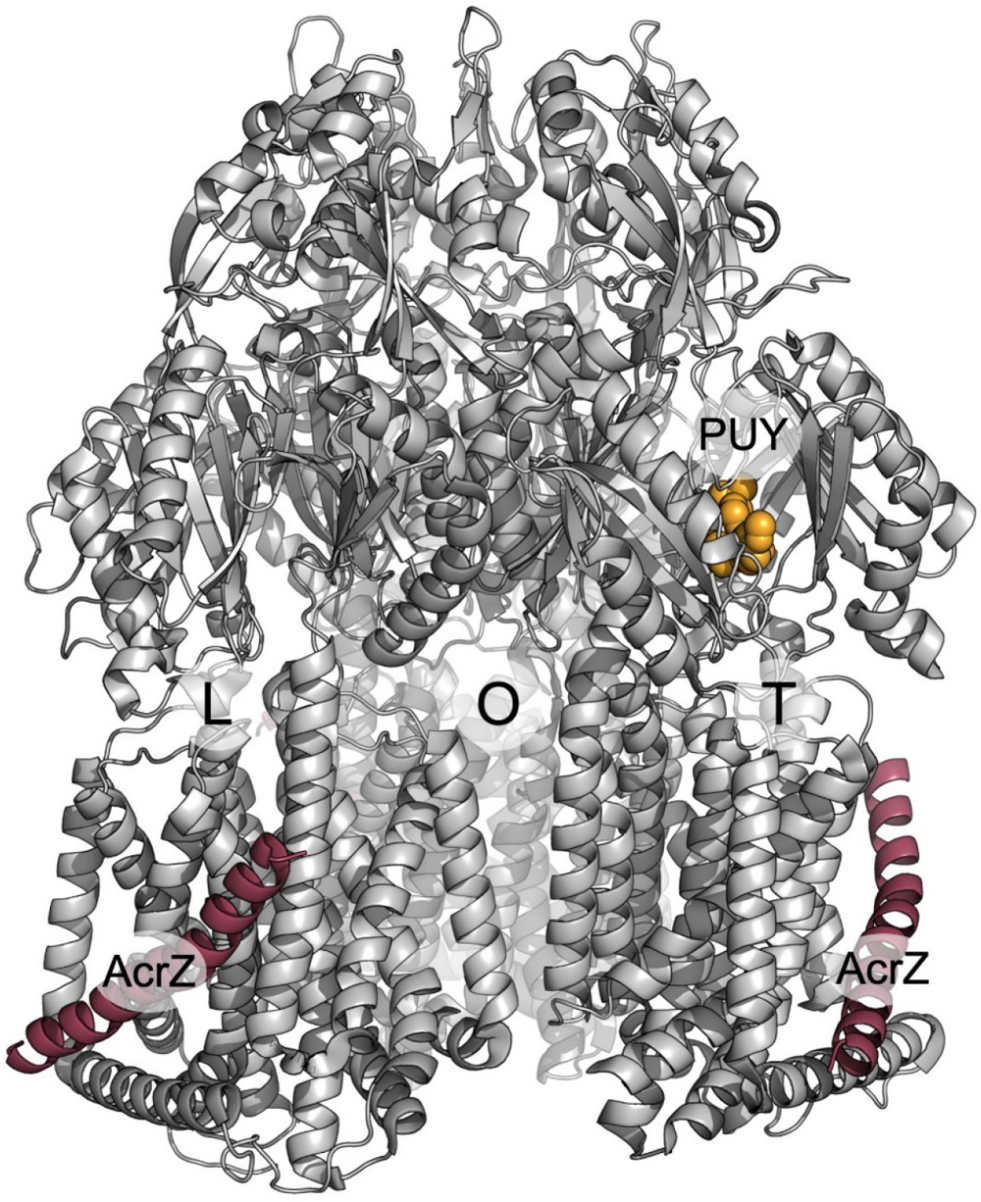
AcrBZ co-structure. The asymmetric AcrB trimer (gray cartoon) with the three protomers in the L (loose), T (tight), and O (open) conformations. The auxiliary transmembrane modulator AcrZ (raspberry cartoon) is located at the surface periphery on each of the AcrB protomers at the transmembrane domain. The X-ray structure display (PDB: 5NC5) was solved in complex with DARPins (not shown) and puromycin (PUY, orange spheres).

A fourth transmembrane component is sometimes present within the RND complexes, e.g., YajC^283^ or AcrZ.^821^ These small proteins are entirely α-helical and bind the transporter within the inner membrane, packing against the TM-helix bundle (Figure 49) in a fashion similar to the γ-subunit of the SecY-complex.^283,287,305^ These proteins appear to be nonessential, playing a modulatory role, possibly changing the efflux profile of the pump in response to environmental stimuli.^283,821^ Recent cryo-EM structures of the AcrBZ-complex in lipid environments and MD studies indicate that the lipid environment affects the packing of the AcrZ on AcrB, resulting, e.g., in preferential interaction of the cardiolipin with AcrBZ. The removal of AcrZ in combination with cardiolipin deficiency increases the bacterial sensitivity to chloramphenicol,^822^ suggesting that the combination of AcrZ and the lipid environment works synergistically to allosterically modulate AcrB activity. Furthermore, the interaction has also been seen to differ between the different conformer states of the RND transporter. The role of these small proteins remains unclear, as they are nonessential and are lacking in most studied RND systems, including the MexAB-OprM.

### 8.11 Functional Assays in Artificial Membrane Environments Provide Additional Insights into the Regulation of the RND Transporters and the Role of the Lipid Environments

Understanding of the molecular basis of pump function and especially the finer modulation and regulatory aspects of the RND tripartite systems requires the use of membrane mimics that enable their characterization in physiologically relevant environments. Methodologies for the structural study of detergent-free membrane protein stabilization involve scaffolding of membrane fragments around the proteins of interest using engineered lipid-avid peptides (nanodiscs), fatty amines, or styrene-grafted amphipathic polymers (amphipols or SMALPs, respectively).^823−825^ All these chemical tools allow for a more physiological environment around the protein. Lipids have an effect on the activity of membrane proteins because of the so-called “bulk” effect that reflects the physical properties of the membrane: bilayer thickness, physical state, curvature, and surface tension can significantly influence the behavior of membrane proteins. For instance, as membrane protein secondary transporters exploit the electrochemical membrane potential to energize the transport of their substrates, the composition of the membrane is obviously crucial. In addition, there are also increasing clues for specific phospholipid–protein interactions where bound lipids act as chemical partners with a prominent role of the lipid headgroup. Overall, the effect of the membrane composition on the function of bacterial efflux pumps has never been systematically explored, but emerging evidence that it plays a significant role is described below. A major drawback is the use of detergents, and it is questionable whether the structures determined in such an environment are representative of what is found *in vivo*. It has been suggested that in some cases, the use of detergent can lead to artifactual structures and to their misinterpretation.^826^ Youzhong Guo realized that the structure of D407A AcrB obtained in detergent^326^ was significantly different when obtained by cryo-EM after reconstitution into polymer-wrapped lipid particles.^330^ Compared to the wild-type protein, the AcrB D407A mutant in detergent shows a significant shrinkage of the central cavity that was interpreted as a mechanistic transport feature.^326^ The narrowing of the central cavity turned out to be impossible in the presence of lipids because of steric constraints. Following similar lines, it was shown that the affinity of AcrB for rhodamine 6G (R6G), determined by fluorescence polarization, is 100× higher in SMALPs^825^ than in detergent.^827^ As further evidence for the importance of lipids in maintaining a functional assembly, Qiu reconstituted the AcrB transporter also in SMALPs and cryo-EM experiments are conducted on these AcrB nanoparticles surrounded by lipids from the *in vivo E. coli* membrane. The resulting structures (*R* = 3.2 Å) show a trimer of the AcrB transporter with additional densities in the transmembrane part of the protein. A lipid patch within the central cavity of the AcrB trimer and isolated lipids around the transmembrane domains that may belong to the lipid belt were described.^330^ This is the first time that such a patch structure is described. It is proposed that this lipid patch is directly involved in the transport mechanism of AcrB by allowing the harmonization of the trans-conformational LTO changes occurring within the three protomers of AcrB. The membrane patch consists of 24 lipids modeled as phosphatidylethanolamine (PE),^330^ the major lipid in the *E. coli* membrane.^828^ Molecular dynamics has been performed on a membrane representative of the one mentioned above with AcrB inserted in this membrane. At the beginning of the simulation, the transporter only interacts with PE molecules; the CL and PG molecules are far away from the protein. During the simulation, the latter 2 lipids come closer to the protein suggesting a role of PG and CL in the stabilization of the AcrB trimer.^822^ Thus, the membrane patch would consist of a more complex mixture than just PEs, with the addition of the lipids PG and CL. The role of PE was also revealed thanks to X-ray structures of the AcrB complex in complex with fusidic acid. Both fusidic acid and DDM were identified at a TM1/TM2 groove of AcrB that was postulated to be an access path for carboxylated substrates. The possible presence of physiological phospholipids at this site was confirmed by performing ensemble docking calculations using structures of AcrB as well as molecular dynamic simulations of the protein embedded in a model POPE bilayer.^334^

Mass spectrometry has developed significantly over the past decade and allows the identification of specific interactions between a membrane protein and a phospholipid which are now maintained in the use of this method.^829,830^ This technique could be envisaged to study AcrB in SMALPs and its lipid composition in a very precise way. The latest advances even make it possible to study the vesicles of the inner and outer membranes of *E. coli* and determine their protein components and associated lipids.^831^ More generally it is believed that it is important to consider the endogenous membranous environment as a pool of potential regulators, local effectors at the membrane, or allosteric ligands. Of foremost importance, AcrZ is a transmembrane protein that was shown to potentiate the activity of AcrAB-TolC against some antibiotics, but the molecular explanation for this effect is lacking.

*In vitro* proteoliposome assays have been shown to be excellent and tunable tools for the characterization of transporters at the molecular level. They offer the possibility of studying purified proteins isolated from the complexity of *in vivo* systems, without pleiotropic and off-target effects, which complicate measurements and interpretations from experiments performed in whole cells. In addition, they allow for a compartmentalization of the protein and hence make it possible to tackle the difficult challenge of determining their catalytic parameters. The path toward the successful reconstitution of a tripartite pump is interspersed with many pitfalls, and several laboratories have contributed to reach a sensible framework to reach this goal. The milestones, major results along this path, and possible improvements are reviewed in refs (832 and 833). For a long time, protocols were based on the reconstitution of the RND pump in the presence or absence of its periplasmic adaptor. As major inputs in the understanding of efflux pumps, reconstitution assays demonstrated that the PAP is mandatory for active transport^311,706^ probably because its presence is required to make the substrate/proton antiport perfectly coupled.^707^*In vitro* studies also allowed a better understanding of the path experienced by the substrate through the transporter^311,354^ or the possible cross reactivity between unrelated pumps.^794^ Ultimately, such methodologies should eventually allow measurement of orders of magnitude of substrate affinities and rates of transport. In their system, Helen Zgurskaya and Hiroshi Nikaido relied on the use of fluorescent phospholipids which are trapped by protein-free acceptor vesicles upon transport. When known substrates of AcrB were added to the test buffer, the efflux of fluorescent phospholipid was inhibited. Measurements of competition of the binding at the equilibrium of the fluorescent lipid by various potential substrates (antibiotics, bile salts, etc.) resulted in IC_50_ values in the order of ten micromolar.^706^ Reconstitution of another RND, CzcA from the efflux pump CzcB(2)A, responsible for heavy metal resistance in *Ralstonia spp*.,^815^ yielded for the first time a rate constant of 385 s^–1^ and a sigmoidal kinetics with a Hill coefficient of 2. However, caution should be taken regarding the physiological relevance of these data because the reported Michaelis–Menten constant (of 6.6 mM) seems to be abnormally high. The overall order of magnitude for the velocity of transport is a matter of heated debate: Aires and Nikaido concluded that ∼0.3 protons are consumed per trimer of AcrD and per second,^311^ and by contrast, in another study, EtBr transport by MexB gave rise to an impressive turnover rate of 500 s^–1^ based on a number of pumps previously estimated by immunoblotting methods. In 2015, it was possible for the first time to measure *in vitro* the transport by the MexAB-OprM efflux pump of *P. aeruginosa* entirely reconstituted in proteoliposomes.^813^ In this setup, a functional reconstitution of the MexAB–OprM system made it possible to trace the respective transport of a fluorescent substrate and protons via a pH-sensitive fluorescent reporter. This system promises insight into the largely unanswered question of H^+^/drug stoichiometry, substrate specificity, and measurement of the catalytic constants of the various pumps.

## 9 Tripartite Assembly of MacAB-TolC

The lack of clear substrate membrane access channels, the very narrow TM-helical bundle, and the reported involvement in the transport of Sec-dependent peptide cargoes^154,548,550^ strongly imply that MacB operates differently from the canonical type IV ABC transporters and floppases. The latter operate on a two-step alternate access model, providing transmembrane shuttling either from the inner-leaflet of the cytoplasmic membrane or directly from the cytoplasm,^390,465,524^ while the cargo of the MacB/FtsX transporters is acquired from the periplasm only. In that respect there is a functional overlap of the family with that of the tripartite RND transporters, which are suggested to work as a universal “second stage” supplementing the single-component transporters that remove the xenobiotics from the cytoplasm.^834^

A reconstruction of the complete *E. coli* MacAB-TolC pump produced from docking of MacB into a hybrid cryo-EM map with varying local resolution (from 3 to 8 Å), derived from the analysis of the fusion-stabilized *Ec*MacB-MacA MPD-domains and of a higher resolution disulfide-stabilized MacA-TolC subcomplex, has been reported by Fitzpatrick et al.^272^ The assembled structure revealed MacB:MacA:TolC protomers in a 2:6:3 stoichiometry consistent with the earlier biochemical and biophysical essays.^573^ The complex presents dimensions comparable to the ones observed in the RND-based assemblies measuring some 320 Å along its long axis (membrane to membrane) to span the whole periplasm.^272^

The PAPs associating with the MacB-FtsX like transporters, such as MacA, similar to the PAPs of RND systems, have a strong propensity to form hexamers in isolation,^759^ that are stable under native-MS conditions^573^ and can be readily crystallized.^571,737,746^ Functional studies have shown that MacA has a role in regulating the function of MacB, increasing its affinity for both erythromycin and ATP.^573^ While the MacA–MacB complex is formed with a nanomolar affinity even in the absence of ATP, the affinity of interaction further increases in its presence,^835^ with the PAP acting as a switch for allowing ATP-hydrolysis by MacB.^836^

Studies by Tikhonova et al.^743^ demonstrated the functional dependency of MacB on MacA, showing that after reconstitution into proteoliposomes, the ATPase activity of MacB was strictly dependent on MacA. The catalytic efficiency of MacAB ATPase was more than 45-fold higher than the activity of MacB alone. Both the N- and C-terminal regions of MacA were essential for this activity. MacA stimulated MacB ATPase only in phospholipid bilayers and did not need the presence of macrolides suggesting that MacA forms a functional subunit with the MacB transporter.^743^

Notably, the lipoyl domains of the PAPs in these assemblies form tight hexameric gaskets, similar to the ones formed by the PAPs in isolation^737^ with an extended flexible loop donated from each protomer creating a “gating ring”,^272,571^ which is suggested to prevent backflow, favoring unidirectional substrate efflux in an outward direction (Figure 35C). In the hexameric structure of *E. coli* MacA, the lipoyl domain loops (G56-L72; T199-I214) protrude into the cavity delineated by the assembled hexamer, providing a constriction of the lumen, which is narrowest at residue N209, which in the stand-alone structure (PDB ID 3FPP)^737^ is effectively sealed (Figure 35C in section 7.3).

Similarly, in the PAP Spr0693 (PDB ID 5XU0), which pairs with the Gram-positive MacB-homologue Spr0694-0695, the hexameric α-helical barrel has an inner diameter of ∼31 Å. A gating ring is formed by the flexible loops from the lipoyl domains (N211–V224), with six G220 residues at the innermost site restricting the internal diameter to around ∼7 Å.^571^

The β-barrel and MP-domains of MacA mediate the interaction with the periplasmic domain of MacB, in agreement with the reported functional data^60,573,836^ and the observation that the MP domain is required for MacA and MacB to associate in solution (with nanomolar dissociation constant).^836^

**Figure 50:**
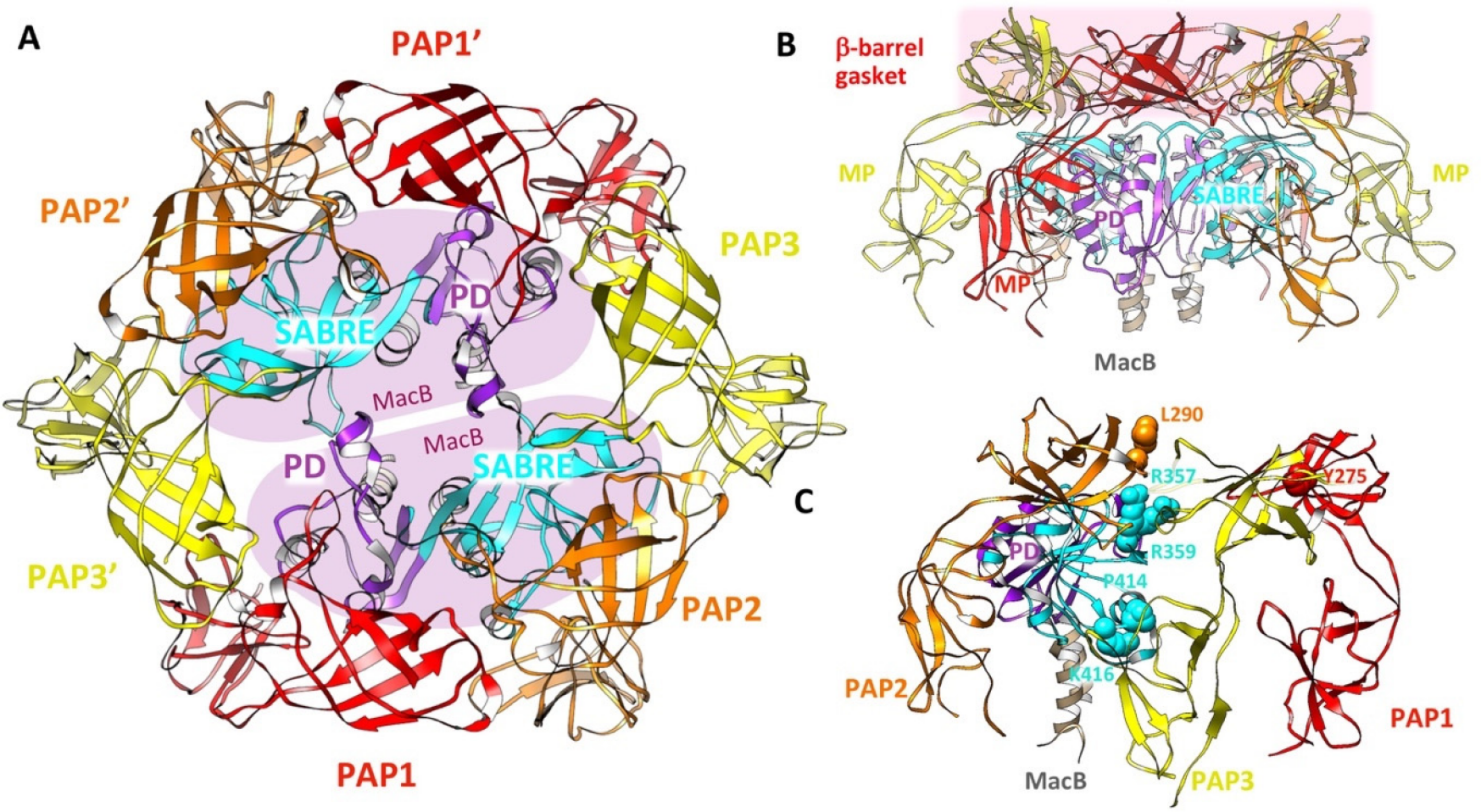
MacAB-association highlighting the differential binding of PAP protomers based on PDB ID 5NIK.^272^ Different PAP protomers can be named PAP1-PAP3 based on their interaction with the MacB transporter in an analogy to the RND transporter complexes. PAP1 can be seen as an intraprotomer relative to the transporter, while PAP2 interacts exclusively with the SABRE subdomain of the same transporter protomer. PAP3 appears to occupy an interprotomer position extending over the cleft between the neighboring transporter protomers. (A) Top view looking down at the interprotomer interface of MacB. (B) Side view showing the position of the β-barrel gasket of the PAP hexamer. (C) Different PAP conformers make inequivalent contacts with the crown of the transporter, and PAP3 in particular, which is interprotomer-bound, is loosely associated with the complex.

In the reconstructed tripartite assembly,^272^ three MacA MPDs are found to bind to a single MacB protomer, with one MPD contacting the PD-sub domain and two contacting the SABRE subdomain. Located above the PCD-crown of the transporter, the β-barrel domains of MacA form what we refer to as a head-to-tail hexameric “gasket”-ring, which docks to the periplasmic domain of MacB (Figure 50 and Figure 40—ATPase rings). Stabilization of this β-barrel gasket-ring is mostly due to the interprotomer interactions of three residues (which in the *Ec*MacA correspond to T293, Y275, and E231), that are strictly conserved across the MacA-family indicating a common mechanism.^737^

The β-barrel domains of the three MacA protomers make nonequivalent contacts with the PCDs of the transporter, which could, in an analogy to the PAP1 and PAP2 discussed in the context of RND transporters, be named PAP1–PAP3, respectively (Figure 50). Of these, PAP1, similar to the PAP1 in the RND assembly, makes intraprotomer interactions only relative to the transporter (e.g., using the chain identifiers from the PDB ID 5NIK).^272^ PAP1 could be assigned to chain D, and both its MP-domain and β-barrel domain interact with the PD-base of the PCD domain of the MacB (chain J), while PAP2 (chain F) interacts exclusively with the SABRE domain of the same transporter protomer (chain J), and the last, PAP3 (chain G), appears to occupy an interprotomer position extending over the cleft between the neighboring transporter protomers. However, in contrast to the tight association of the RND–PAP2 with the interprotomer space in the RND transporters, the “inter-protomer” PAP3 in MacB appears to make very few direct contacts with the underlying MacB using its β-barrel domain, and its association is mainly maintained via lateral contacts with the other PAP mediated via the β-barrel domain loops. Similarly, while the PAP3 MPD does contact the SABRE subdomain of the PCD; this contact, at least in the conformation observed in the cryo-EM structure, is restricted to a single loop. Such limited contact of this PAP3-protomer with the transporter makes this site particularly susceptible to allosteric modulation and may provide a natural switch for assembly/disassembly of the complex.^272^ Furthermore, the asymmetric interactions of the MPDs as well as the β-barrel domains of MacA with the underlying transporter result in distortion of the hexameric β-barrel ring relative to the shape formed by the MacA MPDs in isolation, allowing it to accommodate the 2-fold symmetry of the MacB PCDs.^272^

In an organization which mirrors that observed in PAPs participating in the RND transporter assemblies, the lipoyl domains of MacA form an additional hexameric ring that stacks above the β-barrel gasket but does not contact MacB. The self-association of the “lipoyl-gasket” clearly plays a role in stabilization of the whole PAP hexamer, as can be seen in the example of DevB–DevAC interaction.^206^ From the lipoyl domain-gasket up, the hairpin arrangement of the PAPs from the MacA-family and that of the AcrA-family follow similar trajectories and present a unified interface to the OMF, which is further supported by the aforementioned chimeric-protein studies, swapping the hairpins between MexA and MacA (PDB ID 4DK1).^751^ Consistent with this the MacA-family also shares the conserved RLS-motif at the tip of their α-hairpin.

Thus, the arrangement of PAPs around the MacB-dimer differs substantially from the organization of the RND transporter complexes, showing a much-lighter engagement with the transporter, and the PAP–MacB interactions are restricted to within the same transporter protomer, with any interprotomer contacts being mediated by PAP–PAP interactions instead. This loose association, along with an exposed proposed substrate location site and the apparent lack of enclosure provided by the two stalk helices, poses some questions regarding sealing of the assembled complex and cross-membrane communication with the NBDs, which are suggested to provide remote allostery to the complex.

**Figure 51:**
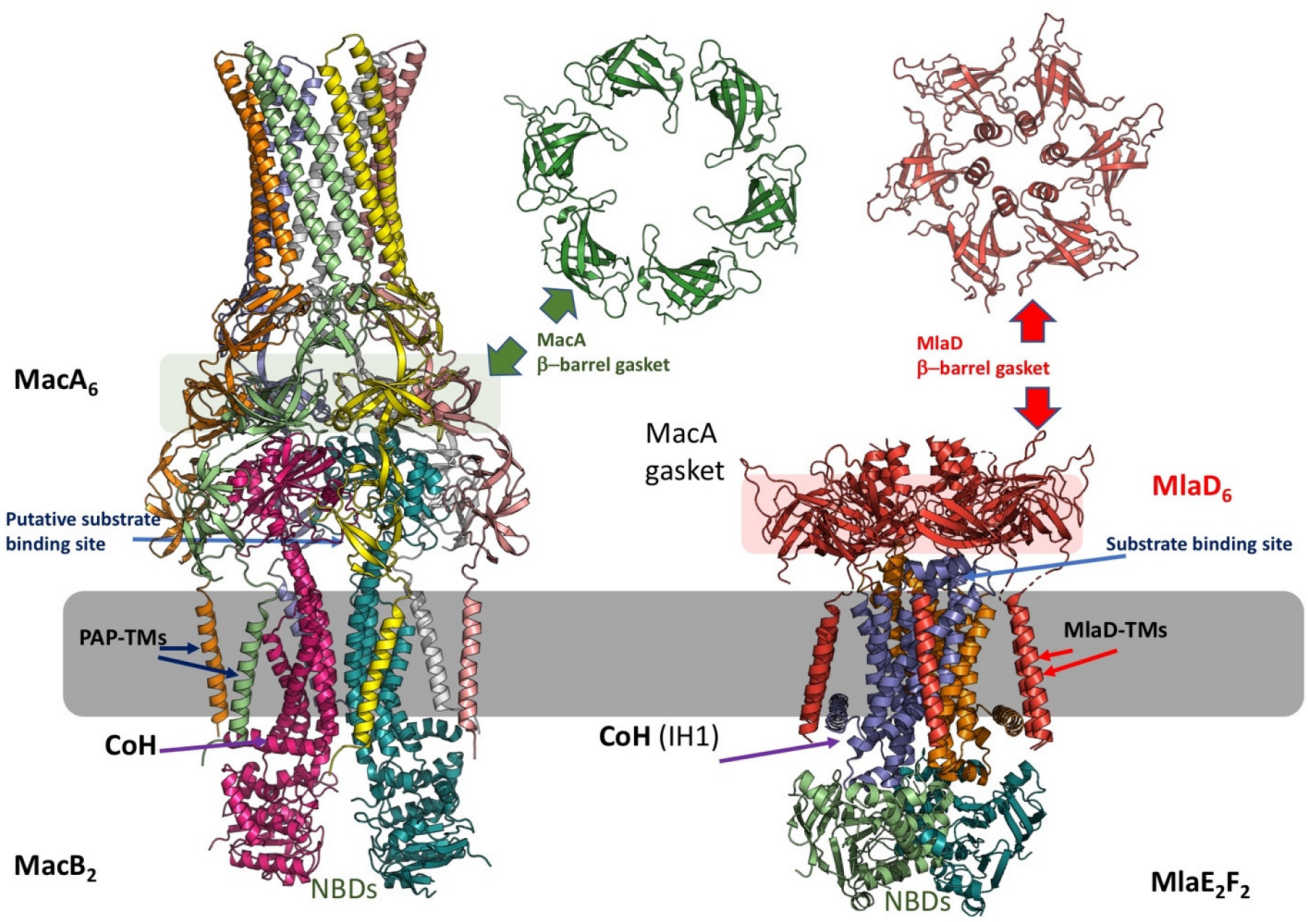
Comparison of the assembly of MacAB with the MlaEF-complex reveals a common organization. Both transporter complexes are stabilized by a hexameric β-barrel gasket (colored green and red), formed of their respective periplasmic partner proteins, which, although structurally nonhomologous, provide an identical solution to binding to a dimeric transpoter interface. Left, a homology model of the MacAB subcomplex based on the PDB ID 5NIK, taking into account the transmembrane portions of the MacA which were unresolved in the cryo-EM structure, indicates that these are of sufficient length to reach the connecting helix (CoH) of MacB and could provide additional allosteric coupling across the membrane, as established for the MlaEF-D complex (on the right, experimental structure (PDB 6XBD)).

An unexpected analogy to the PAP-MacB communication may be found in the function of the maintenance of the phospholipid asymmetry (Mla) MlaFEDB complex, the structure of which has recently become available (PDB ID 7CGN; 7CH0; 7CGE; 6XBD).^437−439^ In the sections above we mentioned the close topological connection of the MacB (and other type VII ABC transporters) with the new MlaE-family, which is suggestive of common operational mechanisms. It is thus even more notable that the same stoichiometry of 2:6 as in the MacB–Mac complex is also observed in the complex between the transporter MlaEF and the periplasmic protein MlaD, which belongs to the mammalian cell-entry (MCE) family, which while phylogenetically not connected to the PAPs,^837^ forms analogous hexameric assemblies with participation of their respective β-barrel domains (Figure 51). Similar to MacA–MacA interaction, three MlaD protomers associate with a single MlaE protomer.

Within this complex, a striking arrangement of TM-helices emanating from MlaD can be seen in the MlaED complex, where it is tempting to suggest the N-terminal helices of MlaD are facilitating the substrate transfer and activity of the transporter.

The TM-helices of different MlaD protomers make distinct contacts with the TMD and connecting helix (CoH) of the transporter MlaE (also referred to as elbow or interfacial helix-1 (IF1),^439^ with the TMs of D1- and D2-protomers of MlaD forming close hydrophobic contacts with the elbow CoH of MlaE, while D3-protomer packs closely against the TM1 and TM3 of MlaE. Mutagenesis of the respective contact residues in MlaE rendered the bacteria sensitive to the treatment of SDS/EDTA,^437^ and correspondingly, mutations in the MlaD-TM domain also rendered the transporter nonfunctional.^439^ Furthermore, truncations of the coupling helix (IF1) of MlaE also abolish transporter function, highlighting the importance of this structural element. The existence of the CoH is a notable feature of MacB, and similar to MlaE, it only has a single coupling helix, with its NBDs being connected to only one porter domain subunit, which suggests a similar mechanism of action. Intriguingly, in the absence of nucleotide MlaE adopts an outward facing conformation.

While the TM-domains of MacA are not resolved in the cryo-EM structure of the complex,^272^ modeling the full-length MacA (see Figure 51) suggests that, similar to the MlaEF–MlaD interaction, these TM-helices could reach and plausibly contact the CoH and/or intramembrane helices of MacB, thus providing direct coupling of the conformational transitions in the periplasm to the transporter and its NBDs. Consistent with such a model and interpretation, while MacB is able to interact with a MacA, which lacks the TM-helix,^573^ this TM-helix is required for activation of the ATPase activity *in vitro*, and its deletion obliterates the MacAB-mediated macrolide resistance *in vivo*.^743^ This also holds true for the MacAB homologues from the Gram-positive organisms, as the ATPase activity of the Streptococcal Spr0694–0695 MacB-like transporter was activated by full-length, but not by the N-terminally truncated version, of the cognate PAP Spr0693 when reconstituted in proteoliposome,^571^ suggesting that the intramembrane communication between the PAP and the transporter is essential for the general function of the MacB-like assemblies and not restricted to tripartite complexes with their participation.

Furthermore, evidence from the related DevBAC system in *Anabaena* shows that N-terminal cytoplasmic domains in the PAPs of MacB-group play an important role in activation of the ATPase activity.^206^ There, a variant of the PAP DevB, lacking 22 N-terminal residues completely abolished the recognition of the glycolipid substrate by the DevAC ATPase despite not having an impaired binding to the transporter. Similarly, the DevB homologue, All0809, is not able to promote a substrate-dependent reaction of the ATPase activity of DevAC, although it binds to DevAC,^555^ suggesting a further layer of the underappreciated PAP role in the process may be present. Importantly, besides the wild-type reference DevB, none of the PAP variants harboring hairpin deletions tested (with small exception) could mediate the substrate-dependent ATPase activity,^206^ suggesting that formation of the hexameric PAP-assembly is critical for the activation of the pump, which is in agreement with an earlier-reported PAP hairpin-targeting mutation causing similar defects.^559^

The above observations suggest that full understanding of the functional cycle of the MacB-based pumps could not be achieved by looking at the transporter in isolation, as similar to the MlaE_2_F_2_D_6_ which presents a complex 10 + 6 TM-assembly, the MacB_2_–MacA_6_ complex should be interpreted as an integrated 8 + 6 TM-system, with the MacA-protomers forming an active part in the presentation of the cargo as well as communication of allosteric changes across the membrane while also providing an additional level of cooperativity.

While such communication would be analogous to the vertical communication from the PAPs to the TMD of the RND transporters discussed in the preceding section, its trans-membrane nature involving the MacB-CoH, and PAP-TM-helices is suggested to enable the periplasm-to-inner-membrane signaling leading to the activation of the NBD-ATPase activity on the opposite side of the membrane. This is in stark contrast to the RND transporter assemblies, where major allosteric communications, as well as energy coupling access (in the form of opening of the proton-accessible channel upon substrate-driven L-to-T transition) are restricted to the periplasmic side of the membrane. This is providing a further suggestion that the unique organization of the MacB transporters and their piston-like TM-assembly suggest that their ATPase cycle may differ significantly from that of the classical “clam”-like type IV ABC transporters and the classical alternating access model.

### 9.1 Functional Cycle of the Tripartite MacB-Pumps

The significant conformational changes of the PD/SABRE domains observed in the different apo- and nucleotide-bound structures, as well as the close proximity of the NBDs in some of the nucleotide occupied structures,^24,272,431^ hint at a possible efflux model, which has been dubbed the “molecular bellows model” in analogy to the blowing of the compressed air in the macroscopic analogue. These parallels are enhanced by the existence of the one-way-valve operation of the gating ring of MacA,^272^ with asymmetric energy need for the drug passage in an outward and inward direction on the example of erythromycin MD. Such an arrangement is supposed to prevent backflow from the OMF and suggests that the gating ring is always engaged. Under the emerging model discussed here, the engagement of PAPs with the OMF could be communicated downward to the lipoyl ring, which experiences a twisting motion settling down, and this causes the gating ring to dilate, allowing simultaneous cargo export. While the “bellows model” agrees that the stitching of the “stalk helices” (leading to the formation of the TM1-TM2 bundle) and the associated compression of the supposed substrate binding site is associated with the efflux of the cargo, the exact nature of the power-stroke has been less clear.

Taking into account the ABC transporter cycles discussed in section 5.6.7, we believe that a very modest modification of the initially proposed “bellows” model can unify the operation of the MacB and type IV ABC systems. In parallel to the differential binding of PAP1 and PAP2 to the different RND-conformers, the differential affinity of MacA to the substrate-occupied and empty states of the MacB PCDs could be a driving force for tripartite complex assembly. Such a model may also explain how separation of the two PCD domains of the transporter upon the ATP-hydrolysis may, due to the loose binding of the PAP3, result in a dissociation of the tripartite assembly. Given the existence of the stable crystal and cryo-EM complexes of MacA-TolC, it is tempting to suggest that in contrast to RND complexes, where the PAP-RND forms a more-stable complex, in the MacB-based pumps the PAP-OMF subcomplex may be more stable. In such case a “top-down” assembly of the PAP-OMF onto the MacB may be possible, with this being contingent with the MacB being already substrate-occupied.

**Figure 52:**
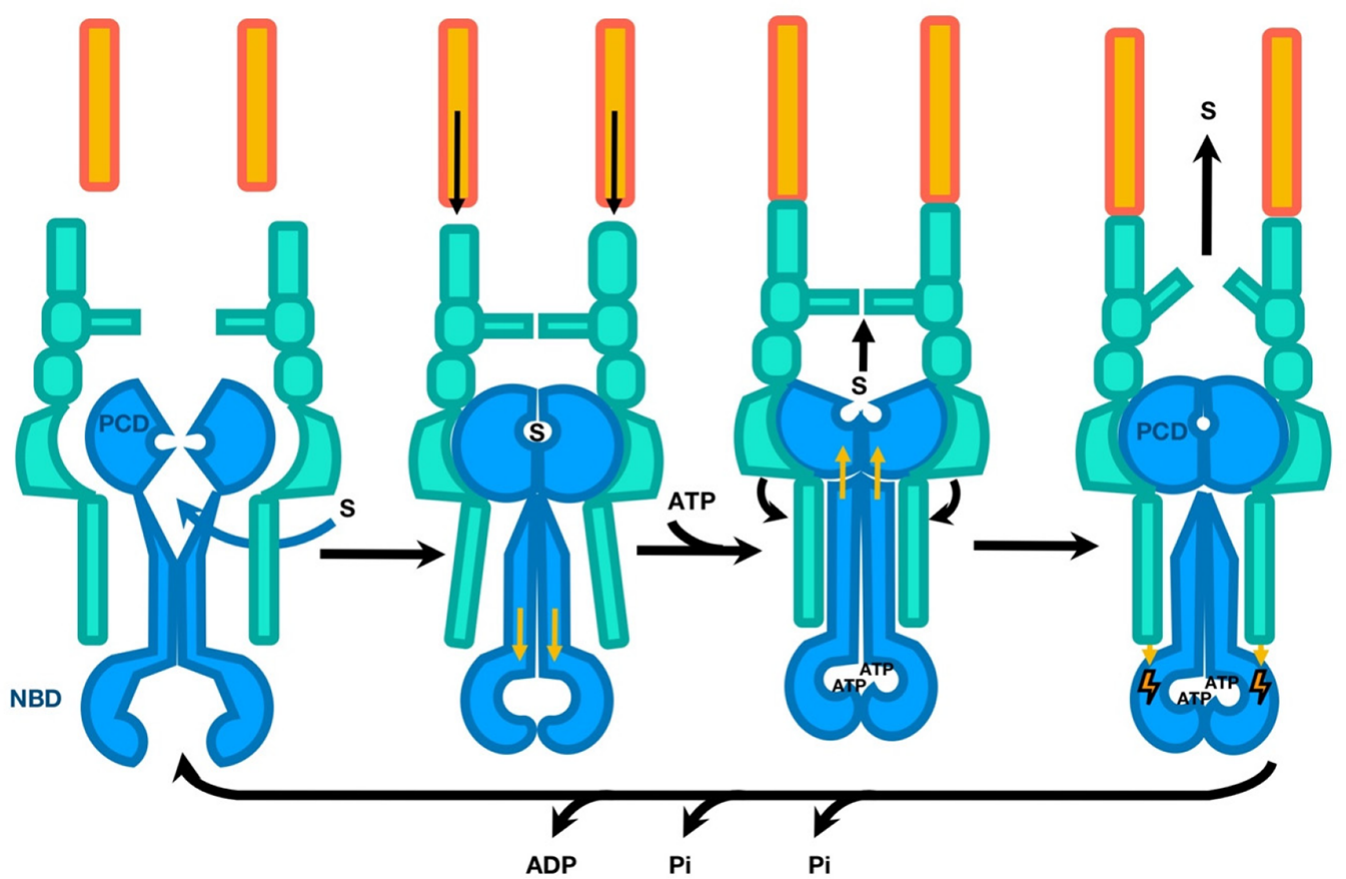
Modified molecular bellows mechanism for the MacAB-TolC pump. MacA (green-cyan) and MacB (blue) undergo allosteric conformational transitions (symbolized by orange arrows) upon binding of substrate and ATP. Similar to type IV ABC transporters, substrate binding is suggested to bring a conformational transition that enables the nucleotide bidning domains (NBDs) to engage with ATP. Substrate is expelled through TolC (orange) upon ATP binding. ATP-hydrolysis (symbolized by the small orange lightning bolts) resets the pump, in order for another cycle of efflux to proceed. Interactions within the membrane between the TM-domains of the MacA and MacB are suggested to provide an additional level of transmembrane communication. See text for further details.

Under our modified bellows model, illustrated in Figure 52, the substrate binding is the primary driving force of tripartite assembly, and similar to the situation discussed in the RND-PAP association, the MPDs of the MacA-type PAPs are suggested to have a strong preference to the substrate-bound conformer of the PCDs, with such binding likely being associated with the recruitment of the OMF.

Indeed, based on the structural homologies discussed above (see the PD-domain connection section), it is plausible that the PCDs may undergo significant conformational changes upon substrate binding, e.g., similar to the PD-transitions seen in the Super *F* to *I* -form of the SecDF transporter.^366^ This initial substrate-binding is suggested to bring the PCDs closer together, creating a conformation analogous to the substrate-bound occluded conformation seen in the type IV transporters, e.g., during the TmrA cycle.^524^ This allosteric transition is communicated via the stalk helices downward across the membrane, enabling the binding of ATP by the NBDs.

In turn, the dimerization and allosteric changes in the NBDs, caused by ATP-binding, result in further tightening of the transmembrane helical bundle, which is communicated back to the PCDs and likely provides the power stroke that leads to the ejection of the substrate into the PAP-OMF cavity above the crown of the transporter, by stabilizing the outer-facing conformation of the PCDs in agreement with the previously proposed models.^431^ Under this scenario, the substrate engagement is the main driver of PCD rearrangement, leading to the initial stitching of the TM1 and TM2, facilitating the ATP-engagement by the NBDs, with the power-stroke being associated with the ATP-binding, rather than ATP-hydrolysis.

The departure of the substrate causes the closure of the outer-facing conformation and produces a stable, ATP-bound, occluded conformation of the transporter, that requires ATP-hydrolysis to drive the NBDs apart. We further suggest that the rearrangement of the PCDs and the TMD of the transporter is sensed by the MPDs and TM-domains of the PAPs, providing additional allosteric signaling. Our modeling suggests that upon ATP-binding by the NBDs, the rearrangement of the transporter TMD allows the MacA-TMs to pack better against the helical core of the TMD, which is likely associated with their ability to convey an ATPase-stimulatory signal (possibly via their interaction with the connecting helix (CoH)), which would explain the functional data.^573,743,835^

Under such a scenario, there could be a possibility of a single ATP-hydrolysis independent cycle of the transporter. Indeed, exactly such an event has been reported in the closely related LolCDE system, the ABC-components of which are virtually identical to MacB.^838^ Uniquely, LolCDE can be purified in a liganded form, which is an intermediate of the lipoprotein release reaction, and it has been demonstrated that the lipoprotein transfer from liganded LolCDE to the periplasmic chaperone LolA in a detergent solution does not require ATP-hydrolysis and a single transfer event can be stimulated by vanadate. The close homology of the two systems suggests that a similar operation may be in place with MacB-based transporters which will support the ATP-binding power-stroke hypothesis.

However, an alternative mode of operation could not be fully ruled out, as recent analysis of MacAB-TolC incorporated into liposomes containing quantum-dots suggested close synchronization of the substrate efflux with ATP-hydrolysis. Furthermore, using the same system did not detect any transfer in the vanadate-treated sample.^839^ While a power-stroke associated with ATP-hydrolysis may be an explanation for such behavior, a closer analysis of the MacAB-assembly provides one intriguing alternative. As mentioned previously, the crown formed of the β-barrel gasket and the lipoyl domain gasket is much-more loosely associated with the transporter than in the case of RND-based assemblies, and it presents a domelike structure, which along with the gating-loops of the lipoyl domain (Figure 50 and Figure 52) creates a “waiting room” type of cavity, where the substrate would plausibly be transferred after the PCD-contraction. The ATP-binding causing the “occluded” substrate-free state of the PCD is suggested to cause contraction of the cavity, squeezing the substrate through the “one-way valve” formed by the gating loops of the lipoyl domain,^272^ and may serve as the trigger initiating the NBD-activation and resetting of the complex. However, taking into account the additional allosteric communication between the PAP TM-domains and the NBDs, their engagement with the ATP-bound NBDs may cause additional twist in the lipoyl domain, which may facilitate the passage of the cargo through the gate. Upon ATP-hydrolysis, the gating loops equilibrate back into the closed state, preventing substrate back-sliding.

Thus, to answer all these questions conclusively, a *bona fide* substrate-bound structure of MacB in complex with the PAP is required, along with functional analysis, and given the rapid progress achieved in the past few years, we are hopeful that this riddle will be resolved soon.

## 10 Tripartite Assembly of the MFS-Based Systems

Tripartite assemblies based on the MFS transporters remain the least-well studied and correspondingly least-well understood, with even complex stoichiometry being a matter of debate. Similar to other PAPs, *E. coli* EmrA in isolation was found to form dimers, and as the dimerization was more pronounced in the full-length, membrane bound protein, it was suggested to take place via a leucine zipper domain located within the N-terminal part of the protein, covering part of the TM-helix domain.^760^ Removal of the N-terminal portion of the protein impacted dimerization even when a major part of TM-helix and membrane anchoring was preserved, suggesting the cytoplasmic N-terminal tail is essential. Later surface plasmon resonance (SPR) measurements^697^ have suggested a trimer formation in agreement with the sequential trimerization (ST)-model, with participation of preassembled dimers with a *K*_d_ of 1.87 μM. The EmrA trimers were also found to be the prevalent cross-linked species.

Although an experimental structure of EmrB is not available at present, reasonably reliable topological estimates could be obtained from the analysis of the structures of the related MFS pumps such as YajR,^382^ EmrD (PDB ID 2GFP),^380^ and PepTSo (PDB ID 4UVM).^271^ The C-terminal part of EmrB likely presents a helical arrangement equivalent of the TM14 of the PepTSo,^271^ and earlier studies suggested that this TM region of the MFS transporter may be involved in a leucin-zipper like interaction with the TM-domain of the PAP EmrA, forming a TM-helical bundle.^760^ A similar suggestion was extended to the N-terminal portion of the transporter, covering a major part of TM1, with the implication of a 2:1 PAP to transporter subunit interaction,^760^ and although current structural data on the organization of TM-helices in the known MFS transporters does not lend itself to a straightforward model of such an assembly, as it requires another PAP-binding site per MFS-protomer, such overall arrangement is appealing as (assuming a dimeric transporter MFS) it gives rise to six PAPs and a 34 TM-helical model (six from PAPs and 28 from the transporter-dimer), which is roughly equivalent to the 36 TM-section observed in the RND-assemblies. Despite their low resolution, the latest EM data on the complete reconstituted EmrAB-TolC^9^ is compatible with such an arrangement, and earlier EM data based on isolated EmrAB has suggested that it forms a “dimer of dimers” in a back-to-back formation;^840^ however, at present a model with a monomeric transporter with six PAPs giving a 20 TM-helix assembly could not be excluded entirely.^738^ A possible hint for such an assembly, where one EmrA is being permanently associated with the EmrB and an additional pair is being recruited, is suggested from the SPR studies which resulted in the ST-model of trimerization mentioned above.^697^

There is limited data available on the potential substrate role in the assembly; however, PAPs from MFS-associated systems are capable of binding drug specifically, providing substrate differentiation as demonstrated for EmrA and HmrA (46.2% identity), and binding to native pump substrates, e.g., CCCP, DNP, and nalidixic acid, provokes conformational changes within the EmrA, which however does not respond to erythromycin, which causes a similar effect in the erythromycin efflux-competent HmrA.^760^ Cargo-specific effects have also been demonstrated for the VceA, a PAP component in the equivalent MFS-based assembly VceABC in *Vibrio*.^122^ This binding occurs in the periplasmic domain and appears to be dependent on the presence of the TM-domains, as no binding was detected to the soluble, TM-truncated versions of the *Ee*EmrA and *Ae*EmrA tested,^738^ providing strong indication that functional TM-domains are needed for such a function. Thus, the existence of extended TM-domains in this class of PAP and their implied engagement in transporter association provide a plausible model for allosteric control and activation.

While future work is needed to elucidate the exact nature of the PAP-MFS interaction, it is clear that the mode of operation of these assemblies is markedly different from the RND- and MacB-based systems, due to the lack of extended periplasmic domains. Consistent with this, the PAPs engaged with the MFS transporters also lack MDP domains, and their TM regions are essential for dimerization and engagement with the transporter.^760^ The rigidity of the extended α-helical hairpin domains also likely provides less allosteric control of the cycle, with the stability of these assemblies being higher than the corresponding RND systems,^697^ and they might be engaged in a constitutive low-level efflux, rather than being engaged temporarily for high-intensity stress-related responses, which is also consistent with their much lower copy numbers in the cell.^696^

## 11 Assembly of Type 1 Secretion Systems

**Figure 53:**
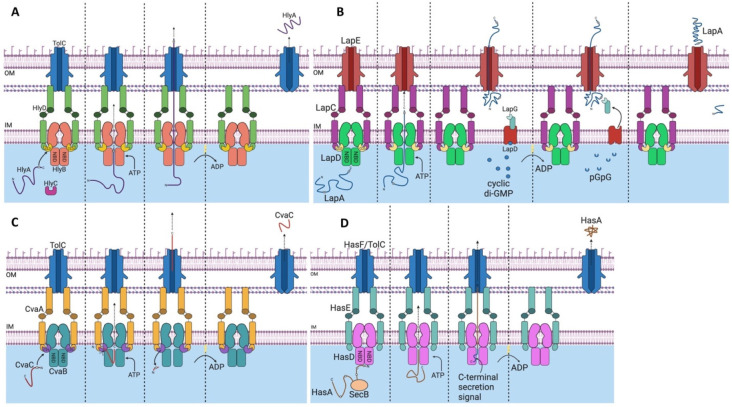
Principal modes of T1SS depending on the cargo. (A) Typical RTX-toxin secretion system based on the HlyABD-TolC. The complex is assembled upon substrate binding and remains stable through a number of iterative ABC-cycles by the HlyB transporter. There is no periplasmic intermediate of transport and no processing of the cargo (HlyA), with the CLD-domains of HlyB (in yellow) playing a cargo-recruitment and chaperoning role. (B) An example of the transport by the bacterial transglutaminase-like cysteine proteinase (BTLCP)-associated transporter family on the example of the LapBCDEG system. Notably, the complex disassembles upon association of the cargo (LapA) with the OMF, where it may be anchored for extended periods by its N-terminal “plug” domain, creating a periplasmic intermediate. While the CLD of the LapB does not proteolytically process the cargo, this is achieved by a periplasmic protease LapG, which is controlled by a cyclic di-GMP receptor LapD in response to environmental stimuli providing control of the cell adhesion. (C) The microcin-based secretion system is exemplified by the CvaABC. The PCAT transporters associated with this type of secretion have catalytically active C39 domains. (D) The HasDEF system of *Serratia marcescens* involved in the secretion of the hemophore HasA presents a departure from the common pattern presented in panels A–C, where the cargoes are fed C-terminus first and instead HasA is threaded N-terminus first. Furthermore, the system lacks both C39 and CLD domains and relies on the chaperoning function of the SecB. Additional details are provided in the main text. Figure modified based on Smith et al.^6^ and Masi and Wandersman.^512^

The cargoes for the type 1 secretion systems (T1SSs) are secreted in a Sec-independent fashion^6,841^ and correspondingly lack the N-terminal signal-sequence typical of periplasmic bound cargoes of the Sec-system.^842^ There are two broad ranges of cargoes secreted by the T1SSs.^843,844^ The first includes relatively small molecules with a molecular weight in the sub-10 kDa range that are secreted in a single step, such as bacteriocins (in the context of Gram-negative bacteria, microcins), e.g., colicin V (aka microcin V; CvaC).^845^ Microcin secretion is facilitated by an N-terminal signal, which is recognized by the C39-domain of the cognate PCAT-type ATPase forming the core of the transporter complex and is then proteolytically processed concomitantly with the secretion of the cargo. This has been discussed in the preceding sections on the example of PCAT1,^505^ so to avoid repetition we will not discuss this group further in great detail. A schematic of their transport is presented in Figure 53, panel C.

The second diverse group of cargoes are generally larger proteins, some of which are among the largest coded within the bacterial proteome and include a number of toxins, adhesins, lipases, and proteases.^449,846,847^ Rather uniquely, the targeting signal for the cargo secretion in this second group is located within the 50–60 residues of the C-terminal.^848−850^ As the nature of this signal is quite diverse and reveals no clear sequence consensus, it has been suggested that secondary structure is at least partially involved.^851^ It appears to present a set of two short α-helical fragments bridged by a linker peptide, with a downstream low-complexity region.^852,853^ The secretion of some of the cargoes within this group relies solely on this C-terminal signal, e.g., the well-studied heme acquisition protein HasA.^529,854^ A major group of the cargoes secreted by the T1SS present additional Ca^2+^ binding motifs known as RTX-motifs or GG-repeats—GGXGXDXUX (where X is any amino acid and U is a hydrophobic amino acid)—precede the C-terminal secretion signal in some of the passenger proteins, including HlyA and LipA.^449,497^ These play an important role in folding of the cargo, and X-ray structures reveal that they form a β-roll motif (e.g., PDB ID 1KAP(500,501)). Within the RTX-group, there is significant diversity of cargoes, which can be ranked in the order of organizational complexity. The hemolysin/cytolysin family of pore-forming toxins includes hemolysin A (HlyA), in which the region N-terminal of the glycine-repeats code just one functional domain—the pore forming toxin itself^214^ and the CyaA from *Bordetella pertussis*,^855^ which apart from the toxin domain, possesses an adenylate cyclase module which affects the cAMP levels upon entering the target host cell. More complex architectures are present in the so-called MARTX (multifunctional autoprocessing repeats in toxins), which contain protease domains and target eukaryotic cells, where they undergo autoprocessing upon binding to inositol hexakisphosphate (InsP6).^847,855,856^

Another large group is the large repetitive RTX adhesins (e.g., FrhA of *Vibrio cholerae*, RtxA of *Legionella pneumophila*, and SiiE of *Salmonella enterica*), which function in biofilm development and cellular adherence and attain very large sizes, sometimes in excess of 1.5 MDa, e.g., in the case of the *Mp*IBP of *Marinomonas primoryensis*.^449,856,857^ These are critical molecules for biofilm formation, and they feature a number of repetitive domains, in addition to the RTX repeats; for example, the *Mp*IBP possesses some 120 bacterial immunoglobulin-like domains (BIg) and a unique ice-binding domain, which is associated with the lifestyle of this bacterium.^857^

The majority of the T1SS appear to translocate their cargoes from the cytoplasm to the extracellular space in a single translocation event,^858^ without disassembly of the complex or any periplasmic intermediates. While the details of the secretion are still not fully confirmed and vary between different assemblies, in a first approximation the process features the following key elements (Figure 53, panel A). The typical RTX cargo (e.g., α-hemolysin) is fully synthesized prior to secretion and is threaded in an unfolded state, C-terminus first. The C-terminal part of the protein carrying the RTX motifs emerges on the cell surface and binds to Ca^2+^ ions to facilitate the folding of the jellyroll RTX domains, which contributes to the secretion process.^214^

As a first step, the RTX-cargo is recruited by the ATPase, and the secretion motif is recognized by the catalytically inactive C39-like (CLD) domain of the PCAT transporter,^506^ which feeds the C-terminus of the cargo into the substrate-binding cavity of the transporter, keeping it unfolded. This association leads to the assembly of the complete translocase and causes a conformational switch in the transporter, allowing for the outer-facing conformation to be attained, which brings the NDBs together, allowing them to bind the ATP^721^ (Figure 53, panel A). ATP-hydrolysis in turn resets the system back for another cycle, continuously threading the cargo, and these cycles continue until the N-terminal end is reached. Following the release of the cargo outside of the cell, the complex disassembles. This is in contrast to the secretion of the microcins (Figure 53, panel C), where the PCAT has an active C39 domain and the secretion signal is N-terminally located on the cargo, which also gets proteolytically processed. Importantly, and in common with the RTX-toxins depicted in panel A, the transfer of the cargo takes a single step.

Recently, a group of RTX-containing proteins related to the RTX-adhesion proteins has been identified and separated into its own class, named, due to linkage to the bacterial transglutaminase-like cysteine proteinase (BTLCP),^859^ BTLCP-adhesins.^6,860^ The prototype of this BTLCP-adhesin group, LapA of *Pseudomonas fluorescens* Pf0-1, also possesses a number of BIg motifs but also a novel, specialized N-terminal “retention module” to anchor itself at the cell surface as a secretion-intermediate threaded through a dedicated OMF LapE (Figure 53, panel B). In addition, they often feature von Willebrand factor A domains (vWFA), which are peptide-binding folds, also found in *Pseudomonas putida* LapA and LapF.^860,861^ The first stage of the transport involves the association of the cargo with the CLDs of the LapB ABC transporter, thus resembling the HlyBCD system. However, a startling discovery has been made, showing that these adhesins break the dogma of “one-stop-transport” which has been associated with T1SS, and instead their translocons are able to dissociate before the adhesin has left the OMF barrel, releasing the PAP transporter pair^860^ (Figure 53, panel B).

Indeed, the very nature of the “retention module” or “plug domain” is to embed the adhesin within the OMF-channel, where it may stay permanently, unless processed by a periplasmic protease, LapG. The stalled LapA functions as a biofilm-promoting factor, which is dependent on the cyclic-di-GMP levels, as the LapG protease is sequestered in the presence of cyclic-di-GMP by a membrane-anchored cyclic-di-GMP receptor LapD but can be released once the levels of linear di-GMP (pGpG) increase^862^ (Figure 53, panel B). Thus, while the actual transporters associated with the T1SS of BTLCP-linked adhesins do not possess active proteolytic sites, their cargoes are proteolytically processed in response to environmental stimuli.

Intriguingly, all the PCAT transporters associated with three groups of T1SS presented so far have N-terminal domains, which are implicated in substrate binding. However, while the CvaB transporter has a C39-domain with an active catalytic site, both the transporters associated with the transport for pore-forming cytolysins (HlyB-type PCATs) and LapB-type transporters associated with the secretion of the BTLCP-linked adhesins have catalytically inactive CLDs. These however differ dramatically in their consensus sequences, suggesting a different mode of operation and cargo specificity.^6^

A surprisingly similar arrangement was also discovered in the secretion of the giant adhesin *Mp*IBP,^857^ where two dedicated modules were found to anchor the adhesin to the inside of the OMF-channel, as a stable periplasmic intermediate, suggesting that this is a more widespread mechanism than previously thought.

This is achieved, as in contrast to the RTX modules, which require a Ca^2+^ concentration of above 3 mM (typically associated with the extracellular medium) to fold, the N-terminal “retention modules” of LapA, and the corresponding N-terminal “plug” domains, and β-barrel spanning domains seen in the *Mp*IBP are structured in the absence of Ca^2+^ and capable of folding during the secretion process, allowing them to engage and anchor themselves with the OMF partner proteins.^6,497^

While markedly divergent, the different T1SSs discussed above present a uniting feature, which is that all of their cargoes are transporter C-terminal first. The implication of this characteristic is that the cargo polypeptide needs to be synthesized in its entirety prior to secretion and may be expected to become at least partially folded. Unless active steps are taken to the contrary by chaperoning systems, and as discussed in the earlier section on the PCAT transporters, this is provided by both the transporter cavities themselves and their associated CLD-domains and in some cases auxiliary chaperones.^214^

In addition to the cargo interaction with the PCAT transporter itself, which is at the heart of the T1SS and was covered in the preceding sections, the second major-driving force behind the assembly of a functional T1SS is the PAPs. Their role appears to be rather multifaceted and involves direct binding of the cargo, stimulation of the assembly process, and recruitment of the OMF, as well as possibly participation in the folding of cargoes. Distinct regions of the PAPs seem to play a different role in the process. Many of the studies into the role of the PAPs in T1SSs derive from the hemolysin A (HlyA) secretion system in *E. coli*, which involves the PCAT-type ATPase HlyB and its cognate PAP HlyD and the OMF TolC.^61^

As mentioned in the previous sections, the transport of HlyA does not involve proteolytic processing,^849^ and correspondingly, the C39-domain of the HlyB is catalytically inactive and is referred to as a CLD.^506^ The regions covering the N-terminal section of the α-helical hairpin domain (residues 127 to 170) and the C-terminal 33 residues of HlyD, respectively, were shown to be required for *in vivo* secretion.^863^ HlyD was also shown to interact directly with the toxin substrate by direct cross-linking, and in addition, masking its C-terminal fragment by in-frame fusion to a c-Myc epitope blocked cargo translocation,^721,863^ suggesting an active role of the PAP in substrate recognition and its presentation to the transporter.

Furthermore, the N-terminal 59 residues of HlyD form a small cytoplasmic domain,^731,864^ truncation of which abolished the secretion of HlyA.^865^ A more detailed analysis of the region, including a dissection of the residues (1–25) forming a predicted amphiphilic helix and a downstream charged region (residues 26–38), demonstrated that these regions are necessary for direct interaction of the PAP with its cargo (HlyA) and subsequent recruitment of the OMF into the functional translocator but not required for either the oligomerization of HlyD or the formation of the PAP/ABC transporter complex.^866^ This data also suggests that HlyA binding to the cytosolic domain of HlyD promotes an outward trans-membrane conformational change, propagated to the periplasmic domain of HlyD, leading to the recruitment of the OMF.

The periplasmic portion of the PAP has also been implicated in cargo-related function, and analysis of random point mutations in PAPs, in particular HlyD^755^ and related PAP CvaA, involved in colicin V secretion^754^ demonstrated that they result in misfolding of the substrates, even when their secretion rate was not affected. These mutations map to the lipoyl domain and the α-helical hairpin of HlyD, and their impact of cargo-folding suggests that the α-barrel of the hexamerised PAP may provide a chaperoning function.^755^ Similarly, TolC barrel mutations have been reported to result in incorrect folding of substrate HlyA affecting its hemolytic activity,^867^ which could be restored by refolding *in vitro*. Furthermore, by analyzing a variety of passenger proteins spliced in front of the HlyA C-terminal signal sequence and introducing folding mutations into the cargo, Bakkes et al.^530^ were able to demonstrate that the efficiency of secretion of HlyA by the *E. coli* HlyBD-TolC is dictated by the folding rate of the substrate.

Importantly, the formation of the inner-membrane HlyBD translocase complex is not just initiated by the substrate but is also ATP-independent, as demonstrated by the use of a HlyB mutant competent in ATP-binding but not in ATP-hydrolysis.^721^ This mutant was capable of ATPase-PAP assembly, substrate recruitment, and bridging of the OMF; however, HlyA stalled in the channel. A similar result was obtained when the PAP HlyD C-terminus was masked. This leads to the conclusion that HlyA export likely occurs via the assembly of a contiguous double-membrane channel which is formed without the requirement for ATP-hydrolysis by the PCAT transporter, by substrate-induced, transient, and reversible bridging of the HlyBD-translocase to the OMF.^721^

In the aforementioned protease secretion system PrtDEF (see PCAT ABC transporter section 5.6) from *Dickeya dadantii* (*formerly Erwinia chrysanthemi*),^531^ responsible for the secretion of a number of proteases, including PrtA, PrtB, PrtC, and PrtG,^532^ the ABC transporter PrtD displays a basal ATPase activity that is specifically inhibited (half-inhibition at 0.1 μM) upon encountering a cognate C-terminal secretion signal of its substrates (PrtB and PrtG metalloproteases) but not by the C-terminal sequences from nonrelated cargoes.^868^ It is thought that this inhibition may take the form of a stericprevention of NBD closure in a manner similar to the inhibition of eukaryotic TAP1/2 by the viral peptide ICP47,^869^ which forms a long-helical hairpin plugging the translocation pathway of TAP from the cytoplasmic side stalling the system.^515^ This inhibited conformation of PrtD binds the PAP PrtE, with the complex of PrtD and PrtE recruiting the OMF PrtF.^492^ Thus, this system presents a clear ordered sequence of assembly, where the substrate first recognizes the PCAT-ABC transporter, which then binds the PAP assembly, which in turn recruits the OMF component.^492^ Notably, while substrate binding was shown to be required for assembly of the three components, there was no evidence for ATP being a driver of the assembly.

A related T1SS in *Serratia marcescens*, formed around the PCAT type transporter HasD, and the PAP HasE are involved in the secretion of the heme acquisition protein HasA^153,870^ (Figure 53D). In *E. coli*, the reconstituted HasA secretion system was dependent on the presence of an OMF channel, which could be either TolC^871^ or the recombinant PrtF, the OMF from the *Dickeya dadantii* T1SS PrtDEF. The analysis of the secretion of the *Serratia marcescens* HasA protein by the HasD indicated a strong coupling between synthesis and secretion in the type 1 secretion pathway^529^ due to slow threading of the substrate, suggesting active maintenance of the cargo folding is involved, and indeed folded HasA has been found to inhibit its own secretion. Primary interactions between HasD and newly synthesized HasA have been suggested to be driven by linear sites which are sequentially exposed on the unfolded cargoes.^512^ ATP-binding promotes dimerization of the NBDs, facilitating and stabilizing the outward facing transporter conformation, and the substrate is then fed into the recruited OMF N-terminus first, while presumably the C-terminus stays associated with the transporter until the threading is complete. These data support a model of type 1 secretion involving a multistep interaction between the substrate and the PCAT transporter that stabilizes the assembled secretion system until the C-terminus of the cargo is presented, and such interaction is suggested to underwrite the multistep processivity required for translocation of large, unfolded cargoes. Crucially, the C-terminal signal of the cargo hemophore HasA was shown to trigger ATP-hydrolysis, driven by the PCAT ABC transporter HasD, leading to disassembly of the complex and separation of OMF (HasF) to its pre-engagement state.^512^ In agreement with this, it was reported that the C-terminal peptide of HasA may also possess an intermolecular activity that is capable of triggering complex dissociation *in vivo* when it is provided as a distinct peptide.^872^ This appears to be a departure from the general model of type 1 secretion discussed above (Figure 53A–C), where the C-terminal part is driving the secretion with a contribution from folding of the RTX-motifs. Furthermore, HasA is not proteolytically processed and its cognate ATPase HasB does not possess a CLD-domain. While at 19 kDa HasA presents a relatively small cargo, it still is necessary for it to be transported in an unfolded state, which is facilitated by SecB on the cytoplasmic side.^873^ Thus, HasA secretion appears to present a distinct mode of T1SS as presented in Figure 53D.

Despite their considerable variety, an emerging common pattern across all the T1SS assemblies discussed above is that the ATP-hydrolysis does not appear to be required for the formation an ABC transporter–PAP–OMF complex, which instead is substrate and PAP-driven,^512,721,866^ while the C-terminal signal of HasA triggers HasD-driven ATP-hydrolysis, leading to disassembly of the T1SS complex.^512^ Such energy dependency also provides further parallels between the assembly and disassembly of the PCAT-based T1SS tripartite complexes and the tripartite complexes arranged around RND transporters discussed above,^705,720,813^ with the interprotomer association being driven by protein–protein interaction and substrate-binding/cargo folding without the requirement of energy input, while the energy-dependent step is likely required for resetting and disassembly of the complex. As discussed in the previous section, similar energy-dependency for complex dissociation has also been described for a MacB-related LolCDE complex, where membrane-extracted lipoproteins are passed over to the periplasmic chaperone LolA,^838,874^ in a process that requires ATP-binding and hydrolysis but not the conformational change associated with the release of the ADP and Pi (as it could be trapped by vanadate), suggesting that a full cycle of ATP-hydrolysis is similarly required for complex disassembly.

Despite the recent advances in the understanding of the functional organization of the T1SS, the structural data on these systems lags compared to other tripartite assemblies. However, the recent availability of all principal components of the T1SS machinery allow for some tentative reconstructions of the complete assembly.

**Figure 54:**
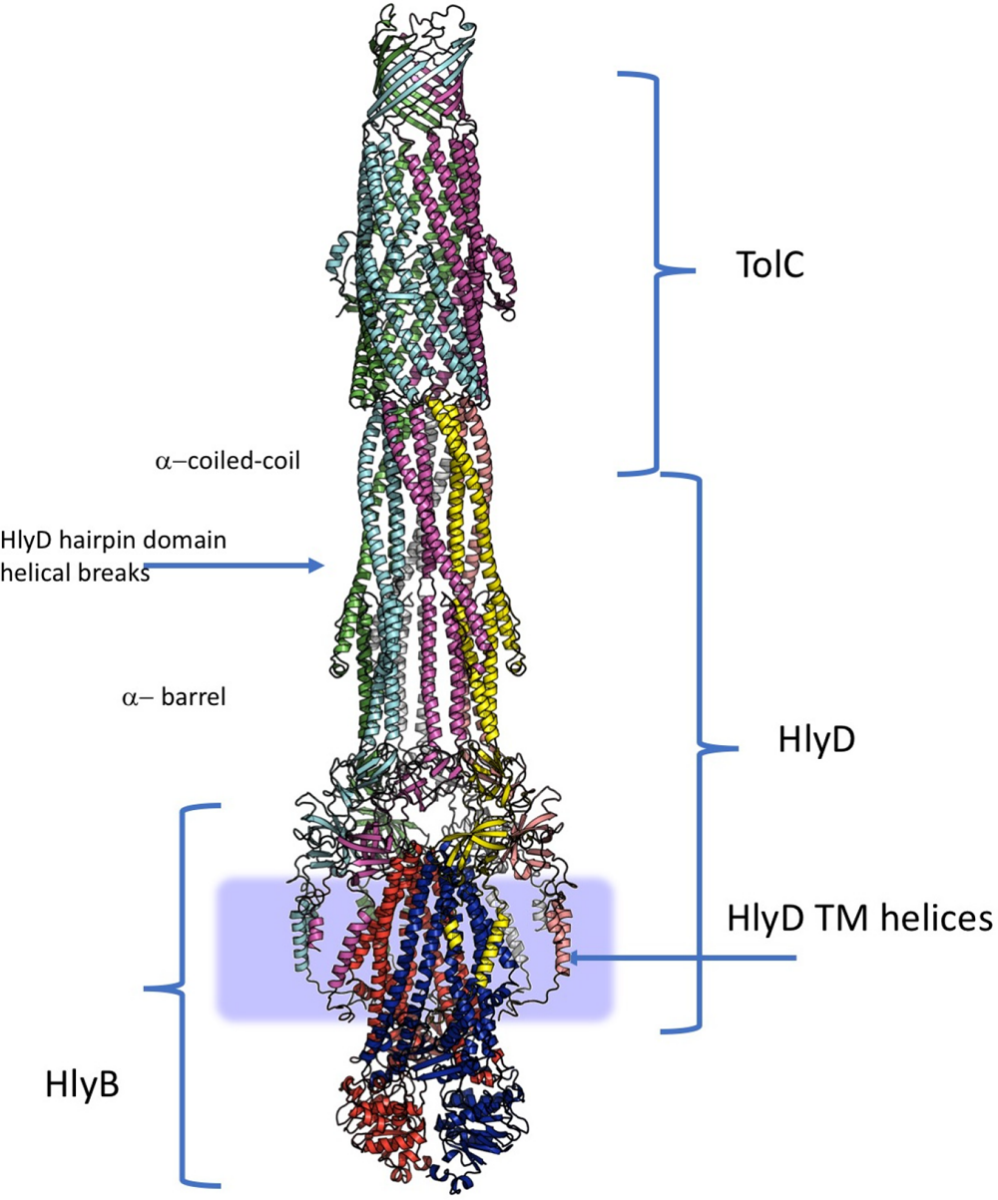
Qualitative model of the complete tripartite T1SS assembly based on the HlyBC-TolC. The assembly is notable for the extended tubular architecture, which presents a unique break in the supercoiling pattern, associated with the architecture of the T1SS PAPs. The hexameric β-barrel ring of HlyD binds closely to the membrane due to absence of the MP-domains, and the PAP-associated transmembrane helices could provide interactions within the membrane. The approximate membrane boundaries are shown with the blue rectangle. See the text for more details.

Previous attempts stopped short of modeling the complete assembly, and to gain some insights into its possible organization, here we have extended the modeling of the HlyAB-TolC system to include both the full HlyD transporter (based on the homology with PrtD and PCAT1 structures)^505,515^ and the β-barrel and the transmembrane domains of HlyD, which have not been resolved in the crystal structure^739^ (Figure 54). The overall assembly is compatible with the available functional data^752,755^ and suggests a unique helical trajectory of the HlyD, which mimics the helical paths of the TolC, and arrangement that is facilitated by the helical breaks in the hairpin domain of HlyD. The modeling of the extended TM-helices of HlyD suggests, similar to the MacAB-model presented earlier, that they may be able to participate in direct intermembrane interactions with the TMD of the HlyB, possibly providing additional allosteric control and modulation of the ATPase activity which could explain the previously accumulated functional data. While the near future will no doubt provide experimental proof of such models, they provide the best current approximation of the function of the T1SS.

## 12 Targeting Tripartite Assemblies—Pump Inhibitors and Disruptors

**Figure 55:**
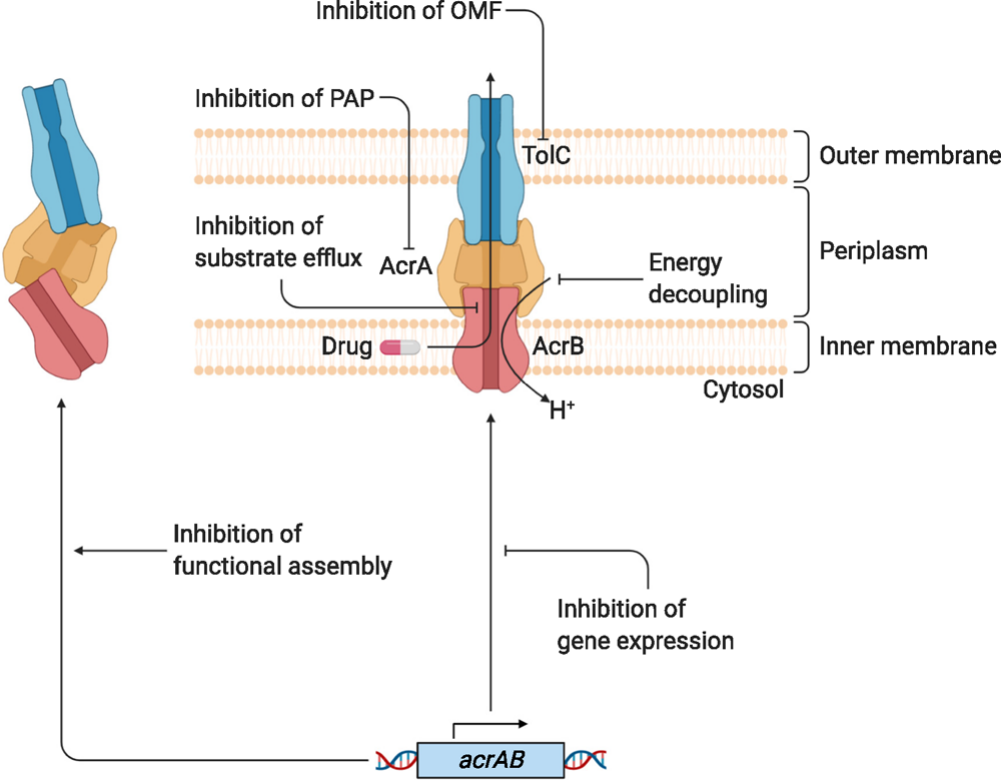
Schematic diagram of the different mechanisms of action of efflux inhibitors. Inhibition of gene expression prevents transcription of efflux genes, thereby reducing efflux pump expression. Compounds that inhibit the functional assembly work by interfering with the interaction surfaces between the components of the tripartite efflux systems. Energy decoupling agents facilitate flow of protons, thereby dissipating the electrochemical gradient, so that it cannot be exploited as an energy source by efflux pumps. Inhibitors of substrate efflux can work as a competitive inhibitor by actively competing with the binding site to prevent substrate binding or by blocking conformational change to prohibit enzymatic catalysis. Inhibitors of periplasmic adaptor proteins (PAP) are thought to work by preventing binding to the inner membrane transporter and by preventing self-association of the PAP protomers. Outer membrane factor (OMF) inhibitors block the periplasmic entrance site to prevent translocation of substrates across the outer membrane. PAP, periplasmic adaptor protein; OMF, outer membrane factor.

The growing threat of antibiotic resistance has significantly reduced the efficacy of existing antibiotics. Therefore, there is an urgent need to develop new therapeutic approaches to treat infections caused by MDR bacteria.^875^ The manifold roles of tripartite efflux systems in Gram-negative bacteria make them attractive drug targets; therefore, efflux inhibition is seen as a prospective strategy to overcome efflux-mediated resistance. Compounds that inhibit the function of efflux pumps directly or indirectly are known as efflux inhibitors (EIs). Although the term efflux pump inhibitor is also used in the literature, EIs is deemed more accurate since not all compounds interact directly with efflux systems to inhibit efflux. The primary rationale for using EIs is to restore the susceptibility of multidrug resistant (MDR) bacteria to antibiotics by preventing their extrusion, thereby increasing intracellular concentrations of antibiotics.^876^ EIs could also be used to revive the effectiveness of older antibiotics that have fallen out of use due to their loss of efficacy against MDR bacteria. Furthermore, due to the reported roles of tripartite efflux systems in biofilm formation and virulence, EIs could also be employed as antibiofilm and antivirulence agents.^185,877^ For a compound to be classified as an EI, it must fulfill two important criteria. First, an EI should not exhibit antibacterial activity, but when used in tandem with an antibiotic, it should potentiate the activity of that particular antibiotic. Second, an EI should not possess activity against a strain that lacks the efflux pump that it is supposed to inhibit. Lastly, an EI should not be a substrate of efflux pumps.^878^ Importantly, EIs should be able to pass across the outer membrane into the periplasm to exert their activity. The chemical diversity of idenitifed EIs has been recently reviewed in detail elsewhere.^879,880^ Studies have found that EIs can exhibit different mechanisms of action (Figure 55), and here we review the literature on this basis.

### 12.1 Targeting the Inner Membrane Transporter

**Figure 56:**
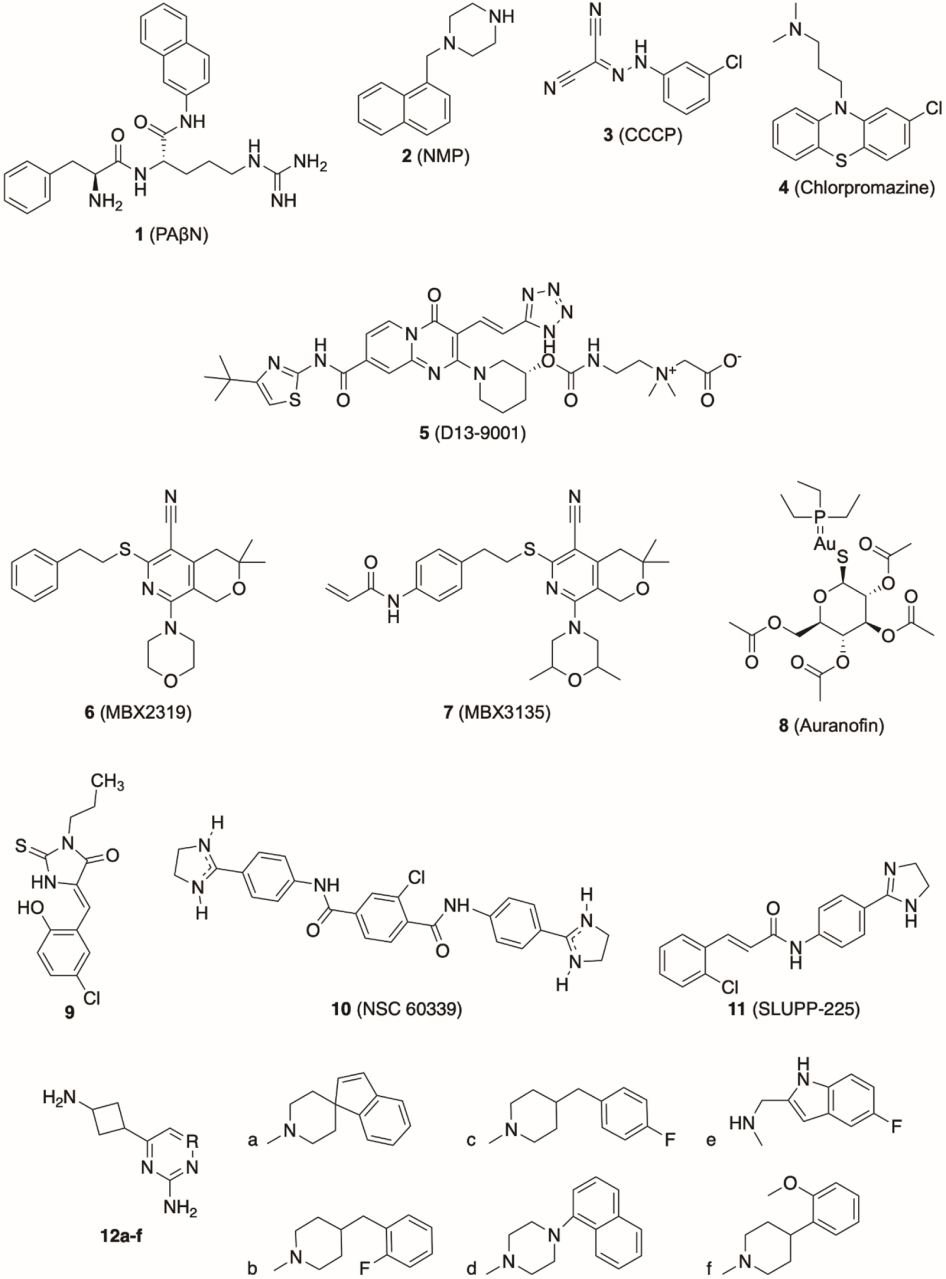
Structural formulas of representative efflux inhibitors that have been discussed in this review.

The inner membrane component of tripartite systems plays an important role in energy transduction, substrate recognition, and uptake. Therefore, the majority of EIs described in the literature are posited to target the inner membrane transporter, such as AcrB. One of the first EIs to be characterized was phenylalanine-arginine β-naphthylamide (PAβN, **1**; Figure 56), a synthetic peptidomimetic inhibitor of the RND family tripartite efflux systems MexAB-OprM, MexCD-OprJ, and MexEF-OprN of *P. aeruginosa*.^881^ Since then, PAβN has been shown to be a broad-spectrum inhibitor of several different tripartite RND efflux systems in Gram-negative bacteria, including AcrAB-TolC from *S.* Typhimurium, and CmeABC from *C. jejuni* and *C. coli*.^50,882^ PAβN has been suggested to act as a competitive inhibitor of RND efflux systems by binding to the distal binding pocket, specifically the hydrophobic trap, to prevent the binding of substrates.^343^ Surface plasmon resonance (SPR) studies have shown that PAβN binds to both AcrA and AcrB with a micromolar affinity.^883^ Lamers et al.^884^ also reported that PAβN can permeabilize the outer membrane of Gram-negative bacteria, indicating an additional mechanism of action for PAβN. A recent study reported that PAβN exerts its EI activity in AcrB by restraining drug-binding pocket dynamics rather than acting as a competitive inhibitor. Computational and experimental data suggested that ciprofloxacin and PAβN bind to AcrB simultaneously at different subsites within the distal binding pocket and that PAβN inhibits efflux by perturbing the substrate translocation pathway.^885^ PAβN was tested *in vivo* to be cytotoxic.^886^ However, it is still one of the most commonly used inhibitors in assays to restore the activity of various antibiotics. One of the other early broad-spectrum EIs to be identified was the synthetic compound 1-(1-naphthylmethyl)-piperazine (NMP, **2**; Figure 55), which was shown to potentiate the activity of several antibiotics, including chloramphenicol, levofloxacin, and linezolid, in *E. coli* strains that were overexpressing *acrAB* or *acrEF*.^887^ In addition, NMP has been shown to potentiate the activity of antibiotics in several other Gram-negative bacteria, such as *A. baumannii, K. pneumoniae, C. freundii*, and *K. aerogenes*.^888−890^ SPR studies have shown that NMP binds to AcrB with a higher affinity than PAβN, even though they act as EIs at the same concentration,^891^ which could be explained by the additional membrane-permeabilizing effects of PAβN. To date, the exact mechanism of action of NMP has not been elucidated. However, molecular dynamics simulations suggest that NMP binds to the outer face of the distal binding pocket to straddle the G-loop of AcrB, thereby reducing its flexibility and impairing the proper binding of substrates.^343,349^ Further development of this compound was discontinued due to its low potency and structural similarity with serotonin agonists.^892^ Another strategy to inhibit inner membrane transporters is by depleting the energy source required for efflux. Carbonyl cyanide *m*-chlorophenylhydrazone (CCCP, **3**; Figure 56) is a protonophore that dissipates the proton motive force, thus inhibiting all secondary active transporters. Due to its toxicity, CCCP has no therapeutic potential; however, it is a useful experimental tool as it is widely used in efflux assays.^893^

Certain clinically approved drugs have also been reported to display EI activity. The psychotropic drug chlorpromazine (**4**; Figure 56) has been reported to inhibit efflux in Gram-negative bacteria.^894^ In *S.* Typhimurium and several other serovars, chlorpromazine was shown to significantly potentiate the activities of various antibiotics. In the same study, chlorpromazine was reported to repress *acrB* expression, suggesting that it may inhibit efflux by reducing efflux gene expression.^895^ Chlorpromazine can also affect cellular morphology and replication, membrane permeability, and energy generation, suggesting additional mechanisms of action.^894^ A recent study has shed some light into the mechanism of how chlorpromazine acts as an EI. Grimsey et al.^896^ reported chlorpromazine and amitriptyline to be substrates and inhibitors of the AcrB pump. Exposure to subinhibitory concentrations of chlorpromazine in *E. coli* and *S.* Typhimurium was found to select for *ramR*- and *marR* mutants, which encode for the regulators of the AcrAB-TolC system. Additionally, it was reported that when *S.* Typhimurium cells with a nonfunctional AcrB pump (D408A mutation) were exposed to subinhibitory chlorpromazine and amitriptyline concentrations, the D408A *acrB* mutants were found to revert to a wild-type *acrB* allele, suggesting that both compounds act as substrates of AcrB. Lastly, molecular docking and dynamics simulations and free energy calculations were shown to demonstrate that both chlorpromazine and amitriptyline bind to the hydrophobic trap within the distal binding pocket of AcrB.

Through high-throughput drug screening and rational drug design, new and more potent EIs have been identified. A pyridopyrimidine (D13-9001, **5**; Figure 56) derivative acts as an inhibitor against the MexAB-OprM pump in *P. aeruginosa*(347) and AcrAB-TolC of *E. coli* and was co-crystallized with AcrB (*E. coli*) and MexB (*P. aeruginosa*).^291^ This compound was shown to bind to the deep binding pocket area, which was designated the hydrophobic trap due to the enrichment of phenylalanine residues in this region. Furthermore, isothermal titration calorimetry was used to show that D13-9001 binds to AcrB and MexB with binding affinities of 1.15 μM and 3.5 μM, respectively.^291^ MBX2319 (**6**; Figure 56) is another pyranopyridine inhibitor of the AcrAB-TolC system which was identified through high-throughput screening of 183,400 compounds. It was shown to potentiate the activity of multiple antibiotics against *E. coli*.^348^ MBX2319 binds tightly to AcrB;^349^ however, it does not display any detectable binding affinity to AcrA as verified by SPR.^883^ Later, molecular docking studies led to the development of more potent derivatives of MBX2319, such as MBX3135 (**7**; Figure 56). Pyranopyridine derivatives (MBX2319, MBX2931, MBX-3132, and MBX-3135) were found to be highly potent in the inhibition of efflux pumps in *E. coli*, *E. aerogenes, K. pneumoniae*, and *S. enterica*.^348,349,897^ Crystal structures of the MBX inhibitors (MBX2319, MBX2931, MBX-3132, and MBX-3135) were obtained with the soluble periplasmic domain of AcrB (AcrBper) and were found to bind in the proximity of the same region in the hydrophobic trap.^341^ The inhibitory effect was suggested to be due to the competitive and overlapping binding of the inhibitors to the deep binding pocket of the T protomer, and due to the tight interaction, it possibly halts the conformational LTO cycling. Interestingly, single-particle cryo-EM structures revealed an LTO trimeric setup in the presence of substrate (puromycin), whereas in the presence of MBX-3135, mostly particles in the TTT conformation were found.^287,305^ A recent study reported the identification of 43 novel EIs of Gram-negative bacteria from a high-throughput drug screening of the Prestwick drug library and the proprietary compound library from Roche. It was found that the majority of identified EIs had a specific EI–antibiotic combination and/or species-specific synergy with antibiotics. Of the EIs identified, auranofin (**8**, Figure 56), an antirheumatic agent, increased ethidium bromide accumulation and potentiated antibiotic activity in checkerboard assays.^898^ While most drug screening efforts have focused on looking at inhibitors of RND inner membrane transporters, a recent study identified an inhibitor of the ABC type efflux pump MacAB in *S.* Typhimurium. Compound **9** (Figure 56) was identified from screening a drug library for compounds that inhibited the growth of a *macAB*-overexpressing strain in the presence of erythromycin. It was reported to increase the susceptibility of this strain to several different macrolides, although its mechanism of action remains to be explored.^899^

The EIs discussed so far, and indeed the majority of the EIs designed and tested so far, target the transporter component of the system, notably the RND transporters, within which the majority of the substrate specificity is thought to reside. However, there is growing evidence for the involvement of PAP and OMF partners in contributing to the selectivity, making them possible targets for competitive inhibition. A recent study by Marshall and Bavro^718^ found several antibiotic-specific phenotypes associated with novel point mutations in TolC from *E. coli*. For example, Q129L and Q352E mutations in TolC were shown to increase susceptibility to only tetracycline and acriflavine, respectively. Another recent study found that the MexXY RND pump of *P. aeruginosa* exports anionic β-lactams, such as carbenicillin and sulbenicillin, when it forms a tripartite complex with OprA but not with OprM. Interestingly, both MexXY-OprA and MexXY-OprM tripartite complexes can extrude aminoglycosides, whereas OprA confers an expanded substrate profile.^665^ This suggests a new role for OMF components of tripartite efflux systems in the vetting of substrates. In other studies, the PAPs ZneB and CusB of the tripartite ZneCAB and CusABC RND efflux systems, respectively, have been reported to contribute to the metal ion specificity of the two systems.^46,360^ Thus, these components may also present themselves as possible chemotherapeutic targets.

### 12.2 Targeting the Periplasm Adaptor Protein

Although the bulk of EIs described in the literature are reported to target inner membrane transporters, the PAP component of tripartite systems has also been shown to be a viable target for efflux inhibition. Abdali et al.^883^ carried out the parallel experimental and structure-based screening of two drug libraries to identify inhibitors of AcrA, the PAP component of AcrAB-TolC. Several EIs were identified and shown to bind to AcrA, change its structure *in vivo*, and potentiate the activities of several antibiotics in *E. coli* and other Gram-negative bacteria. Additionally, the identified EIs, including NSC 60339 (**10**; Figure 56), were found to require AcrA binding to inhibit efflux activity. Further optimization efforts of compound **9** lead to more potent derivatives, such as SLUPP-225 (**11**; Figure 56), which was shown to penetrate the outer membrane of *E. coli* cells and exhibit more potent efflux inhibition activity.^900^ Later, Darzynkiewicz et al.^901^ investigated the potential binding sites of compound **9** and found that it likely binds to a site situated between the lipoyl and β-barrel domains of AcrA. The β-barrel domain of AcrA has been previously shown to be involved in RND binding, and the lipoyl domain is involved in self-association; therefore, AcrA inhibitors may function to prevent the functional assembly of AcrAB-TolC.^902^ In a recent study, Green et al.^903^ searched the ZINC15 database for compounds with physicochemical properties matching those of existing EIs and Gram-negative antibiotics. Subsequently, compounds with matching criteria were computationally docked onto AcrA of *E. coli* at the previously reported binding site of compound **9**, as well as several other sites that when disrupted have been reported to disrupt efflux. The top 34 hits from all docking poses were then analyzed using a combination of *in vitro* binding assays to measure binding affinity to AcrA and AcrB and *in vivo* antibiotic potentiation assays. They identified six compounds (**12a–f**, Figure 56) with a shared scaffold that bound to AcrA within the millimolar range as verified by SPR. Given that the compounds had weak binding affinities, it is possible that they bind to AcrA weakly at multiple sites. Promisingly, the six compounds also potentiated the activities of erythromycin and novobiocin in hyperporinated *E. coli* cells, as well as in wild-type *A. baumannii* and *K. pneumoniae*. However, PAPs are promiscuous, which must be taken into account when targeting PAPs to inhibit efflux. McNeil et al.^902^ demonstrated that in *S. enterica* inactivation of AcrA results in AcrE compensating for its loss, indicating interchangeability between the two PAPs. Therefore, a successful PAP inhibitor would need to target both AcrA and AcrE in *S. enterica* to inhibit efflux.

### 12.3 Targeting the Outer Membrane Factor

The OMF component of tripartite efflux systems plays an important role in expelling substrates across the outer membrane to the external environment. As a paradigm OMF, TolC is rather promiscuous and functions with several different tripartite efflux systems, including T1SS, RND, ABC, and MFS efflux systems. Despite being an attractive drug target, TolC inhibition is relatively under-researched. Higgins et al.^693^ reported the crystal structure of TolC in complex with hexaamminecobalt (HC), a trivalent cation, and showed that it blocked the periplasmic entrance of TolC by forming salt bridges with D374 of each TolC monomer. Later, Gilardi et al.^904^ investigated the biophysical interaction of HC with TolC and found that it binds to TolC with a high affinity and fast association and dissociation rates. Furthermore, HC was found to not exhibit any intrinsic antimicrobial activity nor potentiate antibiotic activity in *E. coli*. Thus, further research is required to identify more potent TolC inhibitors capable of inhibiting efflux and potentiating antibiotic activity. However, TolC inhibition may not be a good target for efflux inhibition in practice. Krishnamoorthy et al.^653^ found that the activity and assembly of the AcrAB-TolC complex in *E. coli* can tolerate substantial fluctuations in the amount of TolC available. Furthermore, they reported that only a small fraction of intracellular TolC is necessary for the efflux requirements of *E. coli* cells, indicating that significant depletion of TolC is well tolerated in bacterial cells.

### 12.4 Other Strategies to Inhibit Efflux

Although chemical inhibition of efflux is predominantly reported in the literature, there have been recent developments in inhibiting efflux through different strategies. One example is using antisense oligomers to reduce or inhibit efflux pump protein translation in a sequence specific manner. Peptide-conjugated phosphorodiamidate morpholino oligomers (PPMOs) are an example of synthetic nucleotide oligomers that inhibit antisense mRNA translation. Ayhan et al.^905^ developed and identified an *acrA*-PPMO capable of increasing the efficacy of several antibiotics, such as cefotaxime, chloramphenicol, clindamycin, and fusidic acid, by up to 40-fold and preventing AcrA protein translation in a dose-dependent manner in *E. coli*. Additionally, the *acrA*-PPMO was shown to be effective in *K. pneumoniae* and *S. enterica* by significantly increasing the activity of piperacillin-tazobactam. More recently, an optimized *acrA*-PMMO was shown to potentiate the activity of azithromycin and levofloxacin in several clinical Enterobacteriaceae strains. Crucially, the *acrA*-PPMO was demonstrated to increase the efficacy of azithromycin *in vivo* in a *K. pneumoniae* septicemia murine model. Inhibition of MexAB-OprM, the homologous RND system in *P. aeruginosa*, was also reported to enhance the efficacy of several antibiotics. PPMOs targeting *mexA* and *mexB* were found to enhance the activities of azithromycin, cefotaxime, and piperacillin-tazobactam by at least 4-fold in the majority of tested clinical *P. aeruginosa* isolates.^906^ In a different study, antisense oligonucleotides were developed to inhibit the translation of the *cmeA* gene, which encodes for the periplasmic adaptor protein CmeA of CmeABC in *C. jejuni*. The *cmeA* antisense oligonucleotide was reported to sensitize *C. jejuni* cells to ciprofloxacin and erythromycin at very low concentrations.^907^

Another strategy for inhibiting efflux is through CRISPR (clustered regularly interspaced short palindromic repeats)-Cas (CRIPSR-associated protein)-mediated genome editing. Owing to the versatility of CRISPR-Cas, it has also been demonstrated to sensitize bacteria to antibiotics by targeting antibiotic resistance genes, such as β-lactamases.^908^ A recent study demonstrated that CRIPSR-Cas can also be used to delete efflux genes. As a proof of principle, Xu et al.^909^ used native CRISPR-Cas in *P. aeruginosa* to delete the *mexB* efflux gene, thereby sensitizing cells to several antipseudomonal antibiotics. Although in its infancy, the CRIPSR-Cas system has the potential to specifically target the sequence of bacterial efflux pump genes, thereby avoiding the problem of toxicity associated with off-target effects of classical EIs. Peptide-based approaches have also been explored as a means to inhibit tripartite efflux systems. By exploiting the transmembrane AcrB TM1–TM8 protein–protein interface, Jesin et al.^910^ designed synthetic peptides to mimic TM1 or TM8 of AcrB to interfere with AcrB trimerization. Using Nile Red efflux assays and antibiotic susceptibility testing, TM1 and TM8 peptides were shown to significantly reduce efflux activity and potentiate the activity of antimicrobials. Targeting protein–protein interactions between components of tripartite efflux systems to prevent complete assembly could be another strategy to inhibit efflux.

## 13 Conclusion

The Gram-negative double membrane is a formidable barrier across which many molecules must be transported. As shown above, bacteria have addressed this challenge by evolving a diverse array of tripartite efflux pumps and type 1 secretion systems, many of which play important roles in the export of xenobiotics and toxic compounds, including antibiotics and a number of other cargoes, including very large virulence factors and adhesion molecules.

We have shown that despite the apparent diversity in structure, function, and role between the complexes there are commonalities between seemingly disparate systems. In particular, our comparative analysis has highlighted the roles of the periplasmic adaptor proteins, indicating that far from being passive connectors they in fact are active architects of the assembly and couple allosteric transitions between the inner membrane transporters and outer membrane factors.

In the case of the tripartite systems which utilize proton motive force, the association of the PAPs with the respective RND- and MFS-based transporters allows these assemblies to combine two different electrochemical gradients across two separate semipermeable membranes in a unique way. This is in stark contrast to classical transporters that perform transport of substrate and counter-transport of ions in opposite directions by coupling their respective electrochemical gradients created across a single membrane.

Unifying the wealth of functional data with the recent advances in the structural biology of tripartite assemblies allows us to propose an integrated model of their assembly and functional cycling. The importance of many of these tripartite systems in resistance to antibiotics and also in virulence makes them attractive targets for the development of inhibitors. While much progress has been made in this field, we believe that a better understanding of the structure–function relationship of individual efflux systems alongside an integrated view across families of these systems will accelerate progress toward finding clinically useful inhibitors.

## 14 Remaining Challenges and Future Directions

Despite recent progress, there are still some persistent questions that remain unanswered within the field. As our understanding of the basic structure of the isolated components of tripartite pumps has grown, the research focus direction has been shifting toward the complete assemblies, including specifically the control of their assembly and disassembly, which could be targeted for therapeutic purposes.

One clear tendency is moving away from *in vitro* studies toward understanding the more dynamic aspects of their function and regulation, especially in the context of the cell. This includes on one hand the structural aspect, e.g., where the potential of future use of cryo-electron tomography has been recently demonstrated and the resolution of the reported *in situ* protein assemblies is rapidly increasing. On the other hand, it also applies to the functional characterization of these assemblies, where significant headway has been made by creation of functional reconstitution assays in native membrane environments. In combination with the rapidly developing field of transportomics, these developments promise to accelerate the characterization of substrate specificity, as well as the screening of potential efflux inhibitors (EIs) targeting these assemblies.

These technologies also promise to address the remaining questions in the energetics of the pump assembly and cycling; as e.g. the H^+^/drug stoichiometries of RND assemblies remain to be established and are difficult to address *in vivo*, they thus would benefit from the controlled environments and sensitivity afforded by such studies. Similarly, the exact nature of the power-stroke and efficiency of the cycle for ABC transporters, which remain a much-discussed subject, could hopefully be addressed by a combination of structural studies of trapped intermediates and functional analysis.

Modularity of the assemblies, in the form of pumps presenting e.g. heterooligomeric IMPs or PAP components, could potentially provide a way to impose regulation on the efflux systems. This is most likely kinetic regulation. While cooperative kinetics have been shown for AcrAB-TolC, its implication for the cell physiology is obscure. One possible explanation for that could be the high energy consumption of a permanently active pump, resulting in wasteful drainage of the proton gradient. In the case of lower concentrations of the drug, a more moderate pace would save energy, whereas at high drug concentrations the pump is fully activated. However, clear experimental proof of the above model is still awaiting.

While the increased understanding of interprotomer interactions within the RND complexes has allowed the development of the integrated model above, the exact roles of PAP1 and PAP2 protomers remain to be fully validated experimentally. The existence of systems such as TriABC, which utilize separate PAPs, also poses the question of their biological function and the benefits that these provide for the bacterium. While our current models of the tripartite complex suggests that the transporters and OMFs do not interact directly and that such interaction is solely moderated by the PAPs, it is difficult to reconcile these models with the existence of PAPs such as BesA which lack their hairpin altogether, suggesting alternative modes of interaction and assembly are possible, if even in just a subset of tripartite assemblies.

Also, while understandably the RND transporters have attracted the lion’s share of research interest over the past decade, the understanding of the mechanisms of the other tripartite assemblies is still lagging behind, and notably the questions of the transmembrane allostery in the T1SSs and MacB-based systems needs to be elucidated. As shown above, full understanding of the transport cycles of these assemblies could be achieved by studying the transporters in isolation due to the close involvement of the PAP in their function, which has been largely ignored to date.

Given the broad substrate specificity of the multidrug efflux pumps, it is unclear why they are presented in so many versions in the bacterial genomes. The overlapping specificities and joint action of the pumps, especially in the context of multiple antibiotic stress, need to be clarified. The understanding the synergistic effects of the activity of the members of the different (super)families is also a rapidly developing field and will likely benefit from the wider deployment of the technologies listed above.

Antimicrobial resistance is a current global health emergency, and tripartite efflux pumps are an important mediator of this resistance. Efflux pumps underpin many mechanisms of resistance; for example, they not only directly lower the intracellular concentration of antibiotic drugs but also reduce the frequency at which mutations conferring resistance via other mechanisms are selected. One significant future challenge will be in developing new drugs that are not subject to resistance via efflux. There has been significant progress in understanding which drugs can be transported by respective efflux systems and the specific residues involved in their transport. In the future, a complete understanding of substrate export will be needed to enable development of drugs that cannot be transported by major efflux pumps and for which selection of simple mutations does not permit development of resistance.

The concept of inhibitors to potentiate the use of existing and novel antibiotics holds a lot of promise, and there is also evidence they may be useful as antivirulence or antibiofilm compounds. However, no EIs have yet successfully reached the clinic largely due to issues with either potency or toxicity. Development of effective and safe EIs is an enormous future challenge, but better understanding of the structure, assembly, and mechanism of these systems will aid rational drug design approaches and rationalize the mechanism of compounds detected through high-throughput screening approaches. As much of the current effort has been targeting transporters, the promise of inhibitors targeting either OMFs or PAPs or interfering with assembly of the functional pump has yet to be fulfilled.

Efflux pumps and secretion systems are increasingly important for biotechnological and industrial applications. Efflux pumps are critical for increased export of synthesized products to aid isolation and in other cases for providing resistance to toxic products which can then increase yield. In contrast to the medically related future challenges, in this sphere, work to optimize transport of specific products is needed to improve the efficiency of industrial and biotechnological applications, and this will rely on a future better understanding of substrate export. Better understanding of the molecular determinants of the interprotomer recognition and the promiscuity of the assembly may allow the creation of “designer pumps” with *a priori* desired specificities.

## Supporting Information Available

Molecular morphing movie illustrating a possible transition from the OMF-free state to the OMF-engaged state (zip)
